# A single cell *Arabidopsis* root atlas reveals developmental trajectories in wild type and cell identity mutants

**DOI:** 10.1016/j.devcel.2022.01.008

**Published:** 2022-02-07

**Authors:** Rachel Shahan, Che-Wei Hsu, Trevor M. Nolan, Benjamin J. Cole, Isaiah W. Taylor, Laura Greenstreet, Stephen Zhang, Anton Afanassiev, Anna Hendrika Cornelia Vlot, Geoffrey Schiebinger, Philip N. Benfey, Uwe Ohler

**Affiliations:** 1 Department of Biology, Duke University, Durham, North Carolina 27708, USA; 2 Department of Biology, Humboldt Universität zu Berlin, 10117 Berlin, Germany; 3 The Berlin Institute for Medical Systems Biology, Max Delbrück Center for Molecular Medicine, 10115 Berlin, Germany; 4 Department of Energy Joint Genome Institute, Walnut Creek, California 94598, USA; 5 Department of Mathematics, University of British Columbia, Vancouver, V6T 1Z2 British Columbia, Canada; 6 Department of Computer Science, Humboldt Universität zu Berlin, 10117 Berlin, Germany; 7 Howard Hughes Medical Institute, Duke University, Durham, North Carolina 27708, USA; 8 These authors contributed equally to this work; 9 These authors contributed equally to this work; 10 Corresponding authors; 11 Lead contact

## Abstract

In all multicellular organisms, transcriptional networks orchestrate organ development. The *Arabidopsis* root, with its simple structure and indeterminate growth, is an ideal model to investigate the spatiotemporal transcriptional signatures underlying developmental trajectories. To map gene expression dynamics across root cell types and developmental time, we built a comprehensive, organ-scale atlas at single cell resolution. In addition to estimating developmental progressions in pseudotime, we employed the mathematical concept of optimal transport to infer developmental trajectories and identify their underlying regulators. To demonstrate the utility of the atlas to interpret new datasets, we profiled mutants for two key transcriptional regulators at single cell resolution, *shortroot* and *scarecrow*. We report transcriptomic and *in vivo* evidence for tissue trans-differentiation underlying a mixed cell identity phenotype in *scarecrow*. Our results support the atlas as a rich community resource for unraveling the transcriptional programs that specify and maintain cell identity to regulate spatiotemporal organ development.

## eTOC summary

How do transcriptional networks regulate organ development? Using scRNA-seq, Shahan and Hsu et al. produced an *Arabidopsis* root atlas, revealing gradual gene expression changes underlying differentiation of cell types and candidate regulators of cell fate. The atlas enabled interpretation of smaller scRNA-seq datasets and revealed new phenotypes in developmental mutants.

## Graphical Abstract

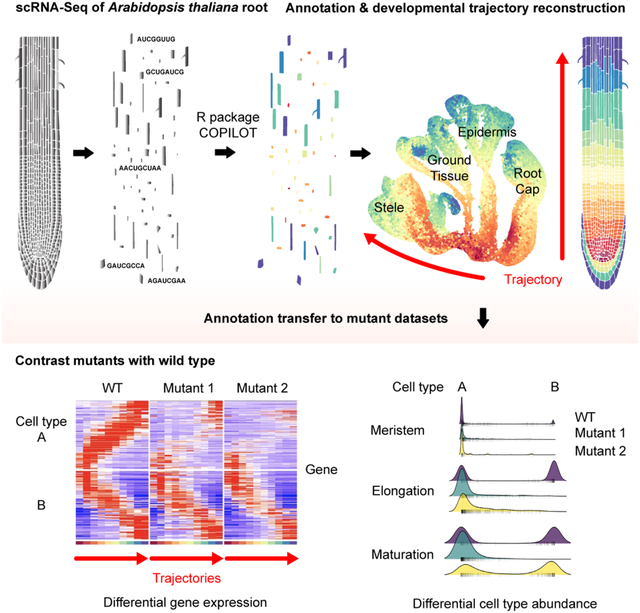

## Introduction

**Figure 1.**
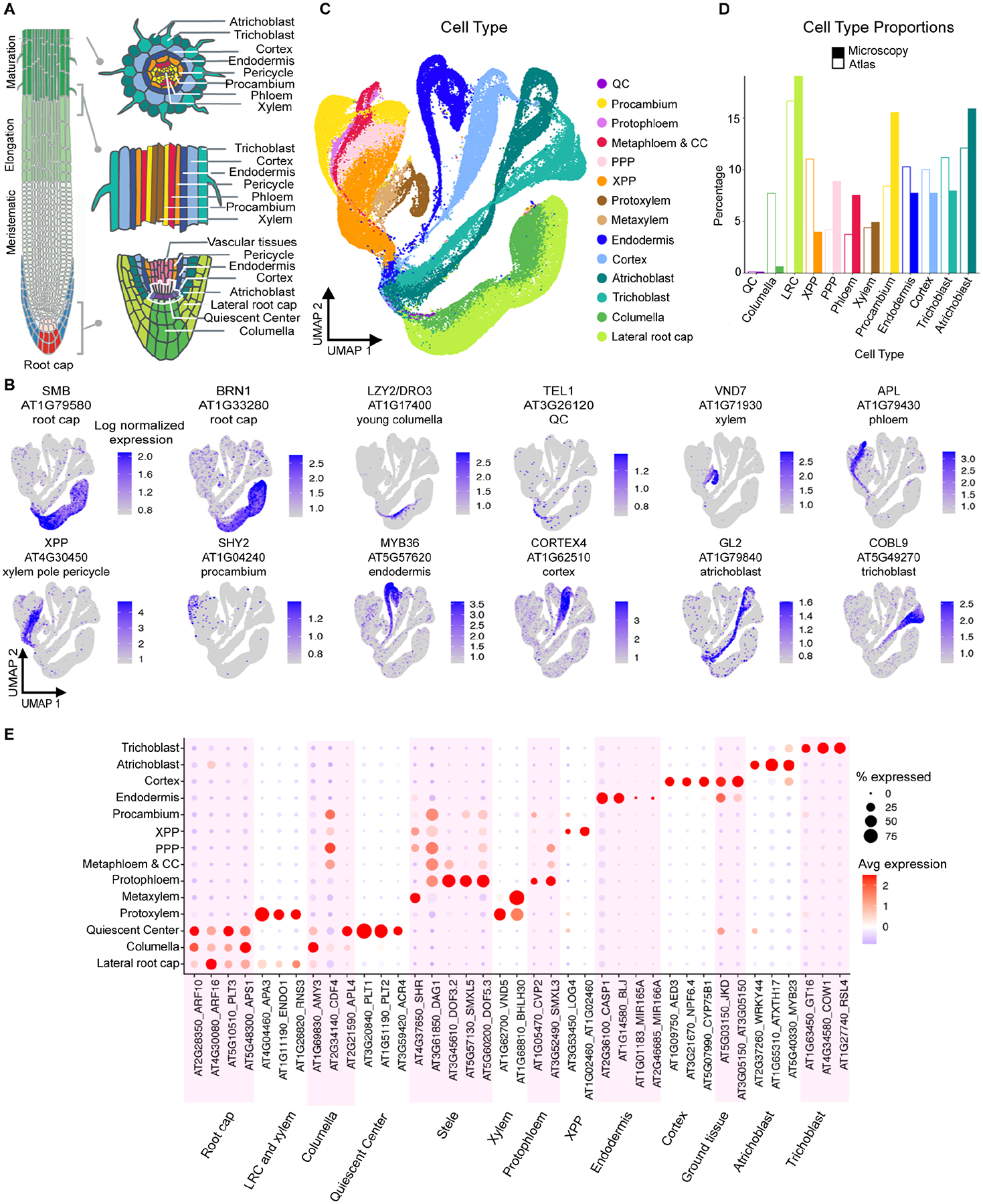
110,427 cell root atlas representing all major cell types. **A)** Developmental zones (left) and radial cell types (right) of the *Arabidopsis* root. White border indicates the location of stem cells surrounding the Quiescent Center. Illustration adapted from the Plant Illustrations repository (Bouché, 2017). **B)** Expression of known cell type markers. The color scale for each plot represents log normalized, corrected UMI counts for the indicated gene. **C)** UMAP with cell type labels. The crossing over or apparent mixture between some cell types, e.g., trichoblast and atrichoblast, is a result of 2D projection and absent in 3D (Supplementary Movie 1). **D)** The proportion of each cell type group in the atlas is comparable to *in vivo* cell type proportions (Cartwright et al., 2009). **E)** Cell type expression for 40 genes, the spatial expression profiles of which have been previously characterized. Dot size represents the percentage of cells in which each gene is expressed (% expressed). Dot colors indicate the average scaled expression of each gene in each cell type group with warmer colors indicating higher expression levels. CC: companion cell; QC: quiescent center; PPP: phloem pole pericycle; XPP: xylem pole pericycle; LRC: lateral root cap. See also Figures S1– S6, Datasets S1– S3, Data S1, and Supplementary Movie 1.

Precisely controlled transcriptional networks specify cell identity, relate positional information, and regulate tissue maturation (Drapek et al., 2017). Defining how these networks orchestrate organ development and function requires detailed knowledge of spatiotemporal gene expression patterns. However, in animal models such as the zebrafish embryo, cells migrate during development and thus present a challenge for cell lineage tracing and subsequent inference of gene expression dynamics (Farrell et al., 2018). The immobile cells and organization of the *Arabidopsis thaliana* root simplify cell lineage tracing and facilitate the study of spatiotemporal organ development (Dolan et al., 1993; Fig. 1A). Cell types are arranged in concentric layers around a central vasculature. Cell lineages are ordered longitudinally along a temporal developmental axis, with the oldest cells closest to the shoot and the youngest cells adjacent to the stem cell niche at the root tip. With each new cell division at the root tip, older cells are displaced shootward from the stem cell niche. Thus, root anatomy simplifies interrogation of the developmental trajectories from stem cell to differentiated tissue (Efroni and Birnbaum, 2016; McFaline-Figueroa et al., 2020).

The *Arabidopsis* root is a tractable model organ with established markers for most cell types as well as expression profiles for morphologically defined developmental stages (Birnbaum et al., 2003; Brady et al., 2007a; Li et al., 2016). Recently, pioneering studies applied droplet-based single cell RNA sequencing (scRNA-seq) to the *Arabidopsis* root and demonstrated the utility of this technology to identify new cell type markers, examine gene expression dynamics across pseudotime, and identify regulators that control cell type-specific responses to environmental conditions (Denyer et al., 2019; Jean-Baptiste et al., 2019; Ryu et al., 2019; Shulse et al., 2019; Zhang et al., 2019; Wendrich et al., 2020). These reports also established foundational principles for root scRNA-seq, including the successful capture of all major cell types from samples prepared from whole roots and the utility of known markers and gene expression profiles to accurately annotate major cell types. However, none of these first-generation atlases combined more than 12,500 cells and only Wendrich et al. (2020) inferred developmental progressions for more than three cell types. Further, each atlas is enriched for a subset of cell types or developmental stages at the expense of others (Fig. S1). Thus, there is currently no comprehensive *Arabidopsis* root atlas that captures a finely resolved spectrum of developmental states for all major cell types.

By contrast, recent developmental studies using animal or human samples profiled hundreds of thousands (Schiebinger et al., 2019) or even millions (Cao et al., 2019) of cells and high temporal resolution was achieved by densely sampling timepoints across development (Briggs et al., 2018; Farrell et al., 2018; Schiebinger et al., 2019; Massri et al., 2021). High pseudotemporal resolution from increased cell numbers provides greater statistical power and enables identification of a finely resolved order of transcriptional events, which is important for considering causal models of gene regulation (Schiebinger et al., 2019). For the *Arabidopsis* root, in which all cell types are represented at all developmental stages, greater pseudotemporal resolution across development will be gained with an atlas that integrates more cells for all cell types and developmental zones.

Here, we present a primary root gene expression atlas with an order of magnitude more cells than previous *Arabidopsis* datasets. Given the continuous nature of cell states represented in our data, we developed a largely cluster-agnostic annotation approach to avoid bias associated with choosing a clustering resolution. In addition to estimating pseudotime progressions for all cell types, we demonstrate the first application of an optimal transport-based method, StationaryOT, to reconstruct developmental trajectories from plant scRNA-seq data. Cell fate probabilities calculated by StationaryOT shed light on how the fate acquisition of each cell type relates to all other major root cell types. Regressions applied to the cell fate probabilities and gene expression data identified known transcription factors (TFs) involved in cell identity and differentiation. Finally, we tested the ability of the atlas to inform new datasets and demonstrated the power of scRNA-seq to identify new developmental phenotypes by profiling two cell identity mutants, *shortroot* and *scarecrow*.

## Results

### Integration of over 110,000 cells produces an organ-scale atlas

To build an atlas, we used the 10X Genomics scRNA-seq platform to profile over 96,000 root cells. We harvested 0.5 cm of tissue from five to seven-day-old primary root tips across thirteen sets of independently grown wild-type (WT) seedlings (Dataset S1). The transcriptional profiles of all samples were highly correlated, suggesting that batch effects such as differences in plant age are unlikely to substantively affect downstream analyses (Fig. S1). Gene expression matrices calculated by kallisto (Bray et al., 2016) and bustools (Melsted et al., 2019) served as input to **C**ell prepr**O**cessing **PI**peline ka**L**list**O** bus**T**ools (COPILOT), our pre-processing software, which incorporates detection and removal of low-quality cells (Data S1; STAR Methods).

To add additional depth and assess lab-to-lab data variability, we selected three published root scRNA-seq datasets (Denyer et al., 2019; Ryu et al., 2019) to combine with data generated in this study (Dataset S1). After excluding genes affected by protoplasting (the process of dissociating plant cells from their cell walls; Denyer et al., 2019) we integrated 110,427 cells into an organ-scale atlas (Fig. S1; Dataset S1; STAR Methods). A median of 2,768 genes were detected per cell with 24,997 total genes detected, representing 90% of the coding genes in the *Arabidopsis* genome.

### Cell annotation places tissues in known developmental contexts

**Figure 2.**
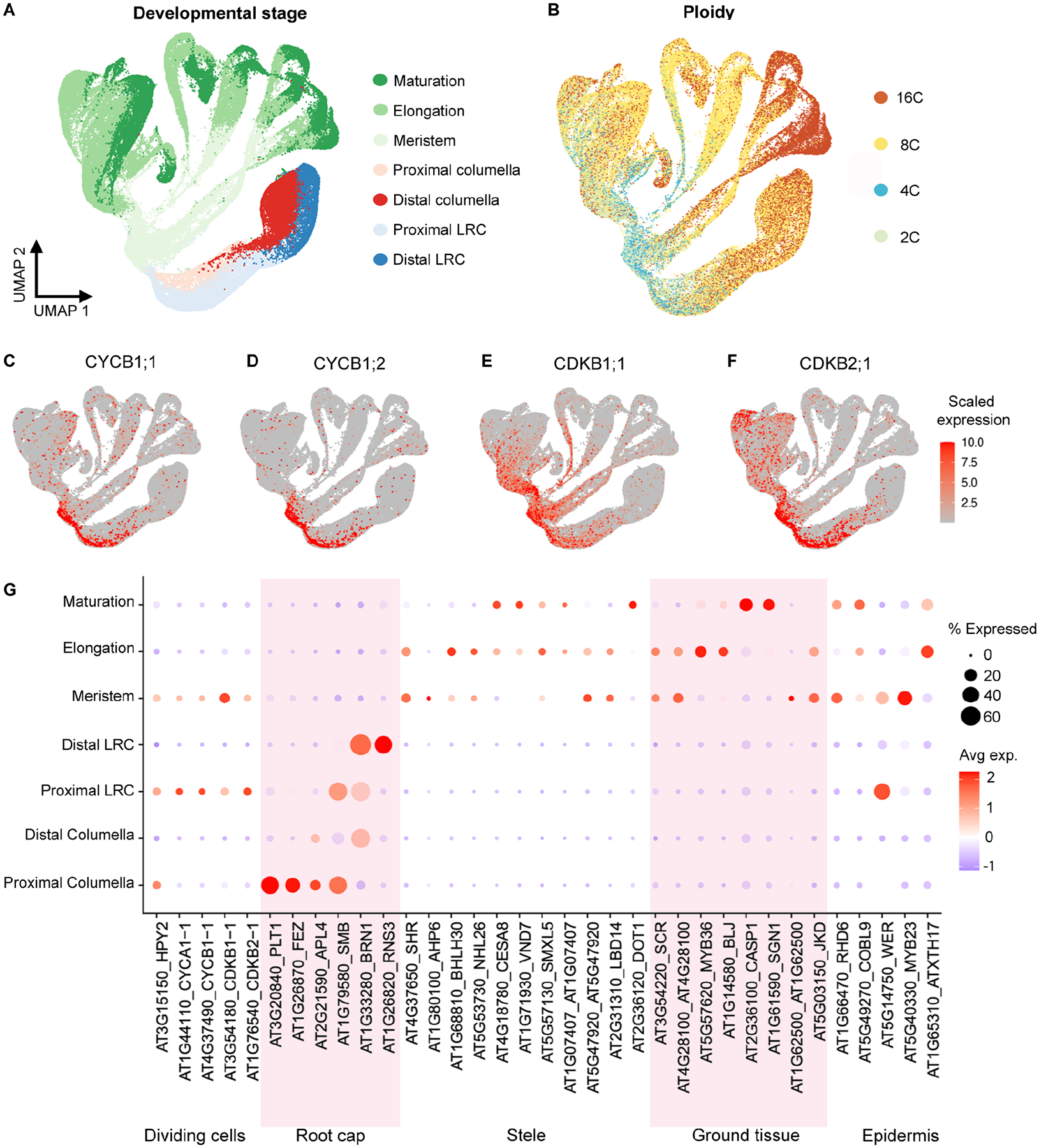
Expression profiles of known genes support the atlas developmental stage annotations. **A)** UMAP with developmental stage annotations. LRC: lateral root cap. **B)** UMAP with cell ploidy annotations based on gene expression profiles from Bhosale et al. (2018). **C-F)** Scaled expression (STAR Methods) of four previously characterized cyclin genes (Ishida et al., 2009). **G)** Developmental stage expression profiles for 35 genes expressed across the four major root tissue types. Dot size represents the percentage of cells in which each gene is expressed (% Expressed). Dot colors indicate the average scaled expression of each gene in each developmental stage group with warmer colors indicating higher expression levels. Root cap: lateral root cap and columella. See also Figures S1– S6 and Datasets S1– S2.

Inspection of marker genes indicated that all major cell and tissue types are discernible as discrete topological features in 2D Uniform Manifold Approximation and Projection (UMAP) space (Fig. 1B). To infer precise cell type annotations, we combined the information from four independent approaches (STAR Methods; Figs. S1–S3; Datasets S1–S3) and assigned each cell to one of fourteen cell types (Fig. 1C) and to one of seven developmental stages (Fig. 2A) in a largely cluster-agnostic fashion.

We first mapped cells to 3D root geometry locations (Schmidt et al., 2014) using novoSpaRc (Nitzan et al, 2019), an algorithm that reconstructs the locations of single cells in space based on scRNA-seq data (Dataset S1; STAR Methods). Secondly, we used SEMITONES (Vlot et al., 2020), an algorithm that identifies enriched features in single cell data without prior clustering, to estimate the enrichment of marker gene expression in cell neighborhoods. Third, we calculated the correlation coefficient of each cell’s expression profile to published gene expression profiles of root cell types isolated with fluorescent reporters (Brady et al., 2007a; Li et al., 2016). Finally, we used an information-theoretic approach to compute Index of Cell Identity (ICI) scores for each cell (Birnbaum and Kussell, 2011; Efroni et al., 2015) (Dataset S3). The ICI score is quantitative and represents the relative contribution of cell identities as determined from a reference expression profile dataset. Combining these approaches allowed the expression profile of each cell to inform the boundaries between cell types and developmental stages.

The resulting atlas ordination consists of cells organized within continuous branches corresponding to four major root tissues (Dolan et al., 1993), each connected to a central group of cells (Fig. 1C). Lateral root cap (LRC) and columella cells comprise the root cap and form a single branch. Trichoblast (hair) and atrichoblast (non-hair) cells constitute the epidermis and form a second major branch. Cortex and endodermis cells, which together make up the ground tissue, form a third branch. Finally, the phloem, xylem, procambium, and pericycle cell types are present in the stele tissue and form a fourth branch. Based on marker genes (Dataset S1), we distinguished additional cell types within the phloem (metaphloem and companion cells; protophloem), xylem (protoxylem and metaxylem), and pericycle (xylem pole and phloem pole pericycle). However, we note that fewer validated markers were available for these subtypes. Surprisingly, the ground tissue and epidermis cell types show a clear ‘sub-branching’ topology at the tips of the main branches on the UMAP (Fig. 1C). These bifurcations may reflect a developmental phenomenon since they are unlikely to reflect technical artifacts such as differences in protoplasting-induced gene expression signatures (Fig. S4).

Overall, atlas cell type proportions are comparable to both microscopy data (Cartwright et al., 2009; Fig. 1D) and previously published root scRNA-seq datasets. Expression profiles of previously characterized genes (not used in the annotation process) also support the accuracy of the annotation (Fig. 1E). Differential expression analyses across all cell type groups (STAR Methods) identified cell type-specific genes that may be useful for the construction of fluorescent reporter lines (Dataset S1; Fig. S5).

We assigned developmental stage annotations to vascular, epidermal, and ground tissue cell types by comparing each cell transcriptome with gene expression profiles of manually dissected root tissue segments corresponding to meristematic, elongation, and maturation zones (Brady et al., 2007a). Based on these annotations, young cells of the proximal meristem are at the base of each major branch followed by distal meristematic, elongating, and finally mature cells at the tips (Fig. 2A). To assign developmental stages to cells in the root cap, we calculated the spatial distance for each cell to the nearest QC cell using the imputed geometry from novoSpaRc (STAR Methods).

To assess the overall accuracy of the developmental stage annotations, we examined expression patterns of previously characterized genes. First, we annotated the atlas with gene expression profiles associated with DNA endoploidy levels (Fig. 2B) (Bhosale et al., 2018; STAR Methods). In agreement with the annotation, the expression of genes associated with increasing ploidy is correlated with increasing maturation. Additionally, the expression of four G2/M phase cell cycle genes supports the meristematic zone annotation and indicates proximal versus distal root cap cells (Fig. 2C–F). The cyclins CYCB1;1 and CYCB1;2 are expressed in the proximal meristem while CDKB1;1 and CDKB2;1 are expressed in both proximal and distal meristematic cells (Ishida et al., 2009). Lastly, developmental stage expression profiles of known genes agree with published *in vivo* characterizations (Fig. 2G).

Overall, the atlas annotations suggest that the combined transcriptome data accurately describe relationships between and within individual cell types. Similar to previous *Arabidopsis* root atlas UMAP and tSNE plots, older cells from each tissue type radiate from a central group of young cells. However, the integration of greater cell numbers captures more cell states along developmental time and therefore suggests a continuous progression of differentiation for all major root cell types.

### Developmental progression can be inferred across individual tissue types

**Figure 3.**
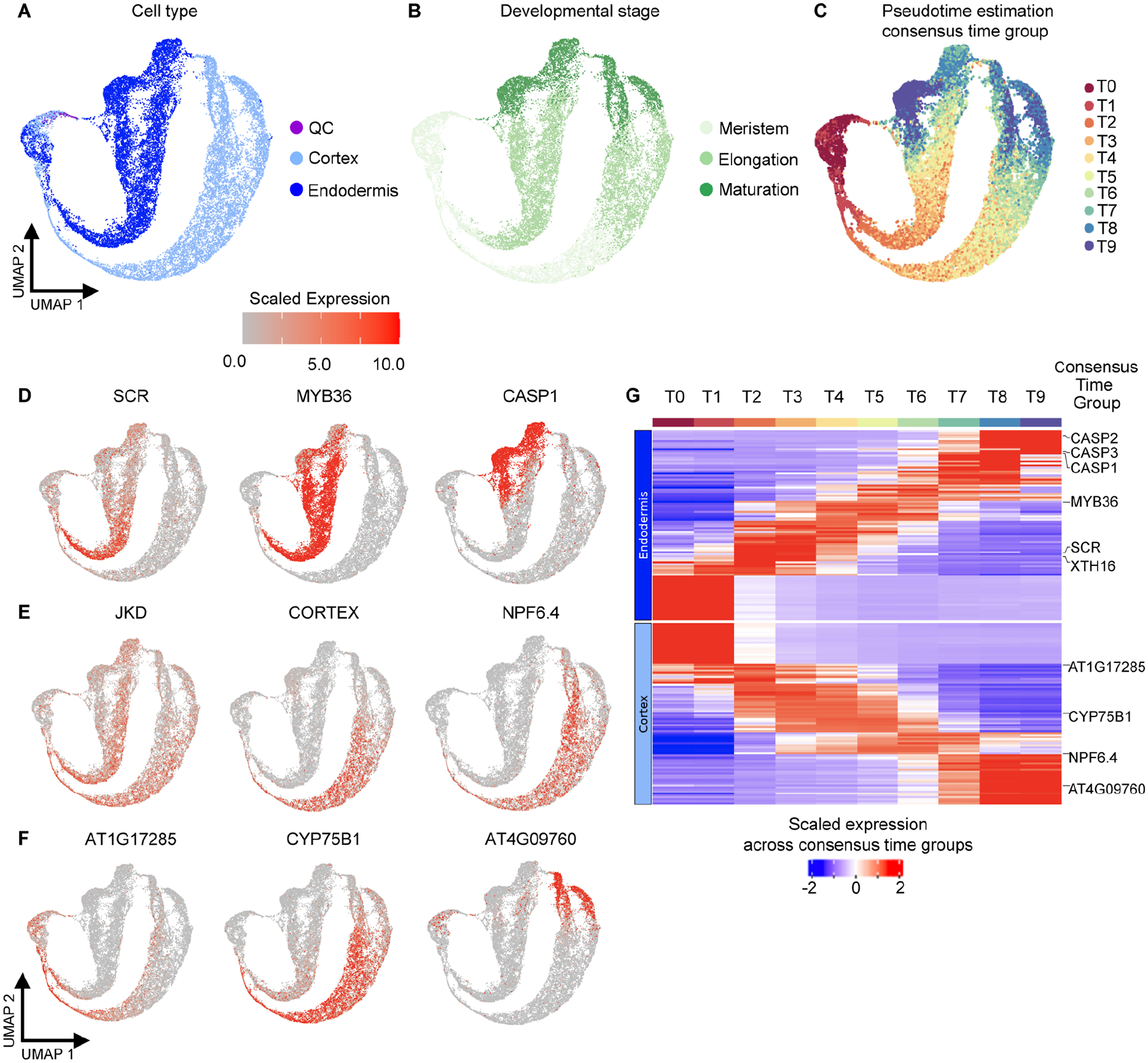
Pseudotime estimates reflect the dynamics of ground tissue differentiation. **A)** Endodermis and cortex-annotated cells (ground tissue) were extracted from the atlas and re-embedded in a 2D UMAP. QC cells were included to help anchor pseudotime estimations. **B)** Ground tissue cells annotated with developmental stage labels. **C)** Ground tissue cells annotated with consensus pseudotime group labels. T0 denotes the youngest cells. **D-E)** Scaled expression patterns (STAR Methods) of known endodermis and cortex markers. **F)** Newly identified cortex-expressed genes are candidates for marker development. **G)** Scaled expression of 90 and 94 non-redundant, differentially expressed genes across consensus pseudotime groups for cortex and endodermis, respectively. Warmer colors denote higher expression. Although thousands of differentially expressed genes were identified across pseudotime, only the most strongly differentially expressed genes for each of the ten pseudotime bins were plotted for simplicity. See also Dataset S4.

**Figure 4.**
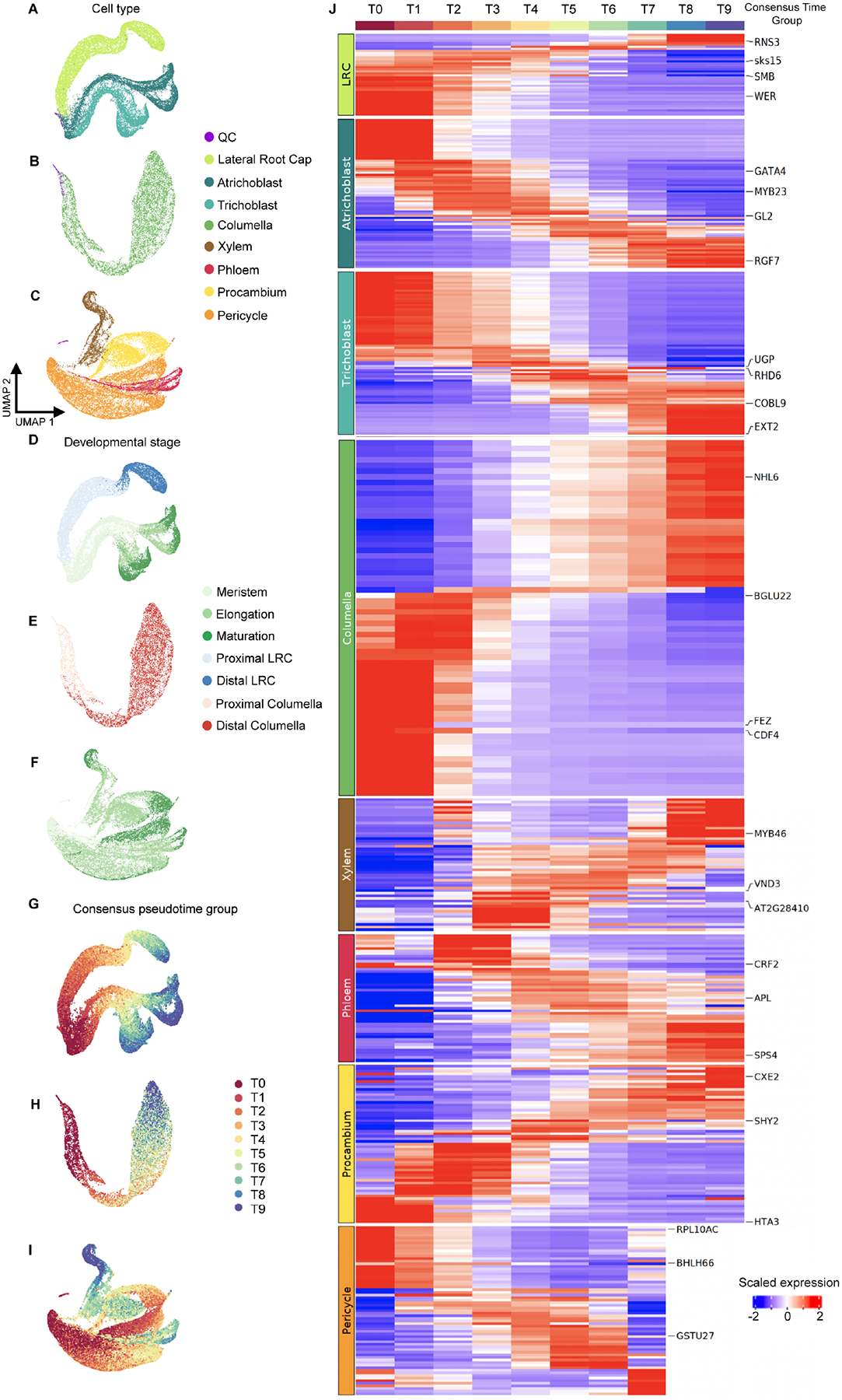
Pseudotime progressions indicate gradual gene expression changes underlie development across tissues and developmental zones. **A-C)** Cells annotated as trichoblast, atrichoblast, and lateral root cap (A), columella (B), and stele (C) were extracted from the atlas and re-embedded in individual UMAPs. **D-F)** UMAPs annotated by developmental stage. **G-I)** UMAPs annotated by consensus time groups. **J)** Scaled expression of the top ten non-redundant, most highly differentially expressed genes across consensus pseudotime groups for each cell type. See also Dataset S4.

To analyze developmental progression in more detail, we started from a simple pseudotime analysis within annotated cell lineages. We subdivided the atlas into four tissue/lineage groups based on the stem cell of origin (Dolan et al., 1993) and quantified cell state progression using two methodologically distinct, non-graph-based tools: CytoTRACE (Gulati et al., 2020) and scVelo (Bergen et al., 2020). CytoTRACE uses gene diversity to estimate pseudotime while scVelo is based on the concept of RNA velocity. The results from both methods were strongly correlated (Dataset S4; STAR Methods), suggesting that they reflect true biological signal. We therefore averaged the pseudotime estimations into a ‘consensus pseudotime’ annotation for each tissue (Figs. 3 and 4). Overall, the pseudotime estimations reflect biological knowledge. For example, the consensus time annotation for the ground tissue corresponds with the developmental stage annotation and with expression of known endodermis and cortex markers (Fig. 3A–F). As expected, given the 0.5 cm length of harvested root tissue, scaled expression (STAR Methods) of *SCARECROW* (*SCR*), *MYB36*, and *CASPARIAN STRIP MEMBRANE DOMAIN PROTEIN 1* (*CASP1*) represent markers for endodermis cells spanning the meristematic zone to early maturation zone. Expression of *JACKDAW* (*JKD*), a ground tissue marker, as well as cortex-specific markers CORTEX (AT1G09750) and NPF6.4 (AT3G21670) also match the expected profiles. Examples of newly identified genes with expression profiles specific to a subset of the developmental progression are shown for cortex (Fig. 3F). Differential expression analyses generated by partitioning the pseudotime ordering into ten groups (T0 to T9) identified a gradual progression of genes dynamically expressed during cortex and endodermis differentiation (Dataset S4), including previously characterized developmental regulators (Fig. 3G).

Similarly, differential expression analyses across ten pseudotime bins show gradual, overlapping waves of gene expression for stele, epidermis + LRC, and columella cells (Fig. 4). In agreement with previous work on the root meristem (Wendrich et al., 2017), these results suggest that gradual changes in gene expression also underlie differentiation in the elongation and maturation zones. Gradual, overlapping gene expression dynamics across development are also supported by a dearth of cell type-specific markers specific to a particular developmental zone (Fig. S6). Interestingly, there are two distinct groups of genes along the columella pseudotime progression (Fig. 4J), consistent with a rapid change in transcription that could reflect the differentiation of cells immediately after stem cell division (Hong et al., 2015). Also of interest was the lack of pericycle cells in the two most mature pseudotime bins (Fig. 4J). This agrees with previous observations that the pericycle matures more slowly than other cell types and retains meristematic characteristics (Beeckman and De Smet, 2014).

### Optimal transport analysis identifies developmental trajectories

Because pseudotime inference indicates that root cell types mature at different rates, we used an optimal transport (OT) based method to infer developmental trajectories across the entire atlas. This method was initially developed as a way to move large quantities of earth with minimal work (Monge, 1781). More recently, OT was used to infer developmental trajectories from animal and human scRNA-seq data (Schiebinger et al., 2019; Marjanovic et al., 2020; Massri et al., 2021) but has yet to be applied to plant data. OT connects cells from one static snapshot to their putative ancestors at earlier developmental stages and to their descendants at later developmental stages (S. Zhang et al., 2021). This allows developmental trajectories of individual cells or entire lineages to be followed through pseudotime. Compared to separating cells by lineage annotation and analyzing pseudotemporal trends within each lineage, OT allows us to probe further back in earlier pseudotime, where lineage-annotations are less reliable. Consequently, this allows us to analyze fate specification events.

**Figure 5.**
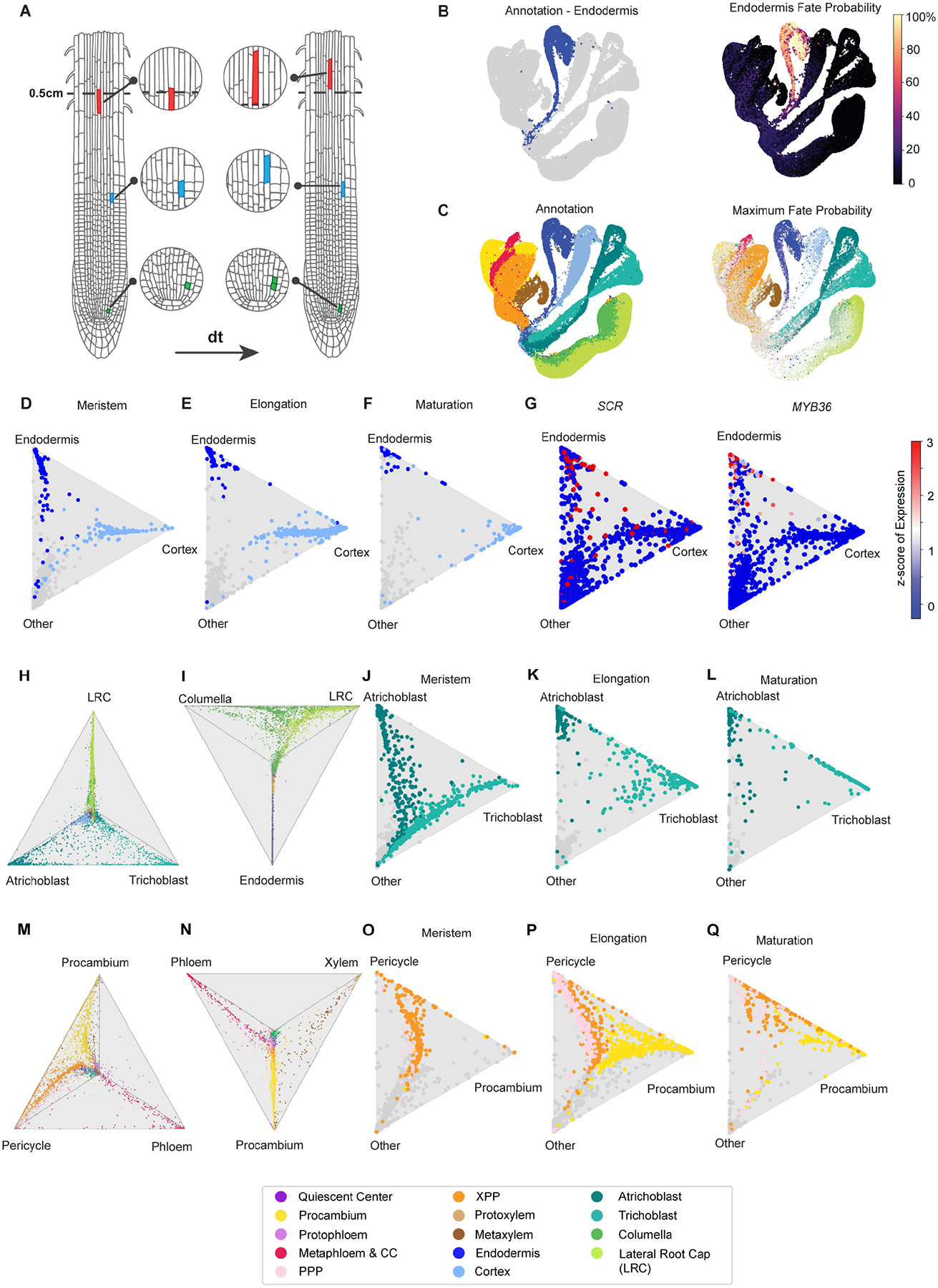
Optimal transport identifies developmental trajectories. **A)** The root tip, denoted here as the 0.5 cm harvested for scRNA-seq, remains in equilibrium over a time period of duration dt. Individual cells progress through developmental stages, including dividing (green; transit amplifying divisions following stem cell divisions), enlarging (blue; elongation zone), and exiting the region of interest (red; early maturation zone). **B)** Endodermis fate probability (right) agrees with endodermis annotations (left), visualized on the UMAP. **C)** All fate probabilities are visualized on the UMAP (right). Cells are colored according to the lineage of maximum fate probability and cells fade to grey as the fate specification becomes less determined (i.e., as the maximum fate probability decreases). **D-Q)** StationaryOT fate probabilities reflect known developmental relationships and, in some cases, fate fluidity between cell types. For each plot, the dataset was down-sampled to 10,000 cells to facilitate visualization. **D-F)** Triangle plots with cells plotted according to cortex, endodermis, and all other fate probabilities. Cells annotated as cortex and endodermis are colored light and dark blue, respectively, with all other cells in gray. The three plots show cells from each of the three developmental stages. **G)** Increasing endodermis fate probabilities agree with developmental stage annotations and with expression patterns of *SCARECROW* (*SCR*) and *MYB36*. The legend shows z-scores of gene expression, where a score of 1 is one standard deviation above mean expression. **H-I**) Cells are arranged on tetrahedron plots according to cell fate probabilities from epidermis and root cap tissues. The top vertex of each face of the tetrahedron plots (looking down) contains all other cell type fates besides the three labeled at each of the remaining vertices. **J-L)** Cells are plotted according to atrichoblast, trichoblast, and all other fate probabilities. Cells annotated as atrichoblast and trichoblast are colored accordingly with all other cells in gray. **M-N)** Tetrahedron plots representing stele cell fate probabilities. Xylem, phloem, and pericycle terminal fates from pseudotime estimates were used for StationaryOT but stele cells are colored here according to annotated sub-types. **O-Q)** Cells are plotted according to pericycle, procambium, and all other fate probabilities. See also Figure S7.

The 0.5 cm portion of the root harvested for the atlas can be thought of as a system in equilibrium: cell divisions in the meristem create new cells, which are balanced against the flux of cells exiting this region. We applied stationary OT analysis (StationaryOT, Fig. 5A) (S. Zhang et al., 2021), which leverages estimates of cellular growth rates to infer trajectories for systems in equilibrium. We used the consensus pseudotime to define groups of cells that represent terminal destinations (i.e., the ‘fates’; STAR Methods) and estimated growth rates for individual cell types based on time-lapse imaging data of dividing cells (Rahni and Birnbaum, 2019). Using these parameters, StationaryOT calculates a vector of fate probabilities for each cell in the atlas, i.e., the likelihood that a given cell will eventually give rise to a mature cell of a particular cell type. Individual fate probabilities can be visualized on the atlas UMAP coordinates and agree with our cell type annotations, as shown for endodermis (Fig. 5B). The maximum fate probability for each cell, which indicates the most likely ultimate cell lineage, agrees with our lineage annotations (Figs. 5C and S7), and cells appear to gradually become more biased towards specific fates at later pseudotimes (Fig. 5C). Taken together, these results suggest that the developmental trajectories inferred by StationaryOT, which are largely independent of the atlas annotations and do not require segmentation of the atlas into constituent lineages, reflect existing biological knowledge for differentiation of each cell and tissue type.

Differentiation events can be visualized by projecting multiple fate probabilities in barycentric coordinates as ‘triangle plots’ (STAR Methods). Contrary to pseudotime inference methods, which are applied to individual tissues or cell lineages, these visualizations can be used to interrogate how fate acquisition of each cell type relates to all other cell types. To explore the divergence of endodermis and cortex identities, we designated a vertex of the triangle for each of these fates with the third vertex representing all other possible fates. Cells were then plotted according to their relative probabilities. The position of meristematic cells in the triangle interior indicates lower cortex or endodermis fate probabilities at earlier developmental stages (Fig. 5D). Mature cells are grouped at the cortex and endodermis vertices, which indicate 100% cortex or endodermal fate probabilities, respectively (Fig. 5F; STAR Methods). Plotting the expression of known endodermis markers indicates that endodermis fate probabilities increase with maturation as expected (Fig. 5G). Interestingly, in the elongation zone, endodermis cells are already strongly fated while cortex fate appears indeterminate (Fig. 5E). This could reflect the putative ‘ground state’ of the cortex for which the ground tissue was named and suggests that elongating cortex cells have the potential to acquire different fates (Esau, 1953; Cui, 2015).

In another example, the fate probabilities for trichoblast and atrichoblast, which together form the epidermis, are more similar to each other than either is to lateral root cap cells, although all derive from the same stem cell (Fig. 5H). Similarly, columella root cap cell fates are distinct from all other fates except lateral root cap (Fig. 5I). Plotting cells by developmental zone annotations (Fig. 5J–L) indicates that atrichoblast and trichoblast cell fates are indeterminate in the meristem with some fluidity in the elongation zone, which agrees with previous observations that epidermal cell fate is not fixed in young cells (Berger et al., 1998a; Ryu et al., 2019).

For stele cell types, plotting cells according to fate probabilities reflects the distinct identities of xylem and phloem, both compared to each other and to procambium and pericycle cells (Fig. 5M–N). This is visualized on tetrahedron plots by, for example, the concentration of xylem cells on the side of the triangle between the ‘other’ and xylem vertices, indicating that the cells have higher fate probabilities for xylem than for phloem or any other cell type (Fig. 5N). By contrast, procambium and pericycle fates appear to be fluid (Fig. 5M, O–Q), similar to the fluidity between atrichoblast and trichoblast fates.

### Optimal transport analysis facilitates identification of developmental regulators

To identify TFs with expression patterns predictive of fate specification probabilities for each cell type, we applied L1-regularized linear regression (i.e., the Lasso) (Dataset S5; Fig. S7). Among top ranked genes were numerous known regulators with positive coefficients, indicating a positive influence on a given cell lineage (STAR Methods; Dataset S5). Examples include: i) *MYB36* and *SCR* for meristematic and elongation endodermis; ii) *JKD* for meristematic and elongation cortex; iii) *GLABRA2*, *MYB23*, and *CAPRICE* for meristematic and elongation atrichoblast; and iv) *RHD6* (*BHLH83)* for meristematic trichoblast. The re-discovery of known regulators for all major root cell types as top candidates supports the utility of the atlas itself as well as the StationaryOT approach to identify TFs with key roles in cell fate specification. Many of the genes identified by the regressions are unstudied and represent a rich resource for functional characterization.

### scRNA-seq reveals differentiation pathways of cell identity mutants

In addition to identifying new candidate regulators, scRNA-seq allows us to ask how known regulators control tissue and organ development. In the root, the TFs *SHORTROOT* (*SHR*) and *SCR* function in a transcriptional regulatory complex and are essential for stem cell niche maintenance and tissue patterning (Benfey et al., 1993; Di Laurenzio et al., 1996). Using annotation label transfer from the atlas to inform new datasets (Stuart et al., 2019), we asked how the loss of *SHR* or *SCR* function affects tissue composition as well as cell identity and differentiation.

**Figure 6.**
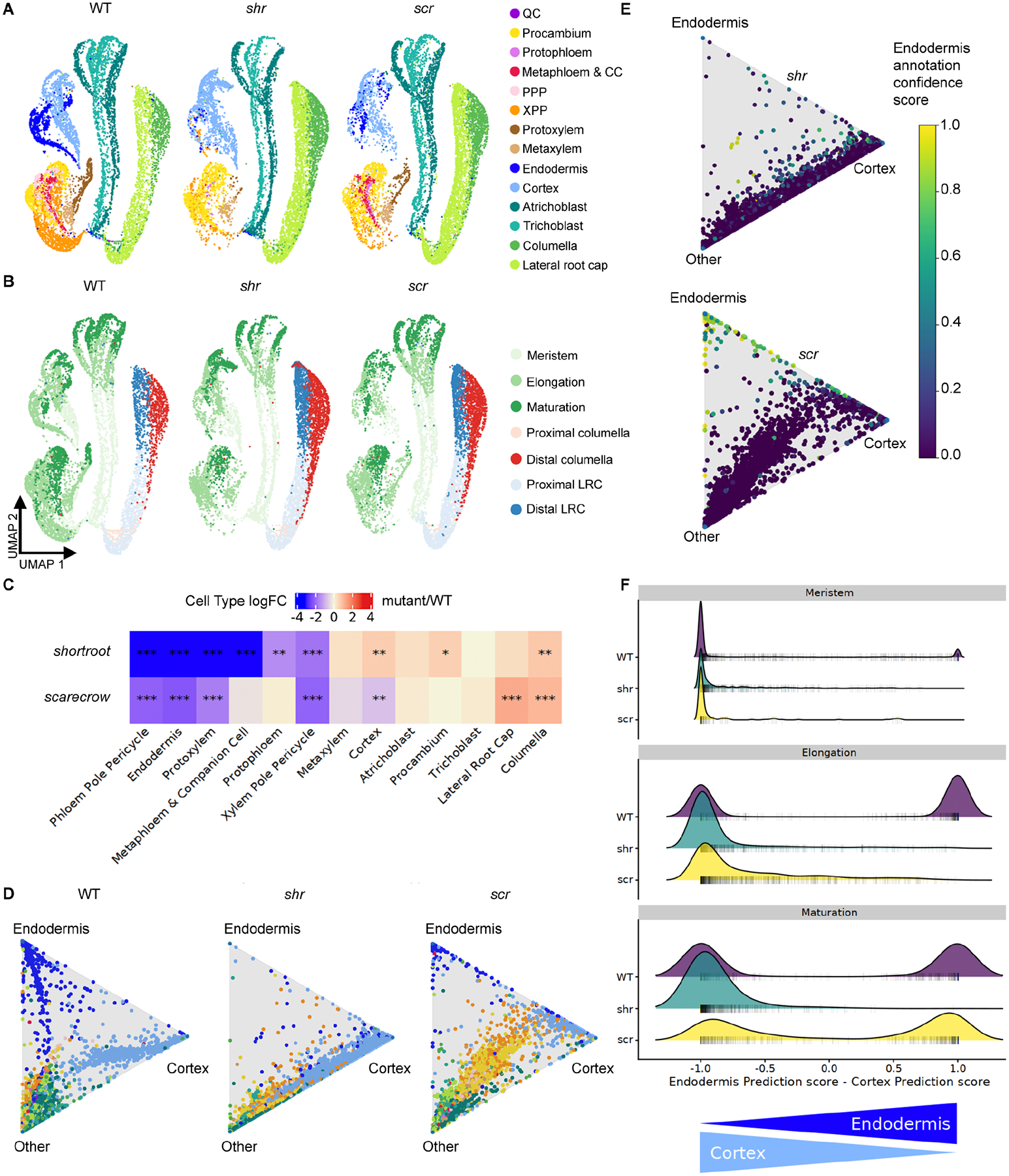
Atlas informs cell type abundance and identity changes in *shr* and *scr* mutants. **A)** UMAPs with cell type annotations representing WT integrated with *shr* and *scr*. Data from each genotype was down-sampled to 10,000 cells to facilitate comparison. **B)** UMAPs from A but labeled with developmental stage annotation. **C)** Differential abundance analysis using the full integrated WT, *shr*, and *scr* dataset reports significant changes in per-label cell type abundance between mutants and WT. *** False Discovery Rate (FDR) < 0.001; ** FDR < 0.01; * FDR < 0.05. **D)** Triangle plots illustrating cell fate probabilities calculated by StationaryOT. Cell type color legend is the same as A. **E)** Triangle plots show cells arranged according to endodermis, cortex, and all other fate probabilities for *shr* (top) and *scr* (bottom) as calculated by StationaryOT. Each dot represents one cell. Dots are colored by endodermis annotation confidence scores after label transfer from the WT atlas by Seurat. Zero and one are the lowest and highest confidence scores, respectively. **F)** Data density plot of the cortex classification score subtracted from the endodermis classification score for each cell, plotted by developmental stage. On the x-axis, a value of 1 indicates confident endodermal classification while a value of −1 indicates confident cortex classification. The annotation of each *scr* and *shr* cell was assigned using a weighted vote classifier based on reference cell labels from the atlas (Stuart et al., 2019). Cell type classification scores range from zero (lowest confidence) to one (highest confidence). Absolute cell numbers are represented by the shaded bars. See also Datasets S1 – S2, Data S1.

Both *shr* and *scr* mutants lack the asymmetric cell division that patterns the ground tissue, resulting in a single mutant tissue layer instead of the cortex and endodermis cell layers. Previous detection of tissue-specific markers and morphologies revealed that the mutant layer has cortex-like attributes in *shr* (Benfey et al., 1993) but a mixture of cortex and endodermis characteristics in *scr* (Di Laurenzio et al., 1996). These phenotypes were reflected in the scRNA-seq data given the significant reduction of cells expressing endodermal markers in both *shr* and *scr* (Fig. 6A–C). A second striking observation was the decrease in protoxylem cell abundance in both mutants and the decrease of protophloem and metaphloem abundance in *shr* (Fig. 6C), consistent with reports of defects in *shr* and *scr* stele development (Levesque et al., 2006; Carlsbecker et al., 2010; Yu et al., 2010; Cui et al., 2011; Kim et al., 2020). In both mutants, we also identified a significant reduction in the abundance of xylem pole and phloem pole pericycle cells (Fig. 6C). This is surprising given that there are cells located in the radial pericycle position in both mutants (Kim et al., 2020; Di Laurenzio et al., 1996). However, in *Arabidopsis*, lateral roots are formed from xylem pole pericycle cells (Beeckman and De Smet, 2014) and lateral root development is altered in the *shr* mutant (Lucas et al., 2011). This observation previously led to the hypothesis that *shr* cells may differentiate into a state that cannot support normal lateral root formation (Lucas et al., 2011). Taken together, these results indicate a putative loss of pericycle identity in *shr*.

### scRNA-seq suggests trans-differentiation of the *scr* mutant layer

We next asked how individual cells contribute to the reported mixed identity of the *scr* mutant layer (Di Laurenzio et al., 1996). One hypothesis is that cells acquire an endodermis or cortex identity early in development and the mutant layer is a heterogeneous mixture of the two cell types along the entire cell file. Alternatively, each cell may have a mixture of cortex and endodermis attributes. A third hypothesis is that cells acquire one identity early in development and subsequently change their fate. To distinguish among these possibilities, we used StationaryOT to calculate *scr* cell fate probabilities. *scr* cortex and endodermis cells exist on a continuum between cortex and endodermis fates, as indicated by the cells aligned on the side of the triangle plot between the cortex and endodermis vertices (Fig. 6D). This reflects the probabilities of both endodermis and cortex fates for these cells. In the *shr* dataset, although some cells are annotated as endodermis, the lack of cells near the endodermis vertex coupled with low confidence scores following label transfer via Seurat (Fig. 6E) suggests that few if any *shr* cells are endodermis-like. Similar to *shr* but unlike WT, *scr* endodermis cells do not show a progression from the central part of the triangle toward the endodermis vertex. This suggests that *scr* cells may not gradually acquire endodermis identity from an undifferentiated state.

**Figure 7.**
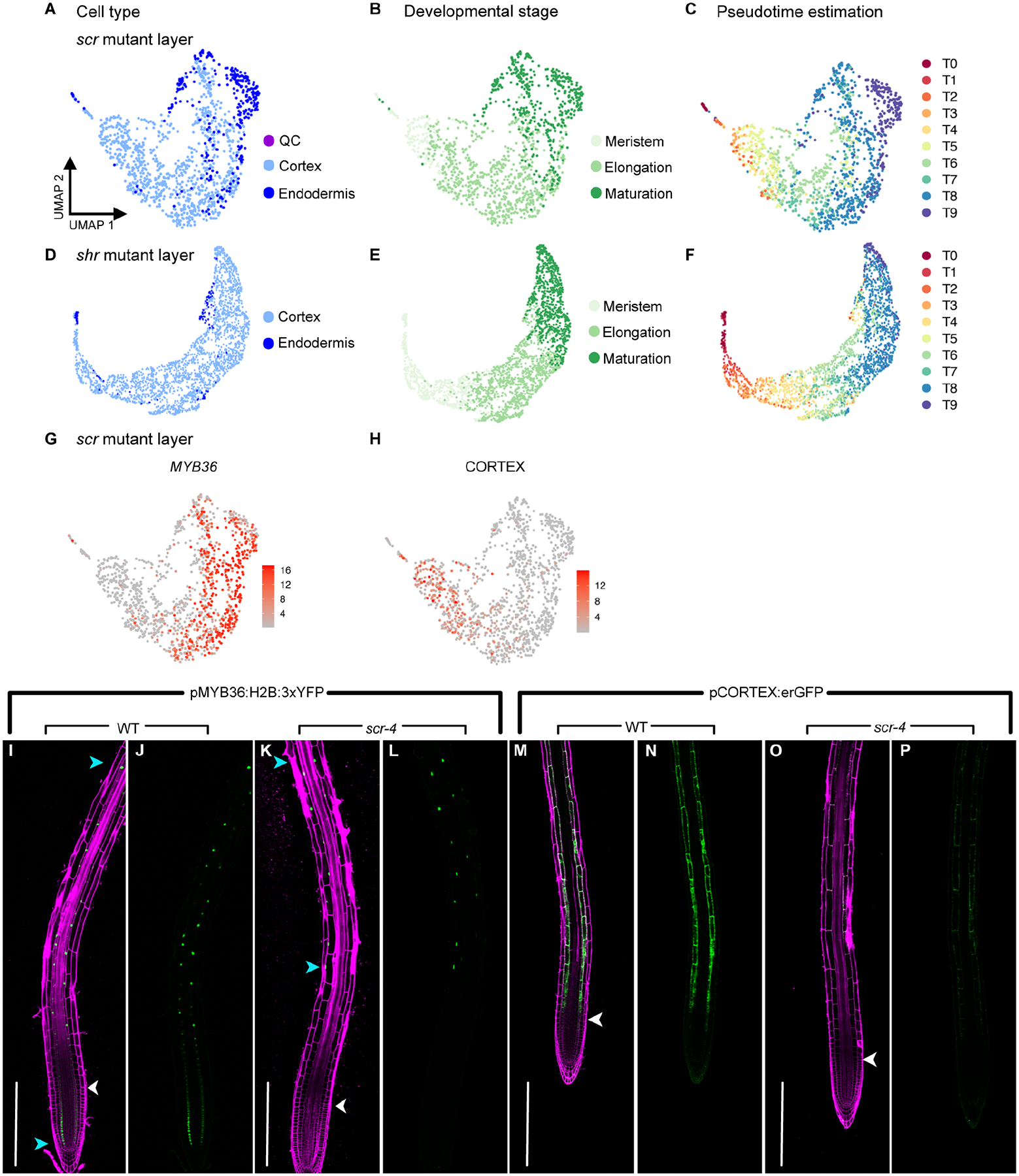
Spatial expression patterns of MYB36 and CORTEX transcriptional reporters are consistent with cortex to endodermis fate transition in the *scarecrow* mutant layer. **A)** Cortex, endodermis, and QC cells extracted from the *scr* dataset and re-embedded in a UMAP. **B)** Developmental stage annotation labels were transferred from the WT atlas to the *scr* mutant layer cells. **C)** Consensus pseudotime group annotation labels were transferred from WT ground tissue to *scr*. Warmer to cooler colors represent the developmental progression from youngest to oldest cells, respectively. **D)** Cortex and endodermis cells were extracted from the *shr* dataset and re-embedded in a UMAP. **E-F**) As for *scr*, developmental stage (E) and consensus time group annotation labels (F) were transferred from the WT atlas to *shr* mutant layer cells. **G-H**) Scaled expression of *MYB36* and AT1G09750 (CORTEX reporter) in cells of the *scr* mutant layer. **I-L)** pMYB36:H2B:3xYFP reporter in WT (I,J) and *scr-4* (K,L) showing loss of meristem and elongation zone expression in *scr* mutant. Blue arrowheads mark the longitudinal location of the first and last cells in the image with visible YFP. **M-P)** pCORTEX:erGFP reporter in *scr-4*/pCORTEX:erGFP F2 progeny with WT ground tissue phenotype (M,N) and *scr-4* mutant layer phenotype (O,P) showing reduced expression of cortex marker as cells mature in the mutant. Red and green channel overlay images (I, K, M, O) are propidium iodide-stained roots (magenta) and YFP or GFP signal. Green channel images (J, L, N, P) are YFP or GFP alone. Scale bars are 200 μm. White arrowheads mark the beginning of the elongation zone. See also Dataset S1.

To further explore the developmental progression of the *scr* mutant layer, we extracted cortex and endodermis-annotated cells from the *scr* dataset. We asked if the proportion of cells with each cell-type annotation changes according to developmental zone. We observed that most meristematic and elongating *scr* cells are confidently classified as cortex, though a subset of cells with low cortex prediction scores is evident in the elongation zone (Fig. 6F). Differentiating *scr* cells, however, are confidently annotated as either cortex or endodermis, though some cells seem to have attributes of both. By contrast, nearly all *shr* mutant layer cells are confidently annotated as cortex (Fig. 6F). In agreement with these results, consensus pseudotime annotation labels transferred from the atlas suggest that the youngest cells of the *scr* mutant layer are primarily cortex-like while endodermis identity is most evident in older cells (Fig. 7A–C). By contrast, cortex identity is predominant in all developmental states for *shr* mutant layer cells (Fig. 7D–F). Together, these results support the hypothesis that *scr* mutant layer cells are cortex-like in the early stages of development but acquire attributes of endodermal identity as they age.

To test our hypothesis *in vivo*, we asked if spatial expression patterns of known cortex and endodermis markers are altered in the *scr* mutant layer. In WT roots, transcriptional reporters for MYB36, an endodermis marker, and AT1G09750 (CORTEX), a cortex marker, are expressed in the elongation zones of their respective cell types (Fig. 3D–E; Liberman et al., 2015; Lee et al., 2006). The MYB36 reporter is also expressed in the meristematic zone of the endodermis. However, scRNA-seq data suggests that these expression patterns are altered in the *scr* mutant layer: CORTEX expression is reduced to the early elongation zone while MYB36 is expressed in older cells of the elongation and maturation zones but not in the meristem (Fig. 7G–H).

Consistent with the scRNA-seq observations, expression from a MYB36 transcriptional reporter was visible only in the late elongation and maturation zones of the *scr* mutant layer while signal from a CORTEX transcriptional reporter was diminished in the elongation and maturation zones (Fig. 7I–P). Additionally, a transcriptional reporter for the meristematic cortex, CO2, was previously shown to be robustly expressed in young mutant layer cells closest to the QC in *scr-4* (Heidstra et al., 2004). Taken together, the *in vivo* expression patterns of MYB36, CORTEX, and CO2 reporters validate developmental observations made from scRNA-seq data and suggest that young *scr* mutant layer cells are cortex-like while the identity of older cells changes to more endodermis-like. Although endodermal identity has been considered independent of *SCR* and the existence of *SCR*-independent regulation of MYB36 has previously been proposed (Drapek et al., 2018), our results indicate that *SCR* is required in meristematic and early elongation cells for *MYB36* expression and endodermal identity.

## Discussion

Observations made from WT and mutant data lay the foundation to address fundamental questions regarding common versus shared developmental regulatory programs between cell types, cell identity transitions, and the roles of neighboring cells in determining cell identity. Building organ-scale gene expression maps is also essential to drive technological innovation such as reprogramming cell identity and inducing phenotypic changes via cell type-specific gene editing. To address these goals, we built a comprehensive root scRNA-seq atlas, developed an iterative pipeline to annotate each cell individually, and developed COPILOT, a species-agnostic quality control software for scRNA-seq data. An interactive web interface is available for the atlas at https://phytozome-next.jgi.doe.gov/tools/scrna/.

The resolution of developmental progression represented in the atlas provides an opportunity to ask how cell fate specification and stabilization differ between cell types, especially those that arise from divisions of the same stem cell. For example, genes uniquely expressed in the cortex or endodermis early in development for each cell type may include new regulators of cell type specification in the ground tissue. StationaryOT, unlike pseudotime estimation methods, allows insight into transcriptional similarities across cell type fate specification. An intriguing question is how and why cell types that arise from the same stem cell, such as procambium and phloem, are more transcriptionally distinct than cell types which arise from different stem cells, e.g., procambium and pericycle.

Transcriptional regulators of tissue patterning, cell identity specification, and differentiation have previously been identified for each root tissue. However, we have by no means discovered all regulators and we have limited understanding of what connects known gene regulatory networks (GRNs) operating at different developmental stages in individual cell types (Drapek et al., 2017). The regression we applied to StationaryOT and gene expression data identified a number of uncharacterized genes as candidate regulators of cell fate. Although we highlighted candidates predicted to push cells toward a given lineage, the analysis also identified genes for each cell type that do not favor the lineage. These genes will be interesting to perturb and test for phenotypes with approaches such as cell type-specific overexpression. Given the applicability of StationaryOT to the full atlas, the candidates may also include TFs that coordinate developmental processes across cell and tissue types.

For future studies, the atlas represents a rich resource to infer GRNs underlying the differentiation of each cell type with tools such as CellOracle (Kamimoto, Hoffmann, and Morris, 2020). The atlas data can also be compared to or combined with data from other modalities to examine gene regulatory relationships and narrow down the candidate TFs that regulate cell fate decisions. For example, GRNs inferred from the atlas data could be compared to DAP-seq data (O’Malley et al., 2016) to determine if TFs of interest bind to regulatory regions of predicted downstream genes. Another promising avenue to identify transcriptional regulators controlling cell fate and differentiation is the combination of chromatin accessibility (scATAC-seq) and scRNA-seq data (Stuart and Satija, 2019; Rautenstrauch et al., 2021), the feasibility of which has been demonstrated for *Arabidopsis* and rice roots (Dorrity et al., 2021; Farmer et al., 2021; T-Q Zhang et al., 2021).

Beyond WT root development, the atlas enables interrogation of cell identity and tissue composition changes in a mutant context. The putative trans-differentiation from cortex to endodermis identity in the *scr* mutant layer represents a new system with which to investigate transcriptional changes underlying cell identity transitions. In regeneration studies, plant cells show a widespread ability to acquire new fates (Efroni et al., 2018), which raises questions such as how do cells ‘forget’ their old fate and are there unstable transitional states required for identity transitions. To date, there are few transcriptome-level datasets describing cell identity changes in plants, although such transitions represent important developmental processes including pericycle cells undergoing identity changes during lateral root formation (Von Wangenheim et al., 2016; Gala et al., 2021). The *scr* scRNA-seq data will allow us to probe questions about these transitional states, such as, “Do cells express heterogeneous mixtures of cortex and endodermal identity” and, “Do cortex cells ‘de-differentiate’ prior to expression of endodermal markers?”

To facilitate the utility of the atlas as a community resource, we produced comprehensive tutorials and toy datasets to demonstrate how the atlas annotation labels can be transferred to new datasets. In addition to analyzing mutants, the atlas can guide interpretation of scRNA-seq data from plants responding to environmental stress, as well as data from crop species for which comprehensive root cell-type markers are unavailable.

### Limitations of the Study

We relied only on transcriptional profiles to determine a cell’s identity and developmental state, which excludes other information such as proteomic profiles. We note that the atlas developmental stage annotation is based on correlation with microarray data from tissue segments hand-dissected according to morphological markers. The boundaries between developmental zones in the atlas may not correlate precisely with root morphology due to variability between roots and between individuals in interpreting the markers.

## STAR Methods

### RESOURCE AVAILABILITY

#### Lead Contact

- Further information and requests for resources and reagents should be directed to and will be fulfilled by the lead contact, Philip N. Benfey (philip.benfey@duke.edu).

#### Materials Availability

Seeds for the *scr-4/pCORTEX:erGFP* and *scr-4/pMYB36:H2B:3xYFP* lines are available from Philip N. Benfey upon request.

#### Data and Code Availability

- Single-cell RNA-seq data have been deposited at GEO with the accession number GSE152766 and are publicly available as of the date of publication. Accession numbers are listed in the key resources table. Microscopy data reported in this paper will be shared by the lead contact upon request.
- All original code has been deposited at Zenodo and is publicly available as of the date of publication. DOIs are listed in the key resources table.
- Any additional information required to reanalyze the data reported in this paper is available from the lead contact upon request.

### EXPERIMENTAL MODEL AND SUBJECT DETAILS

Seeds from wild type *Arabidopsis thaliana* ecotype Columbia (Col-0), *shortroot-2* (Col-0; ABRC stock number CS2972), and *scarecrow-4* (Landsberg background; ABRC stock number CS6505; we backcrossed to Col-0 > 5 times) were surface sterilized with a 50% (v/v) bleach, 0.05% (v/v) Tween-20 solution for 10 minutes and subsequently stratified for 48 hours at 4°C. Seeds were sown at a density of ~150–300 seeds/row on 1X Linsmaier and Skoog (LSP03-1LT, Caisson Labs; pH 5.7), 1% sucrose media covered by 100 μm nylon mesh. Plates were placed vertically in a Percival chamber programmed to 16h light, 8h dark conditions at 22°C.

### METHOD DETAILS

#### Protoplast Isolation and scRNA-seq

Five days after sowing, 1,000–3,500 primary roots/sample were cut ~0.5 cm from the root tip and placed in a 35 mm-diameter dish containing a 70 μm cell strainer and 4.5 mL enzyme solution (1.25% [w/v] cellulase [ONOZUKA R-10, GoldBio], 0.1% Pectolyase [Sigma], 0.4 M mannitol, 20 mM MES (pH 5.7), 20 mM KCl, 10 mM CaCl_2_, 0.1% bovine serum albumin, and 0.000194% (v/v) ß-mercaptoethanol). Roots were harvested 3–4 hours after the lights were illuminated in the growth chamber set to long day conditions. After digestion at 25°C for 1 hour at 85 rpm on an orbital shaker with occasional stirring, the cell solution was filtered twice through 40 μm cell strainers and centrifuged for 5 minutes at 500 × g in a swinging bucket centrifuge. Subsequently, the pellet was resuspended with 1 mL washing solution (0.4 M mannitol, 20 mM MES (pH 5.7), 20 mM KCl, 10 mM CaCl_2_, 0.1% bovine serum albumin, and 0.000194% (v/v) ß-mercaptoethanol) and centrifuged for 3 minutes at 500 × g. The pellet was resuspended with washing solution to a final concentration of ~1000 cells/μL. The protoplast suspension was then loaded onto microfluidic chips (10X Genomics) with v3 chemistry to capture either 5,000 or 10,000 cells/sample. Cells were barcoded with a Chromium Controller (10X Genomics). mRNA was reverse transcribed and Illumina libraries were constructed for sequencing with reagents from a 3’ Gene Expression v3 kit (10X Genomics) according to the manufacturer’s instructions. cDNA and final library quality were assessed with a Bioanalyzer High Sensitivity DNA Chip (Agilent). Sequencing was performed with a NovaSeq 6000 instrument (Illumina) to produce 100bp paired end reads.

#### Transgenic Lines

Plants homozygous for the *scr-4* allele (Fukaki et al., 1998) were crossed with previously published pCORTEX:erGFP (Lee et al., 2006) and pMYB36:H2B:3xYFP (Drapek et al., 2018) transcriptional reporters. F2 generation seedlings were imaged at 5 days old. Individuals homozygous for the *scr-4* allele were identified by the presence of a mutant layer. pCORTEX:erGFP/*scr-4* seedlings were grown on 1X MS plates with 10 μg/mL BASTA to confirm presence of the reporter construct prior to imaging.

#### Microscopy and Image Processing

Roots were stained with 10 mg/ml propidium iodide (PI) for 1 minute and imaged with a Zeiss 880 confocal using a x40 objective. The following are excitation (ex) and emission (em) parameters. PI: ex: 561 nm; em: 600–650 nm; YFP: ex: 488 nm, em: 530–560 nm; GFP: ex: 488 nm; em: 500–550 nm. Median longitudinal sections were chosen for each image and representative images are shown. All image analyses were performed in ImageJ. The minimum signal for each channel was adjusted by measuring the intensity histogram of the background and removing the mean plus two standard deviations from the signal. Brightness was adjusted for each channel to maximize the range of display. When GFP or YFP signals from two images are directly compared, the maximum brightness was adjusted identically for each image.

### QUANTIFICATION AND STATISTICAL ANALYSIS

#### scRNA-seq Data Pre-processing

FASTQ files were generated from Illumina BCL files with Cell Ranger (v3.1.0) mkfastq (10X Genomics). Subsequently, gene-by-cell raw count matrices of spliced and un-spliced transcripts were generated using kallisto (Bray et al., 2016) (v0.46.2) and bustools (Melsted et al., 2019) (v0.40.0) as well as R packages BUSpaRse (Moses and Pachter, 2020) (v1.1.3) and BSgenome (v1.54.0; Pagès, 2020). The pipeline is summarized on our scKB GitHub repository (https://github.com/ohlerlab/scKB). Reads were aligned to the Arabidopsis genome BSgenome object (“BSgenome.Athaliana.TAIR.TAIR9”) with TAIR10 gene annotation file. Samples sc_9 and sc_10 (Dataset S1) contained a mixture of *Arabidopsis* and rice (*Oryza sativa* X. Kitaake) root protoplasts. We mapped the reads to a concatenated version of the *Arabidopsis* TAIR10 and rice MSU7 genomes and retained only the reads which specifically mapped to the *Arabidopsis* genome. The matrices of spliced and un-spliced counts were combined into a total count matrix. Genes with no counts in any cell were removed. Cells were filtered based on the following. First, putative dying cells were identified based on the enrichment of mitochondrial gene expression (> 5% of the total UMI counts) and the mode of the putative dying cells’ count distribution was treated as the initial boundary to separate cells into two groups representing low and high-quality cells. Second, expression profile references were built for both low and high-quality cells by taking the average of log-normalized counts. Third, the whole distribution of low-quality cells was recovered by comparing the Pearson correlation coefficient of each high-quality cell to the two references. In other words, if cells in the high-quality group have higher correlation to the low-quality cell profile than the high-quality cell profile, then those cells would be re-annotated as low quality. COPILOT offers functionality that allows iterative filtering until there are no cells more similar to the low-quality cell expression profile than the high-quality cell expression profile. However, in cases where the count distributions of high-quality cells and low-quality cells are not clearly separated, iterative filtering would result in over-filtering, which removes many cells that should be retained as high-quality cells. Therefore, to avoid over-filtering, we forced the algorithm to perform the cell filtering procedure only once. Finally, the low-quality cells and cells enriched in mitochondrial expression were removed along with the top 1% of high-quality cells in terms of total UMI counts in order to address any issues associated with outliers. In other words, after iterative filtering and removing cells having enriched mitochondrial expression, cells are further filtered for outliers. We used the top 1% of cells in terms of total UMI counts as a cut-off. Putative doublets were removed using DoubletFinder (McGinnis et al., 2019) with default parameters according to the estimated doublet rate (10X Genomics Chromium Single Cell 3’ Reagent Kit User Guide (v3 Chemistry)). This pre-processing pipeline is available as an R package, COPILOT (https://github.com/ohlerlab/COPILOT), with a jupyter notebook tutorial. In downstream analyses, we did not consider mitochondrial, chloroplast, or known protoplasting-affected genes (Denyer et al., 2019) (log2 fold-change >= 2 or <= −2 after protoplasting). These exclusions were biologically motivated with the goal to minimize noise that may affect dimensionality reduction or clustering. e.g., chloroplast development is repressed in roots and protoplasting causes stress-related gene expression changes.

#### Normalization and Dimensionality Reduction

Using Seurat version 3.1.5, data were normalized using the SCTransform method (Hafemeister and Satija, 2019) followed by principal component analysis (PCA) and non-linear dimensionality reduction using UMAP. Fifty principal components were calculated using the RunPCA function with parameters “approx” set to FALSE. UMAP embedding was generated by RunUMAP function using all 50 principal components with parameters n_neighbors = 30, min_dist = 0.3, umap.method = “umap-learn”, metric = “correlation”. All steps are incorporated into the COPILOT R package and a jupyter notebook demonstrating the analysis is provided (https://github.com/ohlerlab/COPILOT).

#### Integration of Seurat Objects

Data were integrated following the Seurat reference-based integration pipeline (Stuart et al., 2019; Butler et al., 2018). The sample with the highest median UMI/gene per cell and number of genes detected was chosen as the reference (sample name: sc_12; Dataset S1). Overall, 16 WT replicates were used to build the atlas, including three previously published samples (Dataset S1). A jupyter notebook demonstrating the integration process is available on Github (https://github.com/ohlerlab/COPILOT).

#### Plotted Gene Expression Values

‘Log-normalized’ indicates expression values extracted from the slot ‘data’ of a Seurat object’s ‘SCT’ assay, which contains the log-normalized, ‘corrected’ counts produced by the SCTransform function (Hafemeister and Satija, 2019). ‘Scaled Expression’ indicates batch-corrected, log-normalized values extracted from the slot ‘data’ of a Seurat object’s ‘integrated’ assay. These values are scaled such that any value above 10 is set to 10 (Stuart et al., 2019). However, the integrated assay only contains genes that are shared among all the samples that are integrated, which excluded some genes of interest. Therefore, given that the observed batch effect among our samples is small (Fig. S1), we chose to make several plots with expression values from the ‘data’ slot of a Seurat object’s ‘SCT’ assay.

#### Cell Type and Developmental Stage Annotation

The atlas annotation is based on comparison to published whole-transcriptome profiles (Brady et al., 2007a; Li et al., 2016) of root cells isolated from reporter lines as well as known markers (Dataset S1) that have been previously validated and show specific local expression on the atlas UMAP. We combined four annotation methods, described below.

#### Annotation Based on Spatial Mapping

We built a 3D root geometry reference based on confocal image stacks published with the interactive *Arabidopsis* root analysis tool iRoCS (Schmidt et al., 2014). The x, y and z confocal image coordination (in micrometers) of each cell’s centroid was manually documented as a location in 3D geometry followed by labeling of cell type, developmental zone, and distance from QC (in number of cells). The 3D root geometry records 3,957 cell locations covering 0.2 cm from the primary root tip (Fig. S2 and Dataset S1). A subset containing 50,000 atlas cells was mapped to the 3D root geometry using novoSpaRc (Nitzan et al., 2019) with default parameters and binarized spatial expression profiles of 49 markers based on published images of transcriptional reporters or in situ hybridizations (Dataset S1). These markers serve as anchors that bridge the scRNA-seq data to the root geometry. The mapping accuracy was estimated by performing left-one-out cross validation over 100 times. Average Pearson correlation of 0.7 was achieved between predictions of the mapped model and ground truth. The mapping information of each cell from the scRNA atlas to a location was extracted, and each cell was annotated according to its mapped location. Distal root cap refers to root cap cells located at the two outermost cell layers of root cap while proximal root cap cells include root cap cell layers closer to QC. This classification is based on the observation that the cells mapped to the outermost cell layer share the same top markers (Columella: AT3G61930; Lateral root cap: AT1G33280) with the cells mapped to the second outermost layer. The cell layers closer to QC share the same sets of markers as well (Columella: AT2G04025, AT1G78520; Lateral root cap: AT1G79580). AT3G61930 is treated as a marker for proximal and distal columella in the annotation method in the next section.

#### Marker Annotation

The enrichment scores of known cell type-specific markers (De Rybel et al., 2013; Schürholz et al., 2018; Muñiz et al., 2008; Menand et al., 2007; Bonke et al., 2003; Clay and Nelson, 2005; Lee and Schiefelbein, 2002; Brady et al., 2007b; Kamiya et al., 2016; Huang et al., 2017; Miyashima et al., 2019; Matsuzaki et al., 2010; Wallner et al., 2017; Ishida et al., 2009; Taniguchi et al., 2017; Kamiya et al., 2015; Aida et al., 2004; Kubo et al., 2005; Lee and Schiefelbein, 1999) (Dataset S1) were calculated for each cell in the atlas using SEMITONES (Vlot et al., 2020; github.com/ohlerlab/SEMITONES). SEMITONES uses cluster/reference-free, rank based statistics to calculate the significance of local enrichment of gene expression based on a distance between cells. Dimension reduction was performed on the raw cell-by-gene matrix and used to estimate the distance among cells to save computational resources. We chose UMAP to reduce dimensions, and distance among cells in the UMAP space was estimated via a radial basis function over the Euclidean distance (RBF kernel) metric. The size of a cell neighborhood was determined by setting the parameter “gamma” to 0.8. A gene is considered significantly enriched with respect to a cell if its enrichment score is more than 5 standard deviations away from the mean of the permutation null distribution. This permutation null distribution is obtained by applying enrichment scoring to 100 times permuted expression vectors. Cells were then annotated with a cell type label according to which significantly enriched marker had the highest enrichment score.

To complement the SEMITONES annotation approach, marker gene expression z-scores were calculated for a second marker annotation that depends on hard-clustering. In this approach, clusters were first defined using the Seurat FindClusters function by setting an extremely high modularity parameter (res = 500), which results in 3,034 clusters that only have tens of cells each. These finely-resolved clusters were then annotated by comparing the average marker gene z-scores. Cells that were annotated with the same cell identity by the SEMITONES and z-score approaches were considered confidently annotated. This combination was particularly useful to annotate very young cells at the base of the UMAP because it incorporates high resolution from the z-score approach with low noise from the SEMITONES annotation.

#### Correlation Annotation

Prior to scRNA-seq sample integration, Pearson correlation coefficient was calculated between each cell and whole-transcriptome reference expression profiles for cell types and developmental zones. We used bulk RNA-seq data (Li et al., 2016) previously generated for 14 cell types isolated with FACS. Further, we compared each cell in the atlas to ATH1 microarray data generated for thirteen cell types and thirteen tissue segments hand-dissected along the longitudinal axis of the root (Brady et al., 2007a). Each expression profile was built by first aligning the quality-filtered FASTQ reads, which are processed by Trimmomatic (Bolger et al., 2014) (v0.39) with default parameters and quality-checked by FastQC (Andrews, 2010) (v0.11.8), to the TAIR10 genome using STAR (Dobin and Gingeras, 2016) (v2.7.1a) with default parameters. Then, count normalization was carried out with DESeq and vst function in R package DESeq2 (Love et al., 2014) (v1.24.0) with default parameters. 181 genes that are highly variable across cell types in both RNA-seq and microarray data were kept, while 500 highly variable genes across 3 developmental zones and 809 highly variable genes across 13 developmental sections were selected, respectively. The SCTranform log-normalized counts in each cell and DEseq2 normalized counts in each expression profile were used to calculate Pearson correlation coefficient. Each cell was labeled with the cell type and developmental zone with which it had the highest correlation coefficient. We defined a high confidence annotation as correlation coefficient > 0.6.

#### Index of Cell Identity (ICI) Calculation

Another method to infer cell identity was an Index of Cell Identity (ICI)-based classification approach (Efroni et al., 2015). We identified 13 datasets (Birnbaum et al., 2003; Brady et al., 2007a; Li et al., 2016; Lee et al., 2006; Nawy et al., 2005; Clark et al., 2019; Dinneny et al., 2008; Gifford et al., 2008; Bargmann et al., 2013; Yadav et al., 2014; Birnbaum and Yuan, 2015) consisting of cell-type specific gene expression profiles (RNA-seq or ATH1 Microarray) for the 18 cell types considered for this atlas (Fig. S3; Dataset S3). RNA-seq data was preprocessed by adapter- and quality-trimming raw FASTAQ reads using the BBDuk tool (BBTools suite; sourceforge.net/projects/bbmap/), using adapter sequences found in the adapters.fa resource within bbtools, and parameters, k=23, mink=11, hdist=1, ktrim=r, and qtrim=10. Trimmed reads were mapped with the STAR (Dobin and Gingeras, 2016) utility (v2.7.2b) using default parameters with counts per gene quantified using the quantMode GeneCounts parameter. Read counts were then processed using the DESeq2 R package (Love et al., 2014) (v1.26.0), using a design matrix that treats datasets generated with the same marker:GFP construct as replicates, by running the estimateSizeFactors, estimateDispersions, and the vst functions to model gene expression. Microarray expression datasets were processed using the gcrma (Gentry et al., 2017) R package (v2.58.0). RNA-seq and microarray expression datasets were then harmonized using the FSQN (Franks et al., 2018) R package (v0.0.1) to model the RNA-seq gene expression distributions using the microarray data as a reference. FSQN-processed data from both the combined ATH1 and RNASeq datasets, as well as the DESeq2-processed RNASeq datasets alone, were then used to build two ICI specificity score (spec) tables (using the same methodology as described by Efroni and colleagues (2015), binning expression of each gene into 10 bins, with a minimum background bin set to 3). Markers were identified from this spec table, using a total information level of 50, and normalized, scaled expression of all identified markers was examined in all original datasets. Based on how well correlated each dataset was with its associated datasets of the same cell type, some datasets were filtered out. After dataset filtering, the final spec tables were re-calculated with the same parameters. The spec tables were then used (with an information level of 50) to compute ICI scores, p-values (using the permutation procedure described previously by Efroni et al., 2015) for all 18 cell types for cells in the atlas, using the log-transformed data values in the SCT assay of each individual dataset’s Seurat object. For each cell, the highest-scoring cell type (from either the combined ATH1/RNASeq or RNASeq only spec tables) was assigned as the ICI-derived annotation. We defined a high confidence annotation as adjusted p-value < 0.01.

#### Combination of Annotation Methods

Final cell type annotations were assigned by combining information from the four annotation approaches. For procambium, metaxylem, and protoxylem cell types, which lack bulk RNA-seq or microarray references, we used only spatial mapping and marker annotation methods. For the remaining cells, if a cell had the same label from at least two of the four annotation methods, then it was annotated as such. Otherwise, the cell was temporarily treated as un-annotated during the first final annotation step. In the second step, we leveraged information from Seurat by clustering with a low modularity parameter (res = 0.5) to further prune out noise. The resulting annotation (“consensus annotation”) represents the most confidently annotated cells. We built new reference expression profiles for each cell type by taking the average of the expression values for cells in the consensus annotation. All cells were then re-annotated using the correlation-based approach by comparison to these newly built references. The annotation of QC cells was performed separately since the correlation-based approach results in cells annotated as QC but that are enriched in expression of cell cycle genes (Dataset S1), which does not agree with the low cell division activity of the QC. In an alternative method, we identified 158 QC cells [~0.1 % of the atlas, which is similar to QC cell type proportions from microscopy data (Cartwright et al., 2009) that have high averaged z-scores of validated QC markers and low averaged z-scores of cell cycle genes in the SEMITONES-defined neighborhood with enriched expression of QC markers. Finally, we performed another round of denoising by clustering to obtain the final annotation.

To assign a developmental stage annotation to each cell, we used an approach similar to that described for cell type annotation, during which we used microarray-based whole-transcriptome profiles from thirteen root longitudinal sections as reference expression profiles (Brady et al., 2007a). Sections meristem 1–6, elongation 7 and 8, and maturation 9–12 correspond to the atlas meristem, elongation, and maturation labels, respectively. In practice, cell type and developmental stage annotations were performed simultaneously, meaning that the newly built references described in previous sections refer to the combination of developmental stage and cell type. A jupyter notebook demonstrating the annotation process is available from Github (https://github.com/ohlerlab/COPILOT).

#### Ploidy Annotation

We assigned each cell a ploidy label based on correlation with four published bulk RNA-seq profiles (Bhosale et al., 2018) (Fig. 2B; Dataset S1). A jupyter notebook demonstrating the annotation process is available from Github (https://github.com/ohlerlab/COPILOT).

#### Differentially Expressed Genes

To identify cell type and cell type + developmental stage marker genes, we used the default Wilcoxon test available from the Seurat FindMarkers function on scaled expression. Significant markers for cell type + developmental stage were selected based on the following criteria: adjusted p-value < 0.05, average log fold change > 3, and pct.dff > 0.4, where pct.diff is defined as the difference of gene percentage expression between the cluster considered and the rest of the cells. Genes that were identified as markers for multiple cell types were reassigned to the cell type with the highest average log fold change and pct.diff. Marker specificity was estimated by percentage expression in cells that do not belong to the cluster considered. The expression pattern of marker genes was also verified with Seurat’s dot plot tool.

In addition to cluster-dependent differential expression analysis implemented in Seurat, the cluster-agnostic tool SEMITONES was used to search for cell type + developmental stage marker genes *de novo* based on scaled expression. Reference cells for each cell type + developmental stage were chosen by searching for cells with the highest average similarity based on a similarity matrix calculated via the RBF kernel on 50 UMAP dimensions.

In DE analyses along pseudotime bins, we used the Seurat FindMarkers function to first prefilter features using a log2 fold-change threshold of 1 and a minimum percentage difference in expression of 0.25. We then performed differential expression testing for each combination of cell type and pseudotime bin using the ROC test implemented in Seurat FindMarkers. A classifier was built for each gene based on the ability of that gene’s expression level to distinguish between two groups of cells. The first group of cells corresponds to the pseudotime bin of interest within a particular cell type whereas the second group is the remaining cells within the trajectory for that tissue. Classification power based on Area under the ROC Curve (AUC) was used to estimate the performance of the classifier. An AUC value of 1 indicates increased expression values in the first group that can perfectly distinguish the two groupings, whereas an AUC of 0.5 indicates that the gene has no predictive power to distinguish the groups. Only markers with an AUC greater than 0.75 were retained for downstream analysis. We rank ordered markers based on AUC, percentage difference, and fold-change.

#### Bifurcation Patterns on Atlas UMAP

To examine bifurcation patterns within cell lineages, ground tissue and epidermis sub-branches were labeled based on clusters identified with Seurat (modularity parameter res = 0.5) (Fig. S4). Gene ontology analysis was conducted on identified DE genes using R package “gprofiler2” (Kolberg et al., 2020).

#### Pseudotime Estimation

Pseudotimes were inferred with the R package CytoTRACE (Gulati et al., 2020) (v0.1.0) and Python-based scVelo (Bergen et al., 2020) (v0.1.25). We opted not to use graph-based tools given their dependency on the selection of dimensional reduction embeddings and parameters. The batch-corrected and scaled (‘integrated’ assay in Seurat object) expression values were used as input for CytoTRACE and scVelo. Instead of using the default scaled expression values which were centered at 0 and capped at 10, all the negative values were treated as no expression and the values were floored at 0. The ratio of spliced and un-spliced transcripts of each gene and cell was calculated using raw counts. The ratio was then multiplied by the batch-corrected non-negative expression count matrix to generate the corresponding spliced and unspliced count matrices, which serve as input for scVelo. Latent time was then estimated by running pp.moments function with parameter, n_pcs = 50, n_neighbors = 100 and tl.velocity function with mode set to “dynamical” in scVelo, while CytoTRACE was implemented with default parameters.

A consensus pseudotime was derived by taking the average of CytoTRACE and scVelo-inferred latent time. Consensus time was estimated for each tissue/lineage independently to address differences in maturation rates. The consensus time for QC cells were then averaged and all the cells in the trajectory were divided into ten evenly sized groups (T0-T9) each containing the same number of cells. We chose ten bins after examining the data annotated according to correlation with microarray data from twelve manually dissected longitudinal tissue sections (Brady et al., 2007a). Of the twelve section labels, we found that two (Meristem-section 6 and Maturation-section12) were outliers and had fewer cells than the other ten sections. We therefore chose ten bins to more evenly spread the cells across all bins. A jupyter notebook demonstrating how results from the two tools were combined is provided under the GitHub repository for COPILOT (https://github.com/ohlerlab/COPILOT).

#### Genes Dynamically Expressed across Pseudotime

We applied the approach described under ‘Differentially expressed genes’ to identify genes that vary along the developmental progression of each tissue type. We used the combination of cell type and consensus time group (10 groups ranging from T0 to T9) as identity of interest among which differential expression analysis was performed. Spearman’s correlation of each marker with consensus time was considered as an additional metric to aid in selecting genes that vary along the gradient of differentiation. Ten genes were selected for each cell type and consensus time group combination. Genes were arranged according to their highest rank along consensus time. Pseudo-bulk expression profiles within each consensus time group were calculated for each gene and row scaled expression values were then displayed using ComplexHeatmap in R (Gu et al., 2016) (v2.10.0). All code used to identify and plot genes differentially expressed across pseudotime is available as a jupyter notebook on GitHub (https://github.com/ohlerlab/COPILOT).

#### Computing Trajectories with StationaryOT

Daily growth rates were estimated from imaging data of the growing meristem over a period of up to a week (Rahni and Birnbaum, 2019). Using these growth rates and examining the proportion of cells in each developmental stage, we estimated that roughly 5% of the cells in each lineage would be replaced in a 6-hour period. We selected the top 5% most differentiated cells from each lineage as sinks, as defined by pseudotime. We applied StationaryOT using entropic regularization with the regularization parameter set to ε = 0.025, and the cost matrix normalized to have unit mean. However, we found the results to be robust using quadratic regularization and varying the time and degree of regularization by a factor of two. Due to computational limitations, the dataset was partitioned into 10 subsets and StationaryOT was applied to each subset. This was repeated 10 times with random partitions to account for sampling error. Cell-by-cell averaging was performed on the computed fates to create a set of consensus fates. Between a single subset in a partition and the consensus fates in the full atlas, 97% of cells shared the same maximum fate type and the maximum fate values had a correlation of 0.96. Accounting for all fate values, rather than just maximums, the correlation rose to 0.99.

#### Visualizing Fate Probabilities

StationaryOT assigns a vector of fate probabilities to each cell. Up to three fates are visualized at a time (e.g., endodermis, cortex, and other being the sum of the remaining fates) using barycentric coordinates to represent 3-dimensional probability vectors in a two-dimensional triangle plot. A corner of the triangle is associated with each of these possible fates, and cells are positioned according to their relative probabilities as follows:

Let a, b, c denote the vertices of the triangle in *R*^2^ and let *p* = (*p*_1_, *p*_2_, *p*_3_) denote the probability vector we wish to visualize. The components of *p* are used as coefficients in a convex combination of the vertices. In other words, the probability vector *p* is mapped to *p*_1_*a* + *p*_2_*b* + *p*_3_*c* ∈ *R*^2^. Note that *p*_1_ + *p*_2_ + *p*_3_ = 1, so each probability vector is mapped to a point inside the triangle. Cells perfectly fated to obtain a single fate are positioned exactly at the corresponding vertex, while cells with indeterminate fates are positioned in the interior of the triangle. The very center of the triangle corresponds to cells that are equally likely to choose any of the three fates, and cells along an edge have zero chance of reaching the opposite vertex.

#### Lasso Regression

To identify genes that play roles in lineage determination, we applied Lasso regression to gene expression data and fate data from StationaryOT. This analysis was applied to cells in each developmental stage, and then the full dataset. As a result, we obtained lists of genes with possible lineage determining roles for each stage. Lasso is a linear regression method with an *L*^1^ regularization term to control sparsity (Tibshirani, 1996). We applied Lasso to gene expression matrices *E*_*s*_, for each developmental stage (meristem, elongation, maturation) and the full atlas. For the regressions, we restricted *E*_*s*_ to only contain expression data from transcription factors (Pruneda-Paz et al., 2014). Note that cells from the root cap were assigned meristematic, elongation, and maturation stage labels according to correlation annotation with bulk RNA-seq datasets generated from hand dissected tissue. This was done to create a fit that is applicable to all cell types, and hence is more selective in its component genes. The regression was performed on a lineage-by-lineage basis against *f*_*s*,*L*_, fate probabilities for cells from stage*s*, to a lineage *L*. In this setting, the objective function for Lasso is:

$$\frac{1}{2n}{\left|f_{s,L}-E_{s}w\right|}_{2}{}^{2}+\alpha |w{|}_{1}$$

Here, *n* is the number of cells, *w* is a vector of regression coefficients, and *α* is a regularization coefficient. To determine an optimal *α* for each regression, *α*_*opt*,*s*,*L*_, that balanced sparsity and predictive power, we tested a range of *α* for each stage and lineage. We created a graph of *R*^2^ versus the number of non-zero coefficients for each fit. We then chose *α*_*opt*,*s*,*L*_ by selecting the value of *α* corresponding to the knee point of the graph, which was determined using the Kneedle algorithm (Satopaa et al., 2011). An example of these graphs for the meristematic zone are found in Fig. S7. The linear_model. Lasso function from the Python package scikit-learn was used as the solver for the regressions (https://scikit-learn.org/stable/about.html#citing-scikit-learn). The regression assigns a coefficient *w*_*i*_ to each gene *i*, which determines its predicted impact on lineage determination. Specifically, the coefficient for a gene is a prediction of how much a unit change in that gene’s atlas expression values (after normalization and integration) affects a cell’s probability of achieving that lineage. Coefficients can be either positive or negative: a positive coefficient for a gene implies that up-regulation of that gene favors the given lineage, while a negative coefficient implies that down-regulation favors the lineage. The magnitude of these regression coefficients can be used to rank genes in terms of lineage determining capacity, providing candidates for further investigation. Gene lists for each stage and lineage are included in Dataset S5.

#### *shortroot* and *scarecrow* Mutant Analysis

Annotations were transferred from the atlas to two *scr-4* biological replicates, two *shr-2* biological replicates, and five wild type biological replicates that were grown and processed together with the mutants (WT samples sc20, 21, 30, 31, and 51). Label transfer was performed following the Seurat pipeline. A jupyter notebook tutorial is available on Github (github.com/Hsu-Che-Wei/COPILOT). Mutant and WT data were integrated following the Seurat reference-based integration pipeline (Stuart et al., 2019; Butler et al., 2018).

#### Cell Identity Differential Abundance

We used differential abundance analysis to examine which cell types were enriched or depleted in *shr* or *scr* compared to WT (Amezquita et al., 2020). First, we quantified the number of cells assigned to each label on a per sample basis. We then used the EdgeR package (Robinson et al., 2010; McCarthy et al., 2012) to fit a negative binomial generalized linear model in which the counts represent cells per label. Normalization was conducted according to the number of cells per sample. Separate contrasts were used to compare *shr* versus WT or *scr* versus WT, each with a blocking factor to account for any potential batch effects between different experimental runs. Differences in abundance were tested using the function glmQLFTest. P-values were adjusted for multiple testing according to Benjamini and Hochberg (1995) and cell type labels with a false discovery rate less than 0.05 were considered significantly altered. We then used ComplexHeatmap (Gu et al., 2016) in R to plot the log2 fold-change estimates (mutant/WT) from EdgeR.

### ADDITIONAL RESOURCES

Data deposition: https://www.ncbi.nlm.nih.gov/geo/

Interactive web browser for the atlas: https://phytozome-next.jgi.doe.gov/tools/scrna/

## Supplementary Material

1 **Data S2. scRNA-seq sample information and details related to annotation. Related to** Figs. 1–2 and 6–7, S1–S2, S4–S6, and STAR Methods. A) Experimental information for each scRNA-seq dataset from this study.
B) Comparisons between this study and previously published *Arabidopsis* root scRNA-seq datasets. Includes number of cells, median genes/cell, total number of genes detected, mean reads/cell, and technology used.
C) Total number of cells in the atlas annotated with each cell type and developmental stage combination.
D) Number of cells in the atlas annotated with each cell type label, separated by sample.
E) Number of cells in the atlas annotated with each developmental stage label, separated by sample.
F) Cell type-specific marker genes used for SEMITONES marker-based method of atlas annotation.
G) Cell type-specific marker genes used for spatial mapping method of atlas annotation (novoSpaRc).
H) 3D root geometry used in spatial mapping annotation (novoSpaRc). See STAR Methods for a detailed explanation. The 3D root geometry reference is based on confocal image stacks published with the iRoCS Toolbox. The x, y, and z confocal image coordinates of each cell’s centroid were manually documented as a location in the 3D geometry. Each cell was then labeled with cell type, number of cells away from the QC, and developmental zone. Layer indicates the longitudinal layer of the 3D geometry to which each cell belongs. The tip of the root is layer 1.
I) Spatial expression profiles of 49 markers used in spatial mapping annotation (novoSpaRc).
J) Published marker genes for cell cycle, ploidy, and differentially expressed genes identified in this study for the cell type sub-branches visible in ground tissue and epidermis.
K) Cell type markers identified from the atlas in this study with the FindMarkers function in Seurat (Wilcoxon test). Differentially expressed genes are ordered by descending log fold change and the top fifty genes for each cell type are shown.
L) Cell type + developmental stage markers identified from the atlas in this study with the FindMarkers function in Seurat (Wilcoxon test). Differentially expressed genes are ordered by descending log fold change and the top fifty genes for each cell type + developmental stage combination are shown.

2 Data S3. Metadata for each cell included in the WT atlas. Related to Figs. 1–2, Fig. S1, and STAR Methods.

3 **Data S4. Datasets and metadata used for Index of Cell Identity annotation. Related to** Figs. 1–2, Fig. S3, and STAR Methods. A) RNA-seq counts used to build the Index of Cell Identity specificity score (spec) table. B-D) Metadata for the ICI-based approach used to infer cell identity for atlas cells. B) ici_metadata is information for bulk RNA-seq data used to create ICI specificity score tables. C) included_cell_types is cell types included in the ICI. D) marker_metadata is metadata for published cell type-specific markers.

4 **Data S5. Correlation between two pseudotime inference methods and differentially expressed genes identified across consensus pseudotime for each tissue. Related to** Figs. 3–4 and STAR Methods. A) Correlation between CytoTRACE, scVelo latent time, and consensus time trajectory annotations for each tissue/lineage. Each value is the Pearson correlation coefficient. Negative correlations are due to differences in the scales implemented in the CytoTRACE and scVelo packages. For CytoTRACE, a value of 1 represents the most un-differentiated cells while for scVelo latent time and consensus time, a value of 1 represents the most differentiated cells. e.g., the ground tissue correlation between cytoTRACE and consensus time is −0.96, which represents a strong correlation but is negative due to CytoTRACE and consensus time scale differences.
B) Top differentially expressed genes identified between 10 consensus pseudotime groups for cortex and endodermis. These are all genes plotted on heatmap in Fig. 3G. See STAR Methods for details on differential expression analysis.
C) 9,777 differentially expressed genes identified between 10 consensus pseudotime groups for cortex and endodermis. See STAR Methods for details on differential expression analysis across pseudotime using the ROC method available from the Seurat FindMarkers function.
D) Top differentially expressed genes identified between 10 consensus pseudotime groups for lateral root cap, atrichoblast, and trichoblast. These are all genes plotted on heatmap in Fig. 4.
E) 4,216 differentially expressed genes identified between 10 consensus pseudotime groups for epidermis and lateral root cap.
F) Differentially expressed genes identified between 10 consensus pseudotime groups for xylem, phloem, procambium, and pericycle.
G) 7,474 differentially expressed genes identified between 10 consensus pseudotime groups for stele cell types.
H) Differentially expressed genes identified between 10 consensus pseudotime groups for columella.
I) 1,004 differentially expressed genes identified between 10 consensus pseudotime groups for columella.

5 **Data S6. Candidate developmental regulators identified for each cell type with StationaryOT and the Lasso regression. Related to** Fig. 5, Fig. S7, and STAR Methods. Transcription factors identified for each lineage with StationaryOT and the Lasso regression for all cells in the atlas (e.g., lineages were not subdivided into the three developmental zones), meristematic cells, elongation zone cells, and maturation zone cells. Transcription factors with possible lineage determining roles for each cell type are listed in individual tabs. The coefficient listed for each gene is a prediction of how much a unit change in that gene’s atlas expression values affects a cell’s probability of achieving the specific lineage. See STAR Methods for additional details. A positive coefficient implies that up-regulation of that gene favors the given lineage while a negative coefficient implies that down-regulation of that gene favors the lineage.

7

8 Supplementary Movie 1. Animation showing 3D UMAP of the atlas with cell type annotations. Related to Fig. 1.

## Figures and Tables

**Table:** KEY RESOURCES TABLE

REAGENT or RESOURCE	SOURCE	IDENTIFIER
Bacterial and Virus Strains		
N/A	N/A	N/A
Biological Samples		
*Arabidopsis thaliana* ecotype Columbia-0	N/A	N/A
*Arabidopsis: pCORTEX:erGFP*	Lee et al., 2006	N/A
*Arabidopsis: pMYB36:H2B:3xYFP*	Drapek et al., 2018	N/A
*Arabidopsis: scarecrow-4*	Fukaki et al., 1998	ABRC stock number CS6505
*Arabidopsis: shortroot-2*	Levesque et al., 2006	ABRC stock number CS2972
		
Chemicals, Peptides, and Recombinant Proteins		
Linsmaier and Skoog medium	Caisson Labs	Product number LSP03–1LT
Cellulase ONOZUKA R-10	GoldBio	Cat#C8001.0005
Pectolyase	Sigma	Cat#P3026
Bovine Serum Albumin	Sigma	Cat#A3912
β-mercaptoethanol	Sigma	Cat#M6250
Mannitol	Sigma	Cat#SLBV3117
MES	Sigma	Cat#6120
KCl	Fisher Scientific	Cat#AM9640G
CaCl_2_	Sigma	Cat#21115
Propidium iodide	Sigma	Cat#P4170
Basta (Glufosinate ammonium)	Fisher Scientific	Cat#J66186-MD
Critical Commercial Assays		
Chromium Single Cell Controller	10X Genomics	Product Code 120263
Chromium i7 Multiplex Kit	10X Genomics	Product Code 120262
Chromium Single Cell 3’ GEM Library & Gel Bead Kit v3	10X Genomics	Product Code 1000092
Chromium Chip B Single Cell Kit	10X Genomics	Product Code 1000074
DNA High Sensitivity Bioanalyzer Kit	Agilent	Cat#5067–4626
Deposited Data		
Single Cell mRNA Sequencing data	This Study	GSE152766
Single Cell RNA-Seq wild type *Arabidopsis* root cells - sc_1	This Study	GSM4625993
Single Cell RNA-Seq wild type *Arabidopsis* root cells - sc_9_at	This Study	GSM4625994
Single Cell RNA-Seq wild type *Arabidopsis* root cells - sc_10_at	This Study	GSM4625995
Single Cell RNA-Seq wild type *Arabidopsis* root cells - sc_11	This Study	GSM4625996
Single Cell RNA-Seq wild type *Arabidopsis* root cells - sc_12	This Study	GSM4625997
Single Cell RNA-Seq wild type *Arabidopsis* root cells - sc_20	This Study	GSM4625998
Single Cell RNA-Seq wild type *Arabidopsis* root cells - sc_21	This Study	GSM4625999
Single Cell RNA-Seq *scr* mutant *Arabidopsis* root cells - sc_25	This Study	GSM4626000
Single Cell RNA-Seq wild type *Arabidopsis* root cells - sc_30	This Study	GSM4626001
Single Cell RNA-Seq wild type *Arabidopsis* root cells - sc_31	This Study	GSM4626002
Single Cell RNA-Seq *scr* mutant *Arabidopsis* root cells - sc_36	This Study	GSM4626003
Single Cell RNA-Seq wild type *Arabidopsis* root cells - sc_37	This Study	GSM4626004
Single Cell RNA-Seq wild type *Arabidopsis* root cells - sc_40	This Study	GSM4626005
Single Cell RNA-Seq wild type *Arabidopsis* root cells - sc_51	This Study	GSM4626006
Single Cell RNA-Seq *shr* mutant *Arabidopsis* root cells - sc_52	This Study	GSM4626007
Single Cell RNA-Seq *shr* mutant *Arabidopsis* root cells - sc_53	This Study	GSM4626008
Single Cell RNA-Seq wild type *Arabidopsis* root cells – col0	This Study	GSM4626009
Single Cell RNA-Seq wild type *Arabidopsis* root cells – tnw1	This Study	GSM4626010
Single Cell RNA-Seq wild type *Arabidopsis* root cells – tnw2	This Study	GSM4626011
Experimental Models: Organisms/Strains		
*Arabidopsis thaliana*		
Oligonucleotides		
TCTCCATACCTCAAACTCCTCC	N/A	F genotyping primer for *shortroot-2*
TTGCCTCTCCGTCTACTGC	N/A	R genotyping primer for *shortroot-2*
CTTATCCATTCCTCAACTCTATT	Fukaki et al., 1998	F genotyping primer for *scarecrow-4*. Amplifies mutant allele.
TGGTGCATCGGTAGAAGAATT	Fukaki et al., 1998	R genotyping primer for *scarecrow-4*
TTATCCATTCCTCAACTTCAGT	Fukaki et al., 1998	F genotyping primer for *scarecrow-4*. Amplifies WT allele.
Recombinant DNA		
N/A	N/A	N/A
Software and Algorithms		
Cell Ranger v3.1.0	10X Genomics	https://support.10xgenomics.com/single-cell-gene-expression/software/pipelines/latest/installation
scKB	This Study	https://github.com/ohlerlab/scKB
COPILOT	This Study	https://github.com/ohlerlab/COPILOT
Seurat v3.1.5	Stuart et al., 2019; Butler et al., 2018	https://satijalab.org/seurat/
iRoCS	Schmidt et al., 2014	https://lmb.informatik.uni-freiburg.de/resources/opensource/iRoCS/
novoSpaRc	Nitzan et al., 2019	https://github.com/rajewsky-lab/novosparc
SEMITONES	Vlot et al., 2020	github.com/ohlerlab/SEMITONES
Trimmomatic v0.39.0	Bolger et al., 2014	http://www.usadellab.org/cms/?page=trimmomatic
FastQC v0.11.8	Andrews, 2010	https://www.bioinformatics.babraham.ac.uk/projects/fastqc/
STAR v2.7.1a & v2.7.2b	Dobin and Gingeras, 2016	https://github.com/alexdobin/STAR
DESeq2 v1.24.0 & v1.26.0	Love et al., 2014	https://bioconductor.org/packages/release/bioc/html/DESeq2.html
BBTools	Joint Genome Institute	https://jgi.doe.gov/data-and-tools/bbtools/
gcrma v2.58.0	Gentry et al., 2017	https://www.bioconductor.org/packages/release/bioc/html/gcrma.html
FSQN v0.0.1	Franks et al., 2018	https://github.com/jenniferfranks/FSQN/
gprofiler2 v0.2.1	Kolberg et al., 2020	https://cran.r-project.org/web/packages/gprofiler2/index.html
CytoTRACE v0.1.0	Gulati et al., 2020	https://cytotrace.stanford.edu/
scVelo v0.1.25	Bergen et al., 2020	https://scvelo.readthedocs.io/installation/
ComplexHeatmap v2.10.0	Gu et al., 2016	https://bioconductor.org/packages/release/bioc/html/ComplexHeatmap.html
StationaryOT	S. Zhang et al., 2021	
EdgeR v3.36.0	Robinson et al., 2010; McCarthy et al., 2012	https://bioconductor.org/packages/release/bioc/html/edgeR.html
Original Codes		
DOI:10.5281/zenodo.5775932	This Study	https://zenodo.org/badge/latestdoi/421176705

## Highlights

- scRNA-seq of >110,000 cells produced a comprehensive *Arabidopsis* root atlas
- Gradual, overlapping waves of gene expression underly development of all cell types
- Developmental trajectories enable visualization of cell specification events
- scRNA-seq of the *scarecrow* mutant reveals a cell identity change occurs over time

## References

[R1] Aida M, Beis D, Heidstra R, Willemsen V, Blilou I, Galinha C, Nussaume L, Noh Y-S, Amasino R, and Scheres B (2004). The PLETHORA genes mediate patterning of the Arabidopsis root stem cell niche. Cell 119, 109–120. 10.1016/j.cell.2004.09.018. 15454085

[R2] Amezquita RA, Lun ATL, Becht E, Carey VJ, Carpp LN, Geistlinger L, Marini F, Rue-Albrecht K, Risso D, Soneson C, (2020). Orchestrating single-cell analysis with Bioconductor. Nat Methods 17, 137–145. 10.1038/s41592-019-0654-x. 31792435 PMC7358058

[R3] Andrews S (2010). FastQC: A quality control tool for high throughput sequence data. https://www.bioinformatics.babraham.ac.uk/projects/fastqc/.

[R4] Bargmann BOR, Vanneste S, Krouk G, Nawy T, Efroni I, Shani E, Choe G, Friml J, Bergmann DC, Estelle M, and Birnbaum KD (2013). A map of cell type-specific auxin responses. Mol Syst Biol 9, 688. 10.1038/msb.2013.40. 24022006 PMC3792342

[R5] Beeckman T and De Smet I (2014). Pericycle. Curr Biol 24, R378–379. 10.1016/j.cub.2014.03.031. 24845660

[R6] Benfey PN, Linstead PJ, Roberts K, Schiefelbein JW, Hauser MT, and Aeschbacher RA (1993). Root development in Arabidopsis: four mutants with dramatically altered root morphogenesis. Development 119, 57–70. 8275864 10.1242/dev.119.Supplement.57

[R7] Benjamini Y and Hochberg Y (1995). Controlling the False Discovery Rate: A Practical and Powerful Approach to Multiple Testing. Journal of the Royal Statistical Society. Series B (Methodological) 57, 289–300.

[R8] Bergen V, Lange M, Peidli S, Wolf FA, and Theis FJ (2020). Generalizing RNA velocity to transient cell states through dynamical modeling. Nat Biotechnol 38, 1408–1414. 10.1038/s41587-020-0591-3. 32747759

[R9] Berger F, Haseloff J, Schiefelbein J, and Dolan L (1998a). Positional information in root epidermis is defined during embryogenesis and acts in domains with strict boundaries. Current Biology 8, 421–430. 10.1016/S0960-9822(98)70176-9. 9550701

[R10] Bhosale R, Boudolf V, Cuevas F, Lu R, Eekhout T, Hu Z, Isterdael GV, Lambert GM, Xu F, Nowack MK, (2018). A Spatiotemporal DNA Endoploidy Map of the Arabidopsis Root Reveals Roles for the Endocycle in Root Development and Stress Adaptation. The Plant Cell 30, 2330–2351. 10.1105/tpc.17.00983. 30115738 PMC6241279

[R11] Birnbaum K, Shasha DE, Wang JY, Jung JW, Lambert GM, Galbraith DW, and Benfey PN (2003). A gene expression map of the Arabidopsis root. Science 302, 1956–1960. 10.1126/science.1090022. 14671301

[R12] Birnbaum KD (2018). Power in Numbers: Single-Cell RNA-seq Strategies to Dissect Complex Tissues. Annu Rev Genet 52, 203–221. 10.1146/annurev-genet-120417-031247. 30192636 PMC6314027

[R13] Birnbaum KD and Kussell E (2011). Measuring cell identity in noisy biological systems. Nucleic Acids Res 39, 9093–9107. 10.1093/nar/gkr591. 21803789 PMC3241637

[R14] Birnbaum K and Yuan S (2015). Auxin induced endodermal to QC transdifferentiation time series and downstream of JKD analysis. GEO Accession viewer, https://www.ncbi.nlm.nih.gov/geo/query/acc.cgi?acc=GSE61408.

[R15] Bolger AM, Lohse M, and Usadel B (2014). Trimmomatic: a flexible trimmer for Illumina sequence data. Bioinformatics 30, 2114–2120. 10.1093/bioinformatics/btu170. 24695404 PMC4103590

[R16] Bonke M, Thitamadee S, Mähönen AP, Hauser M-T, and Helariutta Y (2003). APL regulates vascular tissue identity in Arabidopsis. Nature 426, 181–186. 10.1038/nature02100. 14614507

[R17] Bouché F (2017). Arabidopsis - Root cell types. figshare, 10.6084/m9.figshare.4688752.v1.

[R18] Brady SM, Orlando DA, Lee J-Y, Wang JY, Koch J, Dinneny JR, Mace D, Ohler U, and Benfey PN (2007a). A high-resolution root spatiotemporal map reveals dominant expression patterns. Science 318, 801–806. 10.1126/science.1146265. 17975066

[R19] Brady SM, Song S, Dhugga KS, Rafalski JA, and Benfey PN (2007b). Combining Expression and Comparative Evolutionary Analysis. The COBRA Gene Family. Plant Physiology 143, 172–187. 10.1104/pp.106.087262. 17098858 PMC1761980

[R20] Bray NL, Pimentel H, Melsted P, and Pachter L (2016). Near-optimal probabilistic RNA-seq quantification. Nature Biotechnology 34, 525–527. 10.1038/nbt.3519. 27043002

[R21] Briggs JA, Weinreb C, Wagner DE, Megason S, Peshkin L, Kirschner MW, and Klein AM (2018). The dynamics of gene expression in vertebrate embryogenesis at single-cell resolution. Science 360, eaar5780. 10.1126/science.aar5780. PMC6038144 29700227

[R22] Butler A, Hoffman P, Smibert P, Papalexi E, and Satija R (2018). Integrating single-cell transcriptomic data across different conditions, technologies, and species. Nat Biotechnol 36, 411–420. 10.1038/nbt.4096. 29608179 PMC6700744

[R23] Cao J, Spielmann M, Qiu X, Huang X, Ibrahim DM, Hill AJ, Zhang F, Mundlos S, Christiansen L, Steemers FJ, (2019). The single-cell transcriptional landscape of mammalian organogenesis. Nature 566, 496–502. 10.1038/s41586-019-0969-x. 30787437 PMC6434952

[R24] Carlsbecker A, Lee J-Y, Roberts CJ, Dettmer J, Lehesranta S, Zhou J, Lindgren O, Moreno-Risueno MA, Vatén A, Thitamadee S, (2010). Cell signalling by microRNA165/6 directs gene dose-dependent root cell fate. Nature 465, 316–321. 10.1038/nature08977. 20410882 PMC2967782

[R25] Cartwright DA, Brady SM, Orlando DA, Sturmfels B, and Benfey PN (2009). Reconstructing spatiotemporal gene expression data from partial observations. Bioinformatics 25, 2581–2587. 10.1093/bioinformatics/btp437. 19608707

[R26] Clark NM, Buckner E, Fisher AP, Nelson EC, Nguyen TT, Simmons AR, de Luis Balaguer MA, Butler-Smith T, Sheldon PJ, Bergmann DC, (2019). Stem-cell-ubiquitous genes spatiotemporally coordinate division through regulation of stem-cell-specific gene networks. Nat Commun 10, 5574. 10.1038/s41467-019-13132-2. 31811116 PMC6897965

[R27] Clay NK and Nelson T (2005). Arabidopsis thickvein Mutation Affects Vein Thickness and Organ Vascularization, and Resides in a Provascular Cell-Specific Spermine Synthase Involved in Vein Definition and in Polar Auxin Transport. Plant Physiology 138, 767–777. 10.1104/pp.104.055756. 15894745 PMC1150395

[R28] Cui H, Hao Y, Kovtun M, Stolc V, Deng X-W, Sakakibara H, and Kojima M (2011). Genome-wide direct target analysis reveals a role for SHORT-ROOT in root vascular patterning through cytokinin homeostasis. Plant Physiol 157, 1221–1231. 10.1104/pp.111.183178. 21951467 PMC3252171

[R29] Cui H (2015). Cortex proliferation in the root is a protective mechanism against abiotic stress. Plant Signal Behav 10, e1011949. 10.1080/15592324.2015.1011949. 26039471 PMC4622999

[R30] De Rybel B, Möller B, Yoshida S, Grabowicz I, Barbier de Reuille P, Boeren S, Smith RS, Borst JW, and Weijers D (2013). A bHLH complex controls embryonic vascular tissue establishment and indeterminate growth in Arabidopsis. Dev Cell 24, 426–437. 10.1016/j.devcel.2012.12.013. 23415953

[R31] Denyer T, Ma X, Klesen S, Scacchi E, Nieselt K, and Timmermans MCP (2019). Spatiotemporal Developmental Trajectories in the Arabidopsis Root Revealed Using High-Throughput Single-Cell RNA Sequencing. Dev Cell 48, 840–852.e5. 10.1016/j.devcel.2019.02.022. 30913408

[R32] Di Laurenzio L, Wysocka-Diller J, Malamy JE, Pysh L, Helariutta Y, Freshour G, Hahn MG, Feldmann KA, and Benfey PN (1996). The SCARECROW gene regulates an asymmetric cell division that is essential for generating the radial organization of the Arabidopsis root. Cell 86, 423–433. 10.1016/s0092-8674(00)80115-4. 8756724

[R33] Dinneny JR, Long TA, Wang JY, Jung JW, Mace D, Pointer S, Barron C, Brady SM, Schiefelbein J, and Benfey PN (2008). Cell identity mediates the response of Arabidopsis roots to abiotic stress. Science 320, 942–945. 10.1126/science.1153795. 18436742

[R34] Dobin A and Gingeras TR (2016). Optimizing RNA-Seq Mapping with STAR. Methods Mol Biol 1415, 245–262. 10.1007/978-1-4939-3572-7_13. 27115637

[R35] Dolan L, Janmaat K, Willemsen V, Linstead P, Poethig S, Roberts K, and Scheres B (1993). Cellular organisation of the Arabidopsis thaliana root. Development 119, 71–84. 8275865 10.1242/dev.119.1.71

[R36] Dorrity MW, Alexandre CM, Hamm MO, Vigil A-L, Fields S, Queitsch C, and Cuperus JT (2021). The regulatory landscape of Arabidopsis thaliana roots at single-cell resolution. Nat Commun 12, 3334. 10.1038/s41467-021-23675-y 34099698 PMC8184767

[R37] Drapek C, Sparks EE, and Benfey PN (2017). Uncovering Gene Regulatory Networks Controlling Plant Cell Differentiation. Trends Genet 33, 529–539. 10.1016/j.tig.2017.05.002. 28647055 PMC5522350

[R38] Drapek C, Sparks EE, Marhavy P, Taylor I, Andersen TG, Hennacy JH, Geldner N, and Benfey PN (2018). Minimum requirements for changing and maintaining endodermis cell identity in the Arabidopsis root. Nat Plants 4, 586–595. 10.1038/s41477-018-0213-y. 30061749 PMC6135099

[R39] Efroni I (2018). A Conceptual Framework for Cell Identity Transitions in Plants. Plant Cell Physiol 59, 691–701. 10.1093/pcp/pcx172. 29136202 PMC6018971

[R40] Efroni I and Birnbaum KD (2016). The potential of single-cell profiling in plants. Genome Biol 17, 65. 10.1186/s13059-016-0931-2. 27048384 PMC4820866

[R41] Efroni I, Ip P-L, Nawy T, Mello A, and Birnbaum KD (2015). Quantification of cell identity from single-cell gene expression profiles. Genome Biol 16, 9. 10.1186/s13059-015-0580-x. 25608970 PMC4354993

[R42] Esau K (1953). Plant Anatomy. (Wiley).

[R43] Farmer A, Thibivilliers S, Ryu KH, Schiefelbein J, and Libault M (2021). Single-nucleus RNA and ATAC sequencing reveals the impact of chromatin accessibility on gene expression in Arabidopsis roots at the single-cell level. Molecular Plant 14, 372–383. 10.1016/j.molp.2021.01.001. 33422696

[R44] Farrell JA, Wang Y, Riesenfeld SJ, Shekhar K, Regev A, and Schier AF (2018). Single-cell reconstruction of developmental trajectories during zebrafish embryogenesis. Science 360, eaar3131. 10.1126/science.aar3131. PMC6247916 29700225

[R45] Franks JM, Cai G, and Whitfield ML (2018). Feature specific quantile normalization enables cross-platform classification of molecular subtypes using gene expression data. Bioinformatics 34, 1868–1874. 10.1093/bioinformatics/bty026. 29360996 PMC5972664

[R46] Fukaki H, Wysocka-Diller J, Kato T, Fujisawa H, Benfey PN, and Tasaka M (1998). Genetic evidence that the endodermis is essential for shoot gravitropism in Arabidopsis thaliana. Plant J 14, 425–430. 10.1046/j.1365-313x.1998.00137.x. 9670559

[R47] Gala HP, Lanctot A, Jean-Baptiste K, Guiziou S, Chu JC, Zemke JE, George W, Queitsch C, Cuperus JT, and Nemhauser JL (2021). A single cell view of the transcriptome during lateral root initiation in Arabidopsis thaliana. Plant Cell 33, 2197–2220. 10.1093/plcell/koab101. 33822225 PMC8364244

[R48] Gentry JW, Irizarry R, and MacDonald J (2017). gcrma. Bioconductor, 10.18129/B9.BIOC.GCRMA.

[R49] Gifford ML, Dean A, Gutierrez RA, Coruzzi GM, and Birnbaum KD (2008). Cell-specific nitrogen responses mediate developmental plasticity. Proc Natl Acad Sci U S A 105, 803–808. 10.1073/pnas.0709559105. 18180456 PMC2206617

[R50] Gu Z, Eils R, and Schlesner M (2016). Complex heatmaps reveal patterns and correlations in multidimensional genomic data. Bioinformatics 32, 2847–2849. \ 10.1093/bioinformatics/btw313. 27207943

[R51] Gulati GS, Sikandar SS, Wesche DJ, Manjunath A, Bharadwaj A, Berger MJ, Ilagan F, Kuo AH, Hsieh RW, Cai S, (2020). Single-cell transcriptional diversity is a hallmark of developmental potential. Science 367, 405–411. 10.1126/science.aax0249. 31974247 PMC7694873

[R52] Hafemeister C and Satija R (2019). Normalization and variance stabilization of single-cell RNA-seq data using regularized negative binomial regression. Genome Biol 20, 296. 10.1186/s13059-019-1874-1. 31870423 PMC6927181

[R53] Hayashi K, Hasegawa J, and Matsunaga S (2013). The boundary of the meristematic and elongation zones in roots: endoreduplication precedes rapid cell expansion. Scientific Reports 3, 2723. 10.1038/srep02723. 24121463 PMC3796303

[R54] Heidstra R, Welch D, and Scheres B (2004). Mosaic analyses using marked activation and deletion clones dissect Arabidopsis SCARECROW action in asymmetric cell division. Genes Dev 18, 1964–1969. 10.1101/gad.305504. 15314023 PMC514176

[R55] Hong JH, Chu H, Zhang C, Ghosh D, Gong X, and Xu J (2015). A quantitative analysis of stem cell homeostasis in the Arabidopsis columella root cap. Frontiers in Plant Science 6, 206. 10.3389/fpls.2015.00206. 25870608 PMC4375977

[R56] Huang L, Shi X, Wang W, Ryu KH, and Schiefelbein J (2017). Diversification of Root Hair Development Genes in Vascular Plants. Plant Physiology 174, 1697–1712. \ 10.1104/pp.17.00374. 28487476 PMC5490906

[R57] Ishida T, Fujiwara S, Miura K, Stacey N, Yoshimura M, Schneider K, Adachi S, Minamisawa K, Umeda M, and Sugimoto K (2009). SUMO E3 Ligase HIGH PLOIDY2 Regulates Endocycle Onset and Meristem Maintenance in Arabidopsis. The Plant Cell 21, 2284–2297. 10.1105/tpc.109.068072. 19666737 PMC2751947

[R58] Jean-Baptiste K, McFaline-Figueroa JL, Alexandre CM, Dorrity MW, Saunders L, Bubb KL, Trapnell C, Fields S, Queitsch C, and Cuperus JT (2019). Dynamics of Gene Expression in Single Root Cells of Arabidopsis thaliana. Plant Cell 31, 993–1011. 10.1105/tpc.18.00785. 30923229 PMC8516002

[R59] Kamimoto K, Hoffmann CM, and Morris SA (2020). CellOracle: Dissecting cell identity via network inference and in silico gene perturbation. bioRxiv, 10.1101/2020.02.17.947416. PMC9946838 36755098

[R60] Kamiya M, Higashio S-Y, Isomoto A, Kim J-M, Seki M, Miyashima S, and Nakajima K (2016). Control of root cap maturation and cell detachment by BEARSKIN transcription factors in Arabidopsis. Development 143, 4063–4072. 10.1242/dev.142331. 27803060

[R61] Kamiya T, Borghi M, Wang P, Danku JMC, Kalmbach L, Hosmani PS, Naseer S, Fujiwara T, Geldner N, and Salt DE (2015). The MYB36 transcription factor orchestrates Casparian strip formation. PNAS 112, 10533–10538. 10.1073/pnas.1507691112. 26124109 PMC4547244

[R62] Kim H, Zhou J, Kumar D, Jang G, Ryu KH, Sebastian J, Miyashima S, Helariutta Y, and Lee J-Y (2020). SHORTROOT-Mediated Intercellular Signals Coordinate Phloem Development in Arabidopsis Roots. Plant Cell 32, 1519–1535. 10.1105/tpc.19.00455. 32111671 PMC7203941

[R63] Kolberg L, Raudvere U, Kuzmin I, Vilo J, and Peterson H (2020). gprofiler2 -- an R package for gene list functional enrichment analysis and namespace conversion toolset g:Profiler. F1000Res 9, ELIXIR-709. 10.12688/f1000research.24956.2 PMC7859841 33564394

[R64] Kubo M, Udagawa M, Nishikubo N, Horiguchi G, Yamaguchi M, Ito J, Mimura T, Fukuda H, and Demura T (2005). Transcription switches for protoxylem and metaxylem vessel formation. Genes Dev. 19, 1855–1860. 10.1101/gad.1331305. 16103214 PMC1186185

[R65] Lee J-Y, Colinas J, Wang JY, Mace D, Ohler U, and Benfey PN (2006). Transcriptional and posttranscriptional regulation of transcription factor expression in Arabidopsis roots. PNAS 103, 6055–6060. 10.1073/pnas.0510607103. 16581911 PMC2111400

[R66] Lee MM and Schiefelbein J (2002). Cell Pattern in the Arabidopsis Root Epidermis Determined by Lateral Inhibition with Feedback. The Plant Cell 14, 611–618. 10.1105/tpc.010434. 11910008 PMC150583

[R67] Lee MM and Schiefelbein J (1999). WEREWOLF, a MYB-related protein in Arabidopsis, is a position-dependent regulator of epidermal cell patterning. Cell 99, 473–483. 10.1016/s0092-8674(00)81536-6. 10589676

[R68] Levesque MP, Vernoux T, Busch W, Cui H, Wang JY, Blilou I, Hassan H, Nakajima K, Matsumoto N, Lohmann JU, (2006). Whole-genome analysis of the SHORT-ROOT developmental pathway in Arabidopsis. PLoS Biology 4, e143. 10.1371/journal.pbio.0040143. 16640459 PMC1450008

[R69] Li S, Yamada M, Han X, Ohler U, and Benfey PN (2016). High resolution expression map of the Arabidopsis root reveals alternative splicing and lincRNA regulation. Dev Cell 39, 508–522. 10.1016/j.devcel.2016.10.012. 27840108 PMC5125536

[R70] Liberman LM, Sparks EE, Moreno-Risueno MA, Petricka JJ, and Benfey PN (2015). MYB36 regulates the transition from proliferation to differentiation in the Arabidopsis root. PNAS 112, 12099–12104. 10.1073/pnas.1515576112. 26371322 PMC4593085

[R71] Love MI, Huber W, and Anders S (2014). Moderated estimation of fold change and dispersion for RNA-seq data with DESeq2. Genome Biol 15, 550. 10.1186/s13059-014-0550-8. 25516281 PMC4302049

[R72] Lucas M, Swarup R, Paponov IA, Swarup K, Casimiro I, Lake D, Peret B, Zappala S, Mairhofer S, Whitworth M, (2011). Short-Root regulates primary, lateral, and adventitious root development in Arabidopsis. Plant Physiol 155, 384–398. 10.1104/pp.110.165126. 21030506 PMC3075784

[R73] Marjanovic ND, Hofree M, Chan JE, Canner D, Wu K, Trakala M, Hartmann GG, Smith OC, Kim JY, Evans KV, (2020). Emergence of a High-Plasticity Cell State during Lung Cancer Evolution. Cancer Cell 38, 229–246.e13. 10.1016/j.ccell.2020.06.012. 32707077 PMC7745838

[R74] Massri AJ, Greenstreet L, Afanassiev A, Berrio A, Wray GA, Schiebinger G, and McClay DR (2021). Developmental single-cell transcriptomics in the Lytechinus variegatus sea urchin embryo. Development 148, dev198614. 10.1242/dev.198614. PMC8502253 34463740

[R75] Matsuzaki Y, Ogawa-Ohnishi M, Mori A, and Matsubayashi Y (2010). Secreted peptide signals required for maintenance of root stem cell niche in Arabidopsis. Science 329, 1065–1067. 10.1126/science.1191132. 20798316

[R76] McCarthy DJ, Chen Y, and Smyth GK (2012). Differential expression analysis of multifactor RNA-Seq experiments with respect to biological variation. Nucleic Acids Res 40, 4288–4297. 10.1093/nar/gks042. 22287627 PMC3378882

[R77] McFaline-Figueroa JL, Trapnell C, and Cuperus JT (2020). The promise of single-cell genomics in plants. Curr Opin Plant Biol 54, 114–121. 10.1016/j.pbi.2020.04.002. 32388018 PMC7971421

[R78] McGinnis CS, Murrow LM, and Gartner ZJ (2019). DoubletFinder: Doublet Detection in Single-Cell RNA Sequencing Data Using Artificial Nearest Neighbors. Cell Syst 8, 329–337.e4. 10.1016/j.cels.2019.03.003. 30954475 PMC6853612

[R79] Melsted P, Ntranos V, and Pachter L (2019). The barcode, UMI, set format and BUStools. Bioinformatics 35, 4472–4473. 10.1093/bioinformatics/btz279. 31073610

[R80] Menand B, Yi K, Jouannic S, Hoffmann L, Ryan E, Linstead P, Schaefer DG, and Dolan L (2007). An ancient mechanism controls the development of cells with a rooting function in land plants. Science 316, 1477–1480. 10.1126/science.1142618. 17556585

[R81] Miyashima S, Roszak P, Sevilem I, Toyokura K, Blob B, Heo J-O, Mellor N, Help-Rinta-Rahko H, Otero S, Smet W, (2019). Mobile PEAR transcription factors integrate positional cues to prime cambial growth. Nature 565, 490–494. 10.1038/s41586-018-0839-y. 30626969 PMC7617008

[R82] Monge G 1781. Mé moire sur la thé orie des dé blais et des remblais. Mé m de l’Ac R des Sc, 666–704.

[R83] Moses L and Pachter L (2020). BUSpaRse: kallisto bustools R utilities. R package version 1.2.2 Github, https://github.com/BUStools/BUSpaRse.

[R84] Muñiz L, Minguet EG, Singh SK, Pesquet E, Vera-Sirera F, Moreau-Courtois CL, Carbonell J, Blázquez MA, and Tuominen H (2008). ACAULIS5 controls Arabidopsis xylem specification through the prevention of premature cell death. Development 135, 2573–2582. 10.1242/dev.019349. 18599510

[R85] Nawy T, Lee J-Y, Colinas J, Wang JY, Thongrod SC, Malamy JE, Birnbaum K, and Benfey PN (2005). Transcriptional Profile of the Arabidopsis Root Quiescent Center. Plant Cell 17, 1908–1925. 10.1105/tpc.105.031724. 15937229 PMC1167541

[R86] Nitzan M, Karaiskos N, Friedman N, and Rajewsky N (2019). Gene expression cartography. Nature 576, 132–137. 10.1038/s41586-019-1773-3. 31748748

[R87] O’Malley RC, Huang SC, Song L, Lewsey MG, Bartlett A, Nery JR, Galli M, Gallavotti A, and Ecker JR (2016). Cistrome and Epicistrome Features Shape the Regulatory DNA Landscape. Cell 165, 1280–1292. 10.1016/j.cell.2016.04.038. 27203113 PMC4907330

[R88] Pagès H (2020). BSgenome: Software infrastructure for efficient representation of full genomes and their SNPs. Bioconductor, 10.18129/B9.bioc.BSgenome.

[R89] Pruneda-Paz JL, Breton G, Nagel DH, Kang SE, Bonaldi K, Doherty CJ, Ravelo S, Galli M, Ecker JR, and Kay SA (2014). A genome-scale resource for the functional characterization of Arabidopsis transcription factors. Cell Rep 8, 622–632. 10.1016/j.celrep.2014.06.033. 25043187 PMC4125603

[R90] Rahni R and Birnbaum KD (2019). Week-long imaging of cell divisions in the Arabidopsis root meristem. Plant Methods 15, 30. 10.1186/s13007-019-0417-9. 30988691 PMC6446972

[R91] Rautenstrauch P, Vlot AHC, Saran S, and Ohler U (2021). Intricacies of single-cell multi-omics data integration. Trends Genet. 10.1016/j.tig.2021.08.012 34561102

[R92] Robinson MD, McCarthy DJ, and Smyth GK (2010). edgeR: a Bioconductor package for differential expression analysis of digital gene expression data. Bioinformatics 26, 139–140. 10.1093/bioinformatics/btp616. 19910308 PMC2796818

[R93] Ryu KH, Huang L, Kang HM, and Schiefelbein J (2019). Single-Cell RNA Sequencing Resolves Molecular Relationships Among Individual Plant Cells. Plant Physiol 179, 1444–1456. 10.1104/pp.18.01482. 30718350 PMC6446759

[R94] Satopaa V, Albrecht J, Irwin D, and Raghavan B (2011). Finding a “Kneedle” in a Haystack: Detecting Knee Points in System Behavior. Presented at the 2011 31st International Conference on Distributed Computing Systems Workshops, 166–171. 10.1109/ICDCSW.2011.20.

[R95] Schiebinger G, Shu J, Tabaka M, Cleary B, Subramanian V, Solomon A, Gould J, Liu S, Lin S, Berube P, (2019). Optimal-Transport Analysis of Single-Cell Gene Expression Identifies Developmental Trajectories in Reprogramming. Cell 176, 928–943.e22. 10.1016/j.cell.2019.01.006. 30712874 PMC6402800

[R96] Schiefelbein J, Huang L, and Zheng X (2014). Regulation of epidermal cell fate in Arabidopsis roots: the importance of multiple feedback loops. Front Plant Sci 5, Article 47. 10.3389/fpls.2014.00047. PMC3925829 24596575

[R97] Schmidt T, Pasternak T, Liu K, Blein T, Aubry-Hivet D, Dovzhenko A, Duerr J, Teale W, Ditengou FA, Burkhardt H, (2014). The iRoCS Toolbox--3D analysis of the plant root apical meristem at cellular resolution. Plant J 77, 806–814. 10.1111/tpj.12429. 24417645

[R98] Schürholz A-K, López-Salmerón V, Li Z, Forner J, Wenzl C, Gaillochet C, Augustin S, Barro AV, Fuchs M, Gebert M, (2018). A Comprehensive Toolkit for Inducible, Cell Type-Specific Gene Expression in Arabidopsis. Plant Physiol 178, 40–53. 10.1104/pp.18.00463. 30026289 PMC6130011

[R99] Serrano-Ron L, Cabrera J, Perez-Garcia P, and Moreno-Risueno MA (2021). Unraveling Root Development Through Single-Cell Omics and Reconstruction of Gene Regulatory Networks. Front. Plant Sci 12, Article 661361. 10.3389/fpls.2021.661361. PMC8129646 34017350

[R100] Shulse CN, Cole BJ, Ciobanu D, Lin J, Yoshinaga Y, Gouran M, Turco GM, Zhu Y, O’Malley RC, Brady SM, and Dickel DE (2019). High-Throughput Single-Cell Transcriptome Profiling of Plant Cell Types. Cell Rep 27, 2241–2247.e4. 10.1016/j.celrep.2019.04.054. 31091459 PMC6758921

[R101] Stuart T, Butler A, Hoffman P, Hafemeister C, Papalexi E, Mauck WM, Hao Y, Stoeckius M, Smibert P, and Satija R (2019). Comprehensive Integration of Single-Cell Data. Cell 177, 1888–1902.e21. 10.1016/j.cell.2019.05.031. 31178118 PMC6687398

[R102] Stuart T and Satija R (2019). Integrative single-cell analysis. Nat Rev Genet 20, 257–272. 10.1038/s41576-019-0093-7. 30696980

[R103] Taniguchi M, Furutani M, Nishimura T, Nakamura M, Fushita T, Iijima K, Baba K, Tanaka H, Toyota M, Tasaka M, and Morita MT (2017). The Arabidopsis LAZY1 Family Plays a Key Role in Gravity Signaling within Statocytes and in Branch Angle Control of Roots and Shoots. Plant Cell 29, 1984–1999. 10.1105/tpc.16.00575. 28765510 PMC5590491

[R104] Tibshirani R (1996). Regression Shrinkage and Selection via the Lasso. Journal of the Royal Statistical Society. Series B (Methodological) 58, 267–288.

[R105] Vlot AHC, Maghsudi S, and Ohler U (2020). SEMITONES: Single-cEll Marker IdentificaTiON by Enrichment Scoring. bioRxiv, 10.1101/2020.11.17.386664.

[R106] von Wangenheim D, Fangerau J, Schmitz A, Smith RS, Leitte H, Stelzer EHK, and Maizel A (2016). Rules and Self-Organizing Properties of Post-embryonic Plant Organ Cell Division Patterns. Curr Biol 26, 439–449. 10.1016/j.cub.2015.12.047. 26832441

[R107] Wallner E-S, López-Salmerón V, Belevich I, Poschet G, Jung I, Grünwald K, Sevilem I, Jokitalo E, Hell R, Helariutta Y, (2017). Strigolactone- and Karrikin-Independent SMXL Proteins Are Central Regulators of Phloem Formation. Current Biology 27, 1241–1247. 10.1016/j.cub.2017.03.014. 28392107 PMC5405109

[R108] Wendrich JR, Möller BK, Li S, Saiga S, Sozzani R, Benfey PN, Rybel BD, and Weijers D (2017). Framework for gradual progression of cell ontogeny in the Arabidopsis root meristem. PNAS 114, E8922–E8929. 10.1073/pnas.1707400114. 28973915 PMC5651754

[R109] Wendrich JR, Yang B, Vandamme N, Verstaen K, Smet W, Van de Velde C, Minne M, Wybouw B, Mor E, Arents HE, (2020). Vascular transcription factors guide plant epidermal responses to limiting phosphate conditions. Science 370, eaay4970. 10.1126/science.aay4970. PMC7116379 32943451

[R110] Yadav RK, Tavakkoli M, Xie M, Girke T, and Reddy GV (2014). A high-resolution gene expression map of the Arabidopsis shoot meristem stem cell niche. Development 141, 2735–2744. 10.1242/dev.106104. 24961803

[R111] Yu N-I, Lee SA, Lee M-H, Heo J-O, Chang KS, and Lim J (2010). Characterization of SHORT-ROOT function in the Arabidopsis root vascular system. Mol Cells 30, 113–119. 10.1007/s10059-010-0095-y. 20680487

[R112] Zhang S, Afanassiev A, Greenstreet L, Matsumoto T, and Schiebinger G (2021). Optimal transport analysis reveals trajectories in steady-state systems. PLoS Comput Biol 17, e1009466. 10.1371/journal.pcbi.1009466. 34860824 PMC8691649

[R113] Zhang T-Q, Xu Z-G, Shang G-D, and Wang J-W (2019). A Single-Cell RNA Sequencing Profiles the Developmental Landscape of Arabidopsis Root. Mol Plant 12, 648–660. 10.1016/j.molp.2019.04.004. 31004836

