# Supplementary material for: A single cell Arabidopsis root atlas reveals developmental trajectories in wild type and cell identity mutants
Source: Dev Cell. Author manuscript; Available in PMC 2022 Apr 18. (PMC9014886; doi:10.1016/j.devcel.2022.01.008)

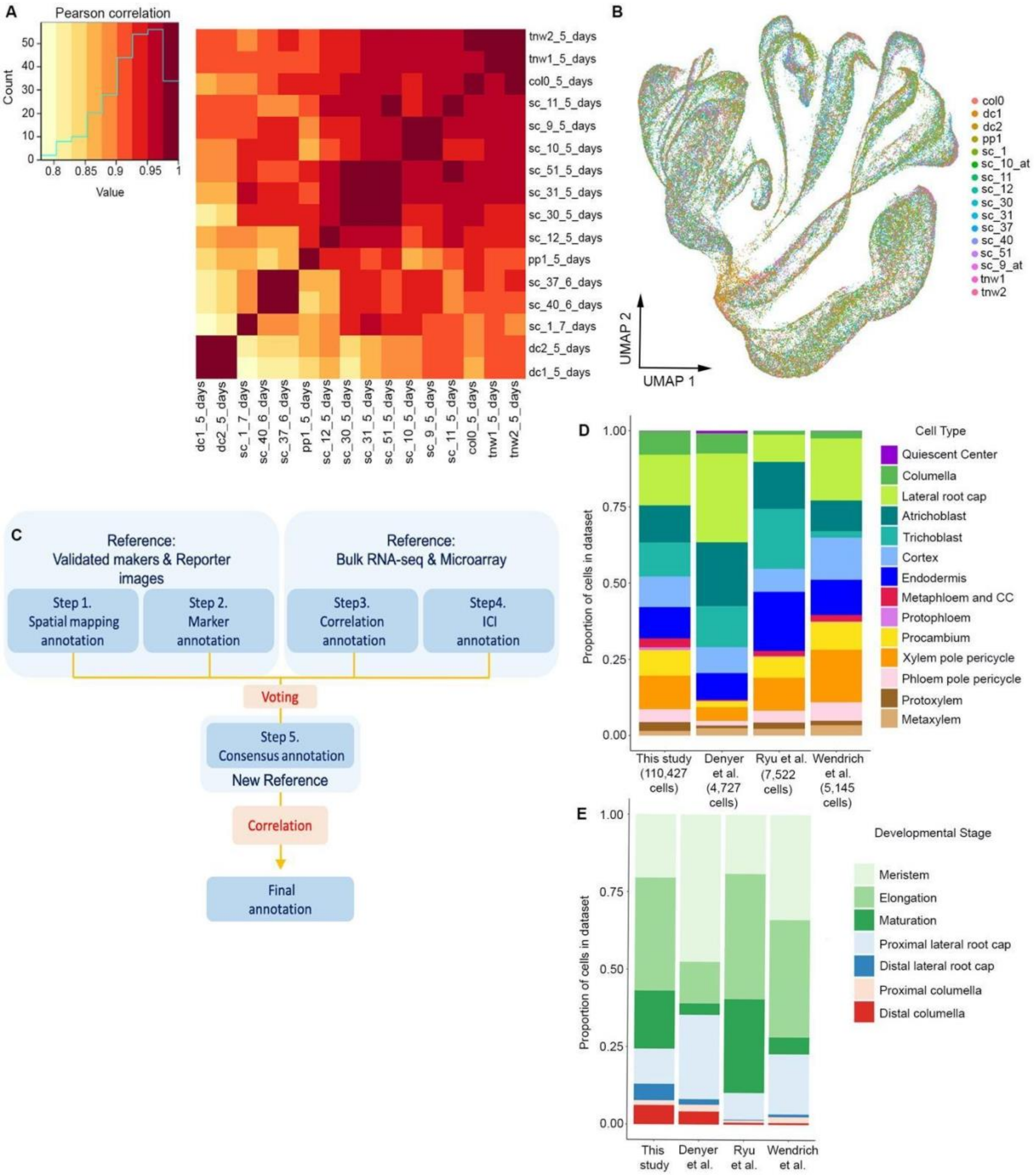
A
Pearson correlation
Count
Value
0.8 0.85 0.9 0.95 1
0 10 20 30 40 50
tnw2_5_days
tnw1_5_days
col0_5_days
sc_11_5_days
sc_9_5_days
sc_10_5_days
sc_51_5_days
sc_31_5_days
sc_30_5_days
sc_12_5_days
pp1_5_days
sc_37_6_days
sc_40_6_days
sc_1_7_days
dc2_5_days
dc1_5_days
B
UMAP 1
UMAP 2
col0
dc1
dc2
pp1
sc_1
sc_10_at
sc_11
sc_12
sc_30
sc_31
sc_37
sc_40
sc_51
sc_9_at
tnw1
tnw2
C
Reference:
Validated makers & Reporter images
Reference:
Bulk RNA-seq & Microarray
Step 1. Spatial mapping annotation
Step 2. Marker annotation
Step3. Correlation annotation
Step4. ICI annotation
Voting
Step 5. Consensus annotation
New Reference
Correlation
Final annotation
D
Proportion of cells in dataset
0.00 0.25 0.50 0.75 1.00
This study (110,427 cells)
Denyer et al. (4,727 cells)
Ryu et al. (7,522 cells)
Wendrich et al. (5,145 cells)
Cell Type
Quiescent Center
Columella
Lateral root cap
Atrichoblast
Trichoblast
Cortex
Endodermis
Metaphloem and CC
Protophloem
Procambium
Xylem pole pericycle
Phloem pole pericycle
Protoxylem
Metaxylem
E
Proportion of cells in dataset
0.00 0.25 0.50 0.75 1.00
This study
Denyer et al.
Ryu et al.
Wendrich et al.
Developmental Stage
Meristem
Elongation
Maturation
Proximal lateral root cap
Distal lateral root cap
Proximal columella
Distal columella

Figure S1. Combination and annotation of 110,427 cells to create a WT root atlas. Related to Figures 1-2, Datasets S1-S3, Data S1, and STAR Methods.

A) The atlas contains sixteen WT scRNA-seq samples with highly correlated gene expression profiles. Despite differences in sample age and lab of origin, Pearson's correlation coefficient is greater than 0.75 for all sample comparisons and greater than 0.8 for all but one comparison. Samples dc1 and dc2 were published by Denyer et al. (2019). Sample pp1 was published by Ryu et al. (2019). All other samples were produced for this study.

B) After reference-based scRNA-seq dataset integration, cells from sixteen WT replicates are well mixed. Each cell on the 2D UMAP is colored based on the sample of origin. Orange cells representing dc1 and dc2 samples (Denyer et al., 2019) are enriched at the base of the UMAP, potentially due to protoplasting conditions that enriched meristematic cells.

C) Four annotation methods were used, with either marker gene expression or bulk RNA-seq/microarray data as a reference. The results of the four methods were combined into a consensus annotation representing confidently annotated labels. This consensus annotation then served as a new reference for the final round of correlation-based annotation.

D) Representation of cell types in this study as compared to three previous *Arabidopsis* root scRNA-seq atlases. To facilitate a fair comparison, raw data from Denyer et al. (2019), Ryu et al. (2019), and Wendrich et al. (2020) were filtered with the COPILOT pipeline from this study. Annotation labels from the atlas in this study were then transferred to the three previously published atlases using the Seurat label transfer functionality. Proportion of cells with each cell type annotation label in each dataset is shown. Total numbers of cells in each dataset after filtering by COPILOT are shown in the x-axis labels.

E) Representation of developmental stages in the atlas from this study as compared to previous root atlases. For cell type labels in **D)**, developmental stage labels were transferred from the atlas in this study to the three previously published datasets. Numbers of cells in each dataset after filtering by COPILOT are the same as in **D**.

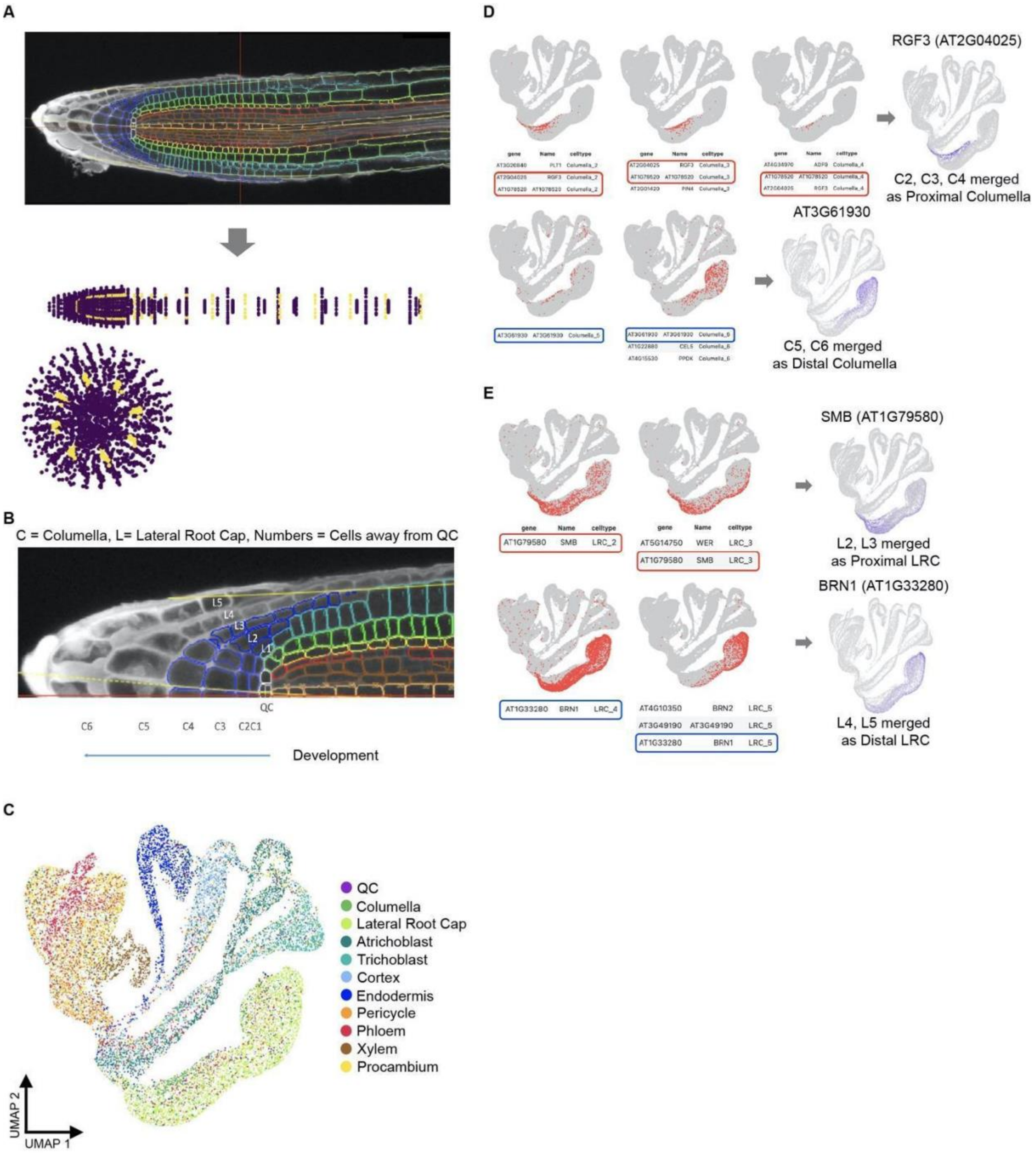
A
B
C = Columella, L= Lateral Root Cap, Numbers = Cells away from QC
L5
L4
L3
L2
L1
QC
C6
C5
C4
C3
C2C1
Development
C
QC
Columella
Lateral Root Cap
Atrichoblast
Trichoblast
Cortex
Endodermis
Pericycle
Phloem
Xylem
Procambium
UMAP 2
UMAP 1
D
gene Name celltype
AT3G20840 PLT1 Columella_2
AT2G04025 RGF3 Columella_2
AT1G78520 AT1G78520 Columella_2
gene Name celltype
AT2G04025 RGF3 Columella_3
AT1G78520 AT1G78520 Columella_3
AT2G01420 PIN4 Columella_3
gene Name celltype
AT4G34970 ADF9 Columella_4
AT1G78520 AT1G78520 Columella_4
AT2G04025 RGF3 Columella_4
RGF3 (AT2G04025)
C2, C3, C4 merged
as Proximal Columella
AT3G61930 AT3G61930 Columella_5
AT3G61930 AT3G61930 Columella_6
AT1G22880 CEL5 Columella_6
AT4G15530 PPDK Columella_6
AT3G61930
C5, C6 merged
as Distal Columella
E
gene Name celltype
AT1G79580 SMB LRC_2
gene Name celltype
AT5G14750 WER LRC_3
AT1G79580 SMB LRC_3
SMB (AT1G79580)
L2, L3 merged
as Proximal LRC
AT1G33280 BRN1 LRC_4
AT4G10350 BRN2 LRC_5
AT3G49190 AT3G49190 LRC_5
AT1G33280 BRN1 LRC_5
BRN1 (AT1G33280)
L4, L5 merged
as Distal LRC

Figure S2. Spatial mapping annotation. **Related to Dataset S1 and STAR Methods.**

A) Based on confocal image stacks from the iRoCs Toolbox (Schmidt et al., 2014), we built a 3D geometry for 0.2 cm of root tissue as measured from the tip. In the model, the spatial expression of a cortex marker AT1G62510 is shown in yellow for both longitudinal and radial sections.

B) Root cap developmental stage labels were assigned to the 3D geometry based on distance from the QC.

C) Cell type annotation that represents atlas cells mapped to 3D root geometry locations using novoSpaRc.

D) Cells mapped to different layers of columella and the associated top markers identified for each layer. Based on the shared markers, layers 2, 3, and 4 were merged into a single label (proximal columella) while layers 5 and 6 were merged into a second label (distal columella).

E) Cells mapped to different layers of lateral root cap and the associated top markers identified for each layer. Based on the shared markers, layers 2 and 3 were merged into a single label (proximal lateral root cap) while layers 4 and 5 were merged into a second label (distal lateral root cap).

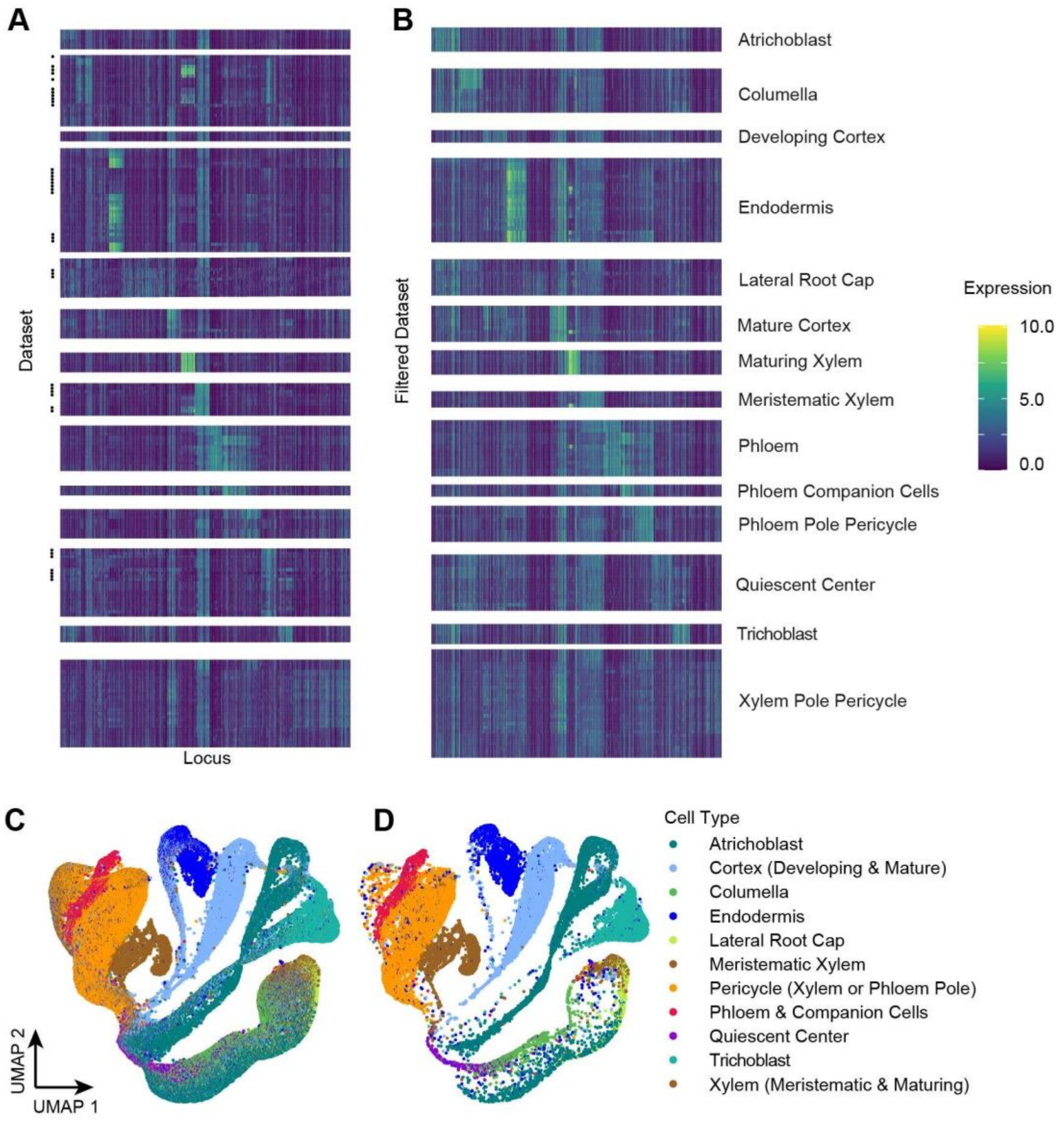
A
B
Dataset
Locus
Filtered Dataset
Atrichoblast
Columella
Developing Cortex
Endodermis
Lateral Root Cap
Mature Cortex
Maturing Xylem
Meristematic Xylem
Phloem
Phloem Companion Cells
Phloem Pole Pericycle
Quiescent Center
Trichoblast
Xylem Pole Pericycle
Expression
10.0
5.0
0.0
C
D
UMAP 2
UMAP 1
Cell Type
Atrichoblast
Cortex (Developing & Mature)
Columella
Endodermis
Lateral Root Cap
Meristematic Xylem
Pericycle (Xylem or Phloem Pole)
Phloem & Companion Cells
Quiescent Center
Trichoblast
Xylem (Meristematic & Maturing)

Figure S3. Filtering datasets for ICI computation. Related to Dataset S3 and STAR Methods. Datasets corresponding to FACS-sorted root protoplasts using a variety of cell-type specific GFP markers (278) were downloaded, normalized together (see STAR Methods), and used to make an ICI specification score table (Efroni et al., 2015). Top markers were identified from this specification score table (corresponding to an information level of 50).

A) Expression levels of identified markers for each cell-type specific dataset. Dots on the left indicate whether that dataset was subsequently filtered out.

B) Expression of newly identified markers after filtering, then re-computing the specification table (using both Affymetrix-based and RNASeq-based datasets together). A pair of specification tables was then generated using either RNASeq-derived data alone, or both RNASeq- and Affymetrix-derived datasets together. ICI scores were then computed using both methods, and the top-scoring cell type is indicated in (**C),** plotted on the Root Cell Atlas UMAP.

D) The same ICI-based cell identities as shown in **C**, but with non-significant (adjusted $P < 0.05$) cell type assignments removed.

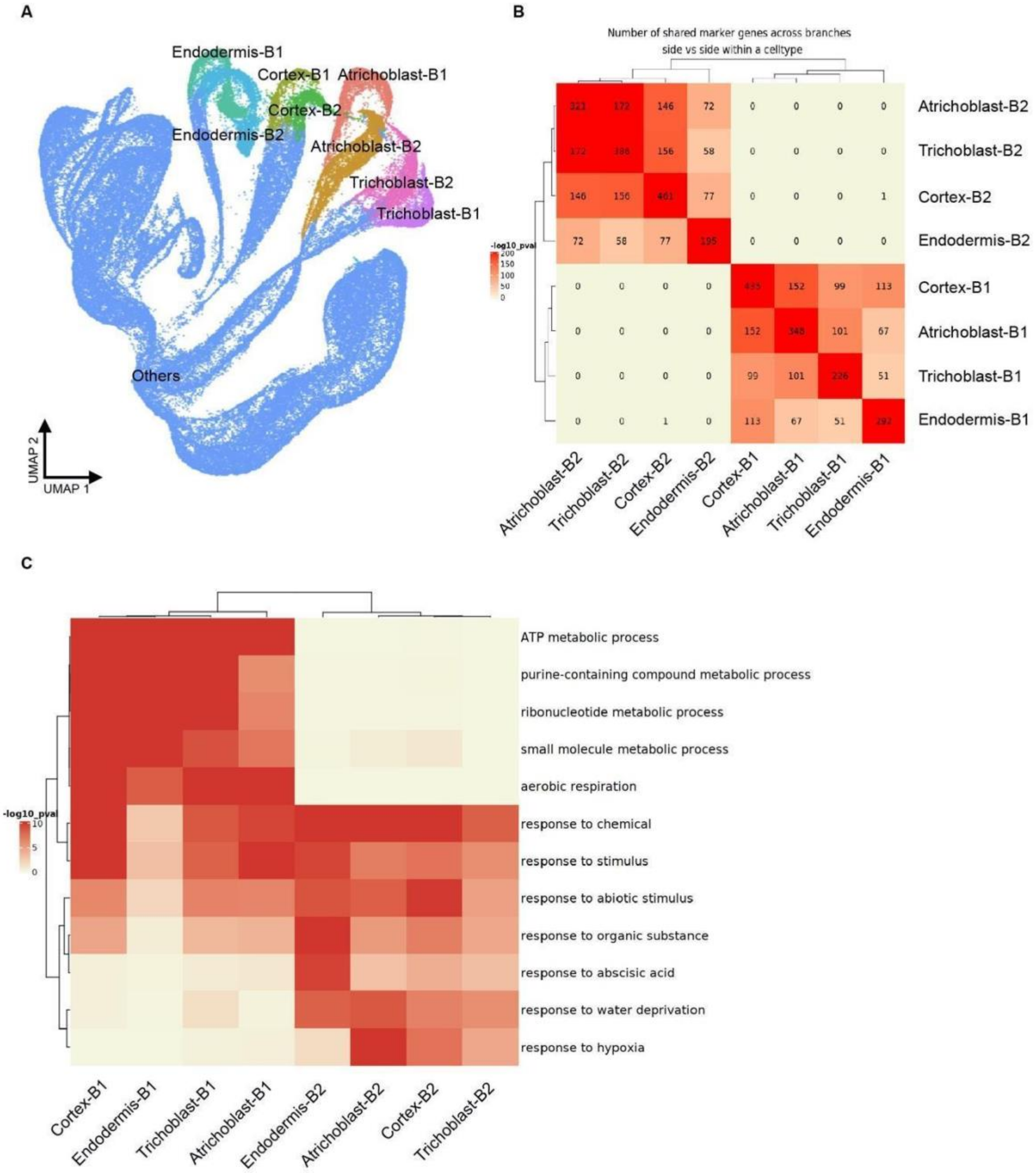

A
Endodermis-B1
Cortex-B1
Atrichoblast-B1
Cortex-B2
Endodermis-B2
Atrichoblast-B2
Trichoblast-B2
Trichoblast-B1
Others
UMAP 2
UMAP 1
B
Number of shared marker genes across branches
side vs side within a celltype
-log10_pval
Atrichoblast-B2
Trichoblast-B2
Cortex-B2
Endodermis-B2
Cortex-B1
Atrichoblast-B1
Trichoblast-B1
Endodermis-B1
C
-log10_pval
ATP metabolic process
purine-containing compound metabolic process
ribonucleotide metabolic process
small molecule metabolic process
aerobic respiration
response to chemical
response to stimulus
response to abiotic stimulus
response to organic substance
response to abscisic acid
response to water deprivation
response to hypoxia
Cortex-B1
Endodermis-B1
Trichoblast-B1
Atrichoblast-B1
Endodermis-B2
Atrichoblast-B2
Cortex-B2
Trichoblast-B2

Figure S4. Distinct Gene Ontology (GO) terms are enriched for cell type sub-branches. Related to Figures 1-2, Dataset S1, and STAR Methods.

A) Within the ground tissue and epidermis, we observed topological bifurcations beginning with elongating cells. However, for each branch on the UMAP, the sub-branches are annotated with the same cell type, developmental stage, and ploidy labels (Figs. 1 and 2). Given our data pre-processing pipeline, which removes known protoplasting-induced genes, they are less likely to reflect technical artifacts such as protoplasting-induced stress. The labels of sub-branches were manually assigned to two distinct clusters that are at the tip of each cell type lineage.

B) A subset of differentially expressed genes is shared across ground tissue and epidermis sub-branches, suggesting that the underlying cause may be a general developmental phenomenon.

C) GO analysis for differentially expressed genes shared among the cell type sub-branches. B1 sub-branches are enriched in metabolic processes and cellular respiration while B2 sub-branches are characterized by 'response' terms, such as response to hypoxia.

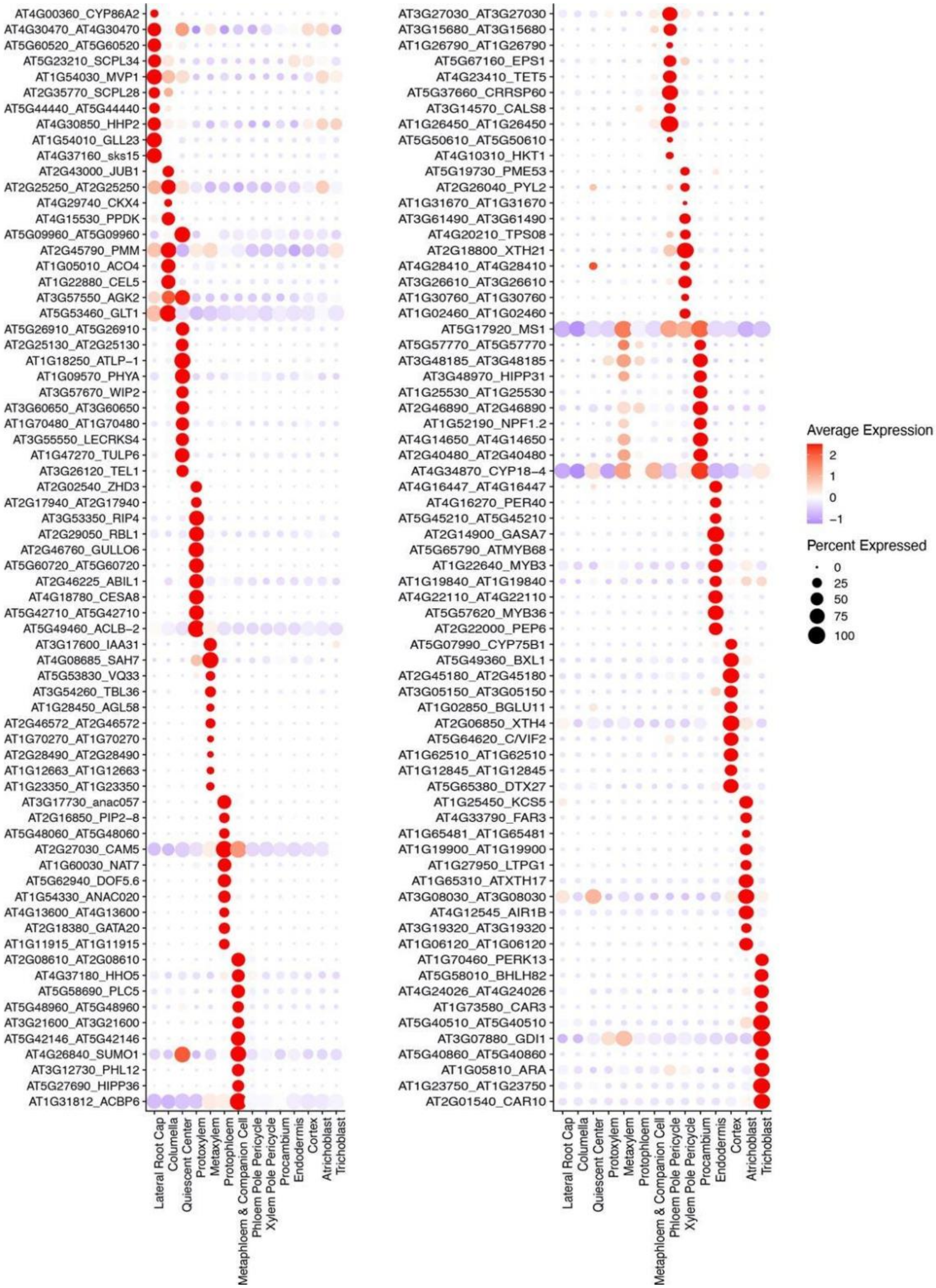
AT4G00360_CYP86A2
AT4G30470_AT4G30470
AT5G60520_AT5G60520
AT5G23210_SCPL34
AT1G54030_MVP1
AT2G35770_SCPL28
AT5G44440_AT5G44440
AT4G30850_HHP2
AT1G54010_GLL23
AT4G37160_sks15
AT2G43000_JUB1
AT2G25250_AT2G25250
AT4G29740_CKX4
AT4G15530_PPDK
AT5G09960_AT5G09960
AT2G45790_PMM
AT1G05010_ACO4
AT1G22880_CEL5
AT3G57550_AGK2
AT5G53460_GLT1
AT5G26910_AT5G26910
AT2G25130_AT2G25130
AT1G18250_ATLP−1
AT1G09570_PHYA
AT3G57670_WIP2
AT3G60650_AT3G60650
AT1G70480_AT1G70480
AT3G55550_LECRKS4
AT1G47270_TULP6
AT3G26120_TEL1
AT2G02540_ZHD3
AT2G17940_AT2G17940
AT3G53350_RIP4
AT2G29050_RBL1
AT2G46760_GULLO6
AT5G60720_AT5G60720
AT2G46225_ABIL1
AT4G18780_CESA8
AT5G42710_AT5G42710
AT5G49460_ACLB−2
AT3G17600_IAA31
AT4G08685_SAH7
AT5G53830_VQ33
AT3G54260_TBL36
AT1G28450_AGL58
AT2G46572_AT2G46572
AT1G70270_AT1G70270
AT2G28490_AT2G28490
AT1G12663_AT1G12663
AT1G23350_AT1G23350
AT3G17730_anac057
AT2G16850_PIP2−8
AT5G48060_AT5G48060
AT2G27030_CAM5
AT1G60030_NAT7
AT5G62940_DOF5.6
AT1G54330_ANAC020
AT4G13600_AT4G13600
AT2G18380_GATA20
AT1G11915_AT1G11915
AT2G08610_AT2G08610
AT4G37180_HHO5
AT5G58690_PLC5
AT5G48960_AT5G48960
AT3G21600_AT3G21600
AT5G42146_AT5G42146
AT4G26840_SUMO1
AT3G12730_PHL12
AT5G27690_HIPP36
AT1G31812_ACBP6
Lateral Root Cap
Columella
Quiescent Center
Protoxylem
Metaxylem
Protophloem
Metaphloem & Companion Cell
Phloem Pole Pericycle
Xylem Pole Pericycle
Procambium
Endodermis
Cortex
Atrichoblast
Trichoblast
AT3G27030_AT3G27030
AT3G15680_AT3G15680
AT1G26790_AT1G26790
AT5G67160_EPS1
AT4G23410_TET5
AT5G37660_CRRSP60
AT3G14570_CALS8
AT1G26450_AT1G26450
AT5G50610_AT5G50610
AT4G10310_HKT1
AT5G19730_PME53
AT2G26040_PYL2
AT1G31670_AT1G31670
AT3G61490_AT3G61490
AT4G20210_TPS08
AT2G18800_XTH21
AT4G28410_AT4G28410
AT3G26610_AT3G26610
AT1G30760_AT1G30760
AT1G02460_AT1G02460
AT5G17920_MS1
AT5G57770_AT5G57770
AT3G48185_AT3G48185
AT3G48970_HIPP31
AT1G25530_AT1G25530
AT2G46890_AT2G46890
AT1G52190_NPF1.2
AT4G14650_AT4G14650
AT2G40480_AT2G40480
AT4G34870_CYP18−4
AT4G16447_AT4G16447
AT4G16270_PER40
AT5G45210_AT5G45210
AT2G14900_GASA7
AT5G65790_ATMYB68
AT1G22640_MYB3
AT1G19840_AT1G19840
AT4G22110_AT4G22110
AT5G57620_MYB36
AT2G22000_PEP6
AT5G07990_CYP75B1
AT5G49360_BXL1
AT2G45180_AT2G45180
AT3G05150_AT3G05150
AT1G02850_BGLU11
AT2G06850_XTH4
AT5G64620_C/VIF2
AT1G62510_AT1G62510
AT1G12845_AT1G12845
AT5G65380_DTX27
AT1G25450_KCS5
AT4G33790_FAR3
AT1G65481_AT1G65481
AT1G19900_AT1G19900
AT1G27950_LTPG1
AT1G65310_ATXTH17
AT3G08030_AT3G08030
AT4G12545_AIR1B
AT3G19320_AT3G19320
AT1G06120_AT1G06120
AT1G70460_PERK13
AT5G58010_BHLH82
AT4G24026_AT4G24026
AT1G73580_CAR3
AT5G40510_AT5G40510
AT3G07880_GDI1
AT5G40860_AT5G40860
AT1G05810_ARA
AT1G23750_AT1G23750
AT2G01540_CAR10
Lateral Root Cap
Columella
Quiescent Center
Protoxylem
Metaxylem
Protophloem
Metaphloem & Companion Cell
Phloem Pole Pericycle
Xylem Pole Pericycle
Procambium
Endodermis
Cortex
Atrichoblast
Trichoblast
Average Expression
2
1
0
−1
Percent Expressed
0
25
50
75
100

Figure S5. Differential expression analyses identify cell type-specific marker genes. Related to Figure 1, Dataset S1, and STAR Methods. Dot plot of the top ten marker genes, ordered by log fold change, identified for each of the fourteen cell types in the atlas. Circle size represents the percentage of cells in which a gene is expressed (percent expressed). Color represents the average expression level of each gene in each cell type (average expression).

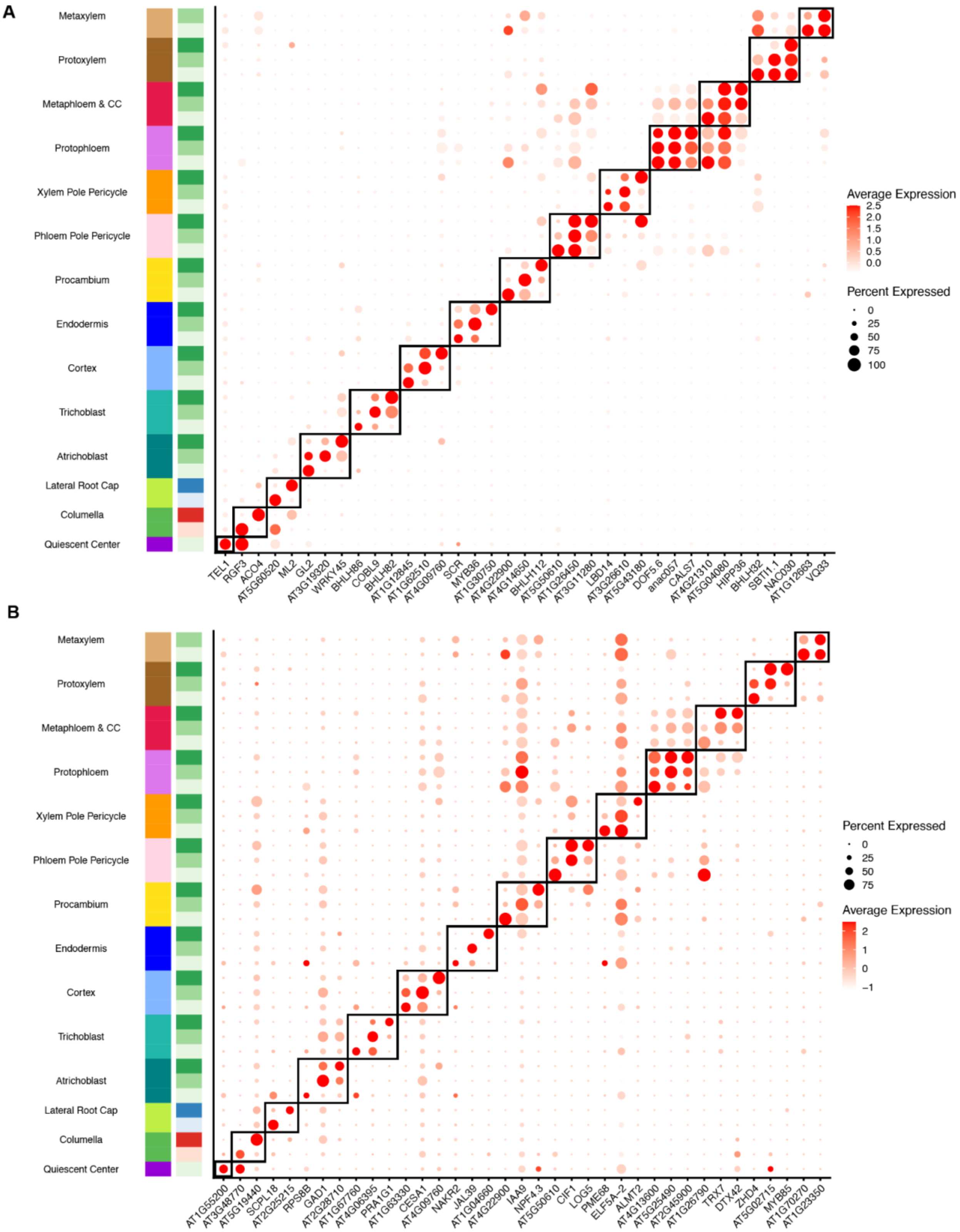
A
Metaxylem
Protoxylem
Metaphloem & CC
Protophloem
Xylem Pole Pericycle
Phloem Pole Pericycle
Procambium
Endodermis
Cortex
Trichoblast
Atrichoblast
Lateral Root Cap
Columella
Quiescent Center
TEL1
RGF3
ACO4
AT5G60520
ML2
GL2
AT3G19320
WRKY45
BHLH86
COBL9
BHLH82
AT1G12845
AT1G62510
AT4G09760
SCR
MYB36
AT1G30750
AT4G22900
AT4G14650
BHLH112
AT5G50610
AT1G26450
AT3G11280
LBD14
AT3G26610
AT5G43180
DOF5.6
anac057
CALS7
AT4G21310
AT5G04080
HIPP36
BHLH32
SBTI1.1
NAC030
AT1G12663
VQ33
Average Expression
2.5
2.0
1.5
1.0
0.5
0.0
Percent Expressed
0
25
50
75
100
B
Metaxylem
Protoxylem
Metaphloem & CC
Protophloem
Xylem Pole Pericycle
Phloem Pole Pericycle
Procambium
Endodermis
Cortex
Trichoblast
Atrichoblast
Lateral Root Cap
Columella
Quiescent Center
AT1G55200
AT3G48770
AT5G19440
SCPL18
AT2G25215
RPS8B
GAD1
AT2G28710
AT1G67760
AT4G06395
PRA1G1
AT1G63330
CESA1
AT4G09760
NAKR2
JAL39
AT1G04660
AT4G22900
IAA9
NPF4.3
AT5G50610
CIF1
LOG5
PME68
ELF5A-2
ALMT2
AT4G13600
AT5G25490
AT2G45900
AT1G26790
TRX7
DTX42
ZHD4
AT5G02715
MYB85
AT1G70270
AT1G23350
Percent Expressed
0
25
50
75
Average Expression
2
1
0
−1

Figure S6. Marker genes specific to both a cell type and a developmental stage are rare, suggesting that gradual transcriptional changes underlie development of each cell and tissue type. Related to Figures 1-2, Dataset S1, and STAR Methods.
Dot plots of one marker gene for each cell type and developmental stage combination. Circle size represents the percentage of cells in which a gene is expressed (percent expressed). Circle color represents the average expression level of each gene in each cell type (average expression). Black boxes denote markers from each cell type. Colors of side annotations indicate cell type and developmental stage (same color scheme as Figs. 1C and 2A). Among the markers that are identified by both cluster-dependent and cluster-agnostic methods, only a few markers for mature endodermis (AT1G04660) and distal lateral root cap (AT2G32620) can be considered strictly specific. As a general trend, maturation zone and distal root cap markers are found when gradually lowering the threshold for specificity of markers, which suggests that the specificity of gene expression increases along with maturation of cells.

A) Markers identified by Seurat's Wilcoxon test. Known markers were plotted if they were identified as differentially expressed in a specific cell type + developmental stage group with an adjusted p-value below 0.05. In cases where a known marker gene was not identified, the top differentially expressed gene, according to average log fold-change, was plotted.
B) Markers identified by SEMITONES. Only the marker with the highest enrichment score is plotted for each cell type + developmental stage.

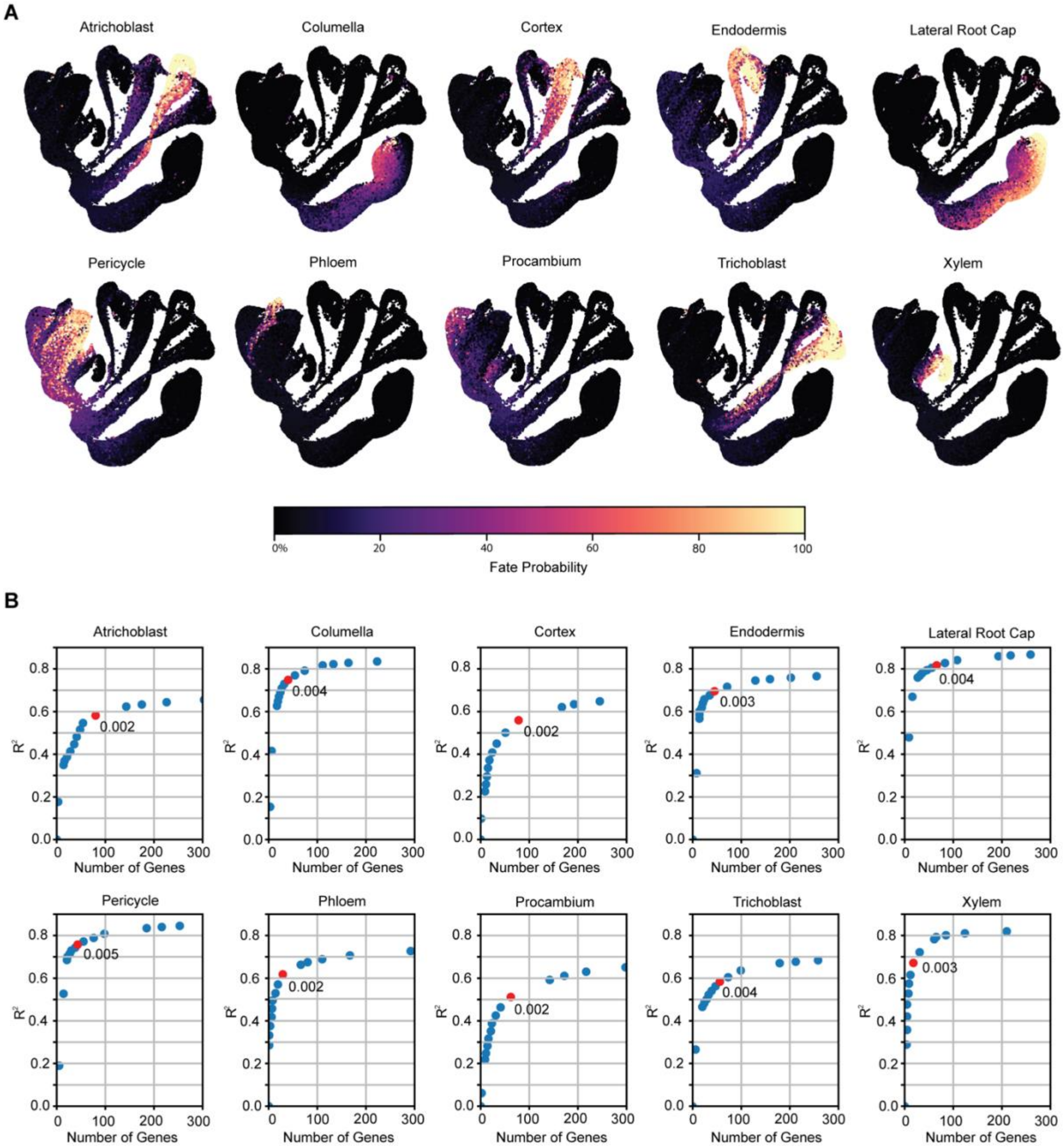
A
Atrichoblast
Columella
Cortex
Endodermis
Lateral Root Cap
Pericycle
Phloem
Procambium
Trichoblast
Xylem
0%
20
40
60
80
100
Fate Probability
B
Atrichoblast
Columella
Cortex
Endodermis
Lateral Root Cap
Pericycle
Phloem
Procambium
Trichoblast
Xylem
R^2
Number of Genes
0.002
0.004
0.002
0.003
0.004
0.005
0.002
0.002
0.004
0.003

Figure S7. Details related to StationaryOT cell fate probability calculations and regressions. Related to Figure 5, Dataset S5, and STAR Methods.

A) The maximum fate probability for each cell in the atlas, as calculated by StationaryOT, agrees with atlas cell lineage annotations. StationaryOT fate probabilities for each of ten cell types are shown. Color scale indicates percent probability of each cell type fate. Compare to the atlas cell type annotation shown in Fig. 1C.

B) Plots of R^2 values versus the number of genes with non-zero coefficients for Lasso regressions across a range of alpha values. Regressions were performed on cells in the meristem against fate probabilities for the displayed cell types. The knee points of the graphs are highlighted in red and are displayed next to their corresponding values of alpha. Note that knee points may vary between trials when random sampling is used to determine training sets for Lasso.

Data S1. COPILOT output for each scRNA-seq sample profiled in this study. Related to Figs. 1-2 and 6-7, Fig. S1, and STAR Methods.

col0 Summary

Processed by COPILOT

Summary Analysis

Parameters

Iteration of Filtering	1
Mitochondrial Expression Threshold	5 %
Top High Quality Cell Filtered	1 %
Doublet Removed	Yes

Cell Stats

Estimated Number of High Quality Cell	6,779
High Quality Cell	9.28 %
Total UMI Counts in High Quality Cell	136,642,155
UMI Counts in High Quality Cell	66.94 %
Median UMI Counts per High Quality Cell	11,648
Median Genes per High Quality Cell	2,994
Total Genes Detected in High Quality Cell	24,854
Cell above Mitochondrial Expression Threshold	8.13 %
Estimated Doublet Rate in High Quality Cell	5.11 %

Sequencing Stats

Number of Reads Processed	385,741,789
Reads Pseudoaligned	88.9 %
Reads on Whitelist	94.66 %
Total UMI Counts	204,120,352
Sequencing Technology	10xv3
Species	Arabidopsis thaliana
Transcriptome	TAIR10

Sample Stats

Sample	col0
Name	WT Col-0
Source	Benfey lab
Genotype	WT Col-0
Transgene	NA
Treatment	untreated
Age	5_day
Timepoint	NA
Rep	NA
Target Cells	5,000
Date	NA
Seq Run	NA

UMI Counts Histogram

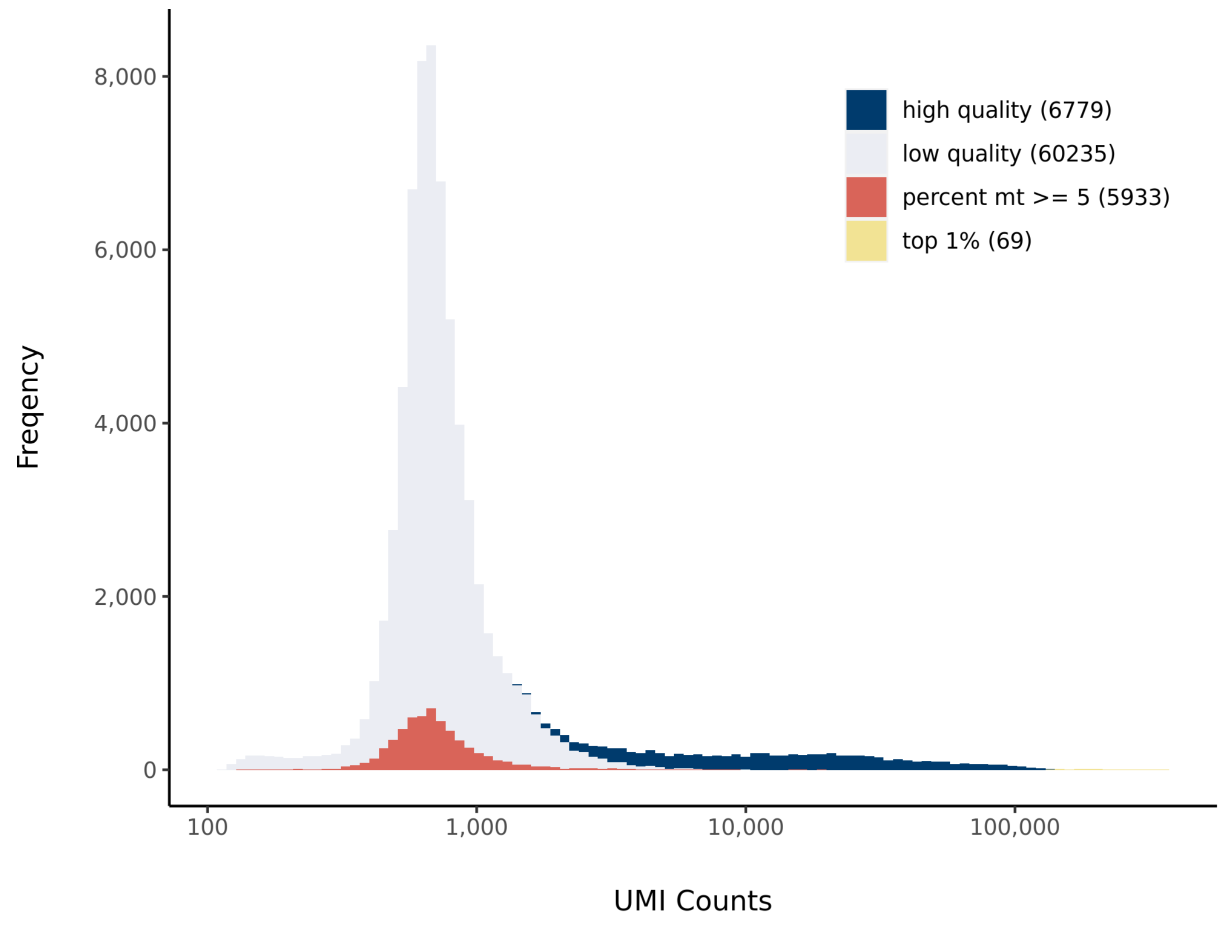

Number of Genes Histogram

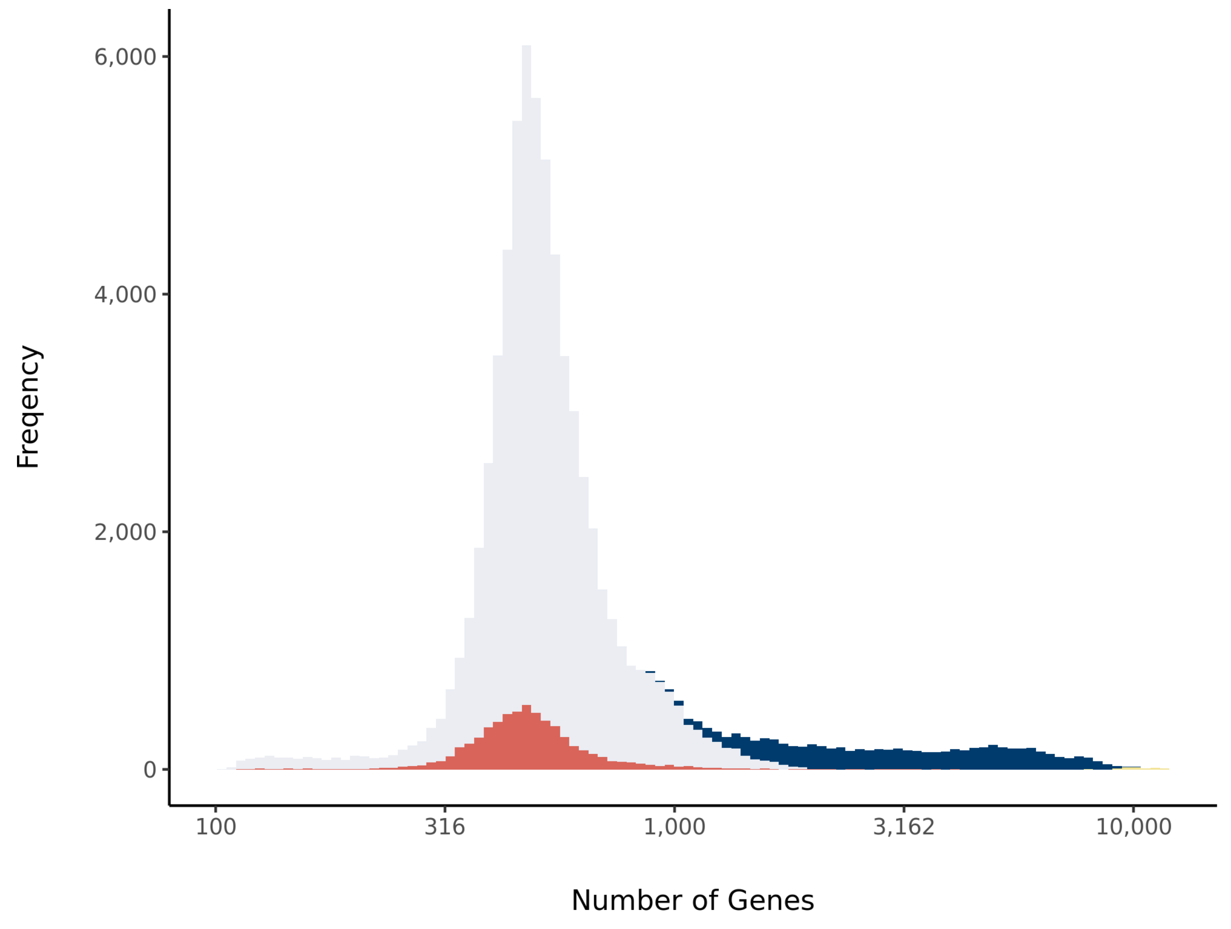

Barcode Rank Plot

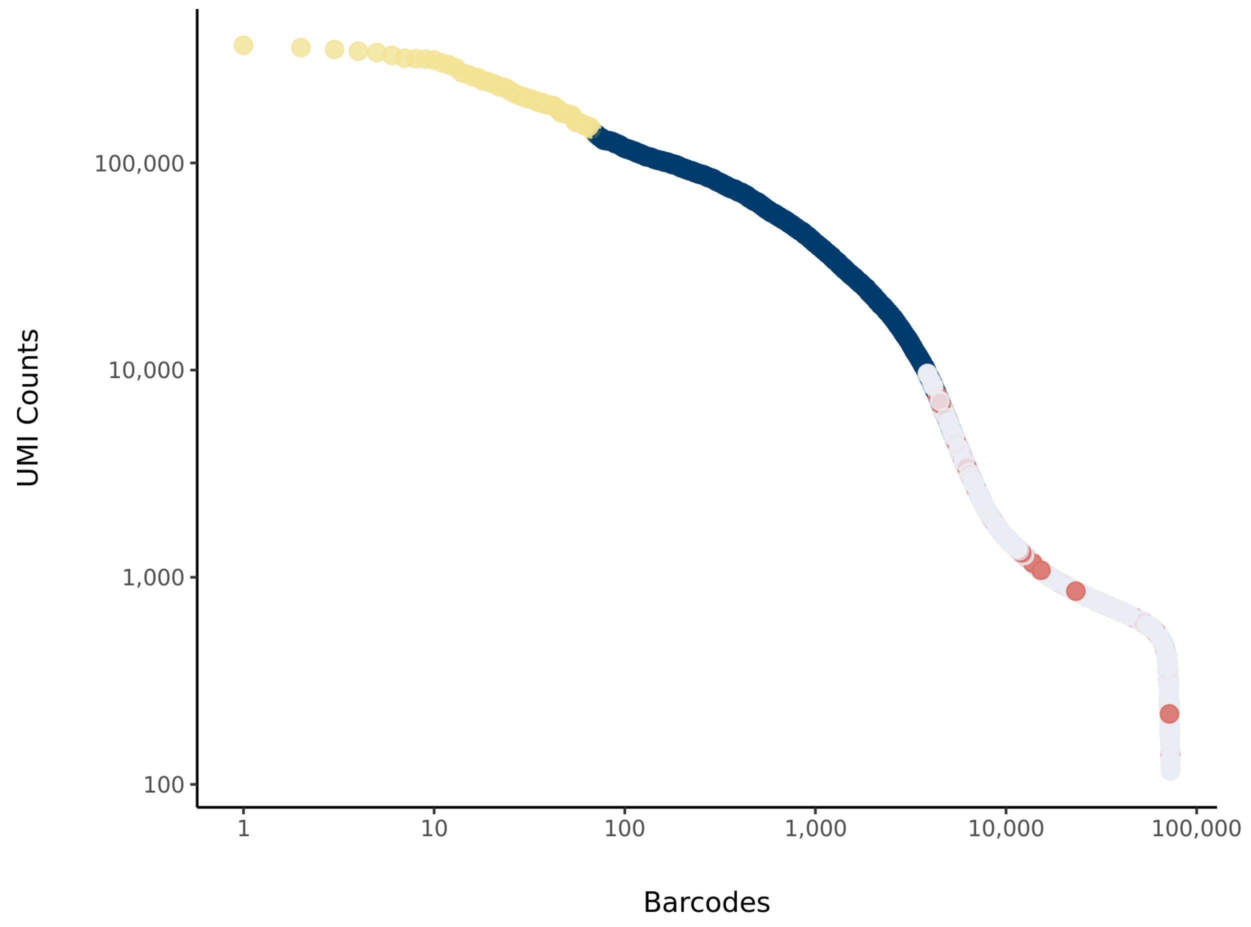

dc1 Summary

Processed by COPILOT

Summary Analysis

Parameters

Iteration of Filtering	1
Mitochondrial Expression Threshold	5 %
Top High Quality Cell Filtered	1 %
Doublet Removed	Yes

Cell Stats

Estimated Number of High Quality Cell	3,471
High Quality Cell	4.65 %
Total UMI Counts in High Quality Cell	68,731,526
UMI Counts in High Quality Cell	48.3 %
Median UMI Counts per High Quality Cell	10,673
Median Genes per High Quality Cell	3,482
Total Genes Detected in High Quality Cell	25,036
Cell above Mitochondrial Expression Threshold	0.07 %
Estimated Doublet Rate in High Quality Cell	2.68 %

Sequencing Stats

Number of Reads Processed	261,013,034
Reads Pseudoaligned	90.1 %
Reads on Whitelist	95.61 %
Total UMI Counts	142,313,355
Sequencing Technology	10xv2
Species	Arabidopsis thaliana
Transcriptome	TAIR10

Sample Stats

Sample	dc1
Name	WT Developmental Cell 1
Source	Denyer et al. 2019, Developmental Cell
Genotype	WT Col-0
Transgene	NA
Treatment	Untreated
Age	6_day
Timepoint	NA
Rep	1
Target Cells	NA
Date	NA
Seq Run	NA

UMI Counts Histogram

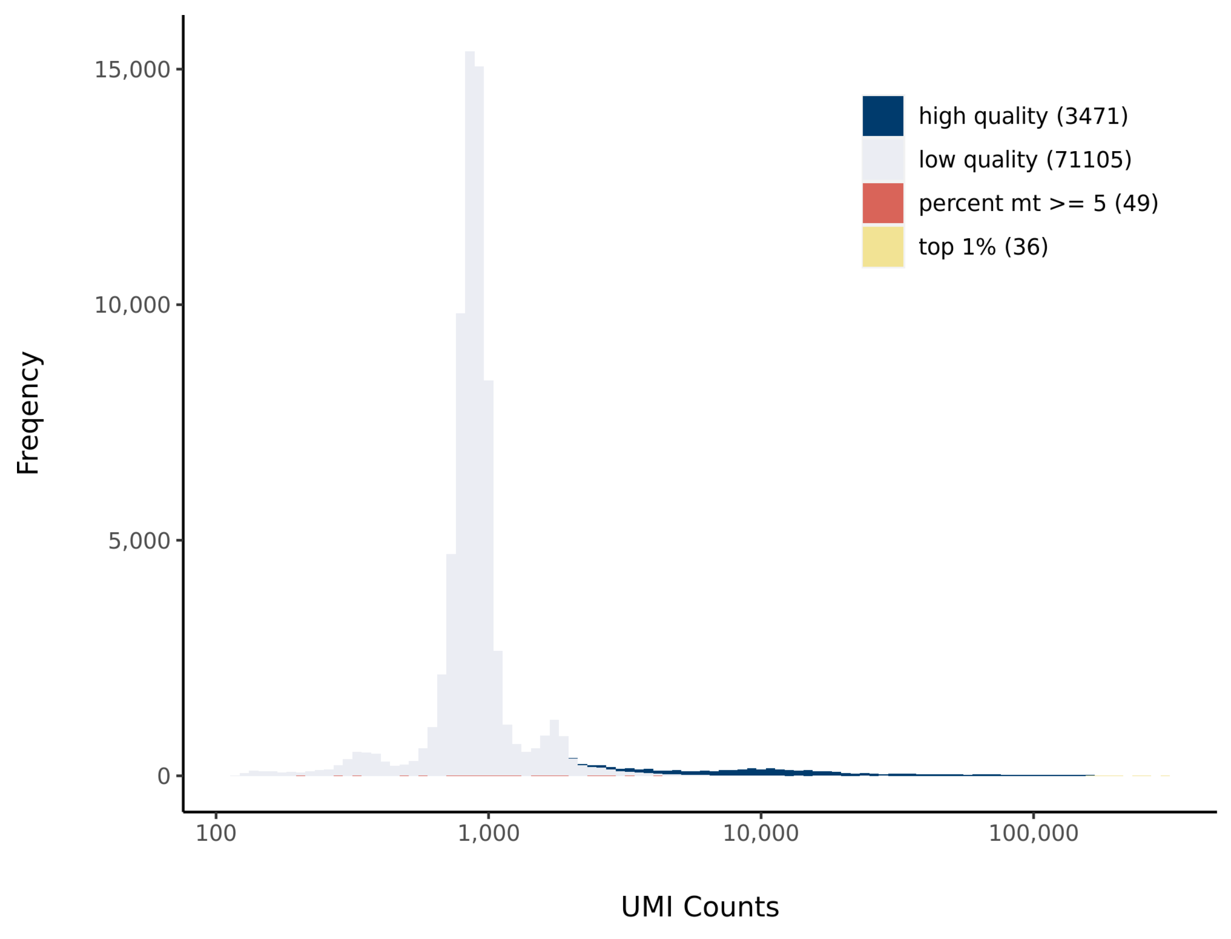

Number of Genes Histogram

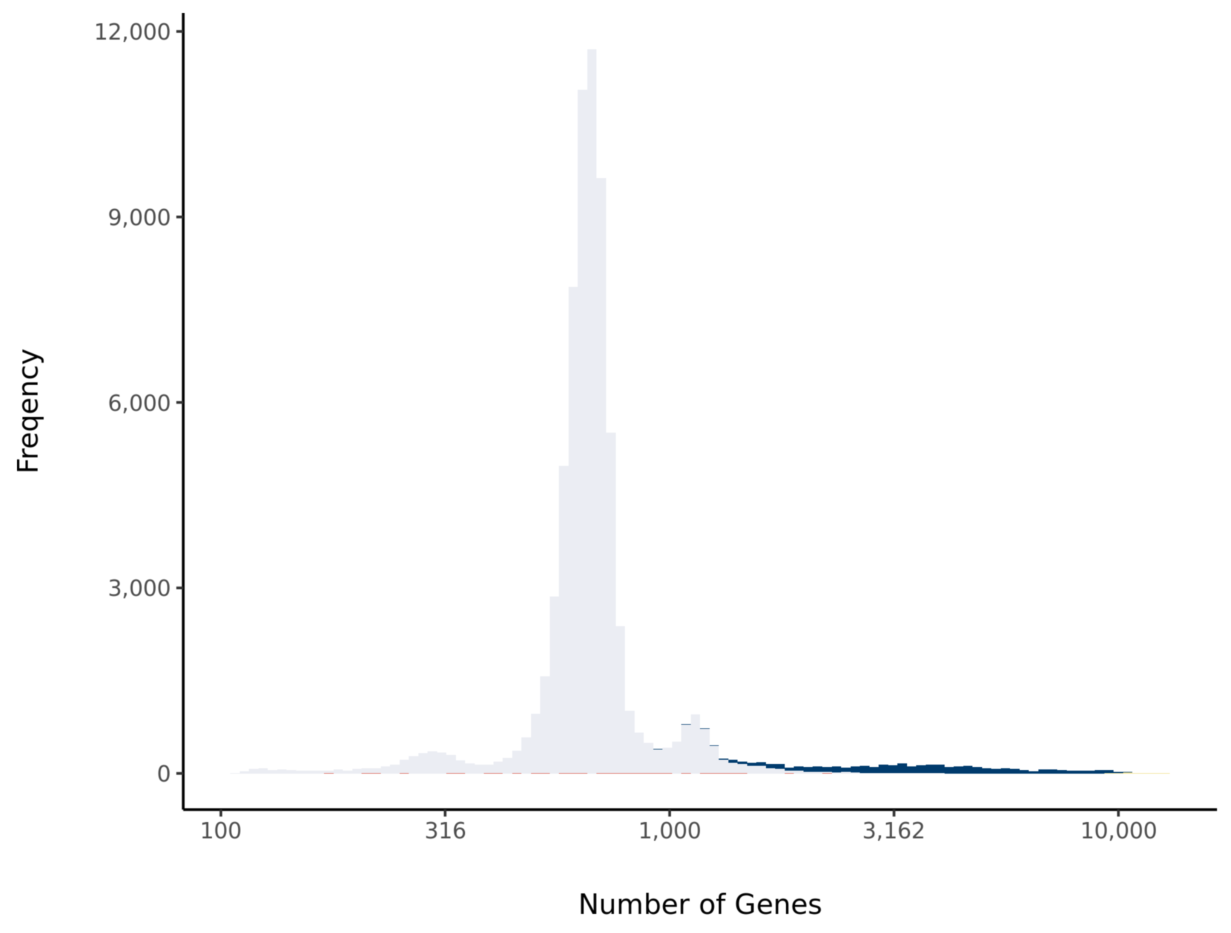

Barcode Rank Plot

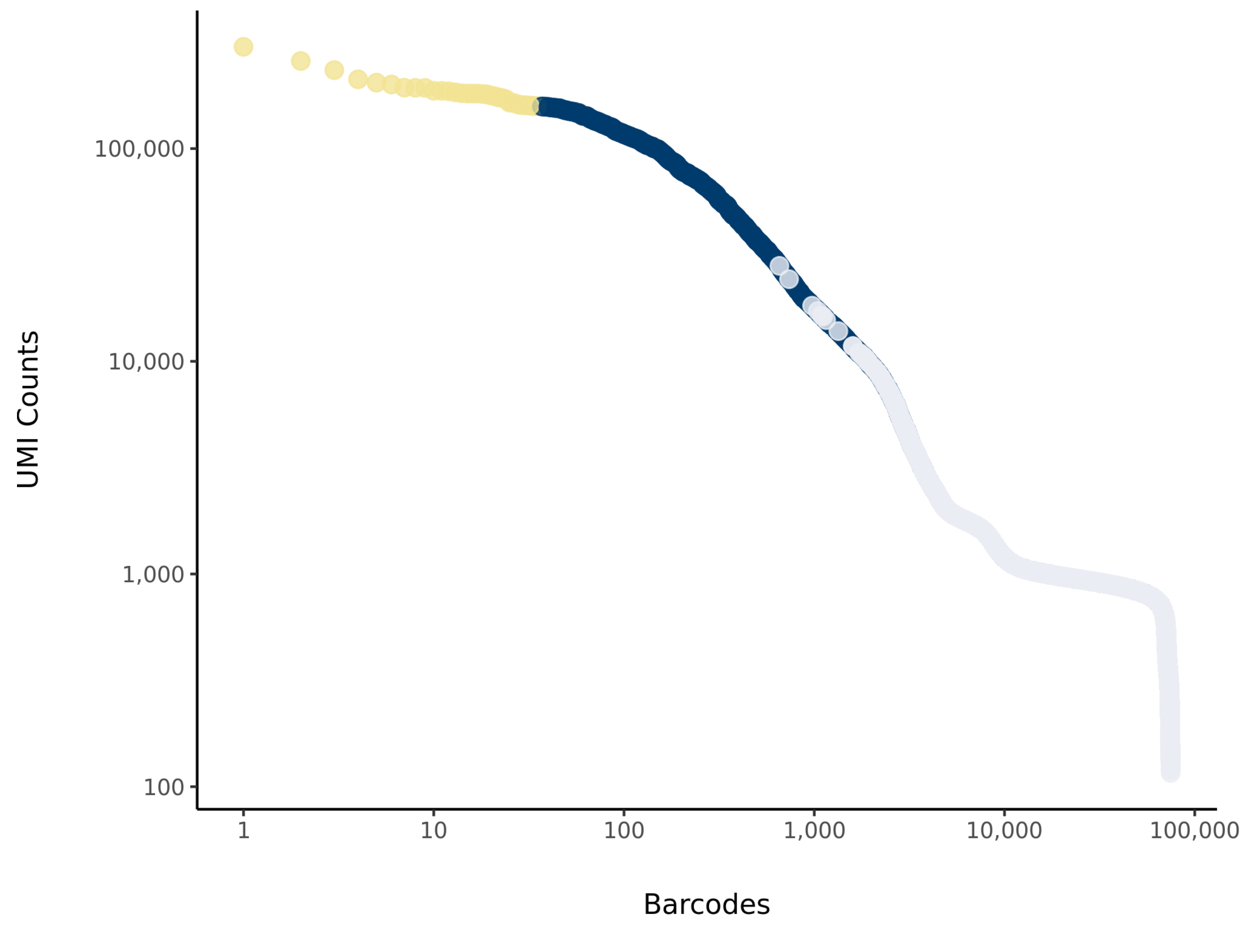

dc2 Summary

Processed by COPILOT

Summary Analysis

Parameters

Iteration of Filtering	1
Mitochondrial Expression Threshold	5 %
Top High Quality Cell Filtered	1 %
Doublet Removed	Yes

Cell Stats

Estimated Number of High Quality Cell	3,371
High Quality Cell	4.56 %
Total UMI Counts in High Quality Cell	68,781,391
UMI Counts in High Quality Cell	63.21 %
Median UMI Counts per High Quality Cell	11,439
Median Genes per High Quality Cell	3,508
Total Genes Detected in High Quality Cell	25,100
Cell above Mitochondrial Expression Threshold	0.15 %
Estimated Doublet Rate in High Quality Cell	2.61 %

Sequencing Stats

Number of Reads Processed	235,863,595
Reads Pseudoaligned	89.6 %
Reads on Whitelist	95.56 %
Total UMI Counts	108,819,006
Sequencing Technology	10xv2
Species	Arabidopsis thaliana
Transcriptome	TAIR10

Sample Stats

Sample	dc2
Name	WT Developmental Cell 2
Source	Denyer et al. 2019, Developmental Cell
Genotype	WT Col-0
Transgene	NA
Treatment	Untreated
Age	6_day
Timepoint	NA
Rep	2
Target Cells	NA
Date	NA
Seq Run	NA

UMI Counts Histogram

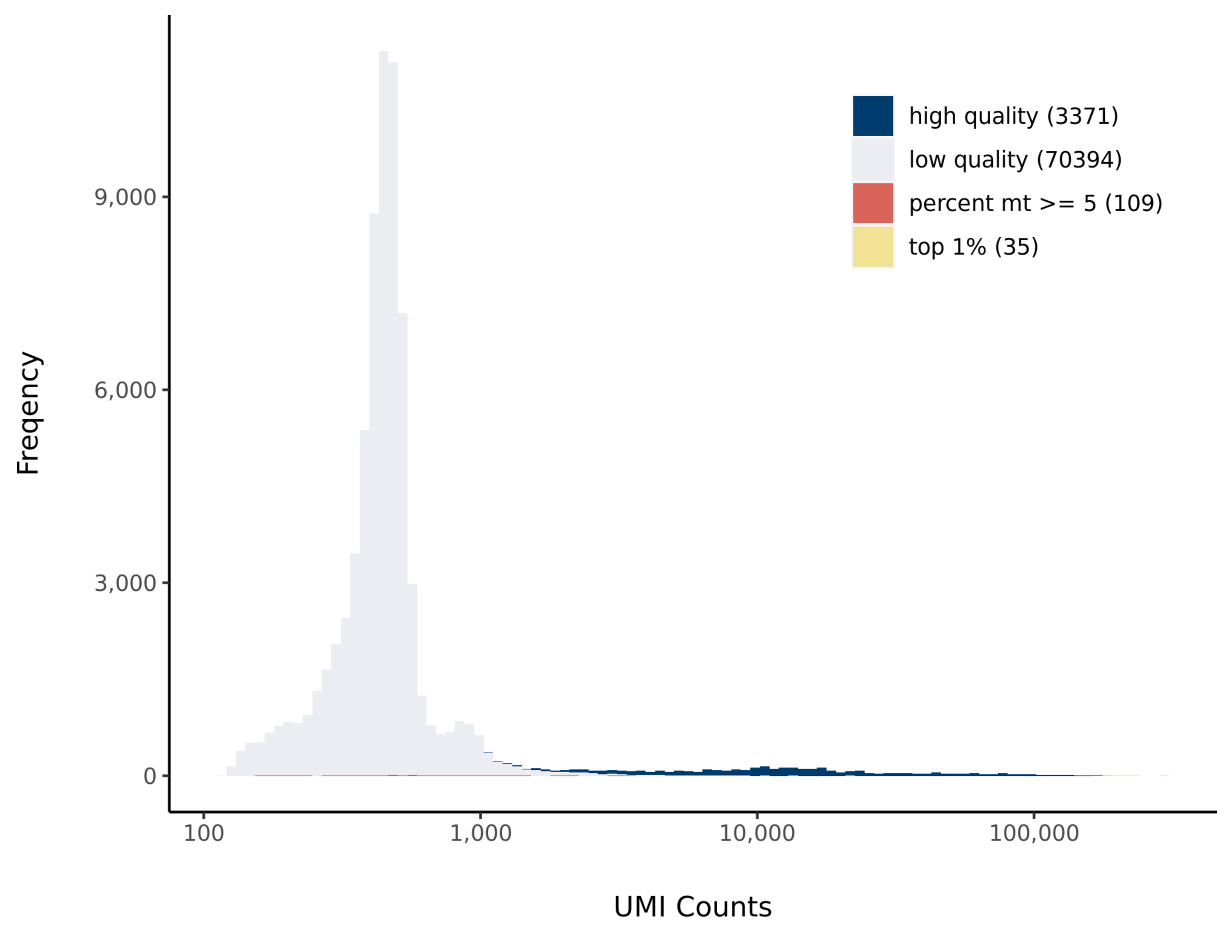

Number of Genes Histogram

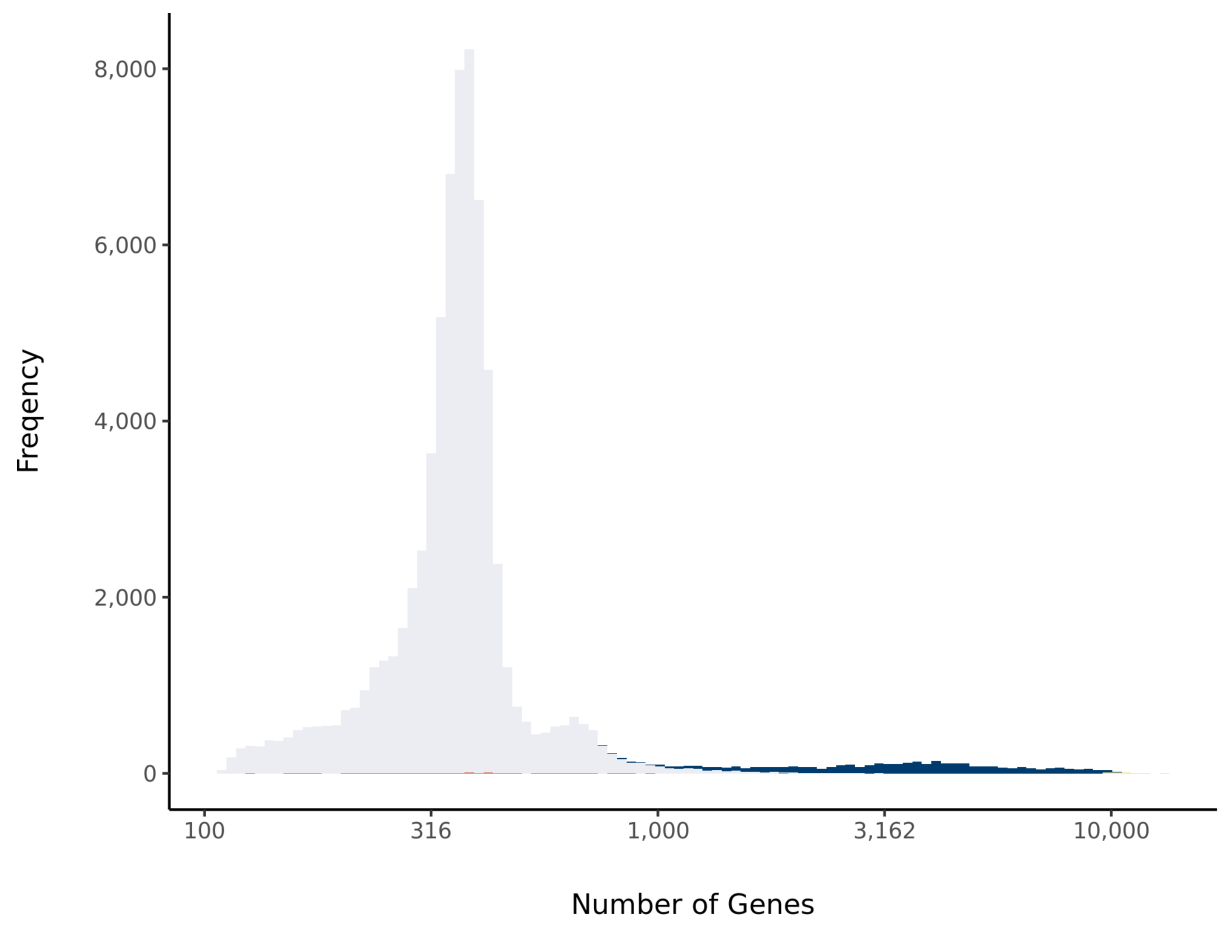

Barcode Rank Plot

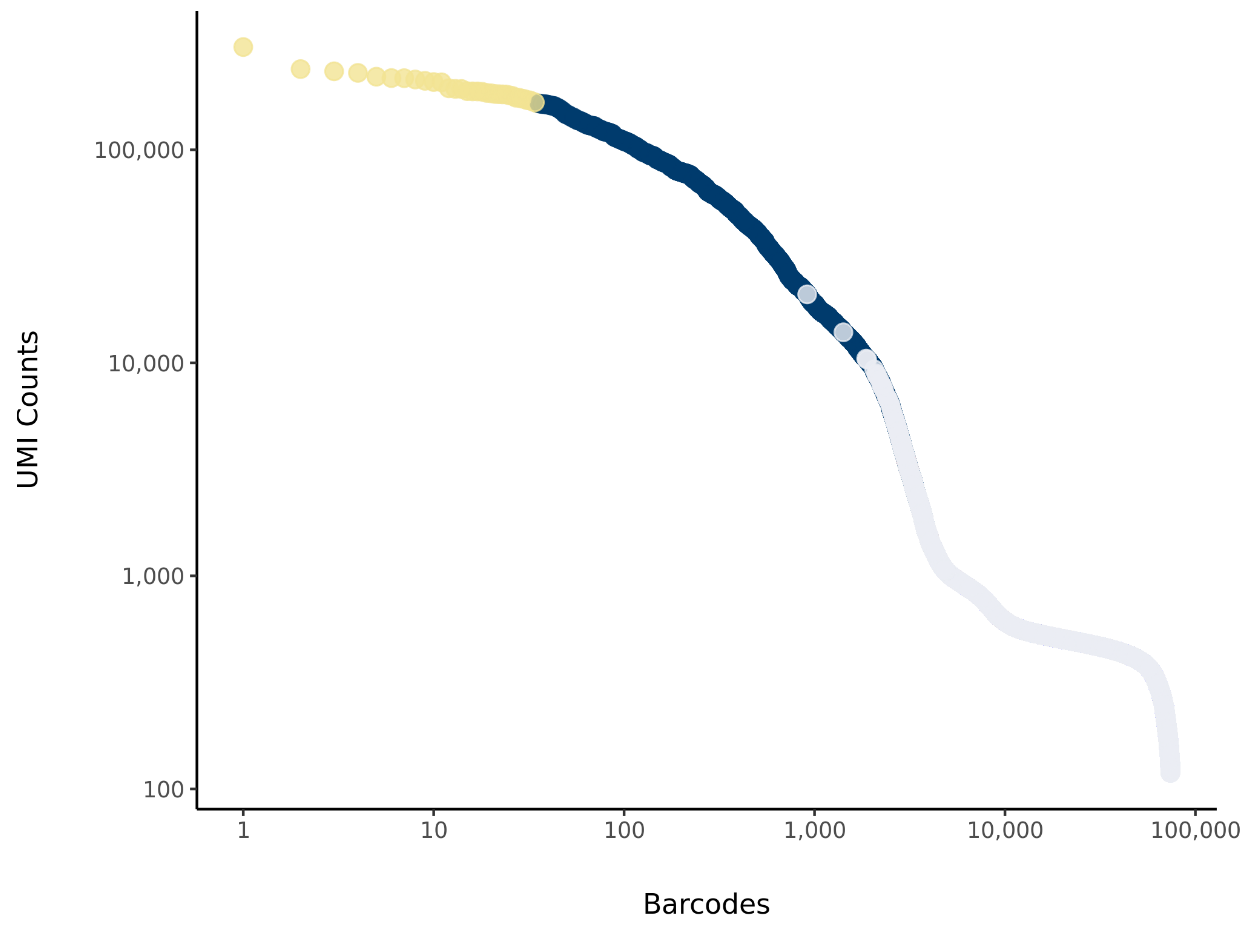

pp1 Summary

Processed by COPILOT

Summary Analysis

Parameters

Iteration of Filtering	1
Mitochondrial Expression Threshold	5 %
Top High Quality Cell Filtered	1 %
Doublet Removed	Yes

Cell Stats

Estimated Number of High Quality Cell	8,168
High Quality Cell	11.82 %
Total UMI Counts in High Quality Cell	165,283,937
UMI Counts in High Quality Cell	72.54 %
Median UMI Counts per High Quality Cell	13,299.5
Median Genes per High Quality Cell	3,777.5
Total Genes Detected in High Quality Cell	25,625
Cell above Mitochondrial Expression Threshold	0.05 %
Estimated Doublet Rate in High Quality Cell	6.13 %

Sequencing Stats

Number of Reads Processed	377,721,321
Reads Pseudoaligned	93.6 %
Reads on Whitelist	96.53 %
Total UMI Counts	227,847,045
Sequencing Technology	10xv2
Species	Arabidopsis thaliana
Transcriptome	TAIR10

Sample Stats

Sample	pp1
Name	WT Plant Physiology 1
Source	Ryu et al. 2019, Plant Physiology
Genotype	WT Col-0
Transgene	NA
Treatment	Untreated
Age	5_day
Timepoint	NA
Rep	1
Target Cells	NA
Date	NA
Seq Run	NA

UMI Counts Histogram

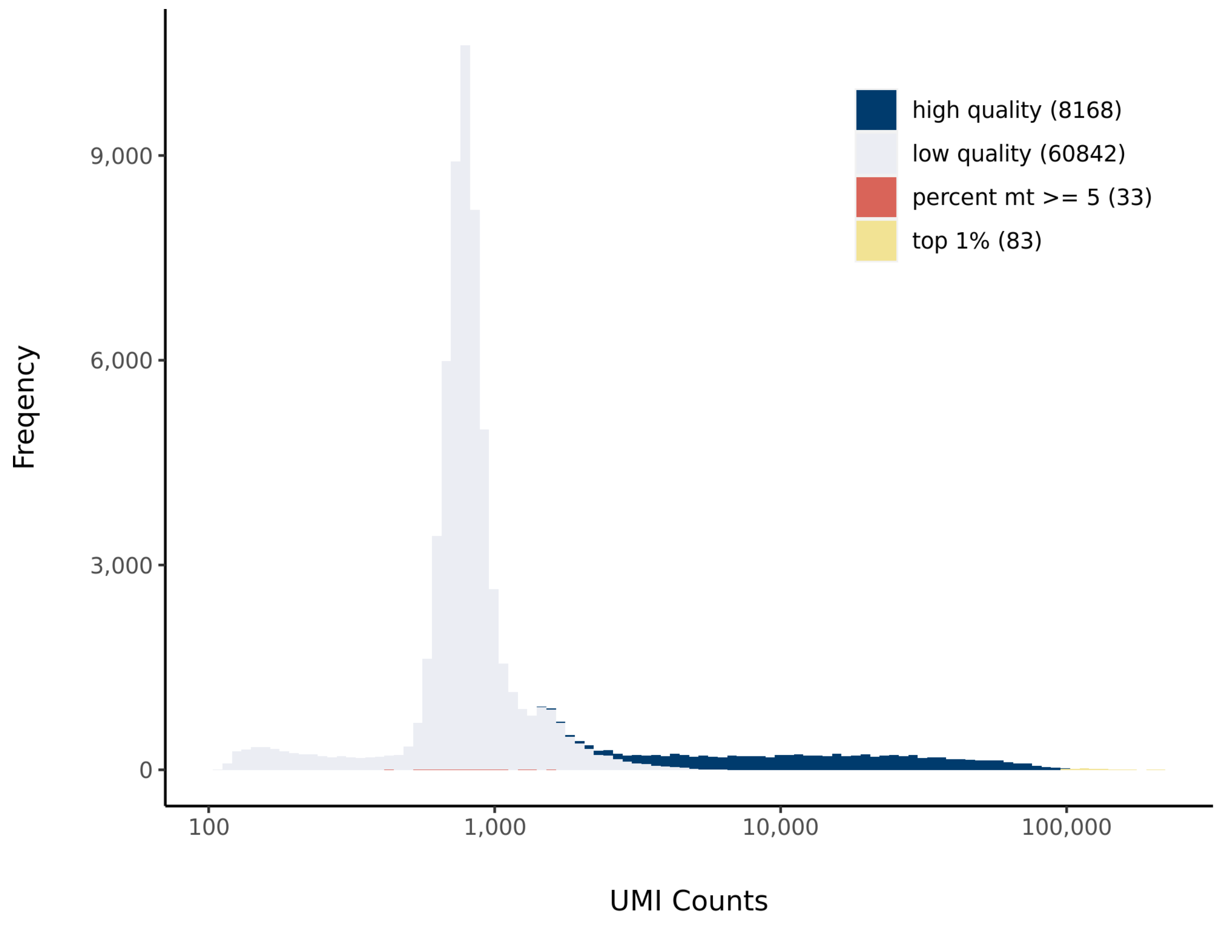

Number of Genes Histogram

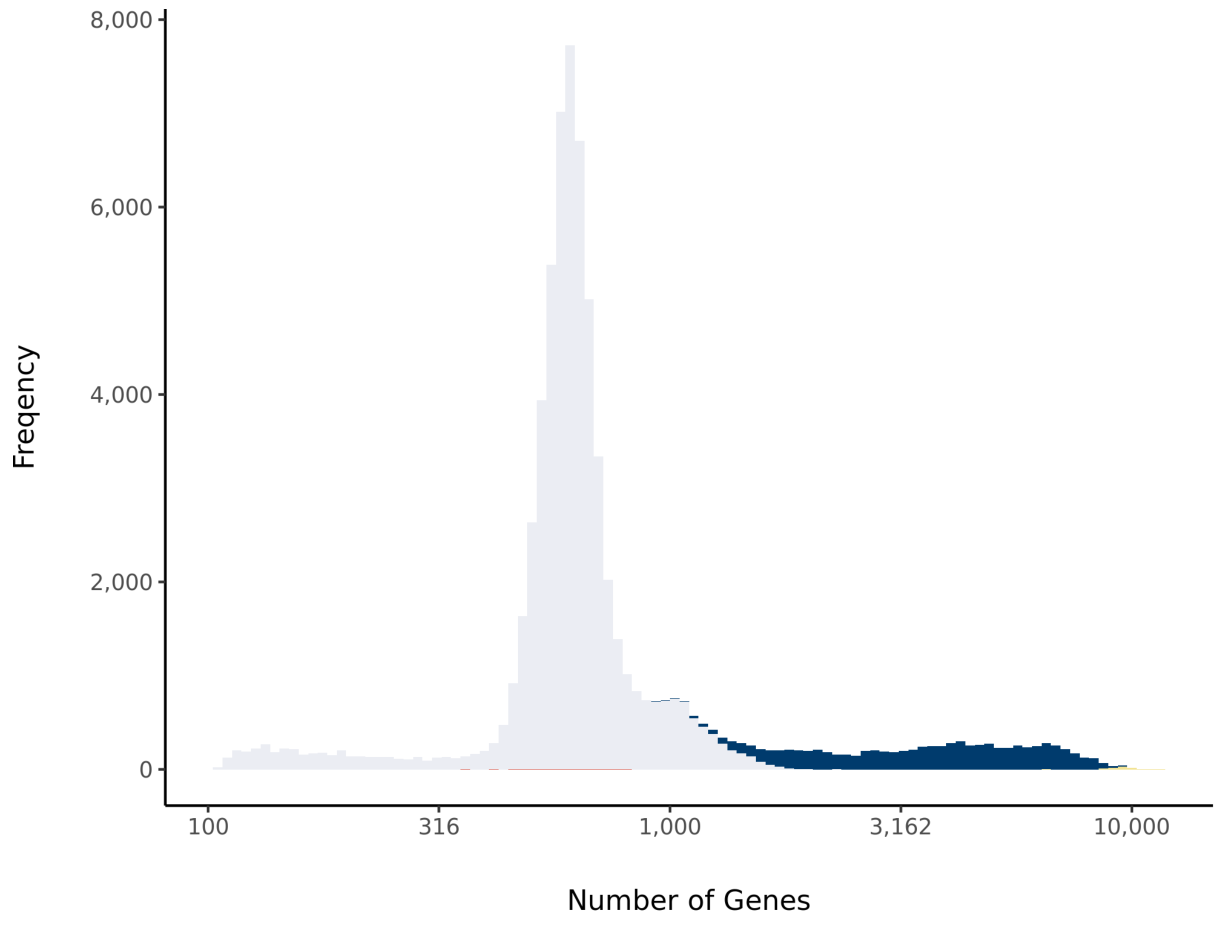

Barcode Rank Plot

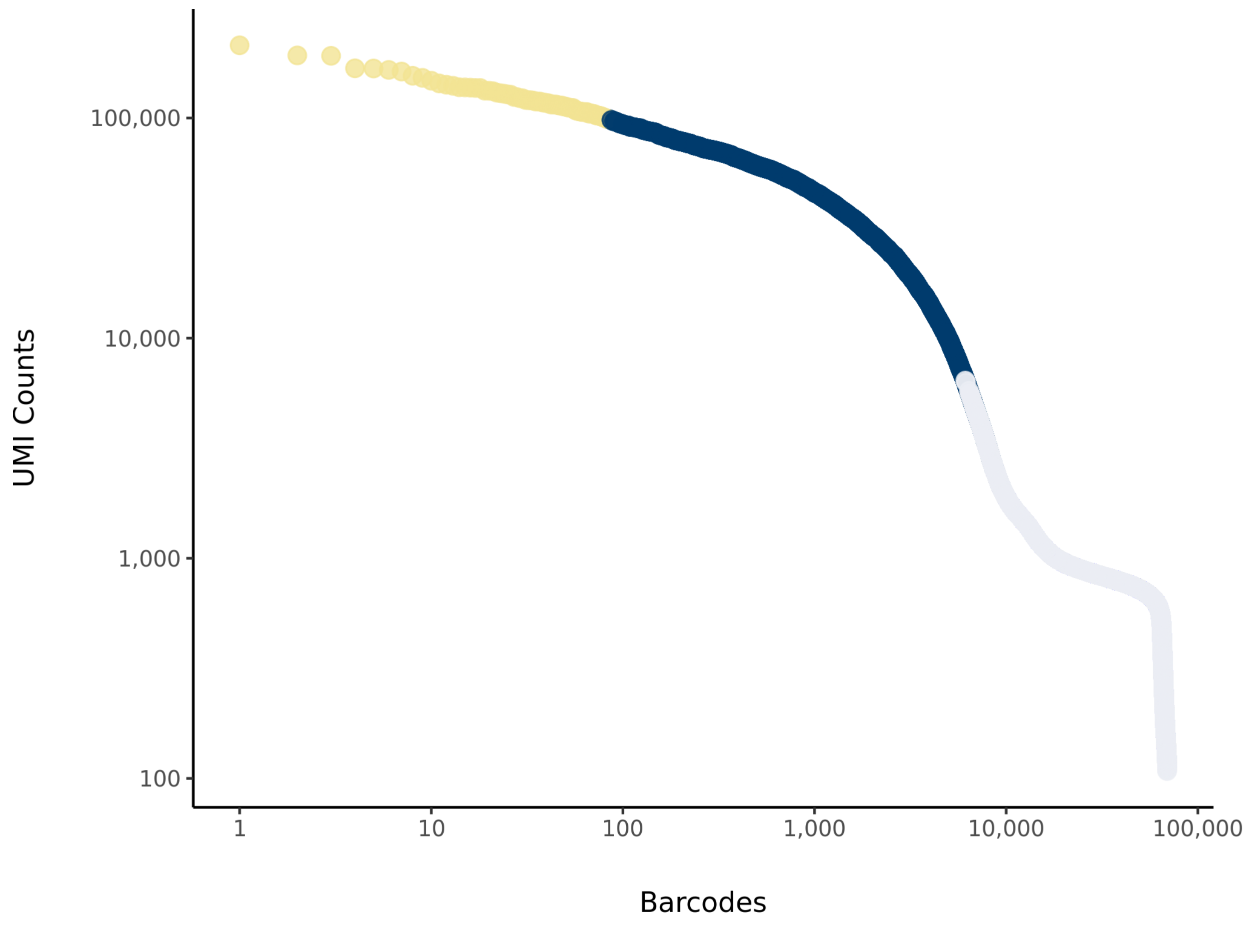

sc_1 Summary

Processed by COPILOT

Summary Analysis

Parameters

Iteration of Filtering	1
Mitochondrial Expression Threshold	5 %
Top High Quality Cell Filtered	1 %
Doublet Removed	Yes

Cell Stats

Estimated Number of High Quality Cell	10,598
High Quality Cell	14.68 %
Total UMI Counts in High Quality Cell	79,958,429
UMI Counts in High Quality Cell	67.1 %
Median UMI Counts per High Quality Cell	3,079
Median Genes per High Quality Cell	1,526
Total Genes Detected in High Quality Cell	24,624
Cell above Mitochondrial Expression Threshold	7.47 %
Estimated Doublet Rate in High Quality Cell	7.92 %

Sequencing Stats

Number of Reads Processed	255,953,705
Reads Pseudoaligned	92.4 %
Reads on Whitelist	95.01 %
Total UMI Counts	119,154,816
Sequencing Technology	10xv3
Species	Arabidopsis thaliana
Transcriptome	TAIR10

Sample Stats

Sample	sc_1
Name	WT control
Source	Benfey lab
Genotype	WT Col-0
Transgene	NA
Treatment	Untreated
Age	7_day
Timepoint	0
Rep	1
Target Cells	10,000
Date	2019-12-14
Seq Run	Nolan_6131

UMI Counts Histogram

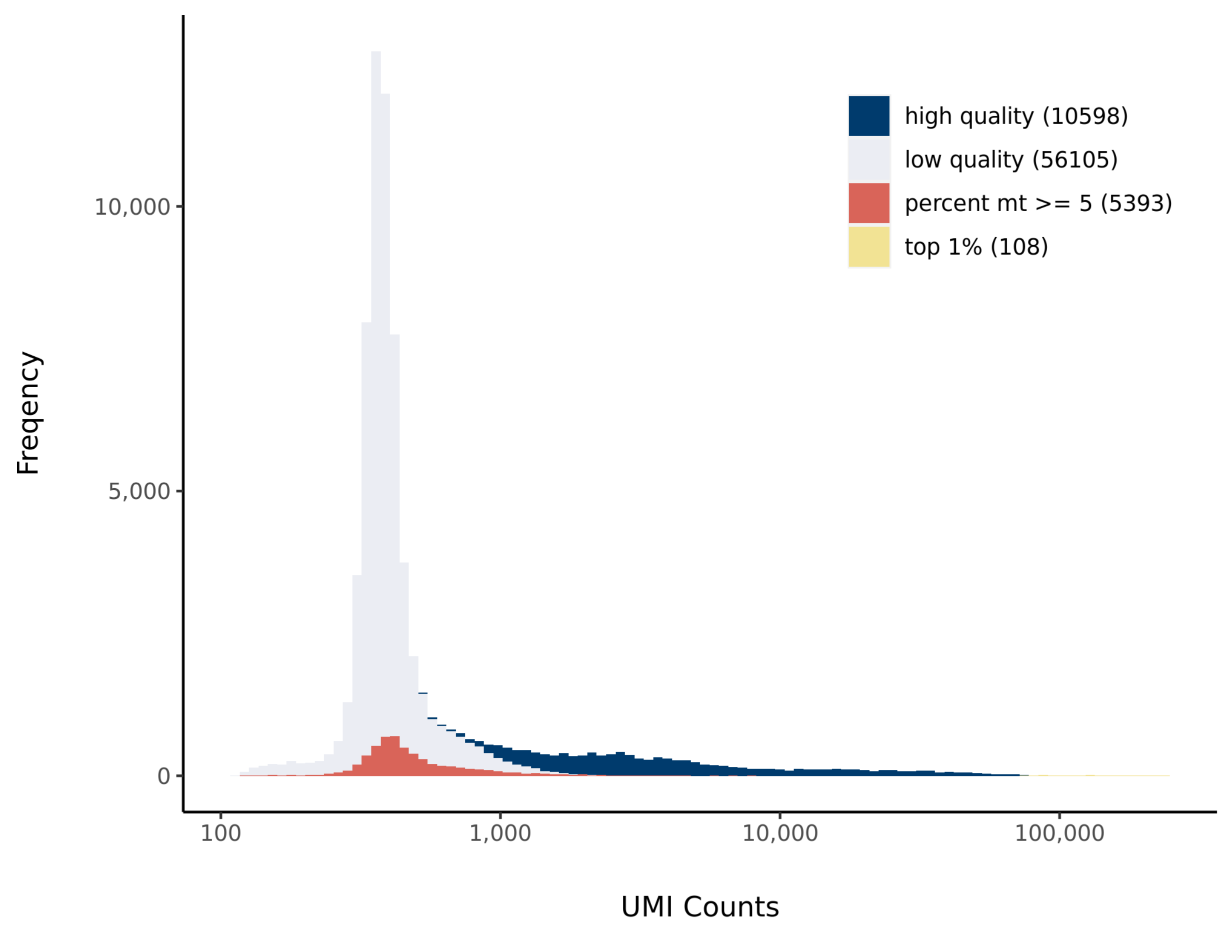

Number of Genes Histogram

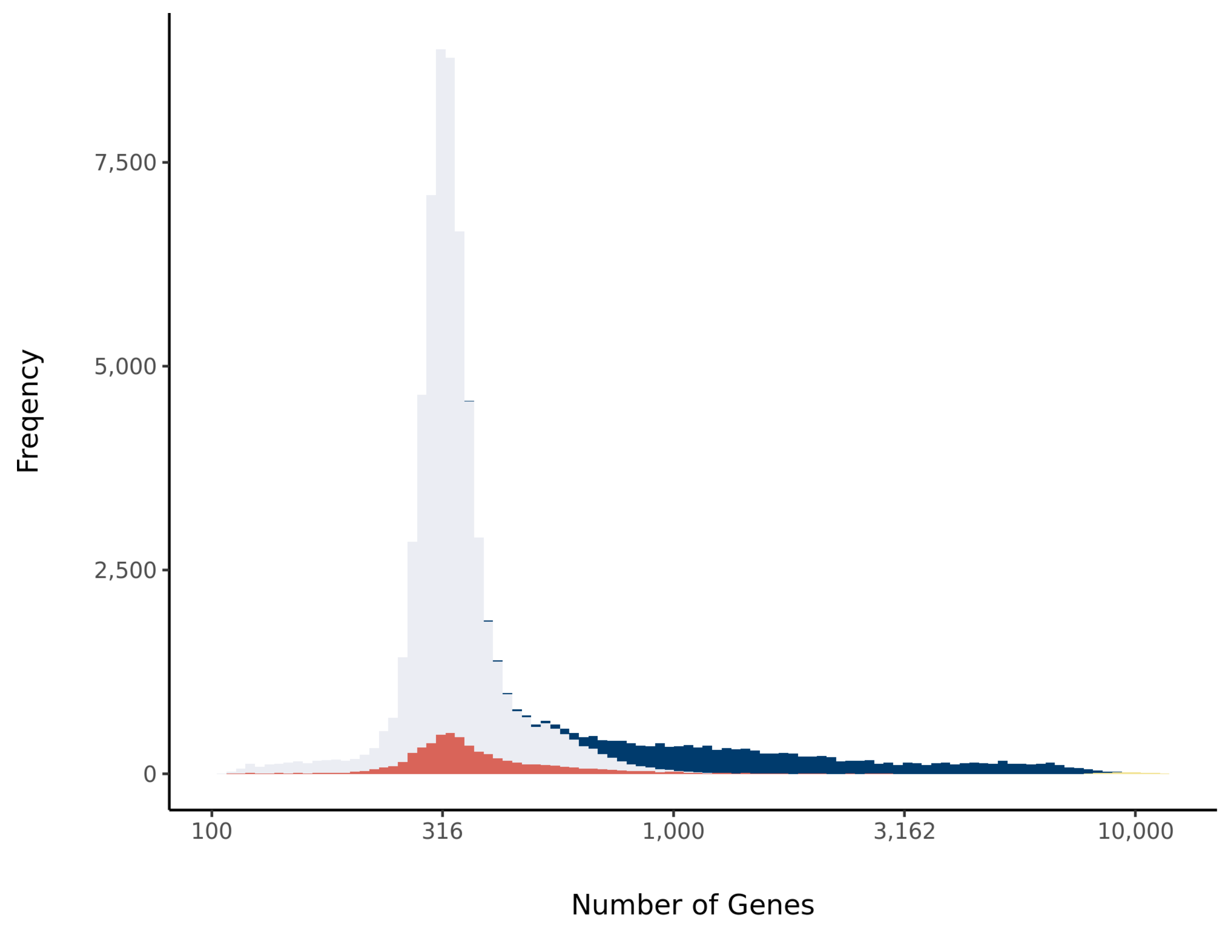

Barcode Rank Plot

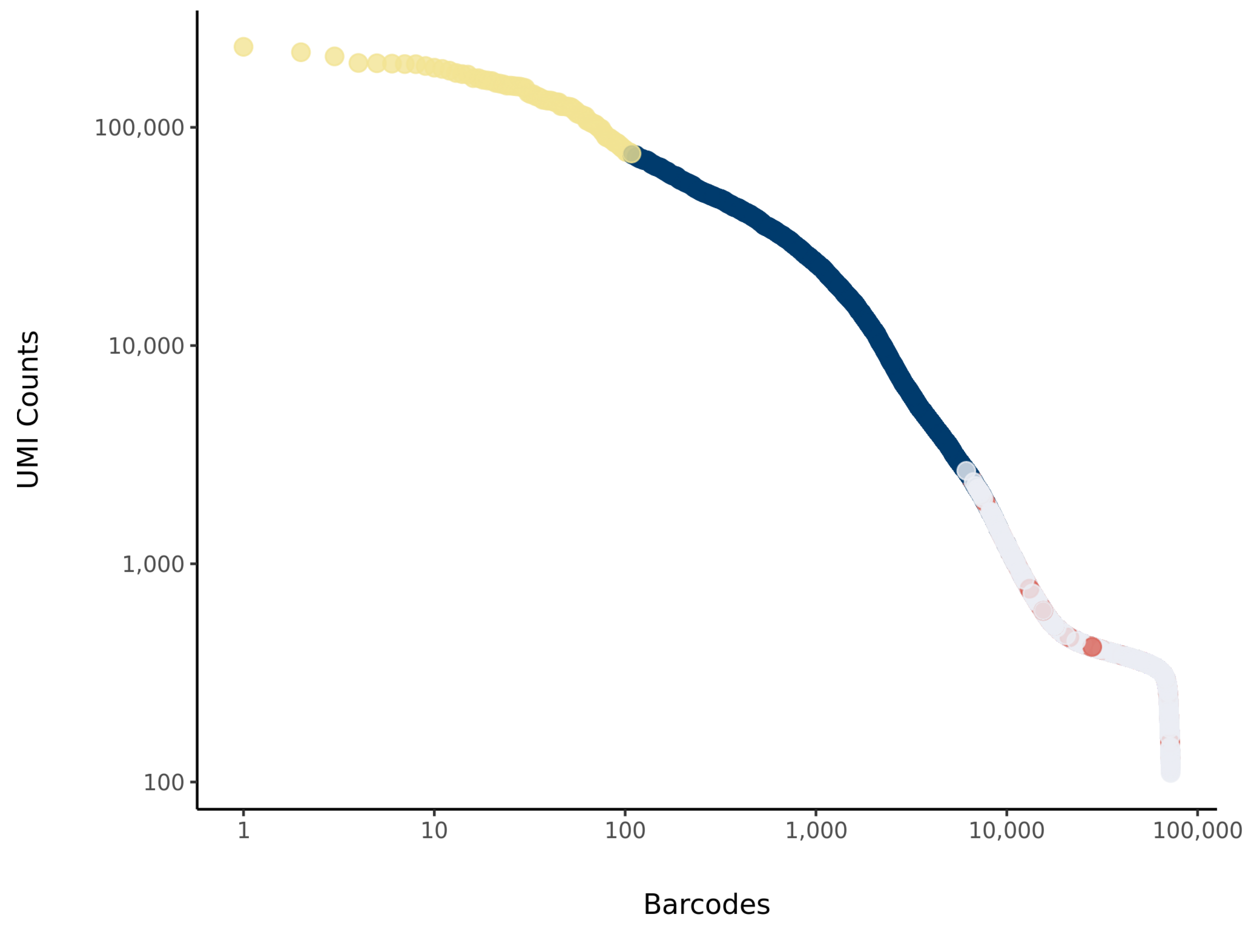

sc_9_at Summary

Processed by COPILOT

Summary | Analysis

Parameters

Iteration of Filtering	1
Mitochondrial Expression Threshold	5 %
Top High Quality Cell Filtered	1 %
Doublet Removed	Yes

Cell Stats

Estimated Number of High Quality Cell	3,926
High Quality Cell	5.9 %
Total UMI Counts in High Quality Cell	67,227,229
UMI Counts in High Quality Cell	54.25 %
Median UMI Counts per High Quality Cell	9,539
Median Genes per High Quality Cell	3,046
Total Genes Detected in High Quality Cell	24,999
Cell above Mitochondrial Expression Threshold	2.82 %
Estimated Doublet Rate in High Quality Cell	3.01 %

Sequencing Stats

Number of Reads Processed	271,063,644
Reads Pseudoaligned	91.8 %
Reads on Whitelist	93.83 %
Total UMI Counts	123,926,323
Sequencing Technology	10xv3
Species	Arabidopsis thaliana
Transcriptome	TAIR10

Sample Stats

Sample	sc_9
Name	Rice 1 cm and Arabidopsis
Source	Benfey lab
Genotype	X.Kitaake; WT Col-0
Transgene	NA
Treatment	Untreated
Age	2_day; 5-day
Timepoint	NA
Rep	NA
Target Cells	10,000
Date	2019-12-18
Seq Run	Nolan_6131

UMI Counts Histogram

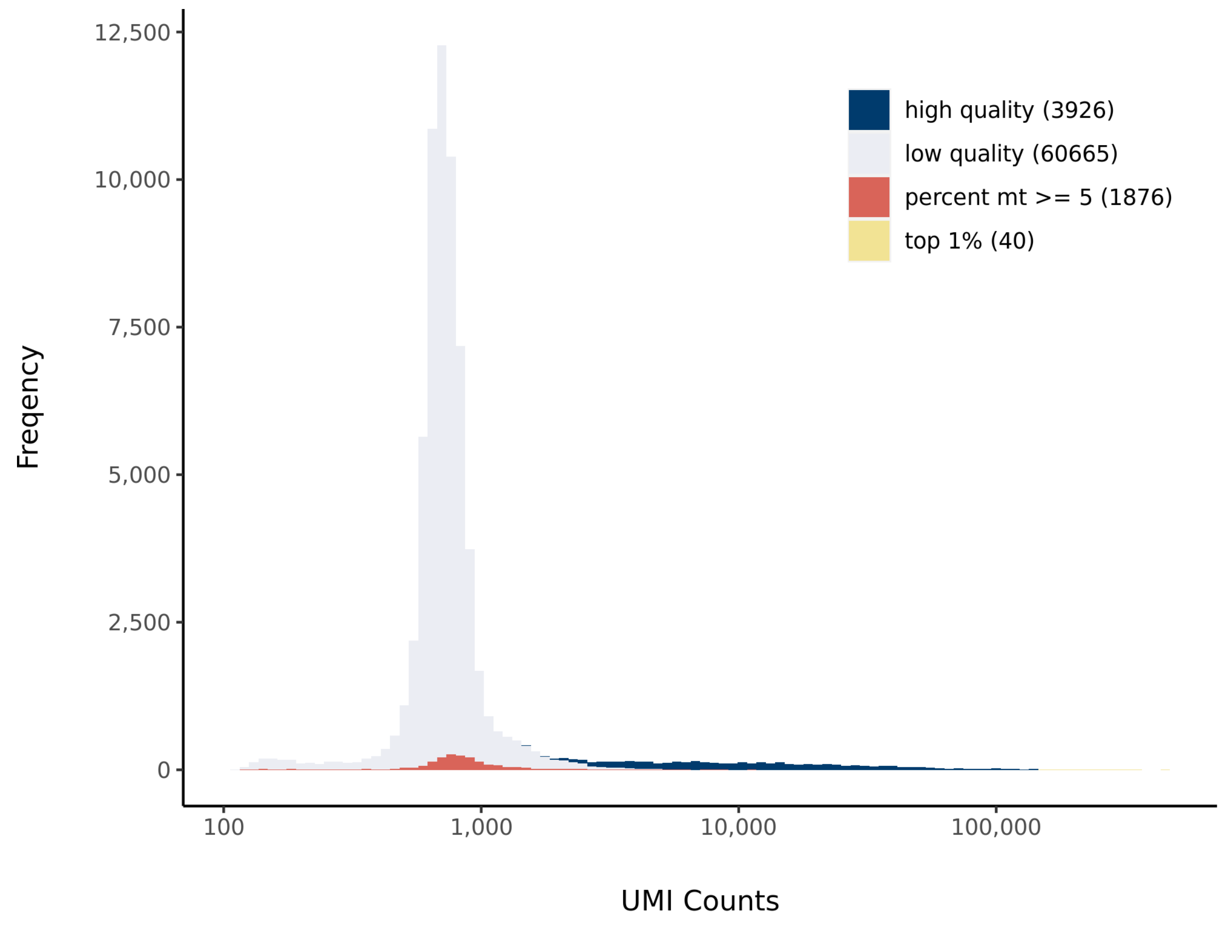

Number of Genes Histogram

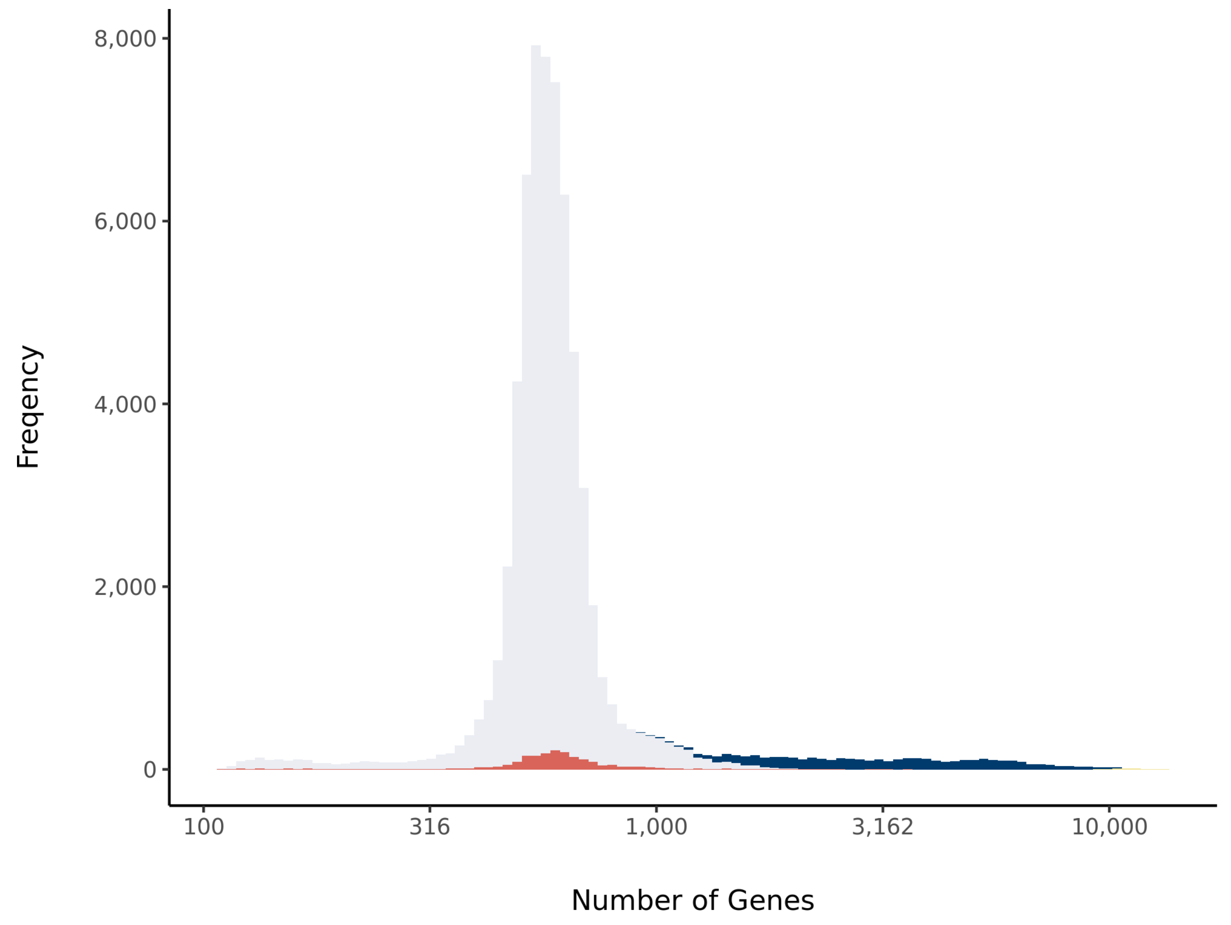

Barcode Rank Plot

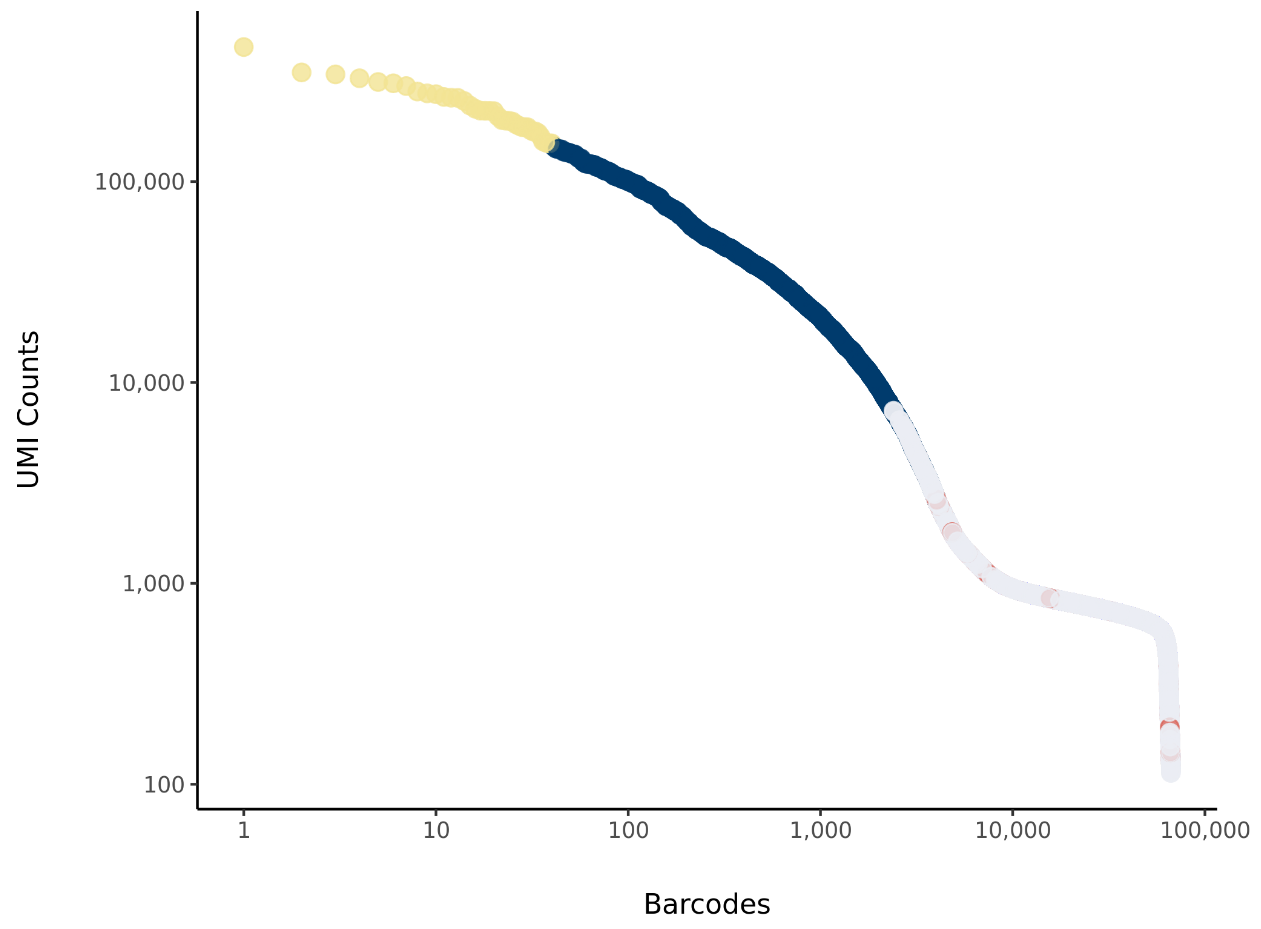

sc_10_at Summary

Processed by COPILOT

Summary Analysis

Parameters

Iteration of Filtering	1
Mitochondrial Expression Threshold	5 %
Top High Quality Cell Filtered	1 %
Doublet Removed	Yes

Cell Stats

Estimated Number of High Quality Cell	10,040
High Quality Cell	13.38 %
Total UMI Counts in High Quality Cell	100,751,671
UMI Counts in High Quality Cell	75.06 %
Median UMI Counts per High Quality Cell	5,553
Median Genes per High Quality Cell	2,279
Total Genes Detected in High Quality Cell	25,502
Cell above Mitochondrial Expression Threshold	69.11 %
Estimated Doublet Rate in High Quality Cell	7.51 %

Sequencing Stats

Number of Reads Processed	300,927,223
Reads Pseudoaligned	90.4 %
Reads on Whitelist	92.36 %
Total UMI Counts	134,231,998
Sequencing Technology	10xv3
Species	Arabidopsis thaliana
Transcriptome	TAIR10

Sample Stats

Sample	sc_10
Name	Rice 1 cm and Arabidopsis
Source	Benfey lab
Genotype	X.Kitaake; WT Col-0
Transgene	NA
Treatment	Untreated
Age	2_day; 5-day
Timepoint	NA
Rep	NA
Target Cells	20,000
Date	2019-12-18
Seq Run	Nolan_6131

UMI Counts Histogram

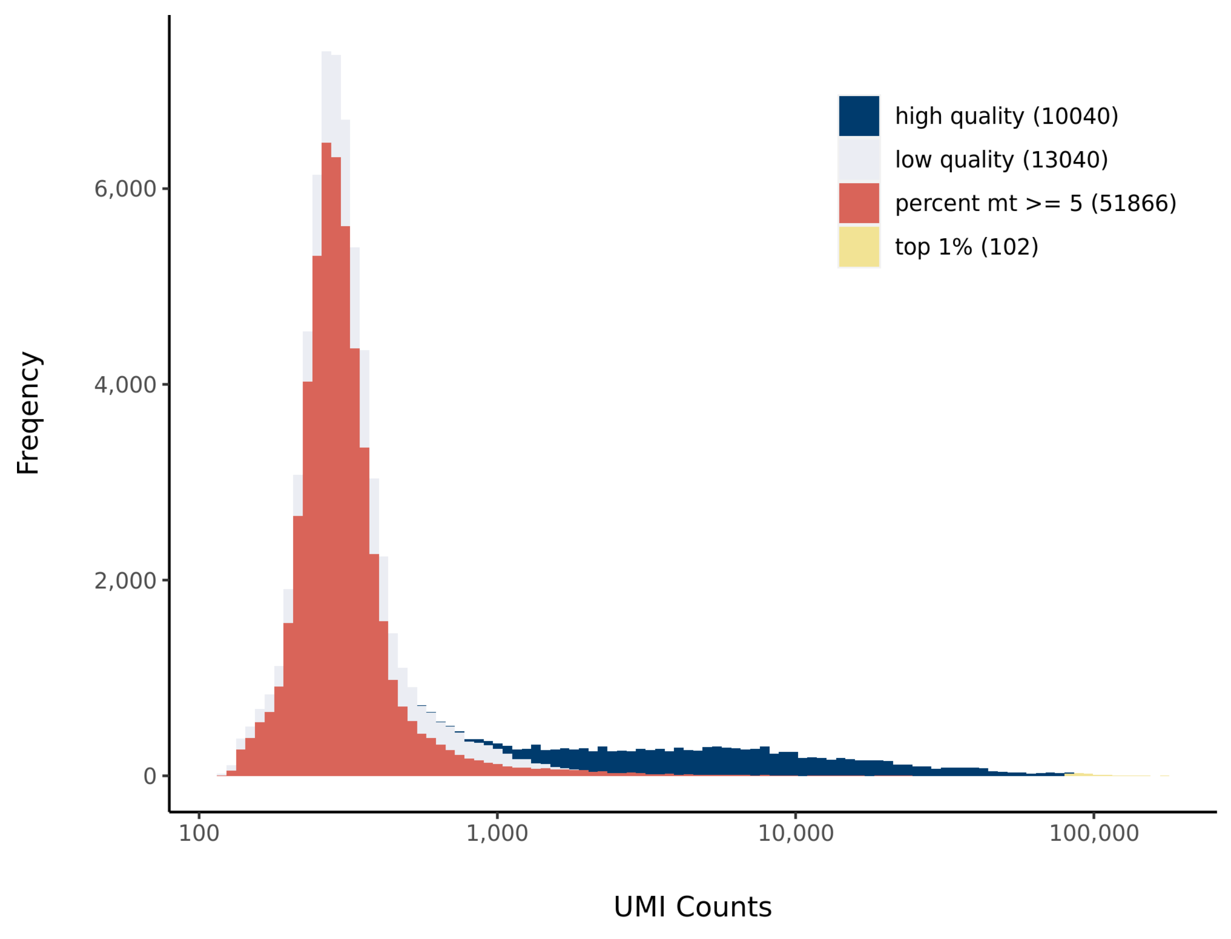

Number of Genes Histogram

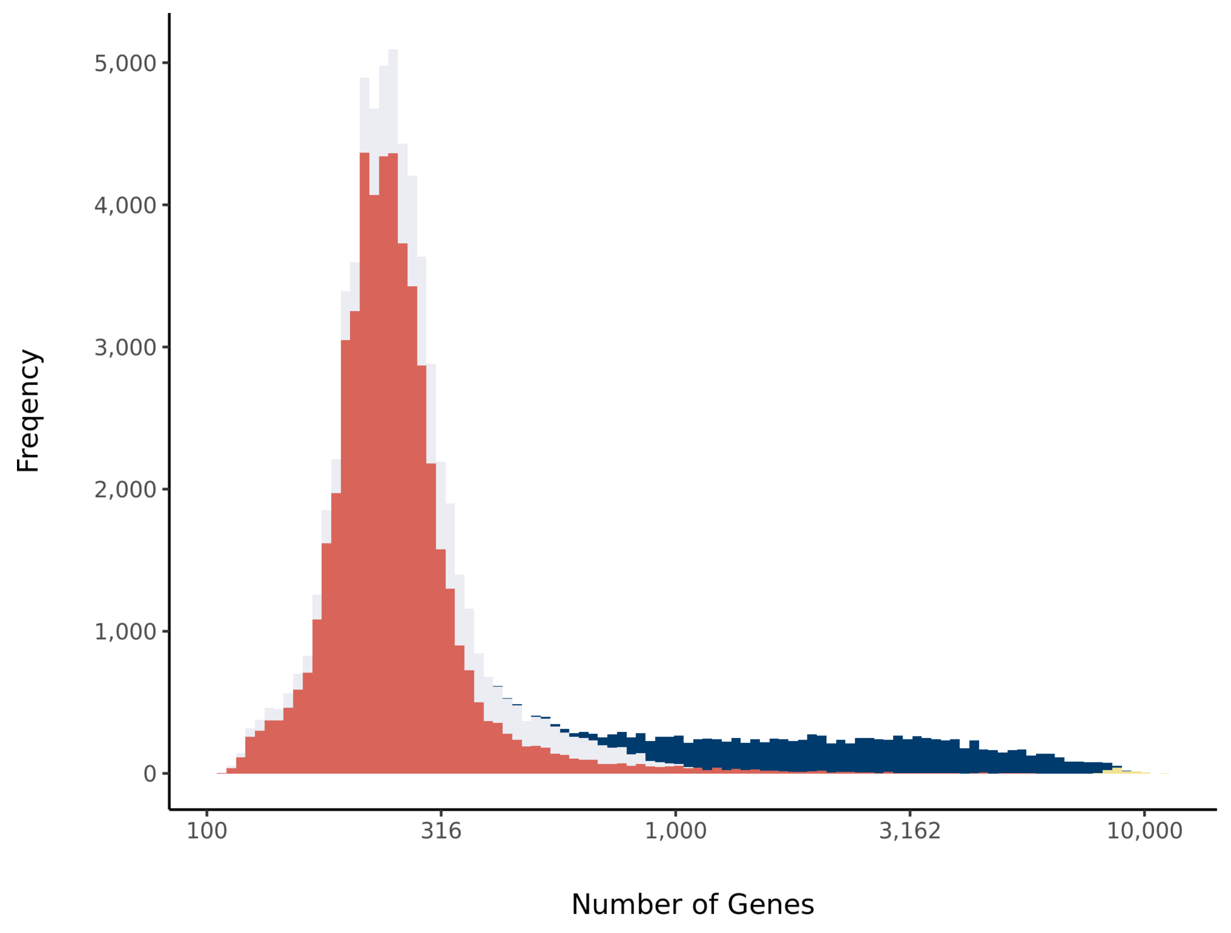

Barcode Rank Plot

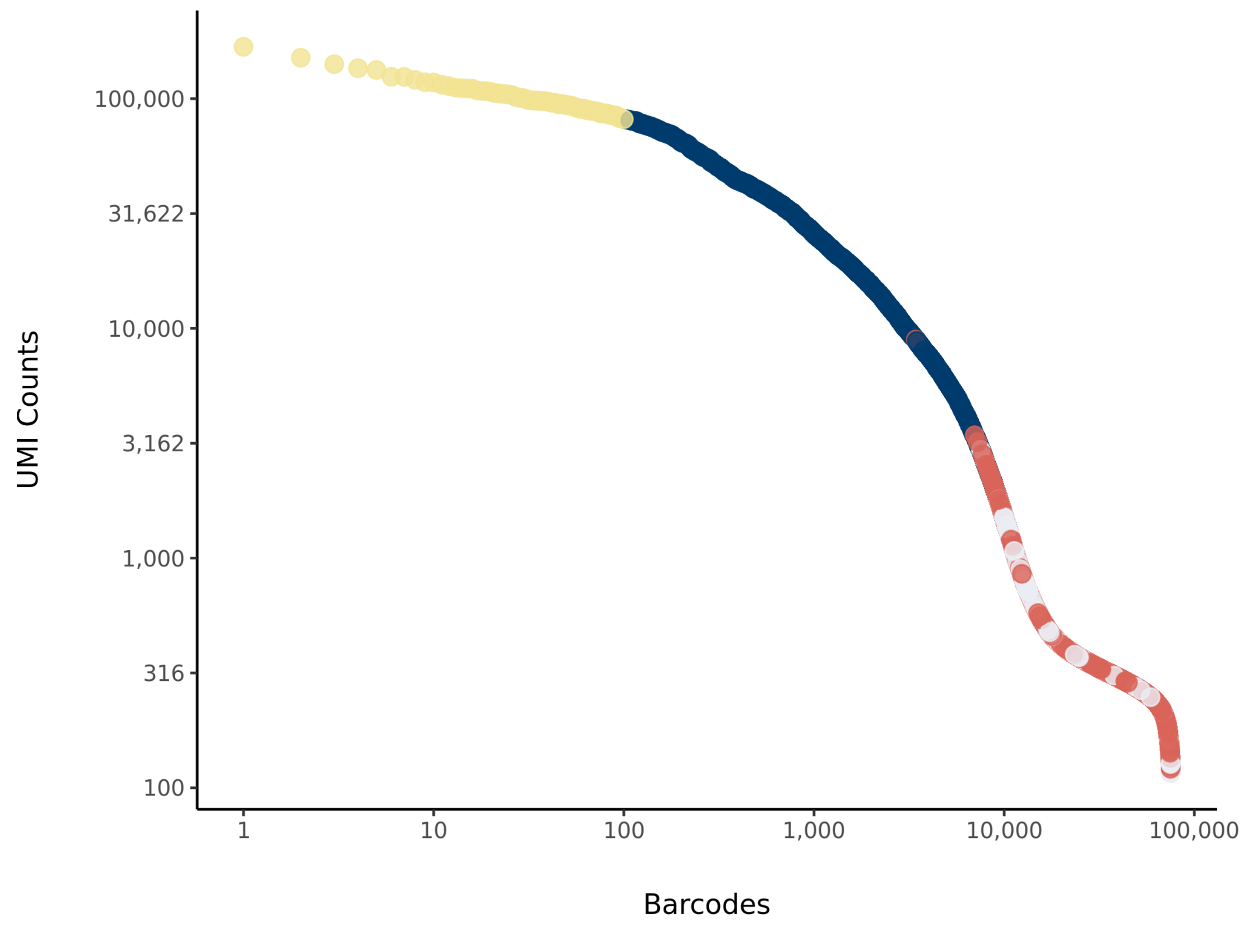

sc_11 Summary

Processed by COPILOT

Summary | Analysis

Parameters

Iteration of Filtering	1
Mitochondrial Expression Threshold	5 %
Top High Quality Cell Filtered	1 %
Doublet Removed	Yes

Cell Stats

Estimated Number of High Quality Cell	9,913
High Quality Cell	13.96 %
Total UMI Counts in High Quality Cell	168,126,295
UMI Counts in High Quality Cell	82.76 %
Median UMI Counts per High Quality Cell	9,146
Median Genes per High Quality Cell	3,050
Total Genes Detected in High Quality Cell	25,040
Cell above Mitochondrial Expression Threshold	8.81 %
Estimated Doublet Rate in High Quality Cell	7.42 %

Sequencing Stats

Number of Reads Processed	329,949,998
Reads Pseudoaligned	93.6 %
Reads on Whitelist	95.21 %
Total UMI Counts	203,138,562
Sequencing Technology	10xv3
Species	Arabidopsis thaliana
Transcriptome	TAIR10

Sample Stats

Sample	sc_11
Name	WT Col-0 untreated
Source	Benfey lab
Genotype	WT Col-0
Transgene	NA
Treatment	Untreated
Age	5_day
Timepoint	NA
Rep	NA
Target Cells	10,000
Date	2019-12-20
Seq Run	Nolan_6131

UMI Counts Histogram

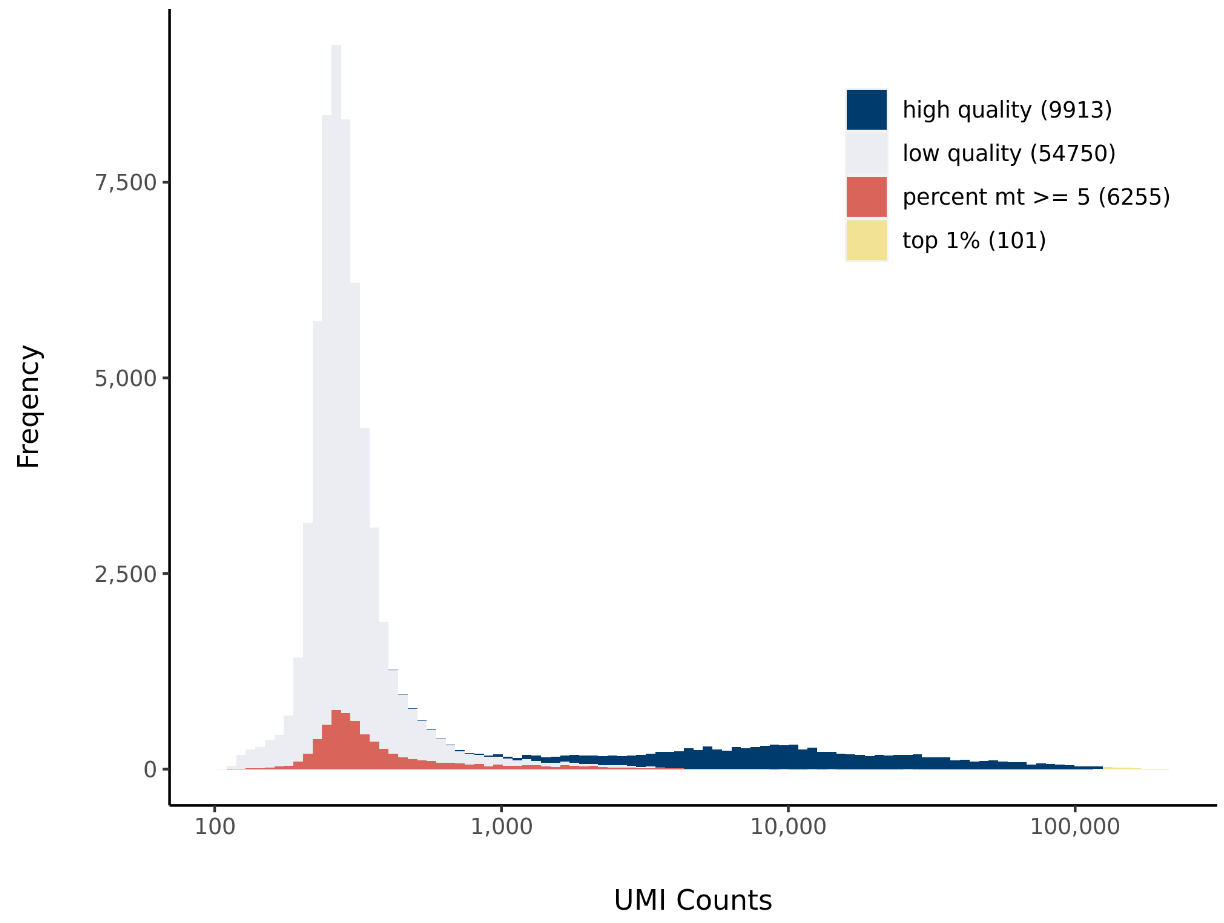

Number of Genes Histogram

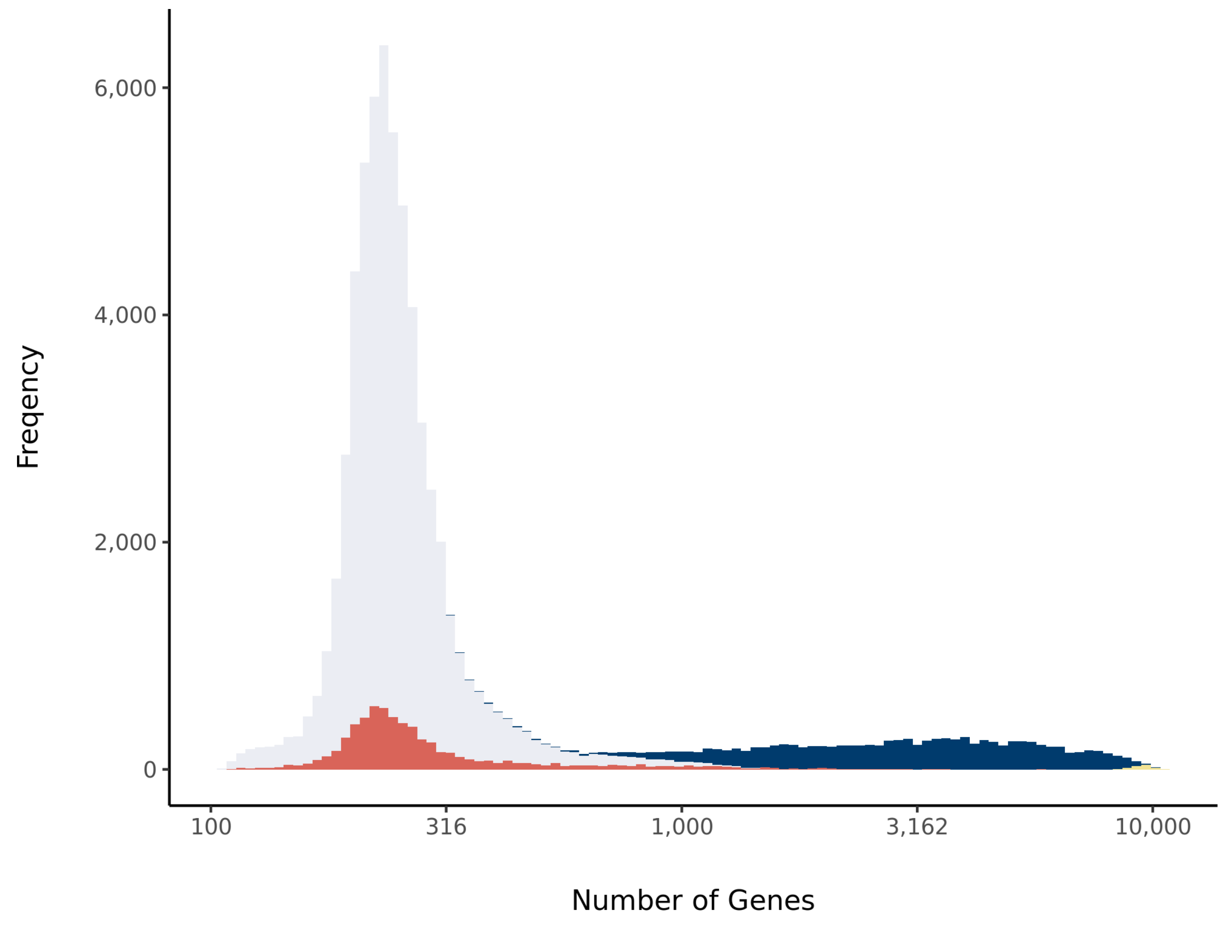

Barcode Rank Plot

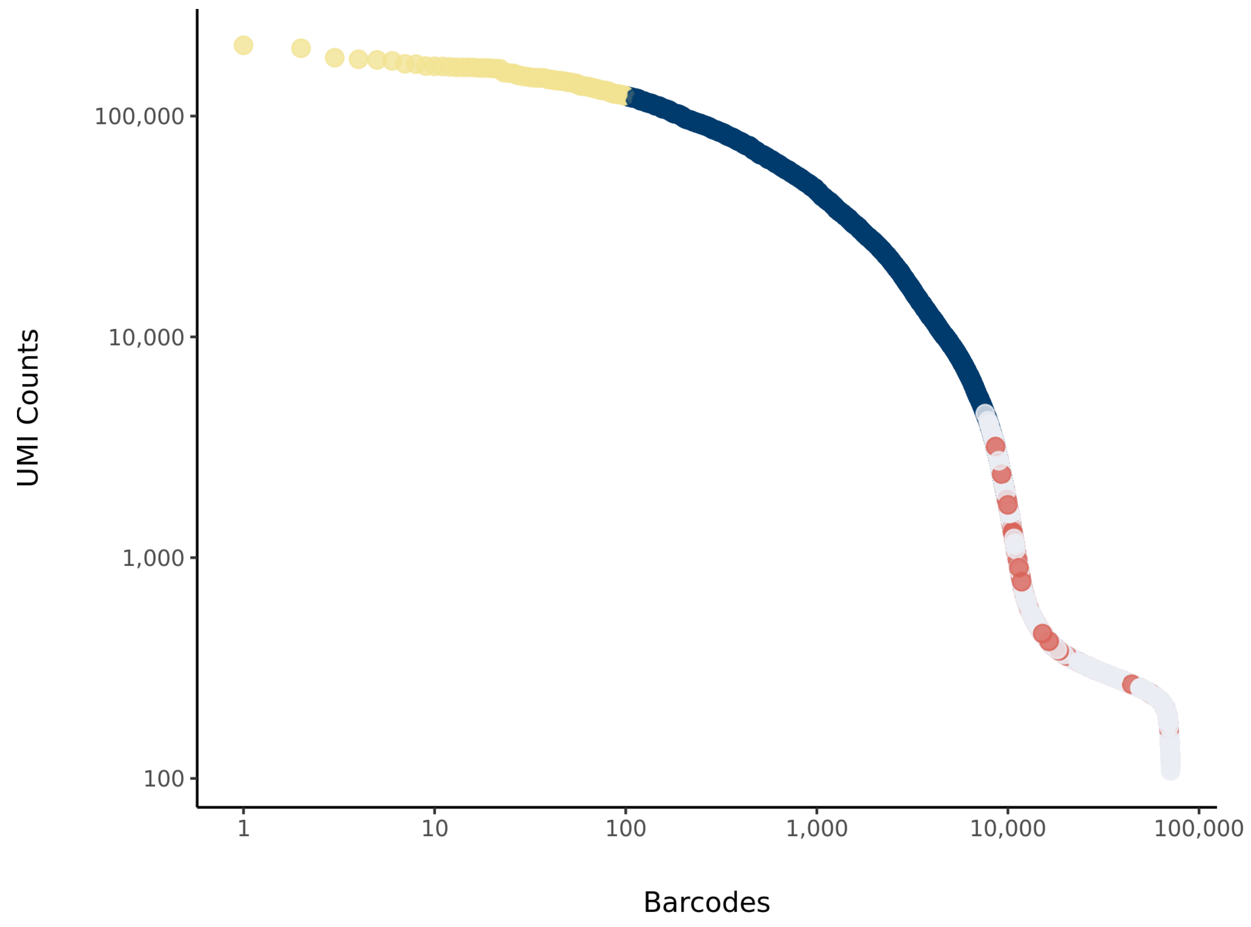

sc_12 Summary

Processed by COPILOT

Summary | Analysis

Parameters

Iteration of Filtering	1
Mitochondrial Expression Threshold	5 %
Top High Quality Cell Filtered	1 %
Doublet Removed	Yes

Cell Stats

Estimated Number of High Quality Cell	11,236
High Quality Cell	13.59 %
Total UMI Counts in High Quality Cell	346,882,014
UMI Counts in High Quality Cell	83.04 %
Median UMI Counts per High Quality Cell	20,788.5
Median Genes per High Quality Cell	4,774
Total Genes Detected in High Quality Cell	26,018
Cell above Mitochondrial Expression Threshold	6.77 %
Estimated Doublet Rate in High Quality Cell	8.39 %

Sequencing Stats

Number of Reads Processed	841,129,261
Reads Pseudoaligned	93.7 %
Reads on Whitelist	96.04 %
Total UMI Counts	417,731,033
Sequencing Technology	10xv3
Species	Arabidopsis thaliana
Transcriptome	TAIR10

Sample Stats

Sample	sc_12
Name	WT Col-0 untreated
Source	Benfey lab
Genotype	WT Col-0
Transgene	NA
Treatment	Untreated
Age	5_day
Timepoint	NA
Rep	NA
Target Cells	10,000
Date	2019-12-20
Seq Run	Nolan_6131;Shahan_6158

UMI Counts Histogram

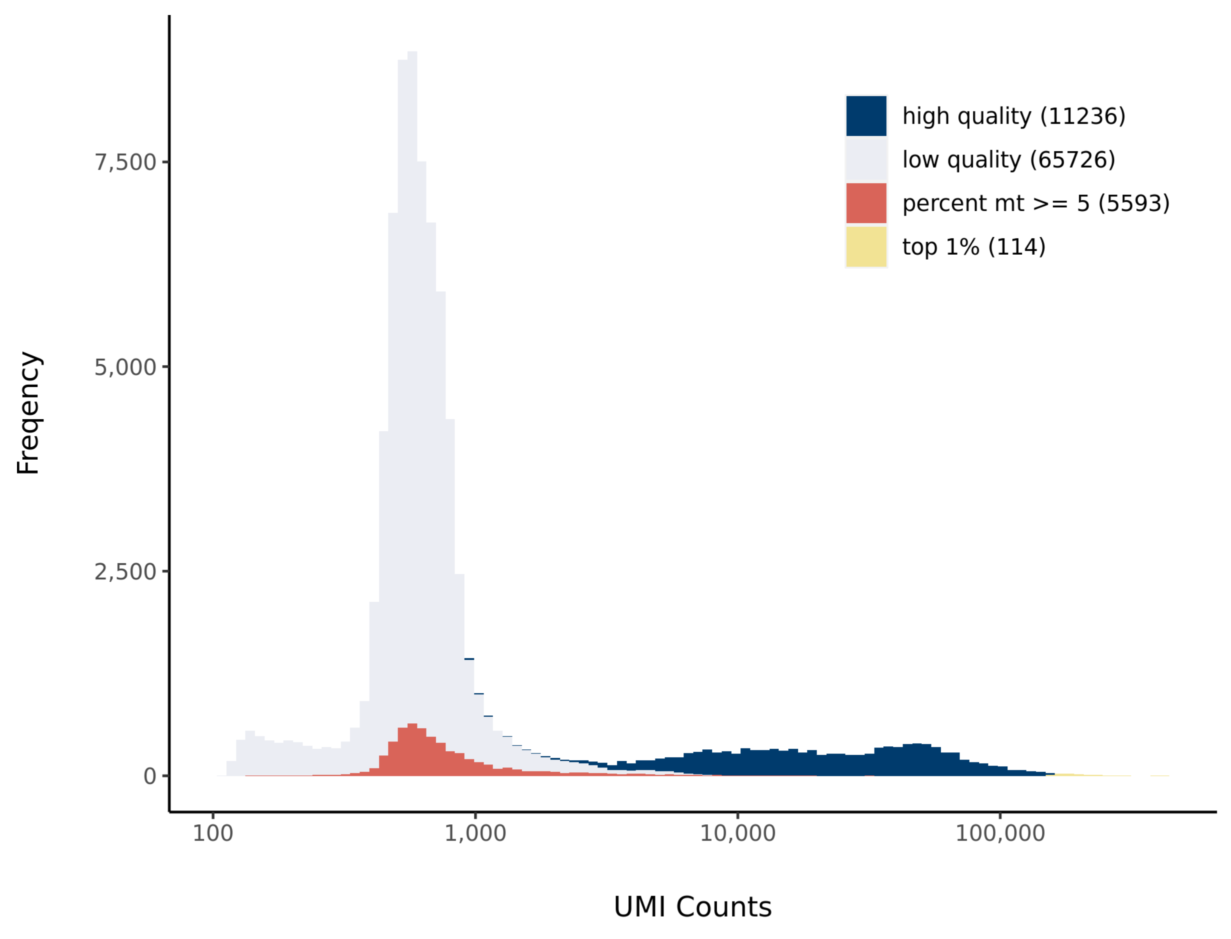

Number of Genes Histogram

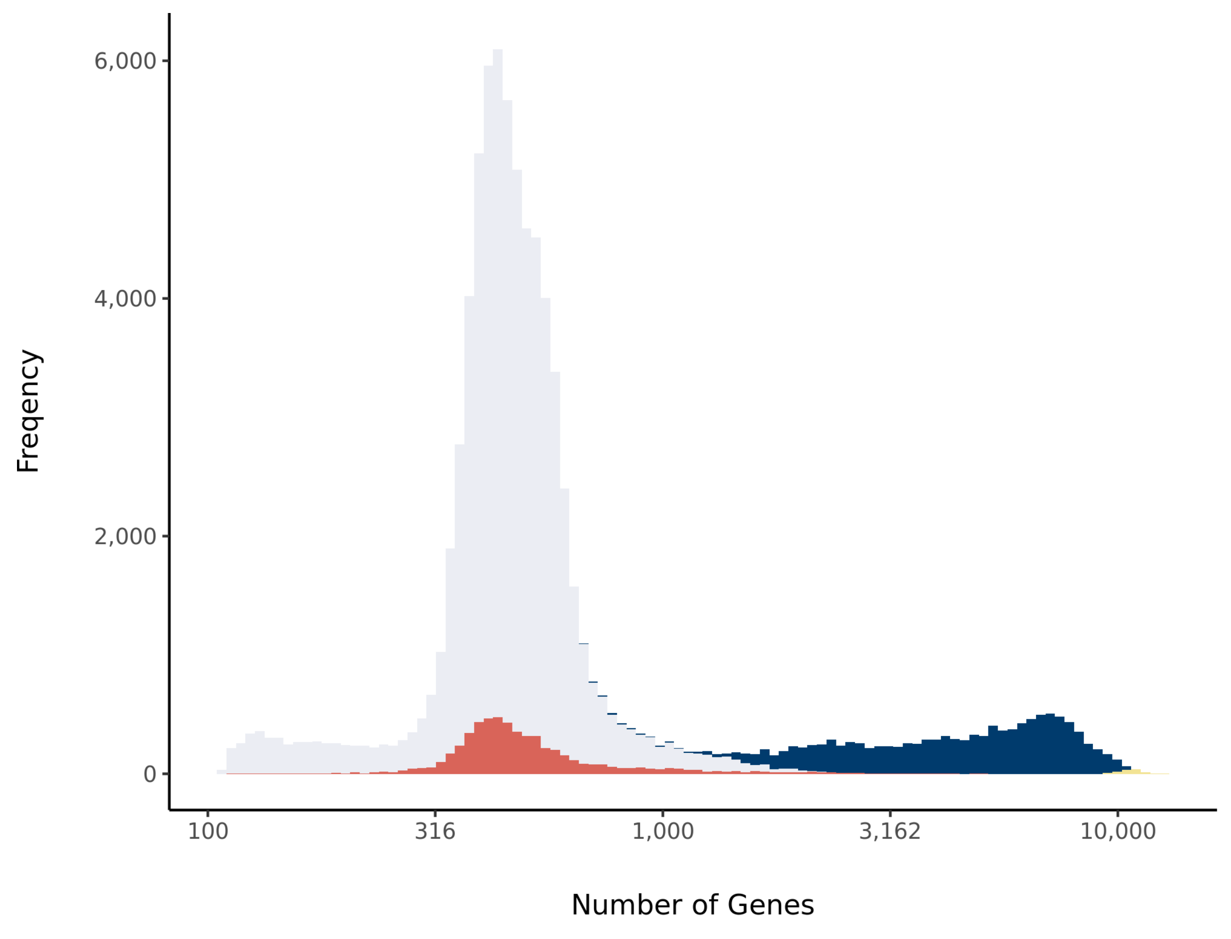

Barcode Rank Plot

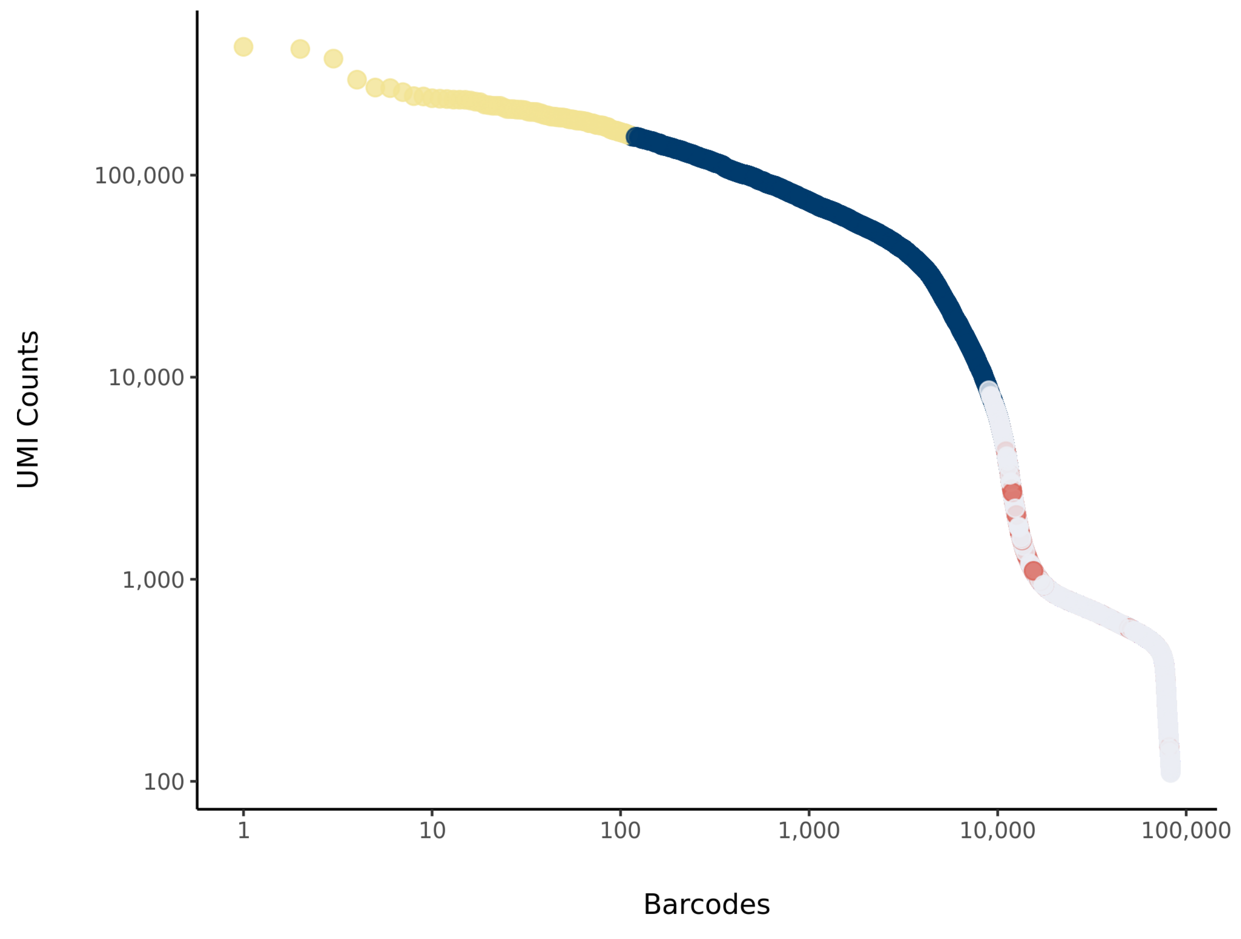

sc_20 Summary

Processed by COPILOT

Summary Analysis

Parameters

Iteration of Filtering	1
Mitochondrial Expression Threshold	5 %
Top High Quality Cell Filtered	1 %
Doublet Removed	Yes

Cell Stats

Estimated Number of High Quality Cell	14,064
High Quality Cell	19.46 %
Total UMI Counts in High Quality Cell	135,176,770
UMI Counts in High Quality Cell	54.36 %
Median UMI Counts per High Quality Cell	6,497
Median Genes per High Quality Cell	2,711
Total Genes Detected in High Quality Cell	25,325
Cell above Mitochondrial Expression Threshold	0.93 %
Estimated Doublet Rate in High Quality Cell	10.47 %

Sequencing Stats

Number of Reads Processed	327,662,124
Reads Pseudoaligned	94.7 %
Reads on Whitelist	96.89 %
Total UMI Counts	248,691,560
Sequencing Technology	10xv3
Species	Arabidopsis thaliana
Transcriptome	TAIR10

Sample Stats

Sample	sc_20
Name	WT Col-0_RS1
Source	Benfey lab
Genotype	WT Col-0
Transgene	NA
Treatment	Untreated
Age	5_day
Timepoint	NA
Rep	NA
Target Cells	10,000
Date	NA
Seq Run	Shahan_6177

UMI Counts Histogram

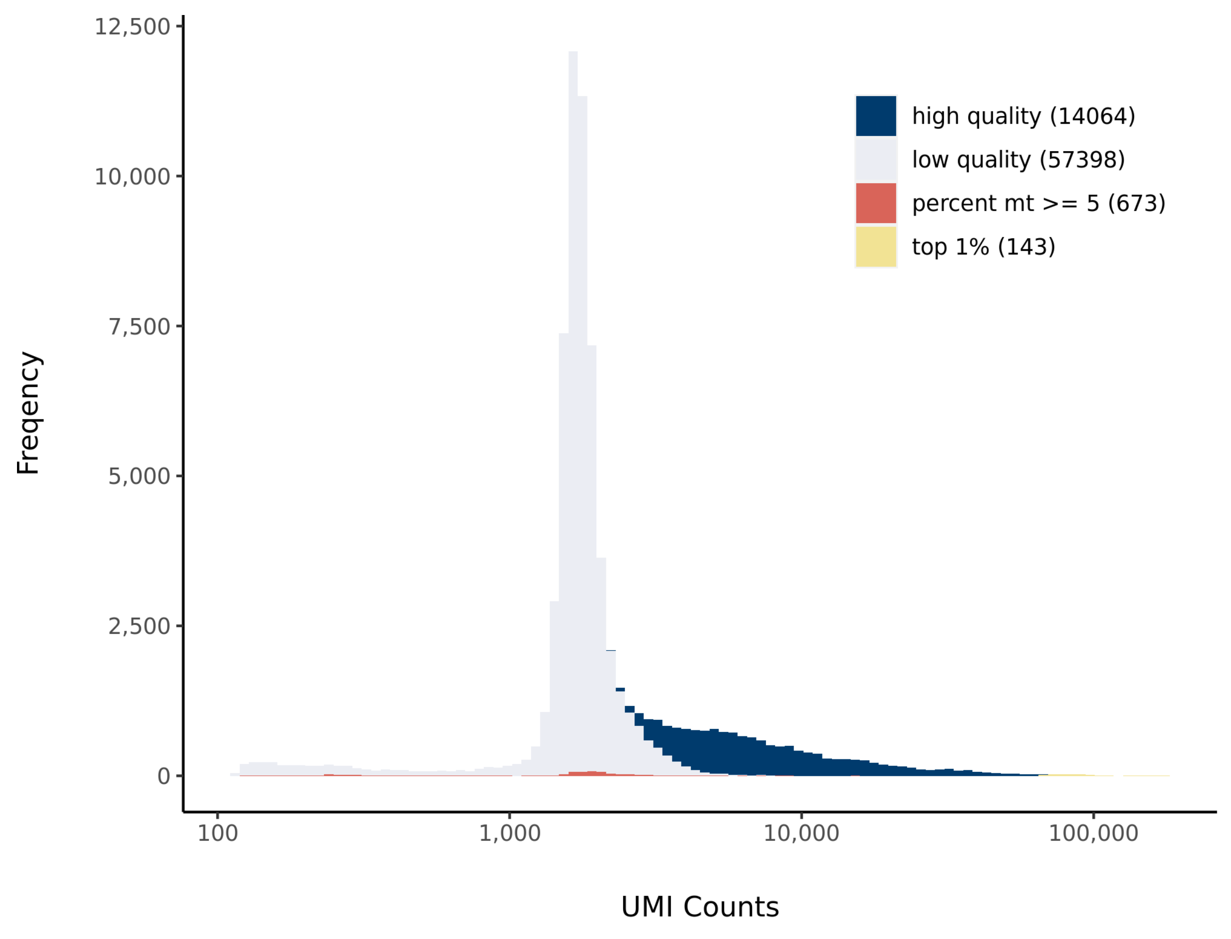

Number of Genes Histogram

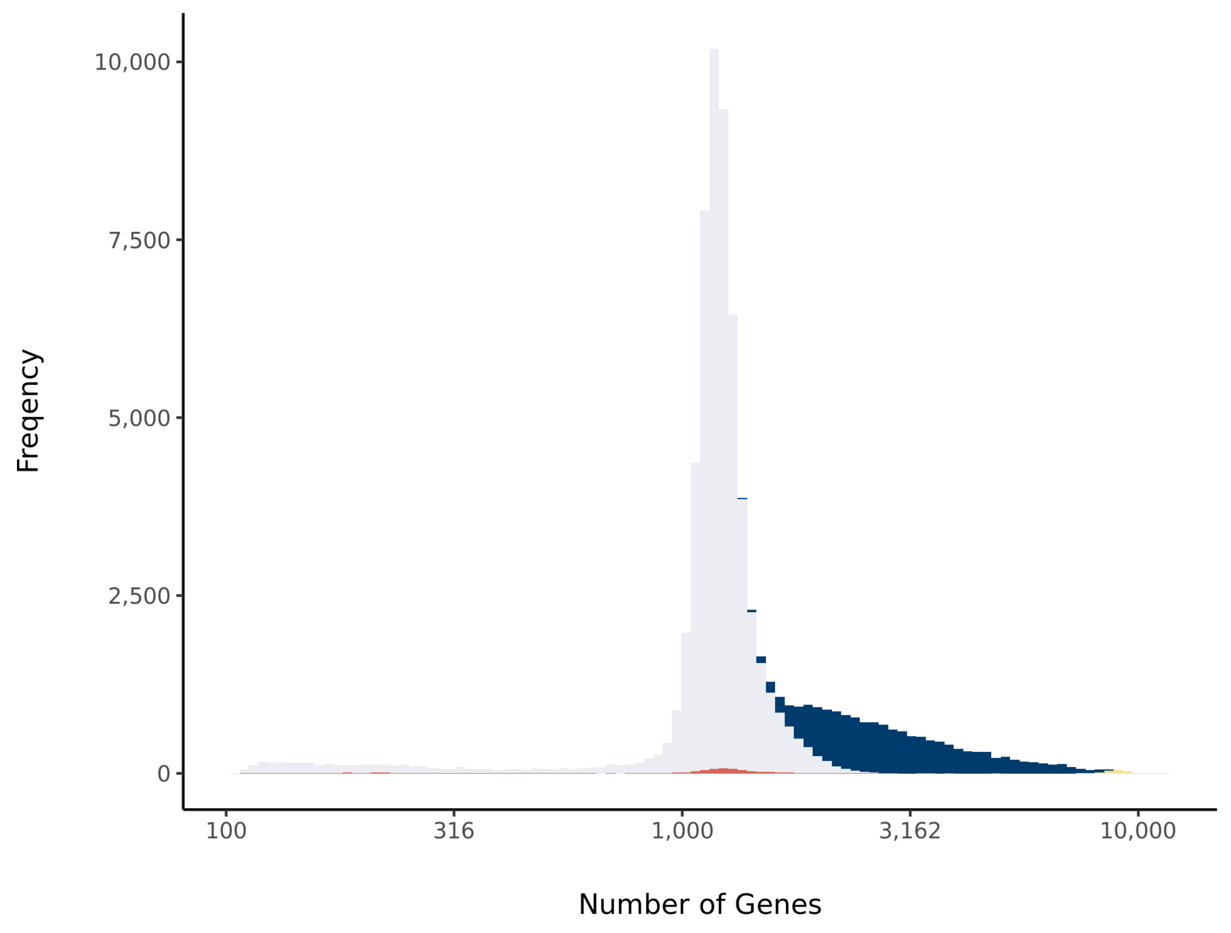

Barcode Rank Plot

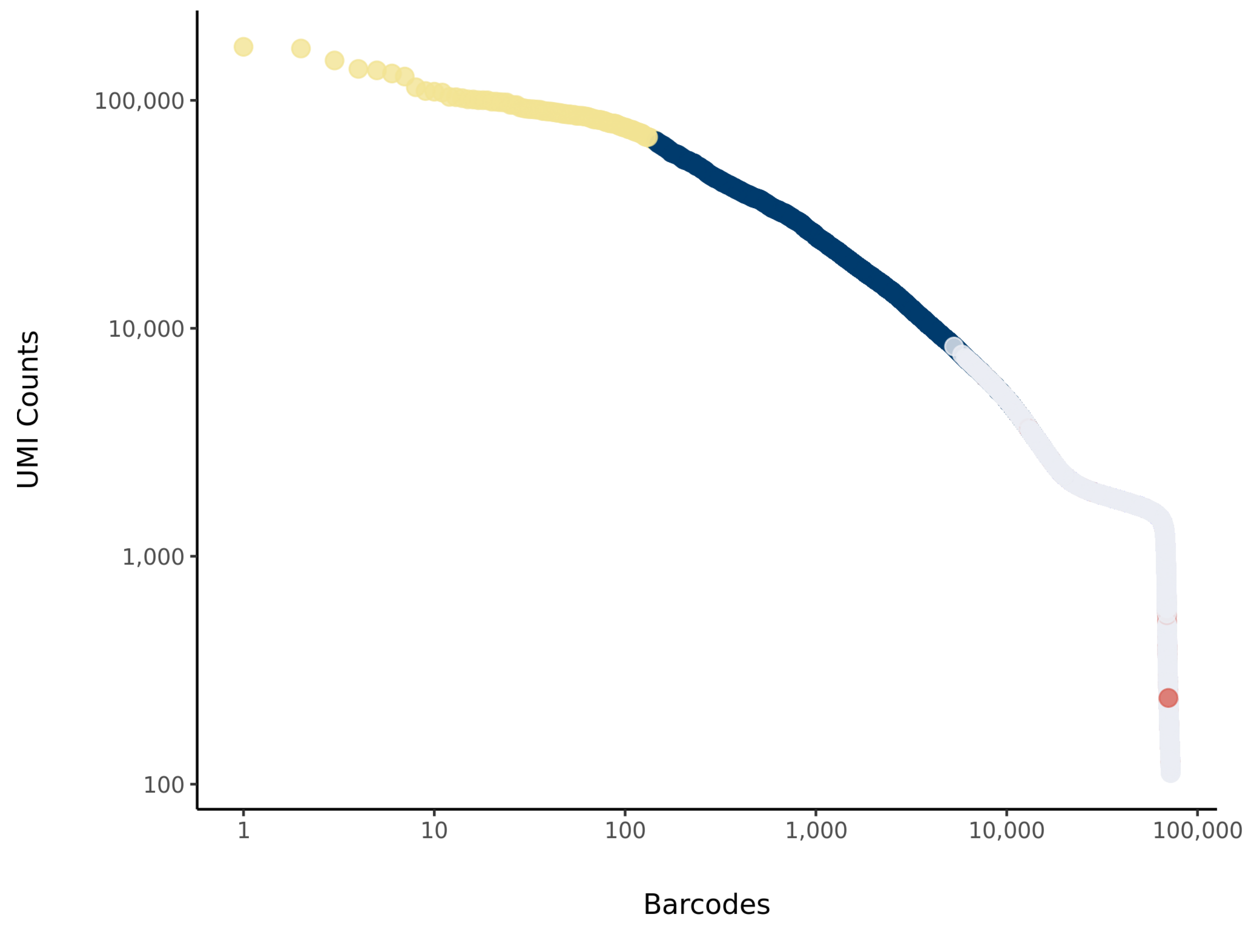

sc_21 Summary

Processed by COPILOT

Summary | Analysis

Parameters

Iteration of Filtering	1
Mitochondrial Expression Threshold	5 %
Top High Quality Cell Filtered	1 %
Doublet Removed	Yes

Cell Stats

Estimated Number of High Quality Cell	10,478
High Quality Cell	14.65 %
Total UMI Counts in High Quality Cell	128,289,748
UMI Counts in High Quality Cell	53.13 %
Median UMI Counts per High Quality Cell	7,833.5
Median Genes per High Quality Cell	2,899
Total Genes Detected in High Quality Cell	25,296
Cell above Mitochondrial Expression Threshold	0.53 %
Estimated Doublet Rate in High Quality Cell	7.83 %

Sequencing Stats

Number of Reads Processed	318,494,791
Reads Pseudoaligned	95.2 %
Reads on Whitelist	96.95 %
Total UMI Counts	241,458,602
Sequencing Technology	10xv3
Species	Arabidopsis thaliana
Transcriptome	TAIR10

Sample Stats

Sample	sc_21
Name	WT Col-0_RS2
Source	Benfey lab
Genotype	WT Col-0
Transgene	NA
Treatment	Untreated
Age	5_day
Timepoint	NA
Rep	NA
Target Cells	10,000
Date	NA
Seq Run	Shahan_6177

UMI Counts Histogram

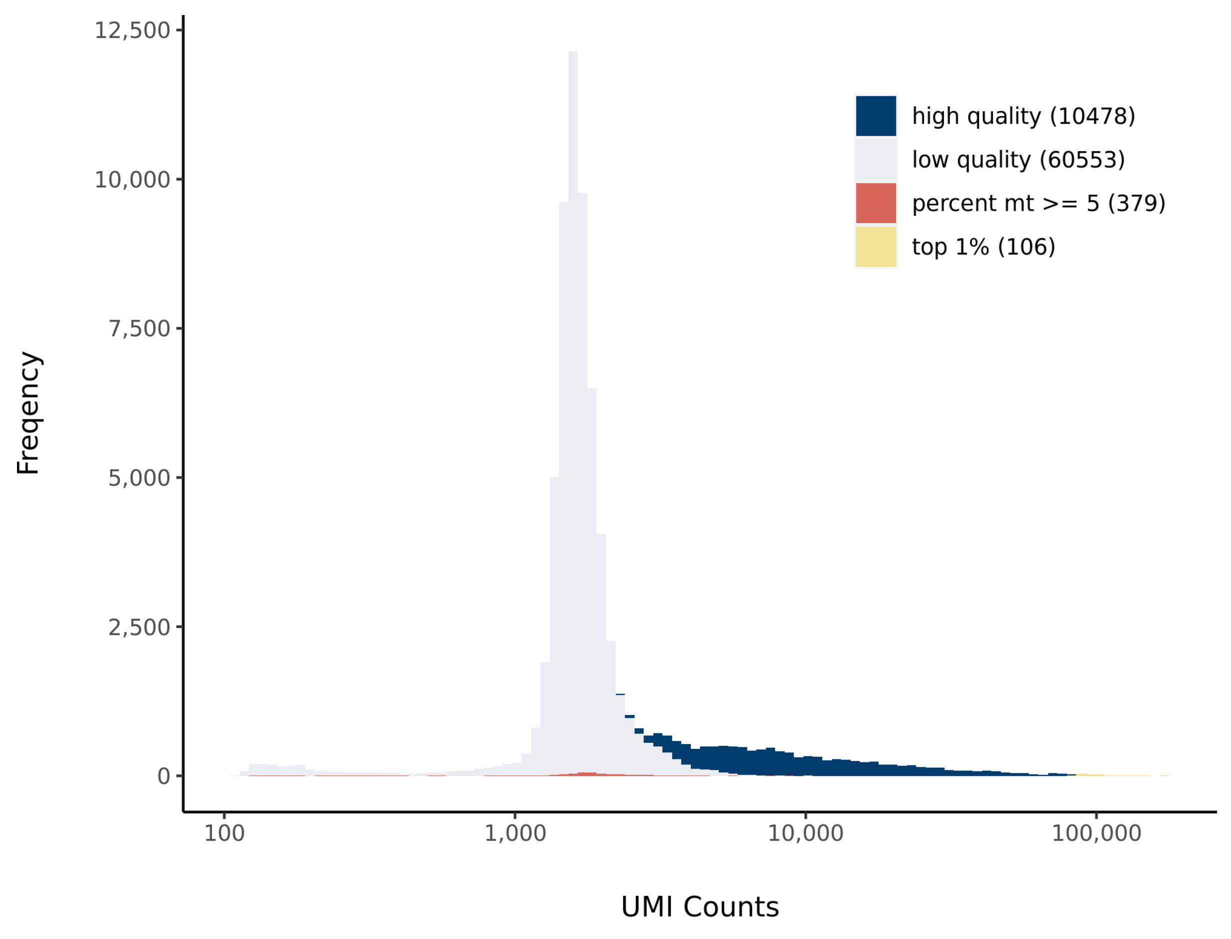

Number of Genes Histogram

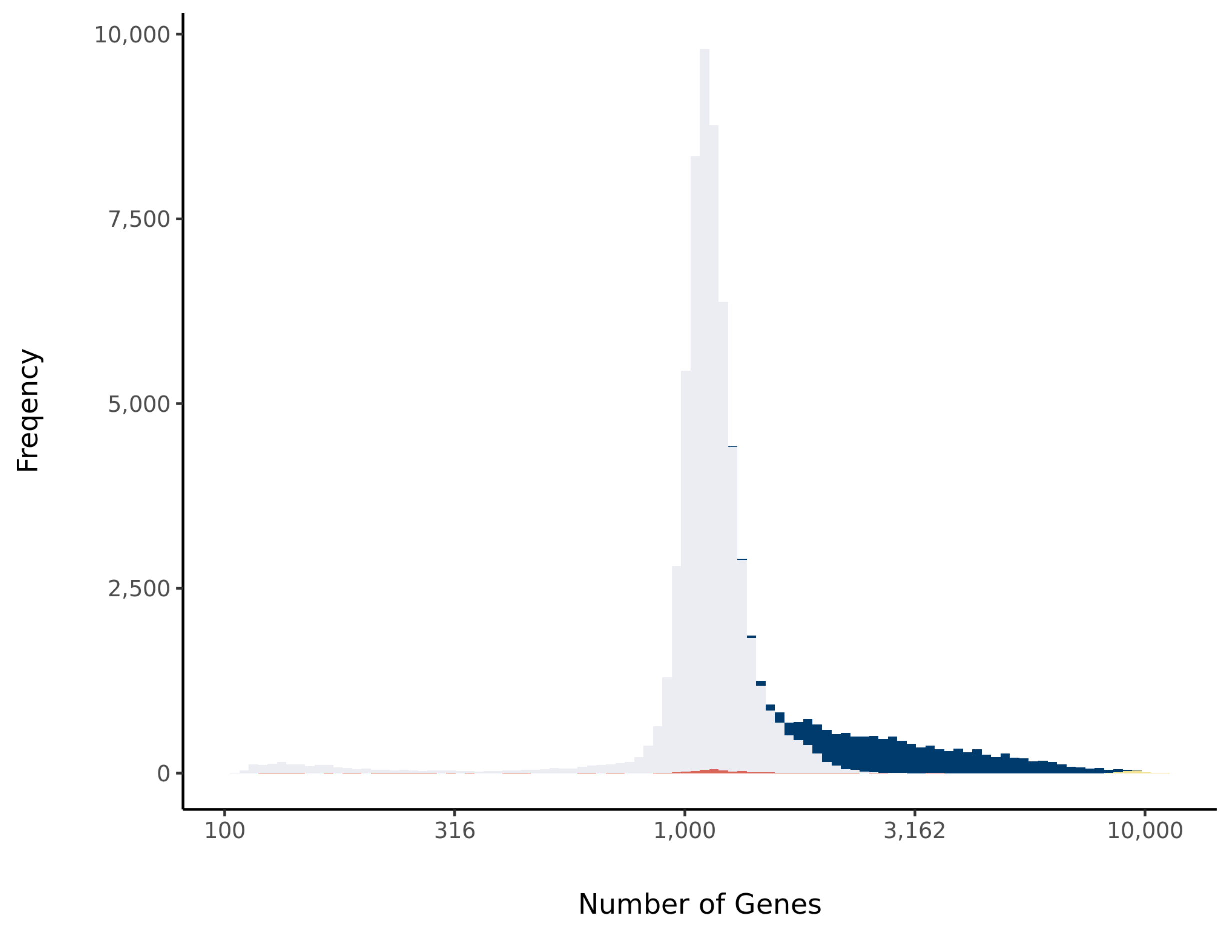

Barcode Rank Plot

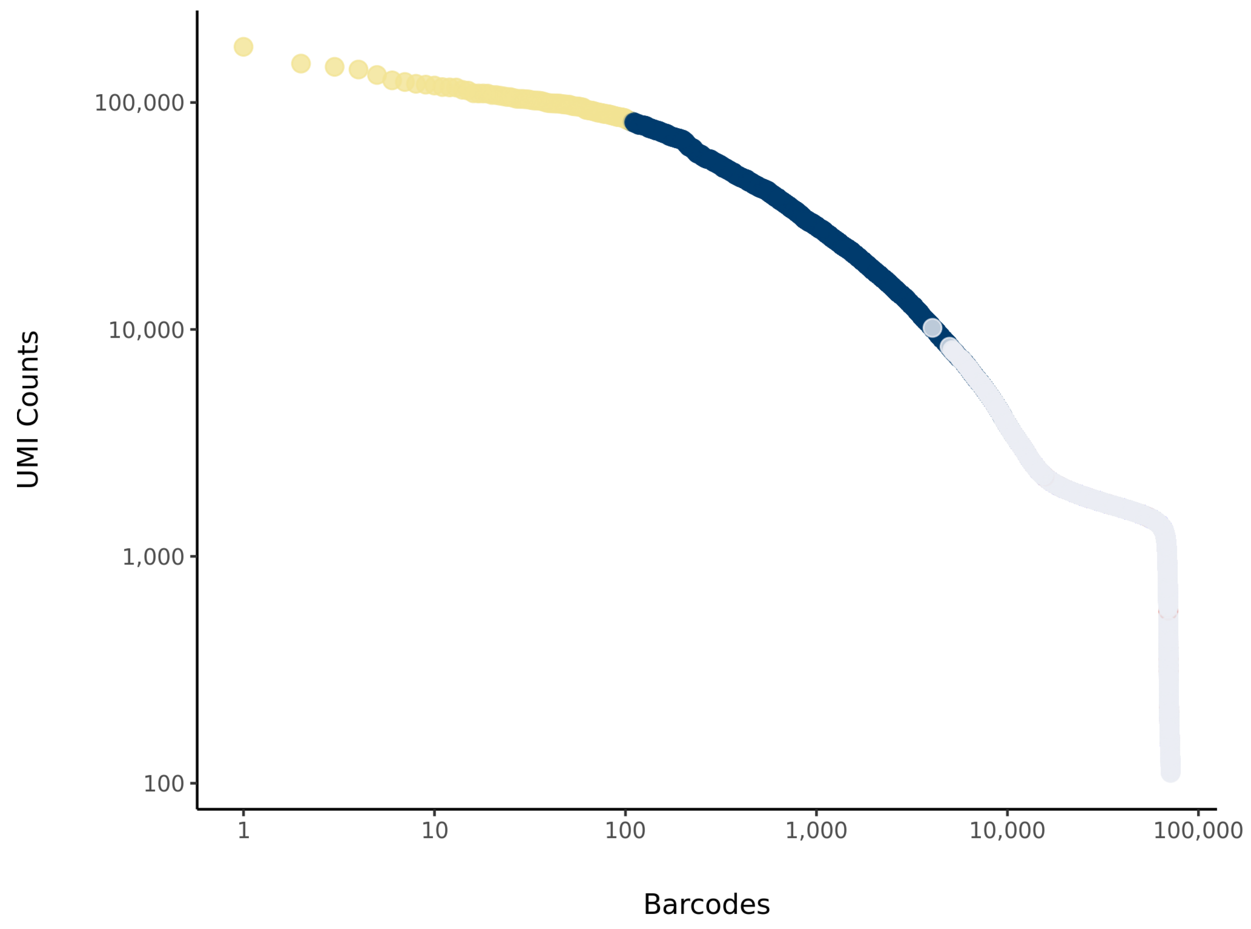

sc_25 Summary

Processed by COPILOT

Summary | Analysis

Parameters

Iteration of Filtering	1
Mitochondrial Expression Threshold	5 %
Top High Quality Cell Filtered	1 %
Doublet Removed	Yes

Cell Stats

Estimated Number of High Quality Cell	8,246
High Quality Cell	12.34 %
Total UMI Counts in High Quality Cell	151,165,418
UMI Counts in High Quality Cell	59.19 %
Median UMI Counts per High Quality Cell	11,167
Median Genes per High Quality Cell	3,465
Total Genes Detected in High Quality Cell	25,744
Cell above Mitochondrial Expression Threshold	1.47 %
Estimated Doublet Rate in High Quality Cell	6.19 %

Sequencing Stats

Number of Reads Processed	357,488,578
Reads Pseudoaligned	94.6 %
Reads on Whitelist	97.17 %
Total UMI Counts	255,403,407
Sequencing Technology	10xv3
Species	Arabidopsis thaliana
Transcriptome	TAIR10

Sample Stats

Sample	sc_25
Name	scr-4_1
Source	Benfey lab
Genotype	scr-4 (Ws backcrossed to Col?)
Transgene	NA
Treatment	Untreated
Age	5_day
Timepoint	NA
Rep	NA
Target Cells	10,000
Date	NA
Seq Run	Shahan_6177

UMI Counts Histogram

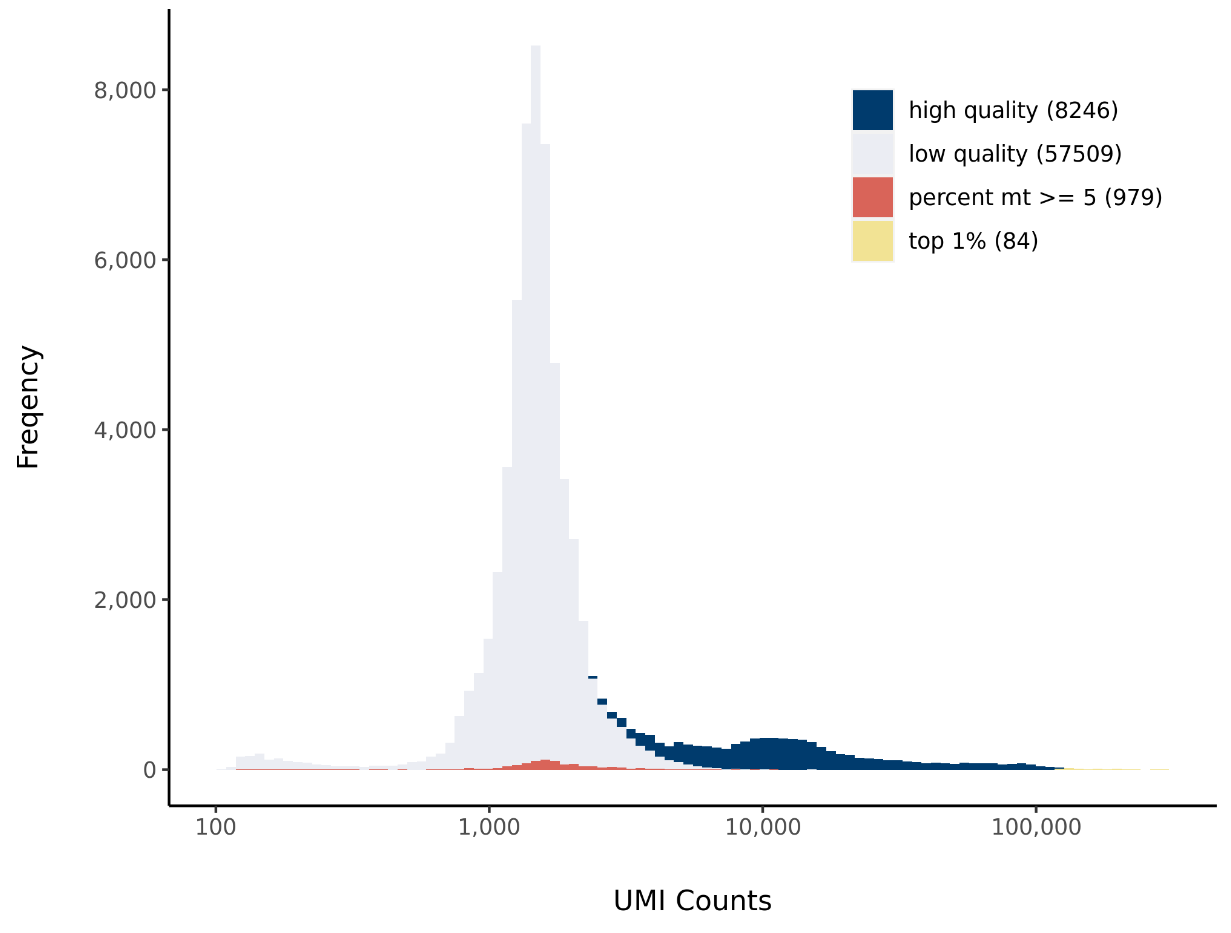

Number of Genes Histogram

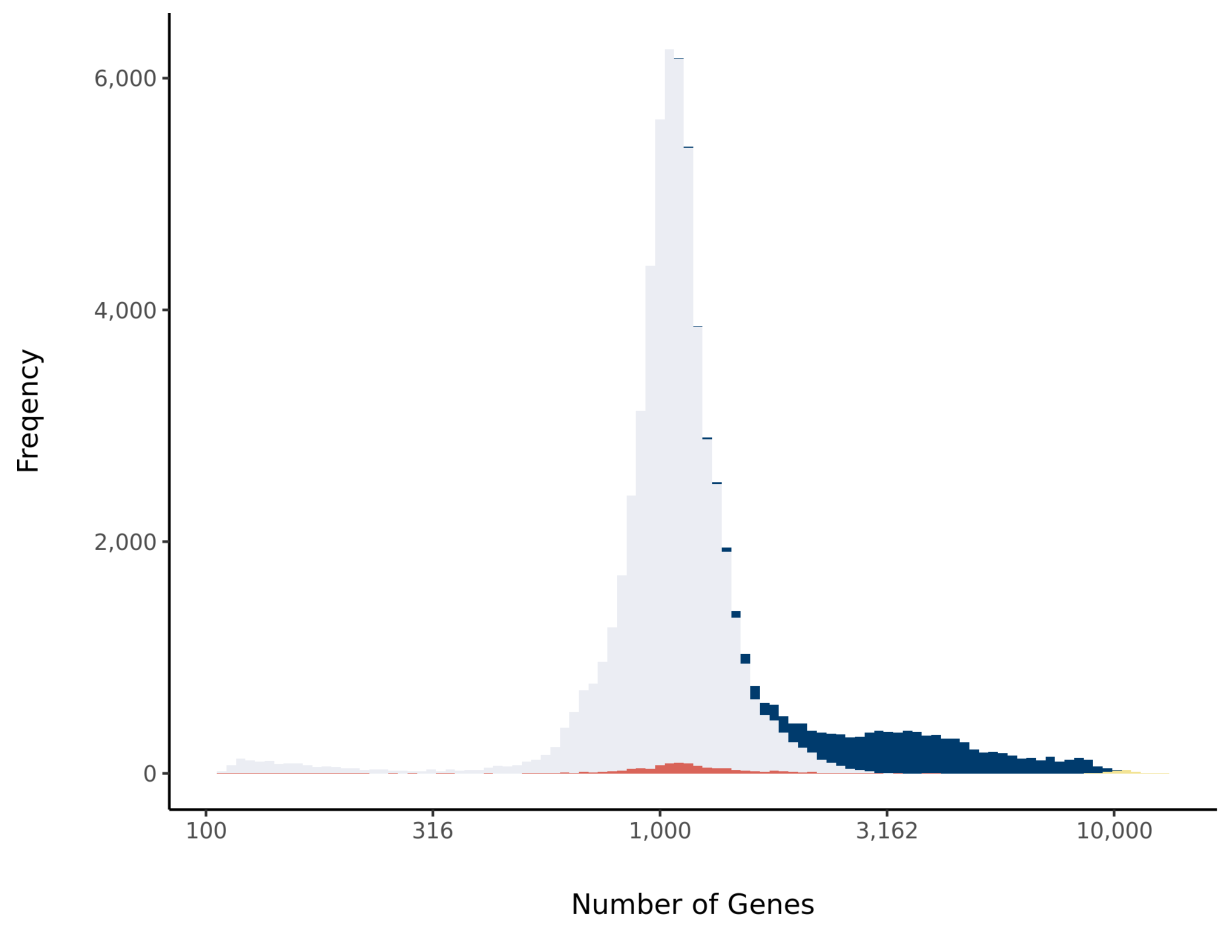

Barcode Rank Plot

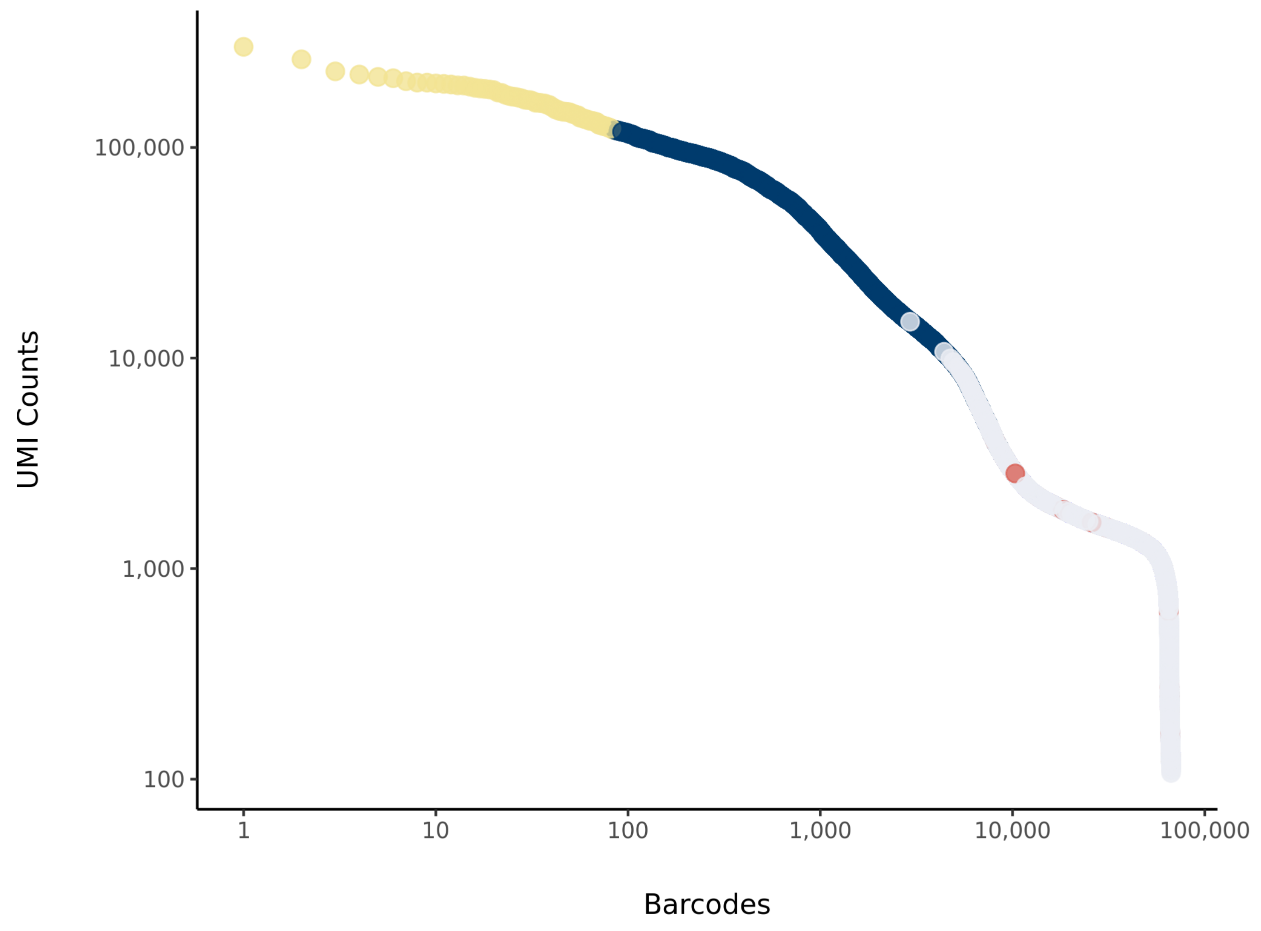

sc_30 Summary

Processed by COPILOT

Summary Analysis

Parameters

Iteration of Filtering	1
Mitochondrial Expression Threshold	5 %
Top High Quality Cell Filtered	1 %
Doublet Removed	Yes

Cell Stats

Estimated Number of High Quality Cell	11,118
High Quality Cell	14.83 %
Total UMI Counts in High Quality Cell	137,632,134
UMI Counts in High Quality Cell	79.01 %
Median UMI Counts per High Quality Cell	6,495
Median Genes per High Quality Cell	2,466.5
Total Genes Detected in High Quality Cell	24,739
Cell above Mitochondrial Expression Threshold	4.71 %
Estimated Doublet Rate in High Quality Cell	8.3 %

Sequencing Stats

Number of Reads Processed	263,408,174
Reads Pseudoaligned	93.5 %
Reads on Whitelist	96.22 %
Total UMI Counts	174,187,605
Sequencing Technology	10xv3
Species	Arabidopsis thaliana
Transcriptome	TAIR10

Sample Stats

Sample	sc_30
Name	WT Col-0_RS3
Source	Benfey lab
Genotype	WT Col-0
Transgene	NA
Treatment	Untreated
Age	5_day
Timepoint	NA
Rep	NA
Target Cells	10,000
Date	NA
Seq Run	Nolan_6199 (NextSeq); Nolan_6226 (NovaSeq S4)

UMI Counts Histogram

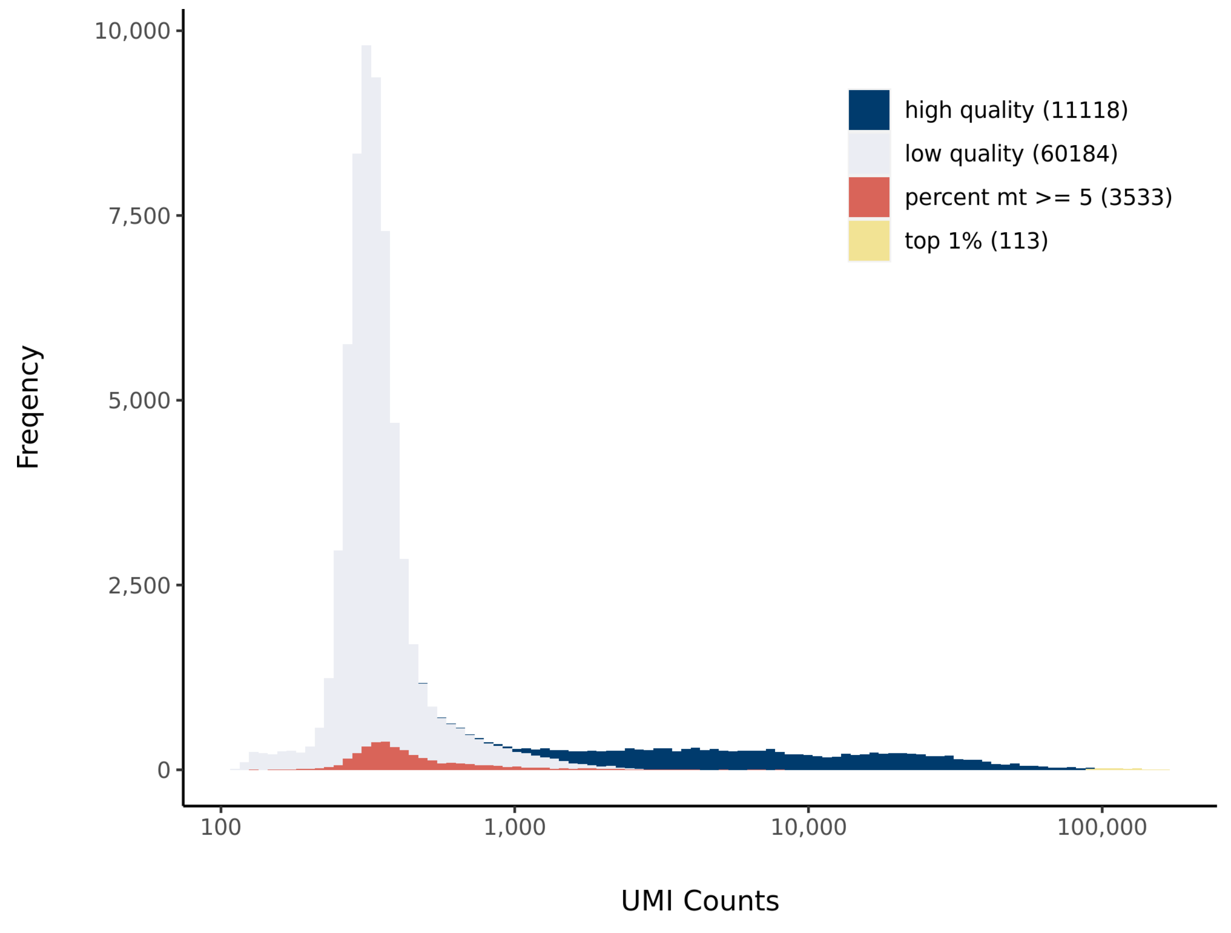

Number of Genes Histogram

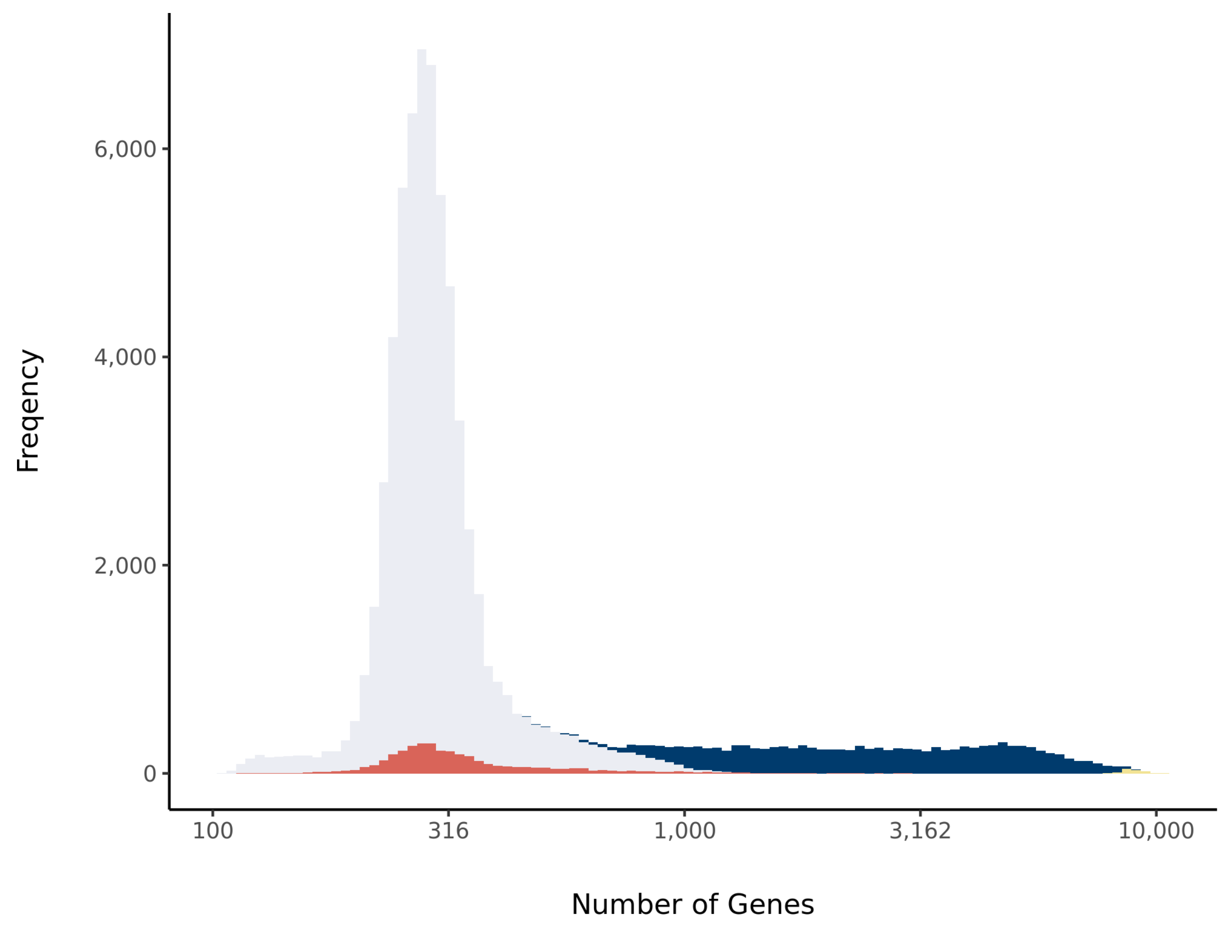

Barcode Rank Plot

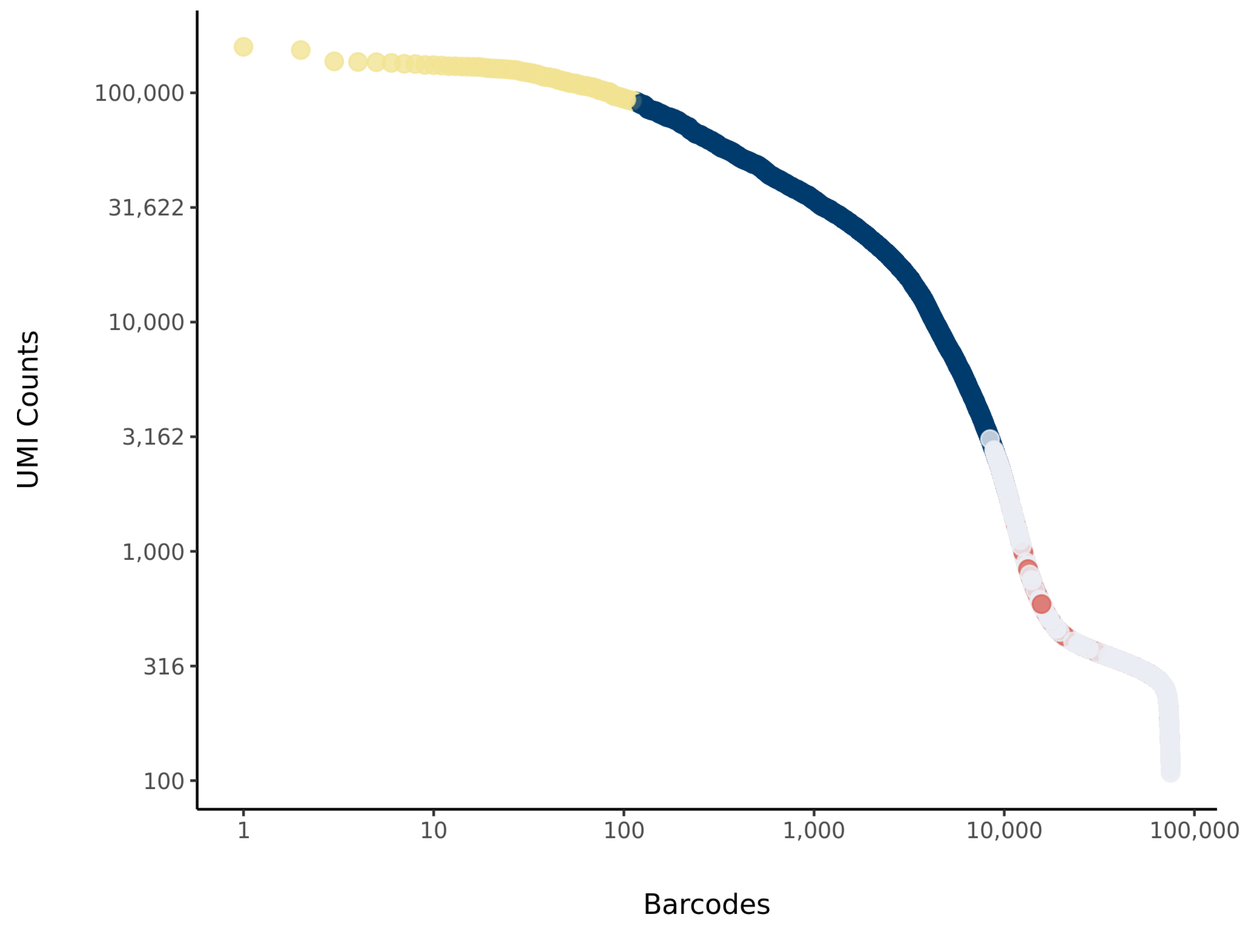

sc_31 Summary

Processed by COPILOT

Summary Analysis

Parameters

Iteration of Filtering	1
Mitochondrial Expression Threshold	5 %
Top High Quality Cell Filtered	1 %
Doublet Removed	Yes

Cell Stats

Estimated Number of High Quality Cell	9,443
High Quality Cell	12.54 %
Total UMI Counts in High Quality Cell	128,218,174
UMI Counts in High Quality Cell	76.36 %
Median UMI Counts per High Quality Cell	6,034
Median Genes per High Quality Cell	2,308
Total Genes Detected in High Quality Cell	24,826
Cell above Mitochondrial Expression Threshold	16.13 %
Estimated Doublet Rate in High Quality Cell	7.07 %

Sequencing Stats

Number of Reads Processed	266,362,743
Reads Pseudoaligned	92.5 %
Reads on Whitelist	95.91 %
Total UMI Counts	167,906,304
Sequencing Technology	10xv3
Species	Arabidopsis thaliana
Transcriptome	TAIR10

Sample Stats

Sample	sc_31
Name	WT Col-0_RS4
Source	Benfey lab
Genotype	WT Col-0
Transgene	NA
Treatment	Untreated
Age	5_day
Timepoint	NA
Rep	NA
Target Cells	10,000
Date	NA
Seq Run	Nolan_6199 (NextSeq); Nolan_6226 (NovaSeq S4)

UMI Counts Histogram

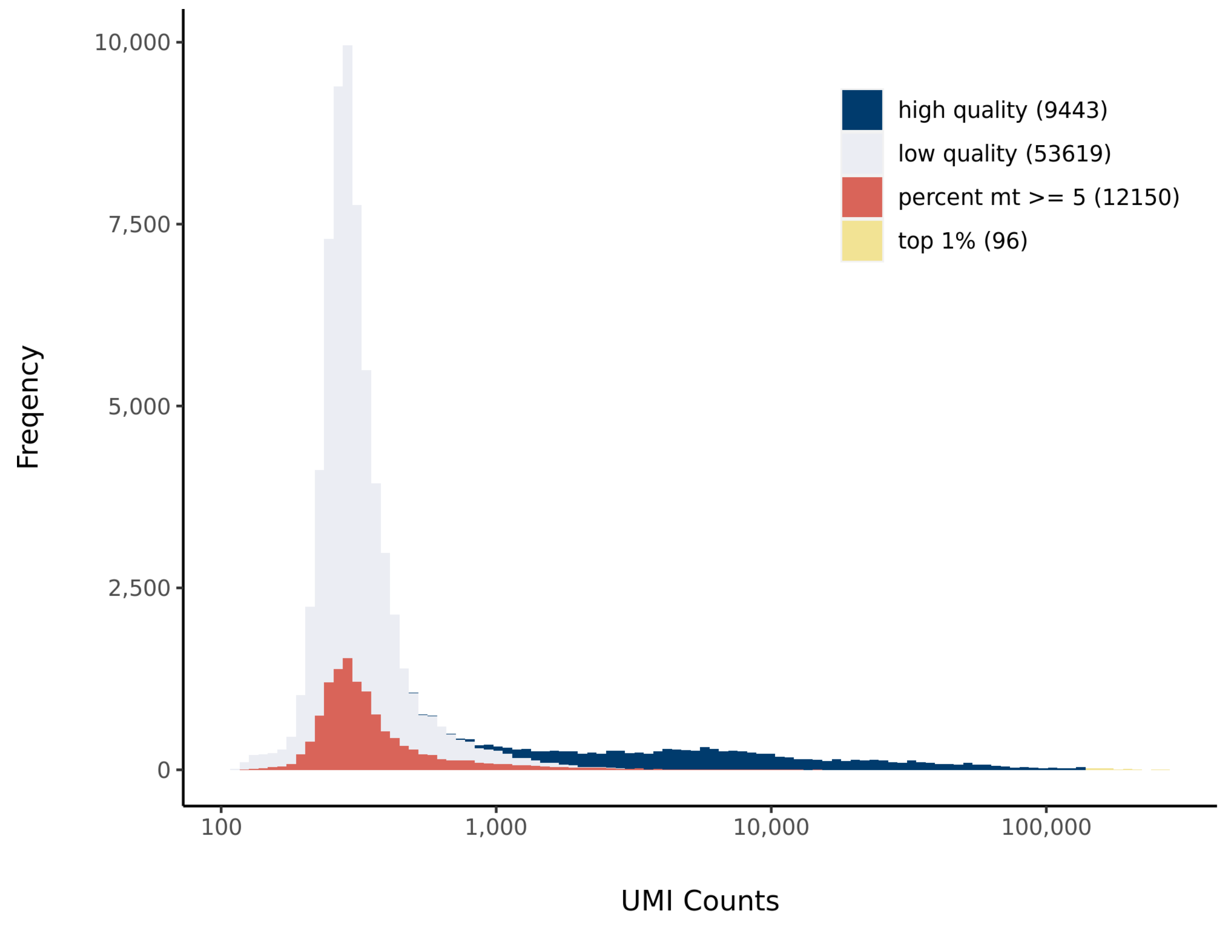

Number of Genes Histogram

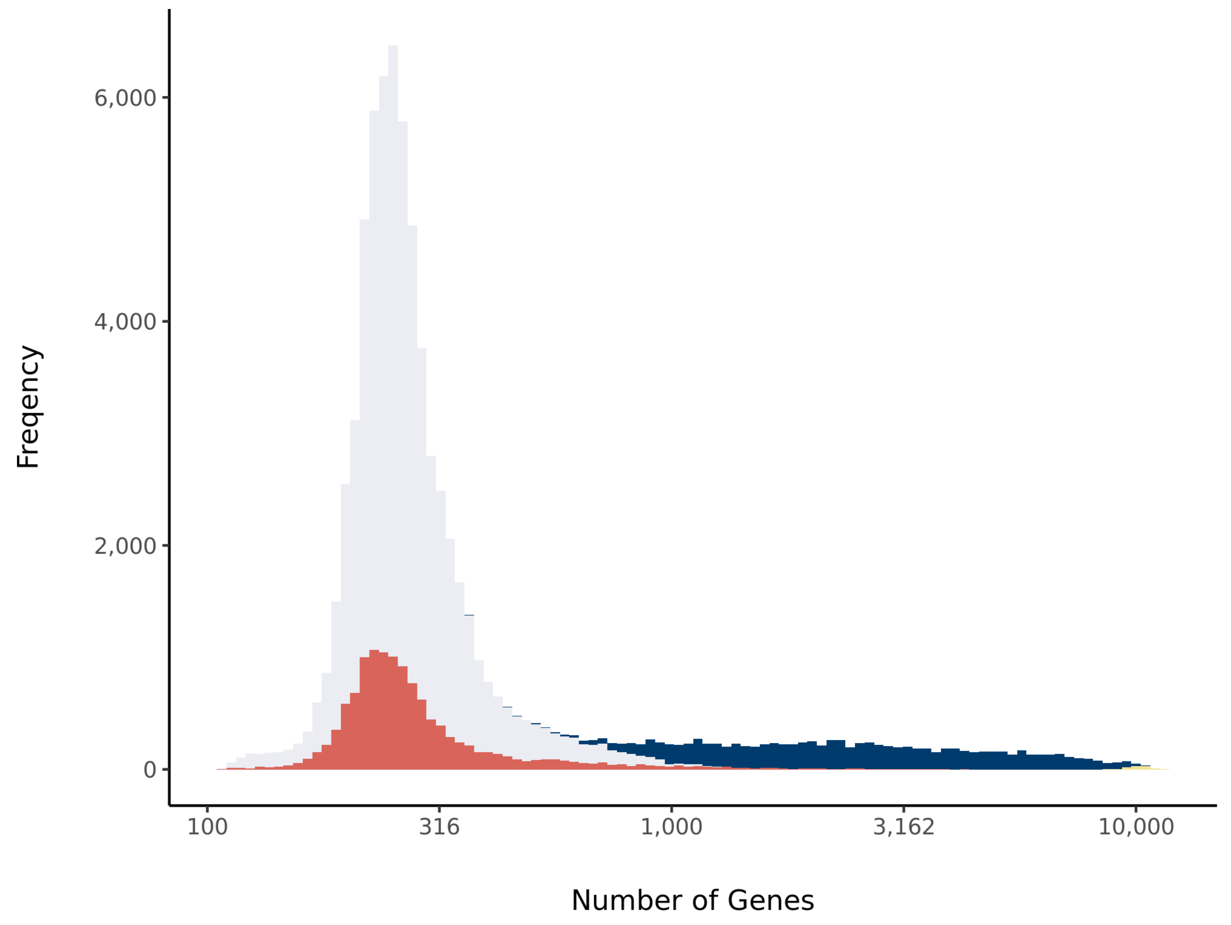

Barcode Rank Plot

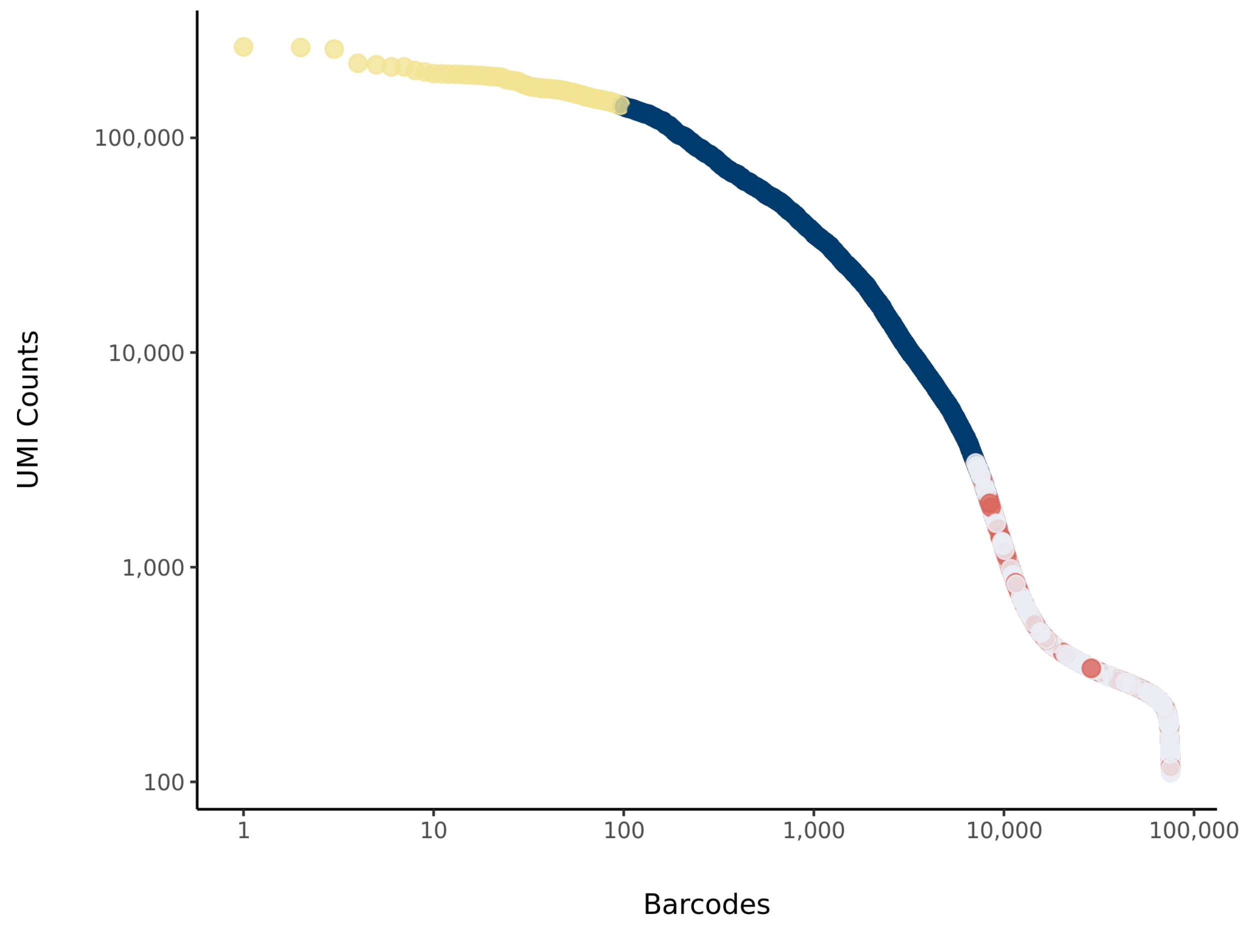

sc_36 Summary

Processed by COPILOT

Summary Analysis

Parameters

Iteration of Filtering	1
Mitochondrial Expression Threshold	5 %
Top High Quality Cell Filtered	1 %
Doublet Removed	Yes

Cell Stats

Estimated Number of High Quality Cell	6,306
High Quality Cell	7.84 %
Total UMI Counts in High Quality Cell	114,638,530
UMI Counts in High Quality Cell	76.67 %
Median UMI Counts per High Quality Cell	9,849
Median Genes per High Quality Cell	3,209
Total Genes Detected in High Quality Cell	25,147
Cell above Mitochondrial Expression Threshold	33.79 %
Estimated Doublet Rate in High Quality Cell	4.76 %

Sequencing Stats

Number of Reads Processed	280,421,300
Reads Pseudoaligned	91.3 %
Reads on Whitelist	95.85 %
Total UMI Counts	149,515,088
Sequencing Technology	10xv3
Species	Arabidopsis thaliana
Transcriptome	TAIR10

Sample Stats

Sample	sc_36
Name	scr_4_2
Source	Benfey lab
Genotype	scr-4
Transgene	NA
Treatment	Untreated
Age	5_day
Timepoint	NA
Rep	NA
Target Cells	10,000
Date	NA
Seq Run	Nolan_6199 (NextSeq); Nolan_6226 (NovaSeq S4)

UMI Counts Histogram

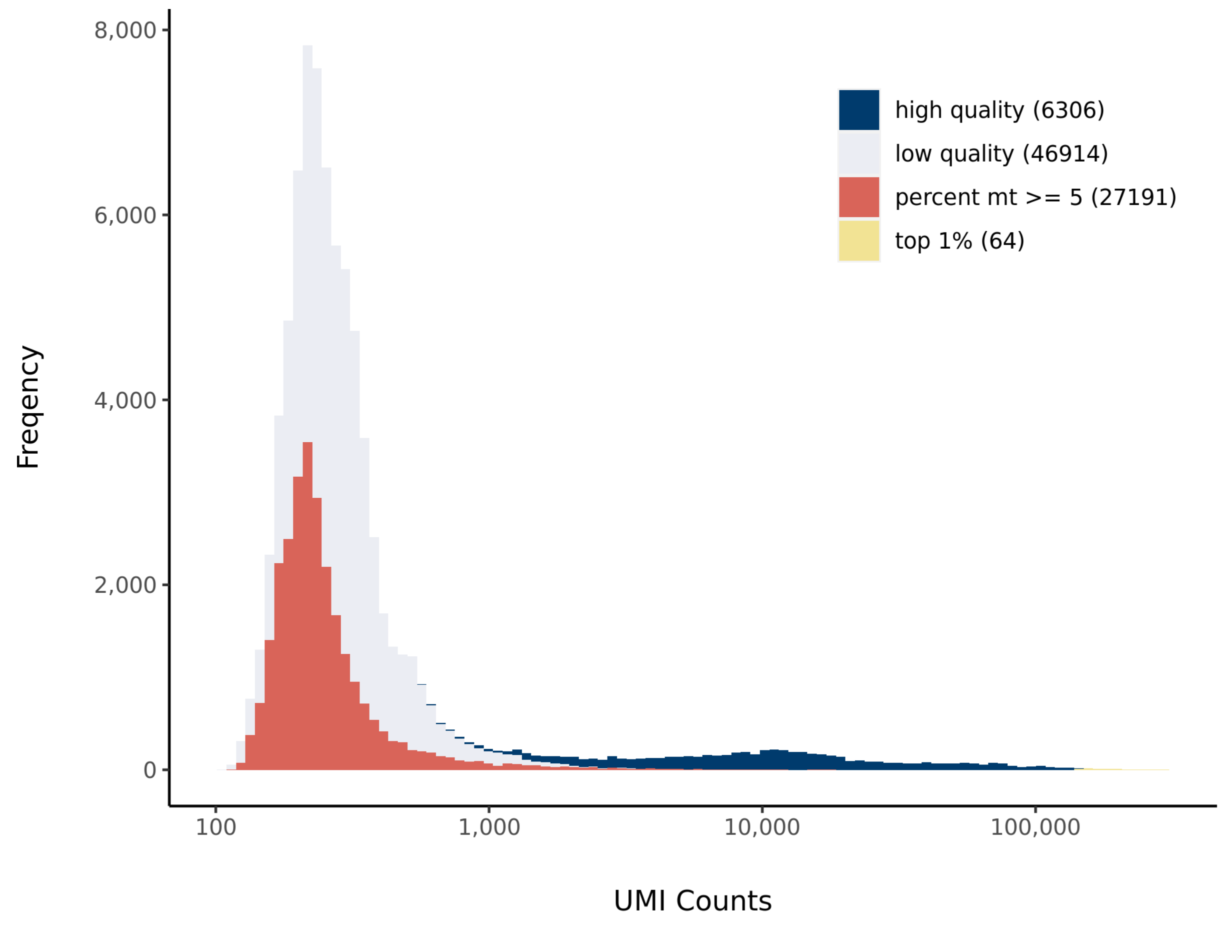

Number of Genes Histogram

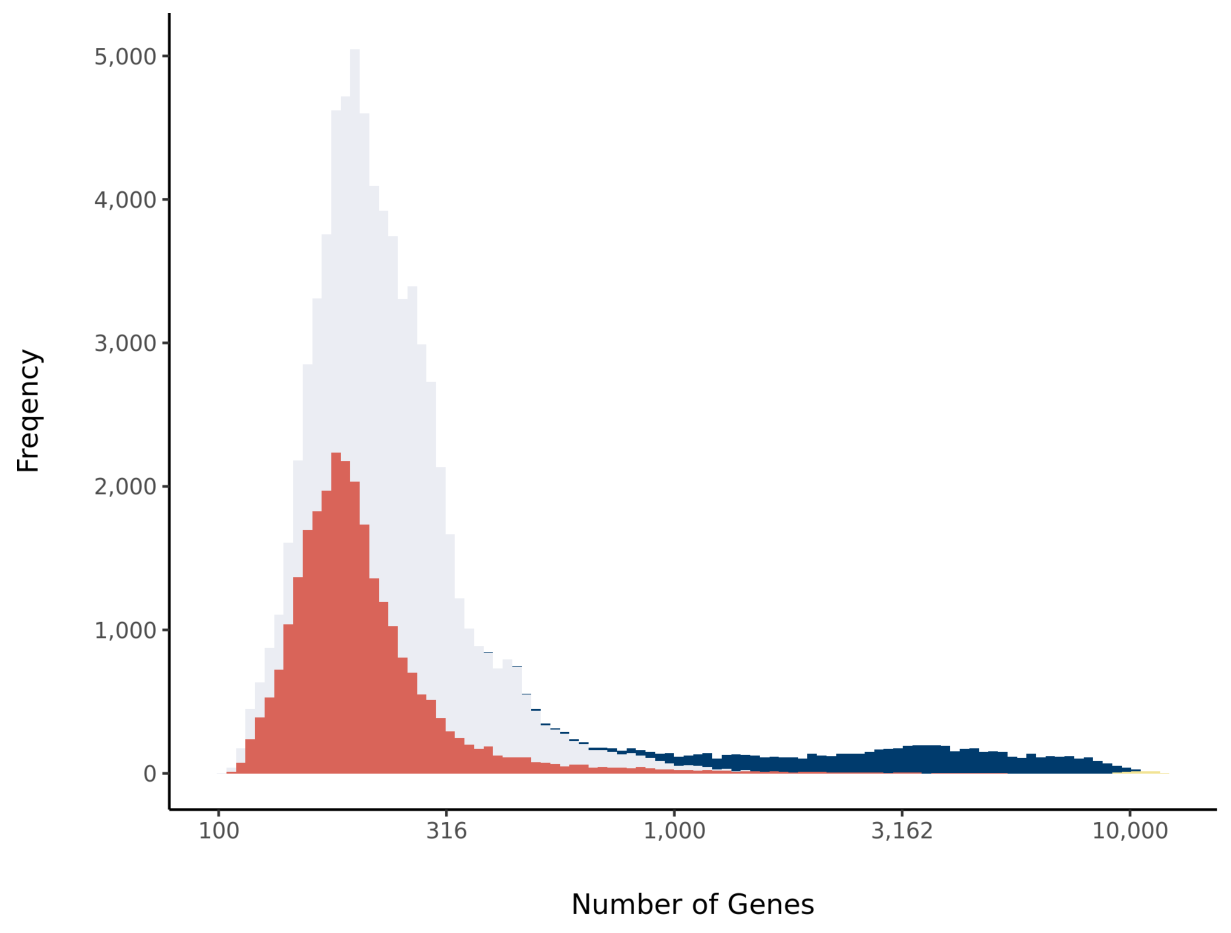

Barcode Rank Plot

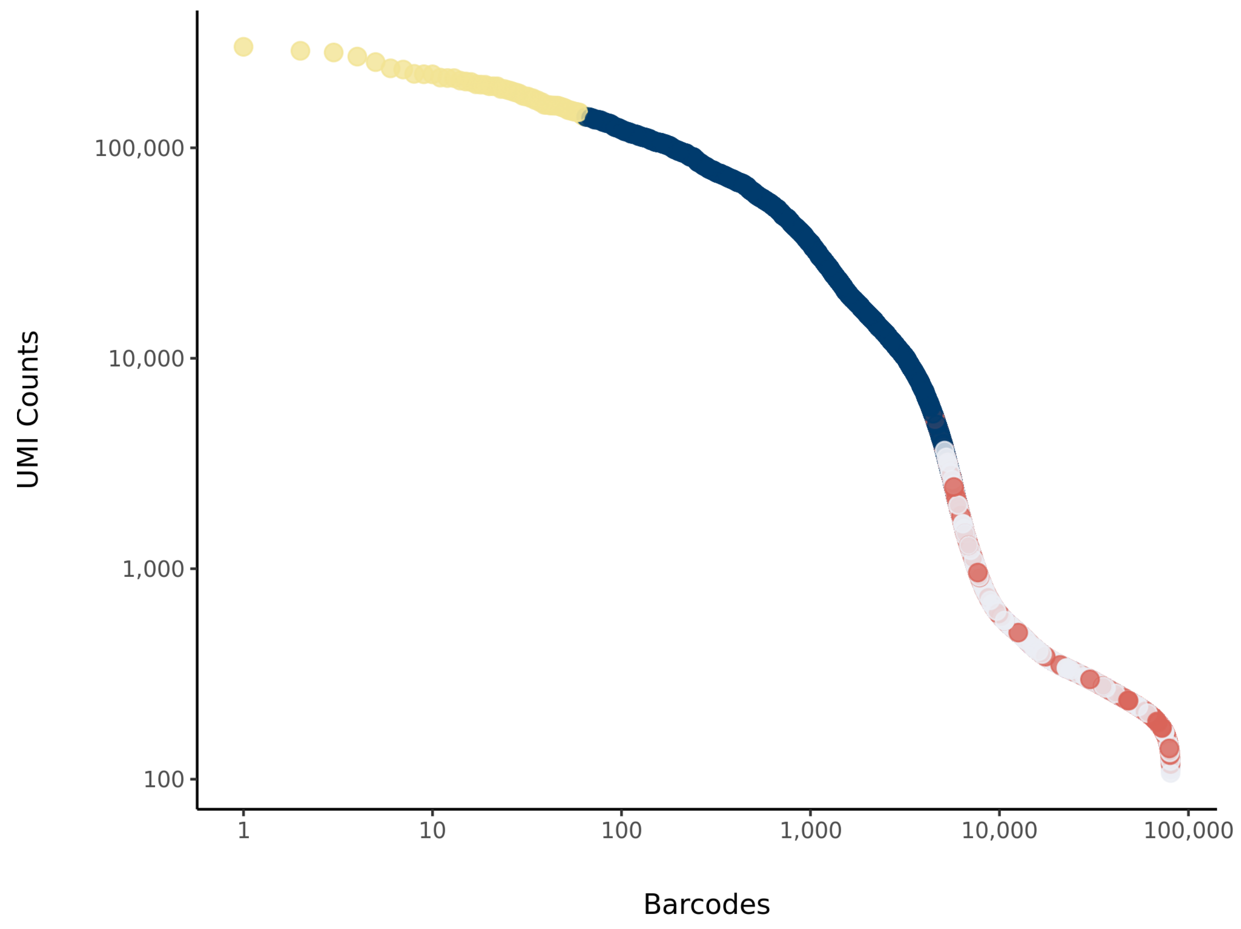

sc_37 Summary

Processed by COPILOT

Summary | Analysis

Parameters

Iteration of Filtering	1
Mitochondrial Expression Threshold	5 %
Top High Quality Cell Filtered	1 %
Doublet Removed	Yes

Cell Stats

Estimated Number of High Quality Cell	6,156
High Quality Cell	7.98 %
Total UMI Counts in High Quality Cell	106,794,708
UMI Counts in High Quality Cell	77.91 %
Median UMI Counts per High Quality Cell	5,611
Median Genes per High Quality Cell	1,984
Total Genes Detected in High Quality Cell	25,262
Cell above Mitochondrial Expression Threshold	3 %
Estimated Doublet Rate in High Quality Cell	4.65 %

Sequencing Stats

Number of Reads Processed	286,251,558
Reads Pseudoaligned	94.3 %
Reads on Whitelist	96.52 %
Total UMI Counts	137,082,512
Sequencing Technology	10xv3
Species	Arabidopsis thaliana
Transcriptome	TAIR10

Sample Stats

Sample	sc_37
Name	WT Col-0 untreated
Source	Benfey lab
Genotype	WT Col-0
Transgene	NA
Treatment	Untreated
Age	6_day
Timepoint	0
Rep	NA
Target Cells	10,000
Date	2020-02-11
Seq Run	Nolan_6199 (NextSeq); Nolan_6226 (NovaSeq S4)

UMI Counts Histogram

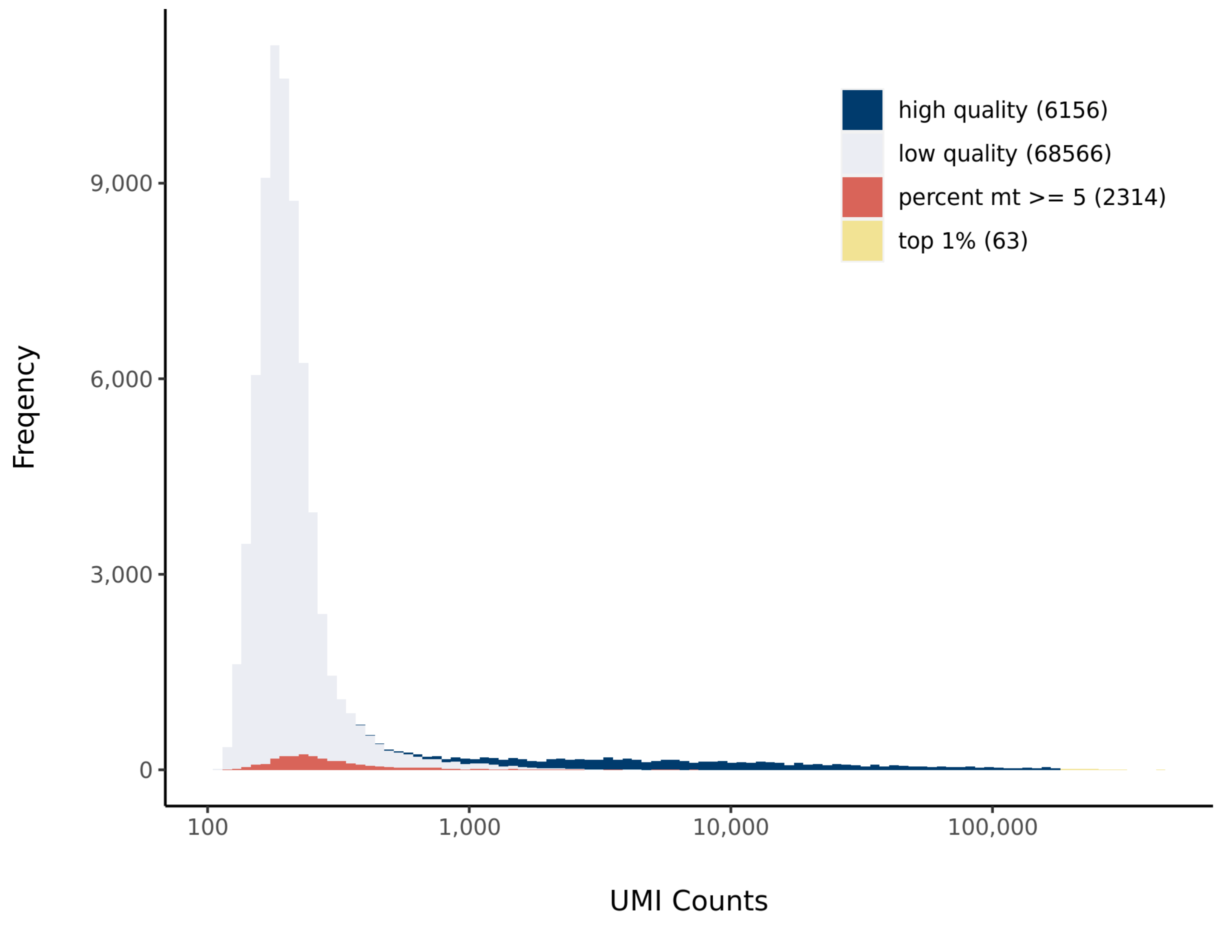

Number of Genes Histogram

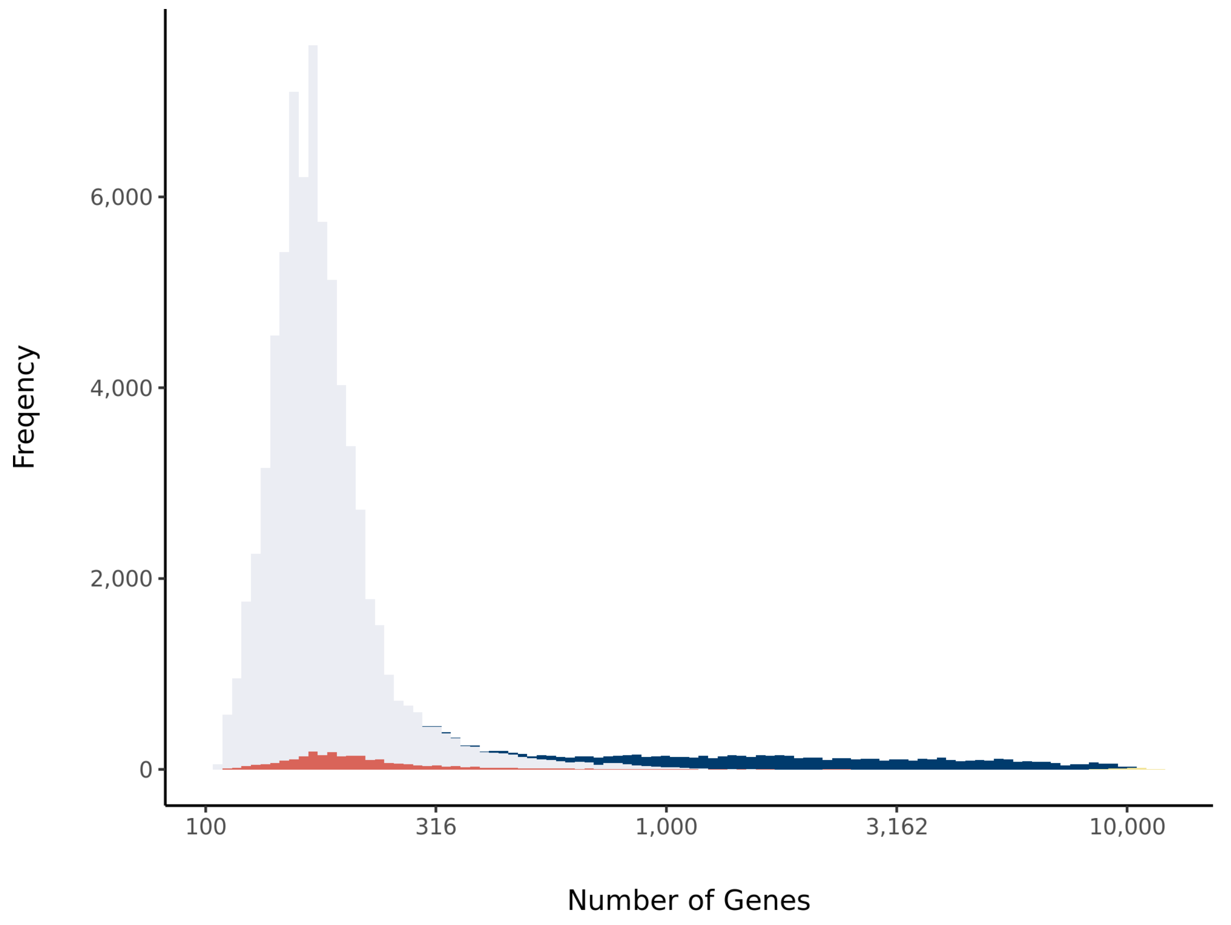

Barcode Rank Plot

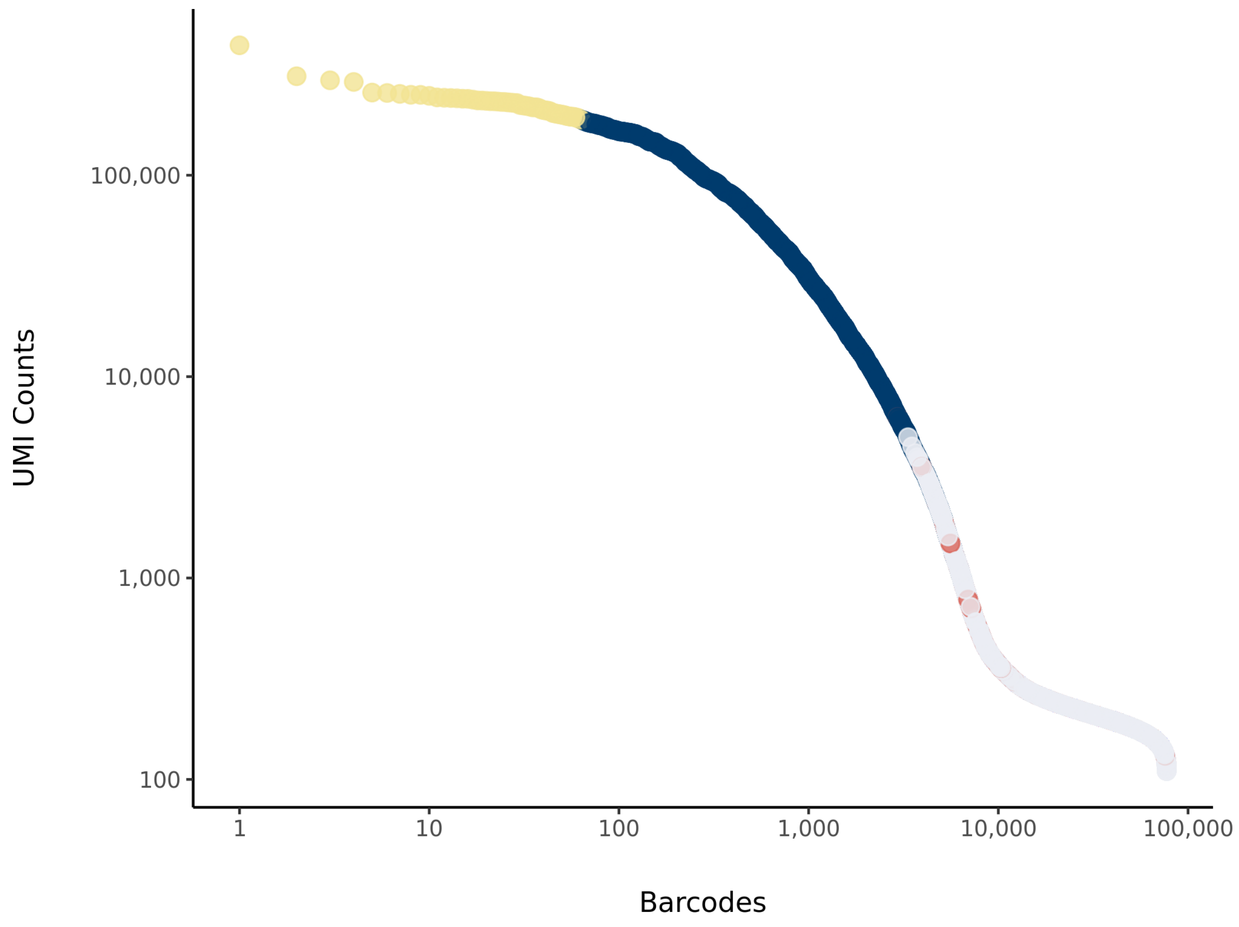

sc_40 Summary

Processed by COPILOT

Summary Analysis

Parameters

Iteration of Filtering	1
Mitochondrial Expression Threshold	5 %
Top High Quality Cell Filtered	1 %
Doublet Removed	Yes

Cell Stats

Estimated Number of High Quality Cell	7,733
High Quality Cell	30.25 %
Total UMI Counts in High Quality Cell	95,968,452
UMI Counts in High Quality Cell	84.61 %
Median UMI Counts per High Quality Cell	4,244
Median Genes per High Quality Cell	1,681
Total Genes Detected in High Quality Cell	25,693
Cell above Mitochondrial Expression Threshold	16.34 %
Estimated Doublet Rate in High Quality Cell	5.81 %

Sequencing Stats

Number of Reads Processed	276,614,617
Reads Pseudoaligned	93 %
Reads on Whitelist	96.28 %
Total UMI Counts	113,425,205
Sequencing Technology	10xv3
Species	Arabidopsis thaliana
Transcriptome	TAIR10

Sample Stats

Sample	sc_40
Name	WT Col-0 untreated
Source	Benfey lab
Genotype	WT Col-0
Transgene	NA
Treatment	Untreated
Age	6_day
Timepoint	0
Rep	NA
Target Cells	10,000
Date	2020-02-11
Seq Run	Nolan_6199 (NextSeq); Nolan_6226 (NovaSeq S4)

UMI Counts Histogram

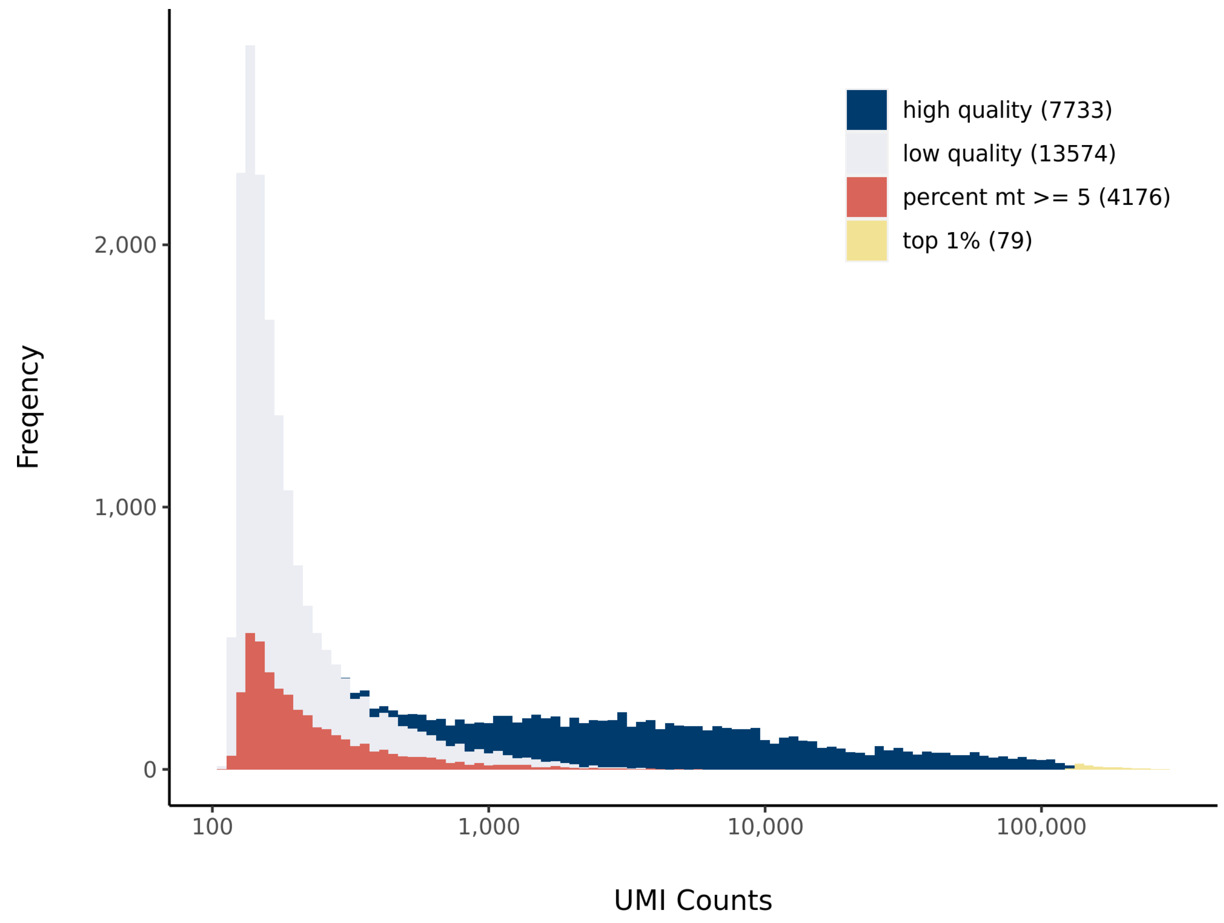

Number of Genes Histogram

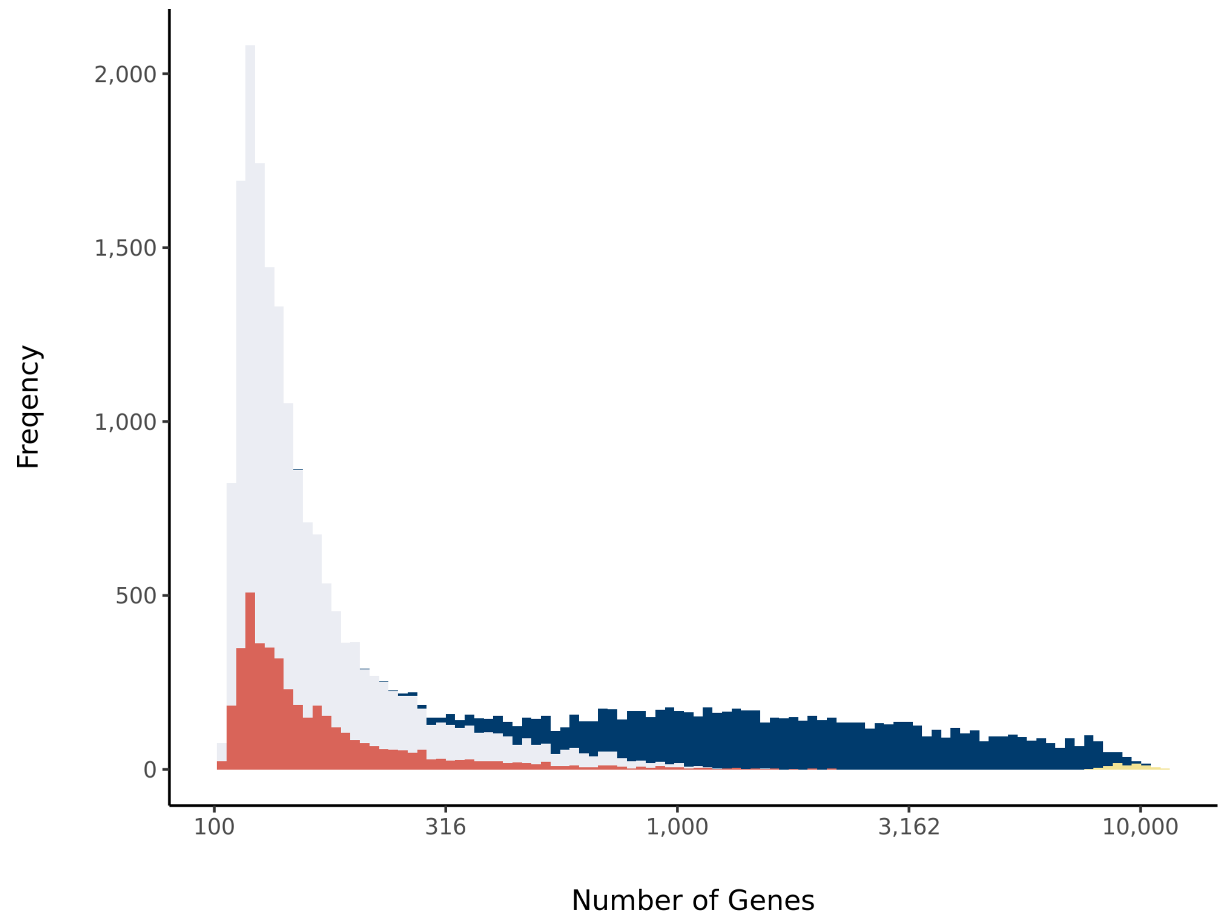

Barcode Rank Plot

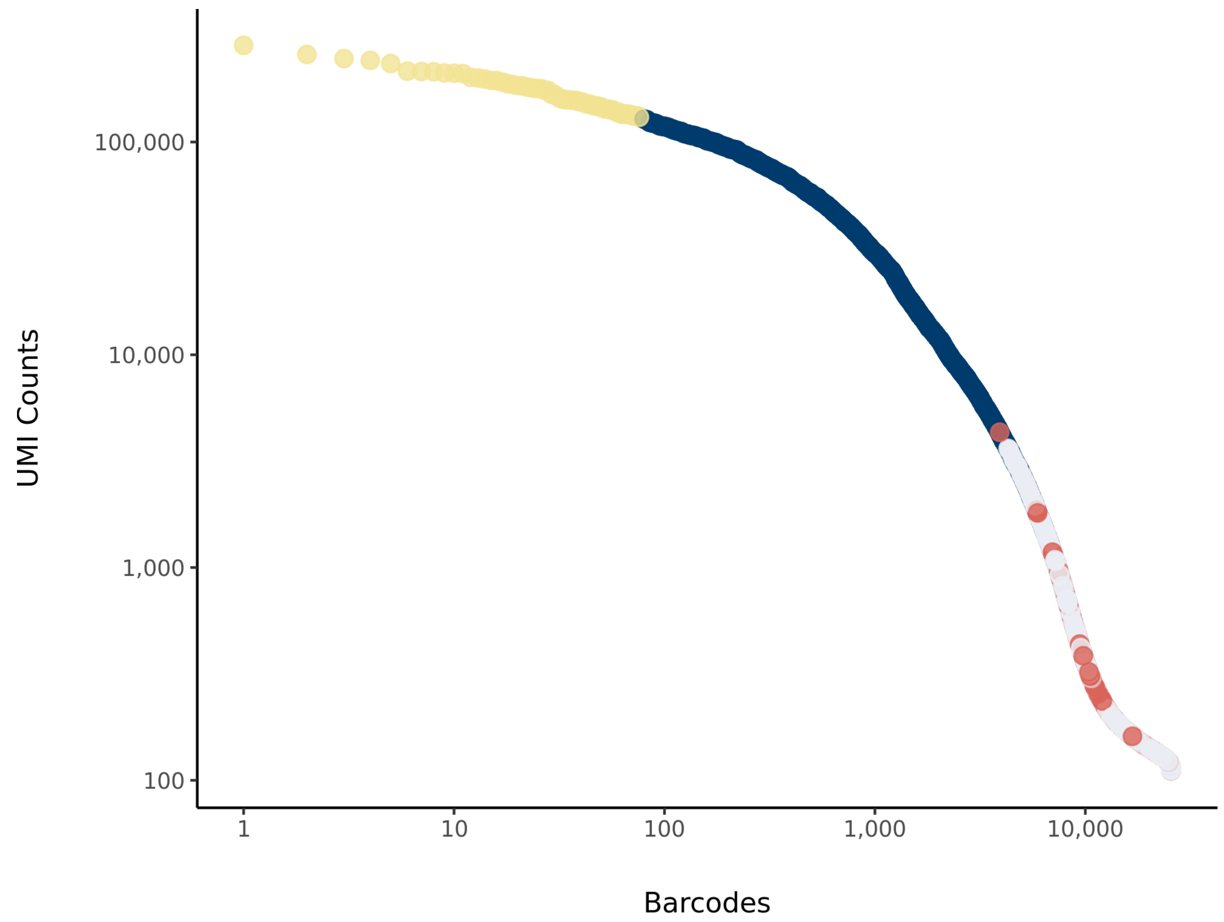

sc_51 Summary

Processed by COPILOT

Summary Analysis

Parameters

Iteration of Filtering	1
Mitochondrial Expression Threshold	5 %
Top High Quality Cell Filtered	1 %
Doublet Removed	Yes

Cell Stats

Estimated Number of High Quality Cell	6,736
High Quality Cell	9.44 %
Total UMI Counts in High Quality Cell	119,520,842
UMI Counts in High Quality Cell	77.74 %
Median UMI Counts per High Quality Cell	8,893.5
Median Genes per High Quality Cell	2,997.5
Total Genes Detected in High Quality Cell	24,754
Cell above Mitochondrial Expression Threshold	11.29 %
Estimated Doublet Rate in High Quality Cell	5.08 %

Sequencing Stats

Number of Reads Processed	246,772,773
Reads Pseudoaligned	94 %
Reads on Whitelist	96.29 %
Total UMI Counts	153,752,550
Sequencing Technology	10xv3
Species	Arabidopsis thaliana
Transcriptome	TAIR10

Sample Stats

Sample	sc_51
Name	WT Col (RS_5)
Source	Benfey lab
Genotype	WT Col-0
Transgene	NA
Treatment	Untreated
Age	5_day
Timepoint	NA
Rep	NA
Target Cells	10,000
Date	2020-02-24
Seq Run	Nolan_6226

UMI Counts Histogram

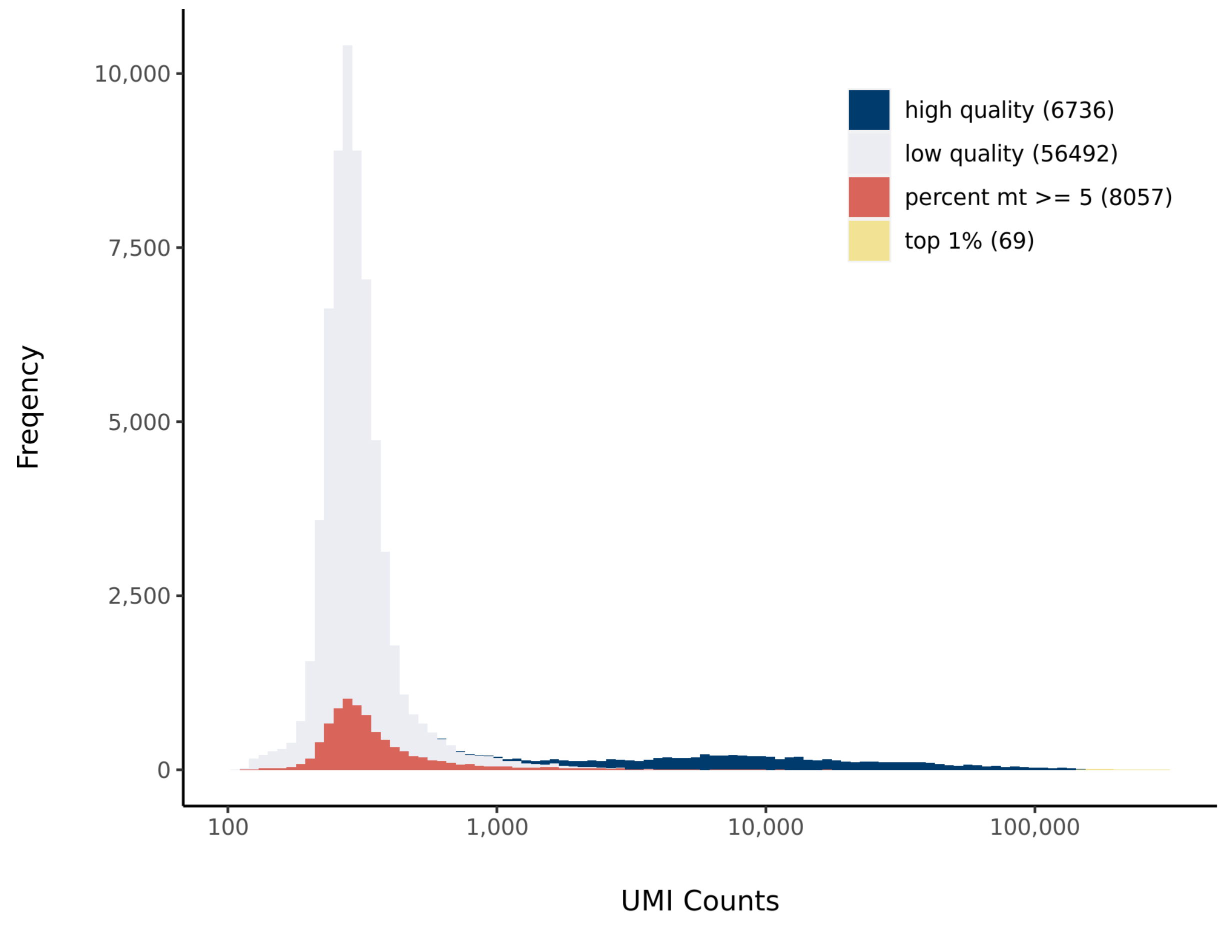

Number of Genes Histogram

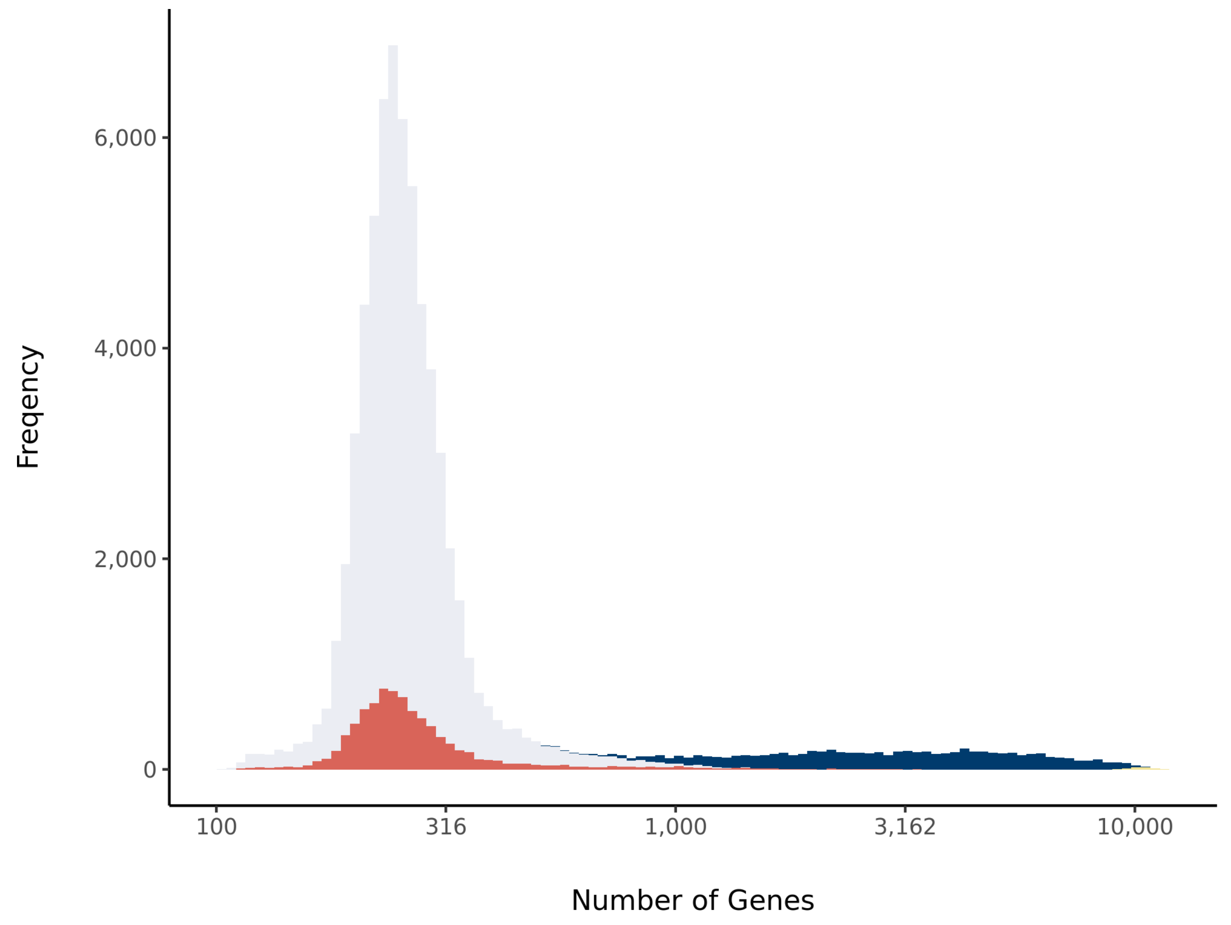

Barcode Rank Plot

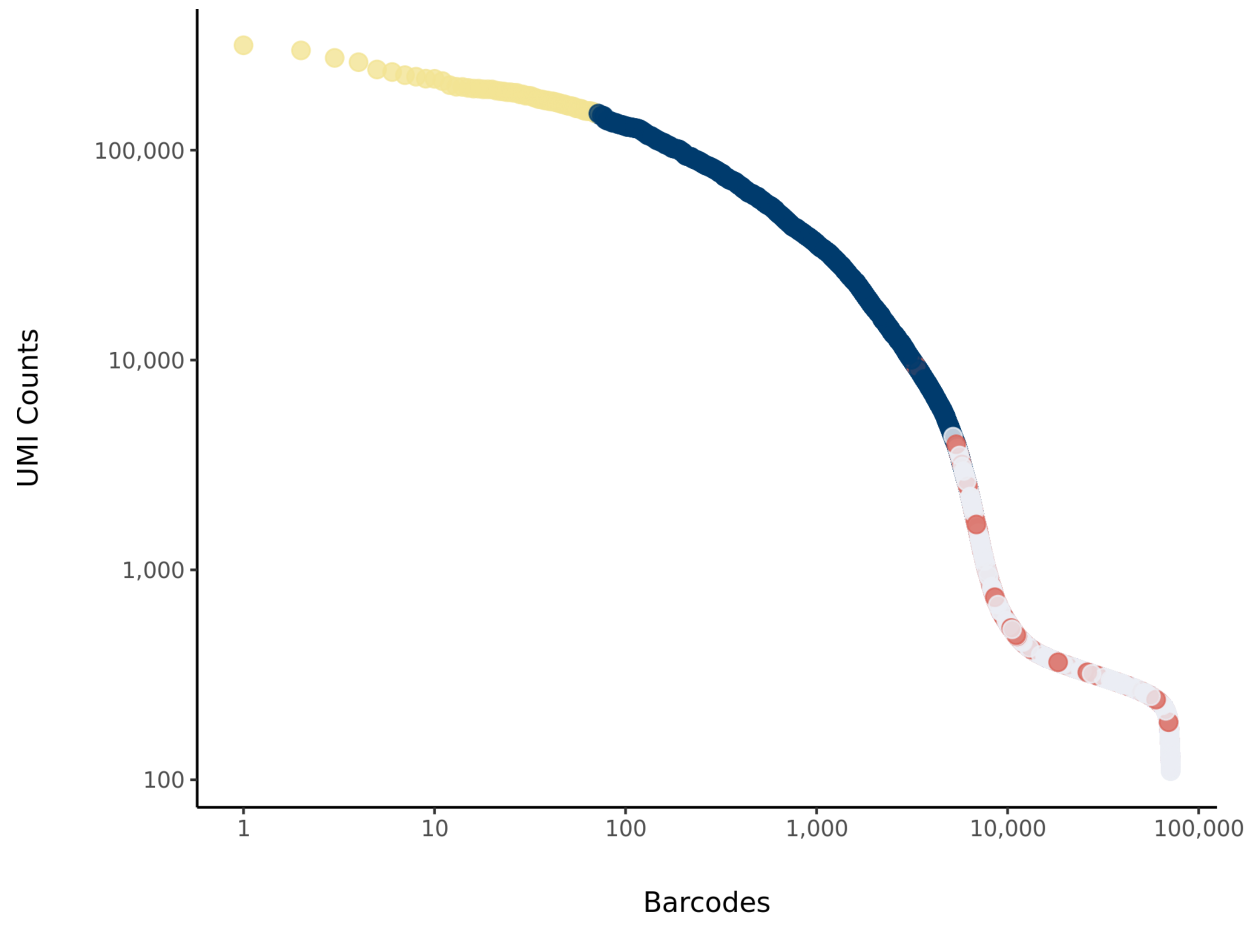

sc_52 Summary

Processed by COPILOT

Parameters

Iteration of Filtering	1
Mitochondrial Expression Threshold	5 %
Top High Quality Cell Filtered	1 %
Doublet Removed	Yes

Cell Stats

Estimated Number of High Quality Cell	6,566
High Quality Cell	17.22 %
Total UMI Counts in High Quality Cell	122,323,546
UMI Counts in High Quality Cell	87.97 %
Median UMI Counts per High Quality Cell	9,925.5
Median Genes per High Quality Cell	3,122.5
Total Genes Detected in High Quality Cell	24,747
Cell above Mitochondrial Expression Threshold	20.25 %
Estimated Doublet Rate in High Quality Cell	4.95 %

Sequencing Stats

Number of Reads Processed	239,215,784
Reads Pseudoaligned	94.1 %
Reads on Whitelist	96.37 %
Total UMI Counts	139,046,144
Sequencing Technology	10xv3
Species	Arabidopsis thaliana
Transcriptome	TAIR10

Sample Stats

Sample	sc_52
Name	shr-2
Source	Benfey lab
Genotype	shr-2
Transgene	NA
Treatment	Untreated
Age	5_day
Timepoint	NA
Rep	NA
Target Cells	10,000
Date	2020-02-24
Seq Run	Nolan_6226

UMI Counts Histogram

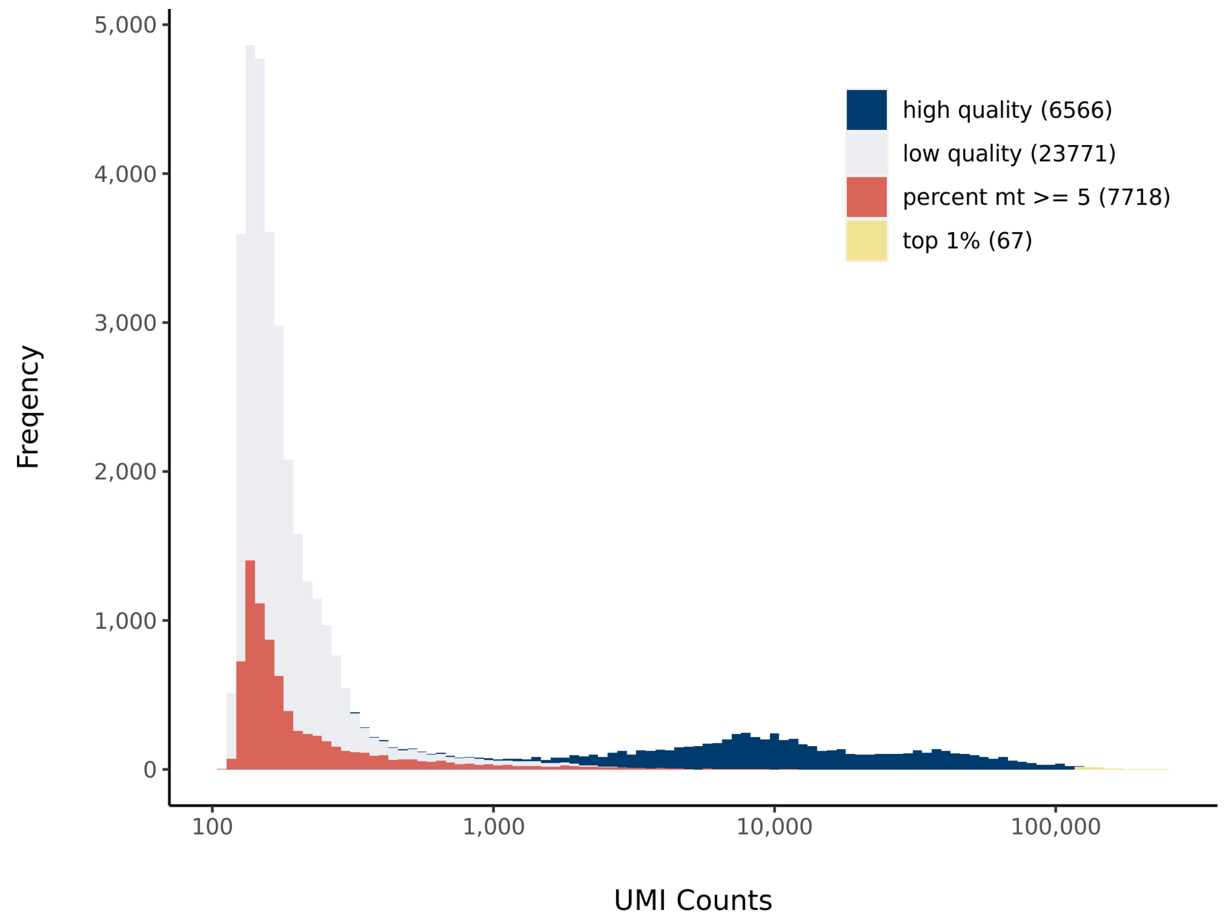

Number of Genes Histogram

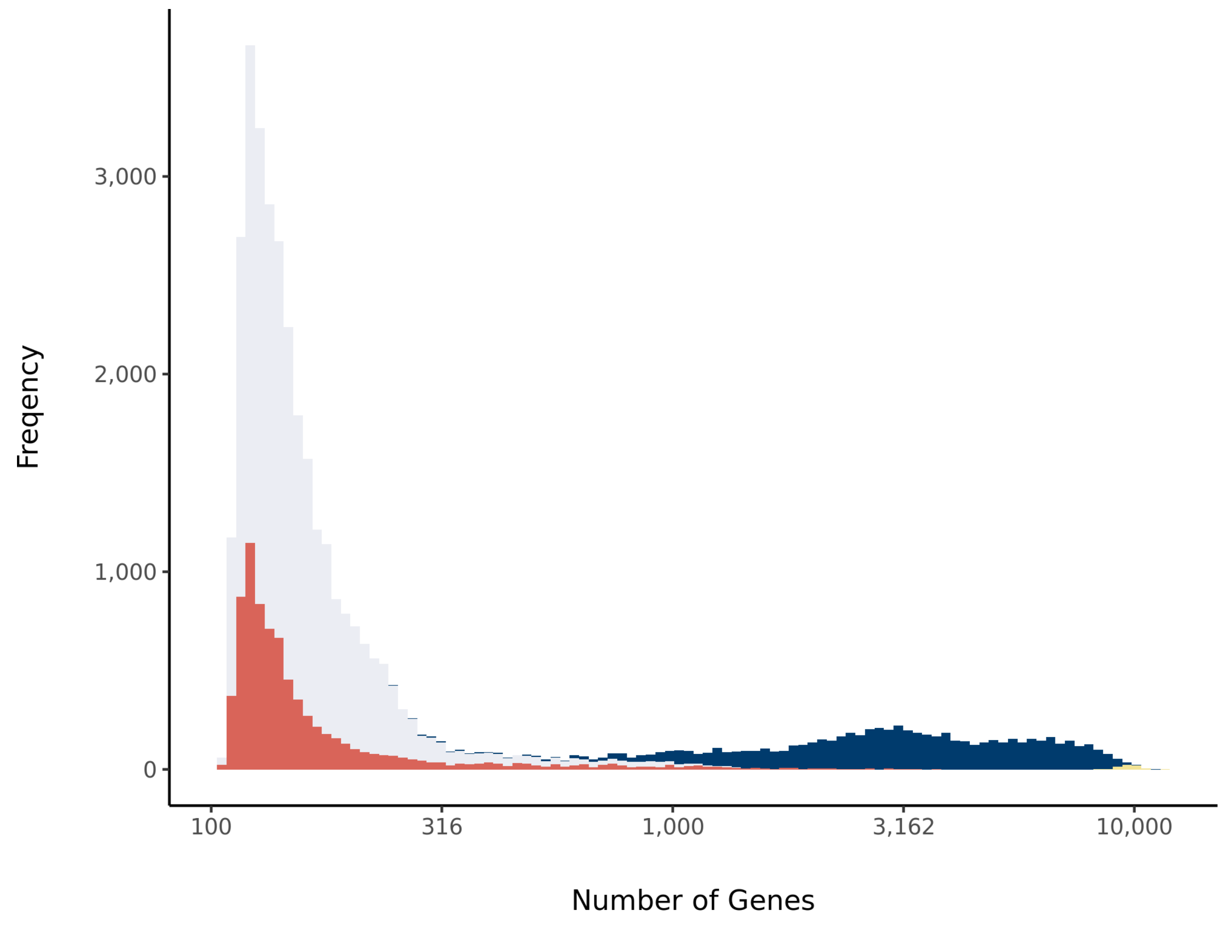

Barcode Rank Plot

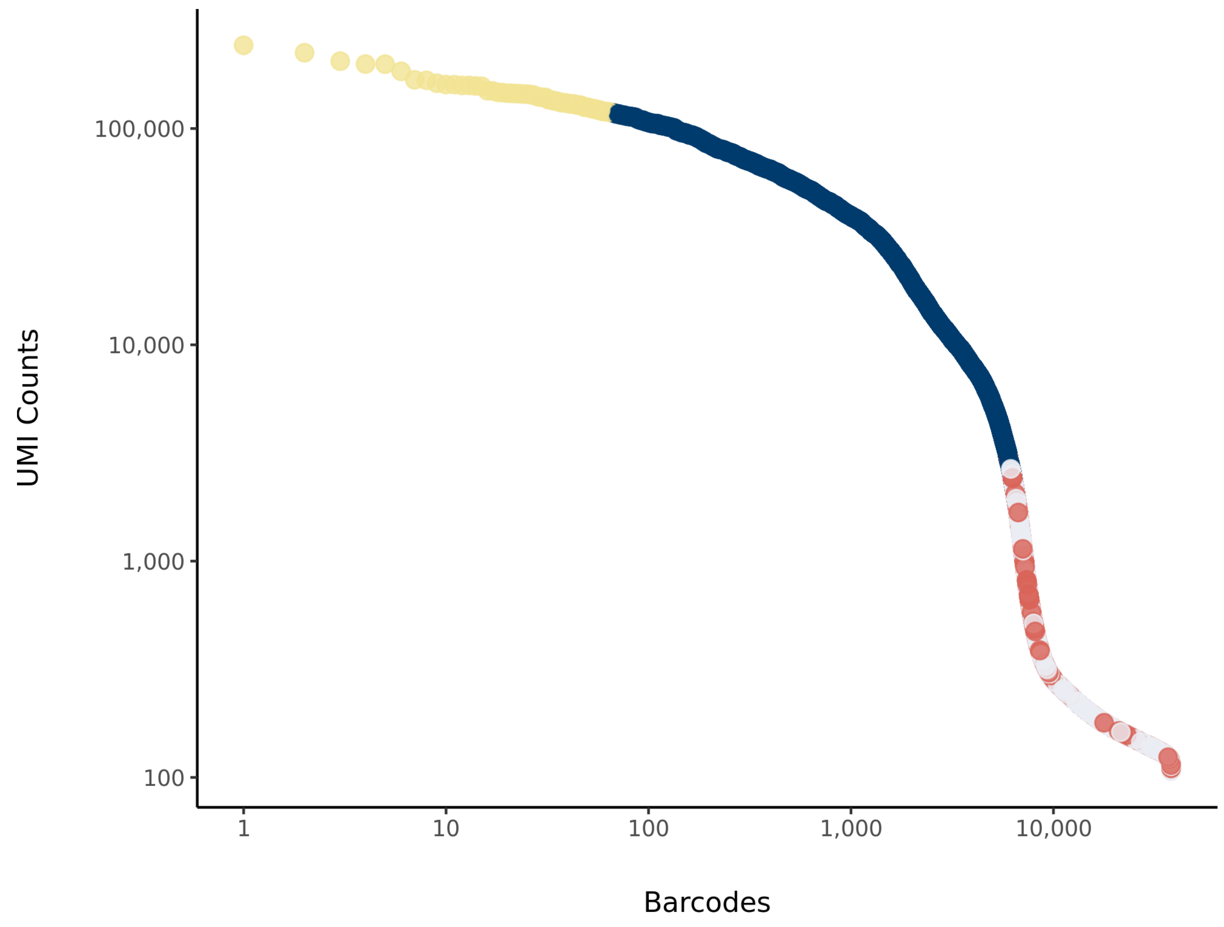

sc_53 Summary

Processed by COPILOT

Summary Analysis

Parameters

Iteration of Filtering	1
Mitochondrial Expression Threshold	5 %
Top High Quality Cell Filtered	1 %
Doublet Removed	Yes

Cell Stats

Estimated Number of High Quality Cell	8,975
High Quality Cell	28.61 %
Total UMI Counts in High Quality Cell	130,874,774
UMI Counts in High Quality Cell	89.12 %
Median UMI Counts per High Quality Cell	7,585
Median Genes per High Quality Cell	2,784
Total Genes Detected in High Quality Cell	24,809
Cell above Mitochondrial Expression Threshold	15.34 %
Estimated Doublet Rate in High Quality Cell	6.73 %

Sequencing Stats

Number of Reads Processed	244,261,968
Reads Pseudoaligned	93.9 %
Reads on Whitelist	96.36 %
Total UMI Counts	146,853,427
Sequencing Technology	10xv3
Species	Arabidopsis thaliana
Transcriptome	TAIR10

Sample Stats

Sample	sc_53
Name	shr-2
Source	Benfey lab
Genotype	shr-2
Transgene	NA
Treatment	Untreated
Age	5_day
Timepoint	NA
Rep	NA
Target Cells	10,000
Date	2020-02-24
Seq Run	Nolan_6226

UMI Counts Histogram

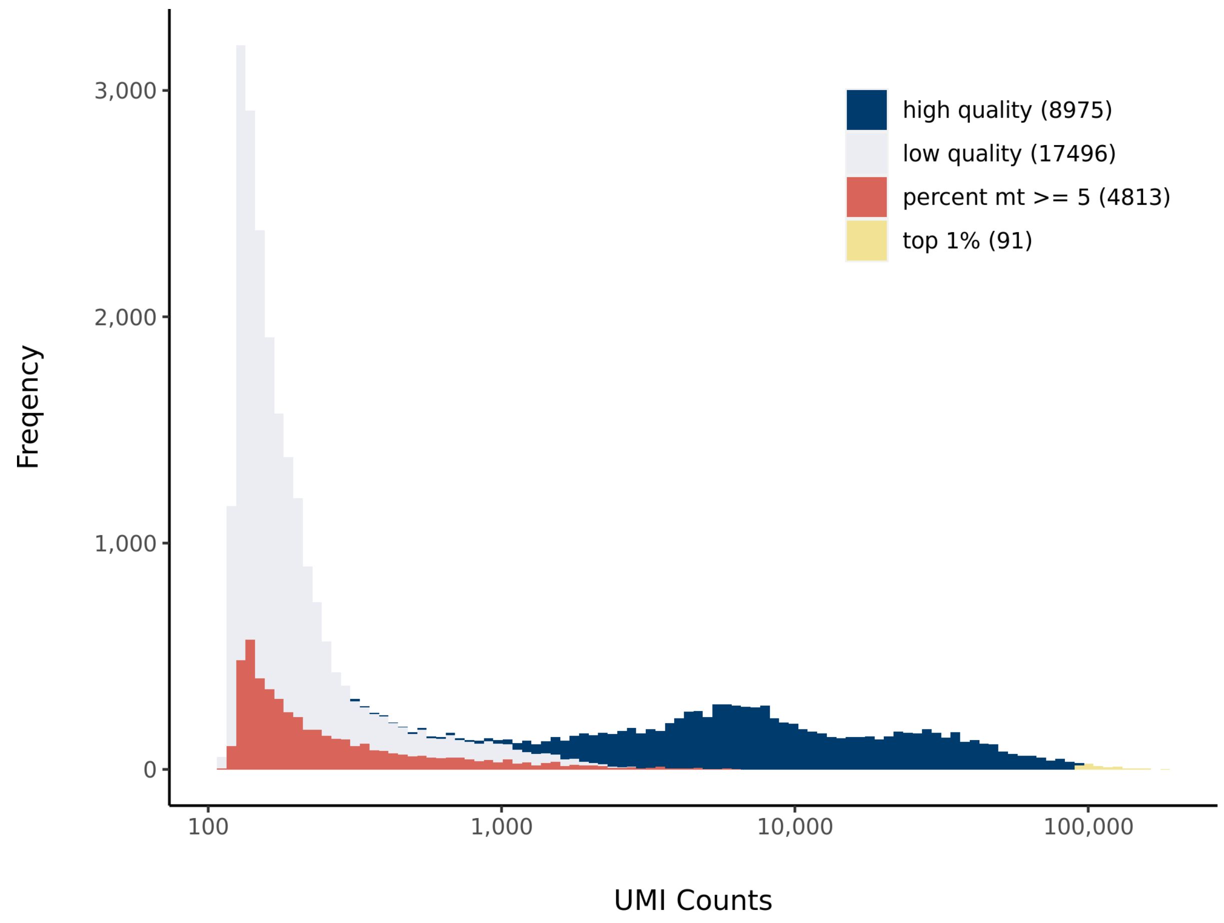

Number of Genes Histogram

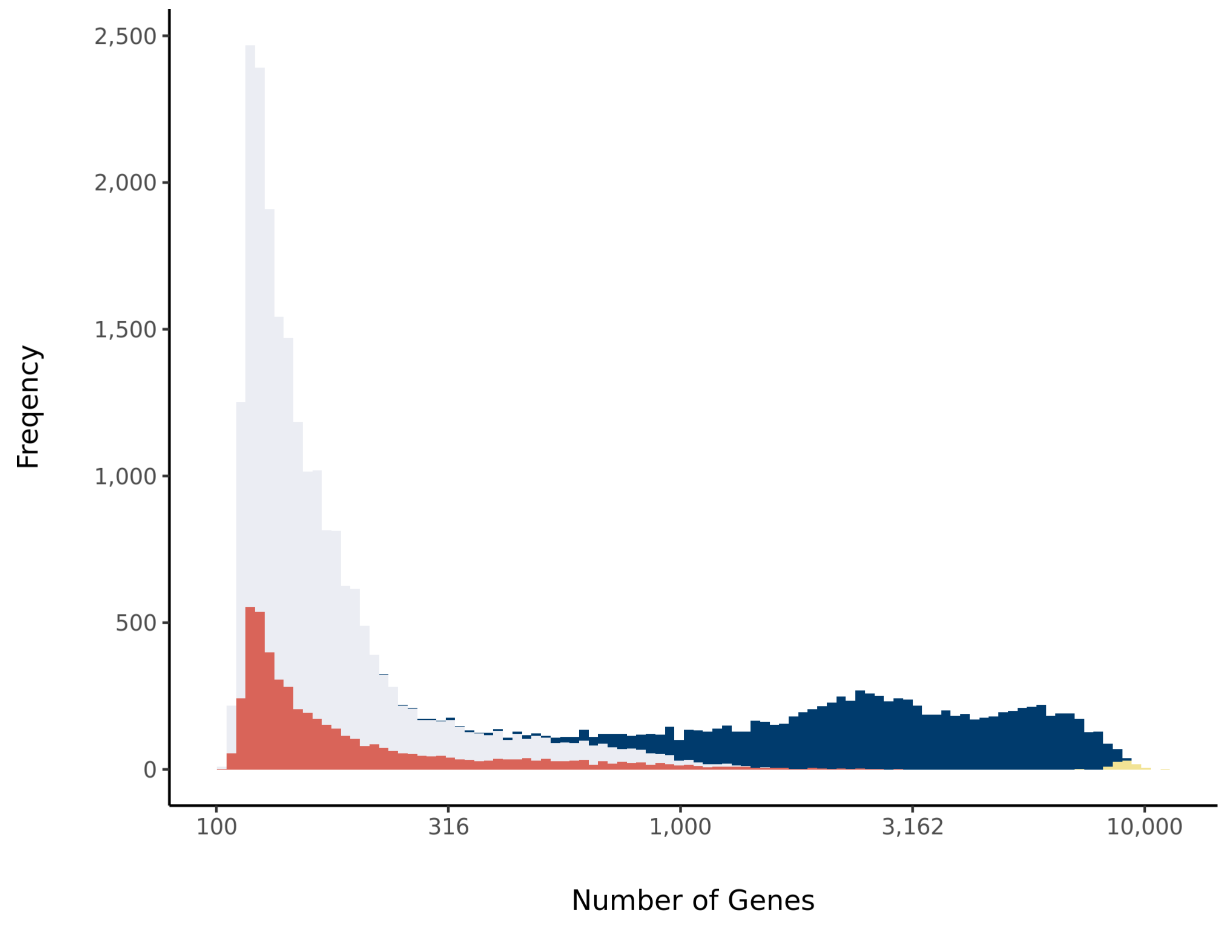

Barcode Rank Plot

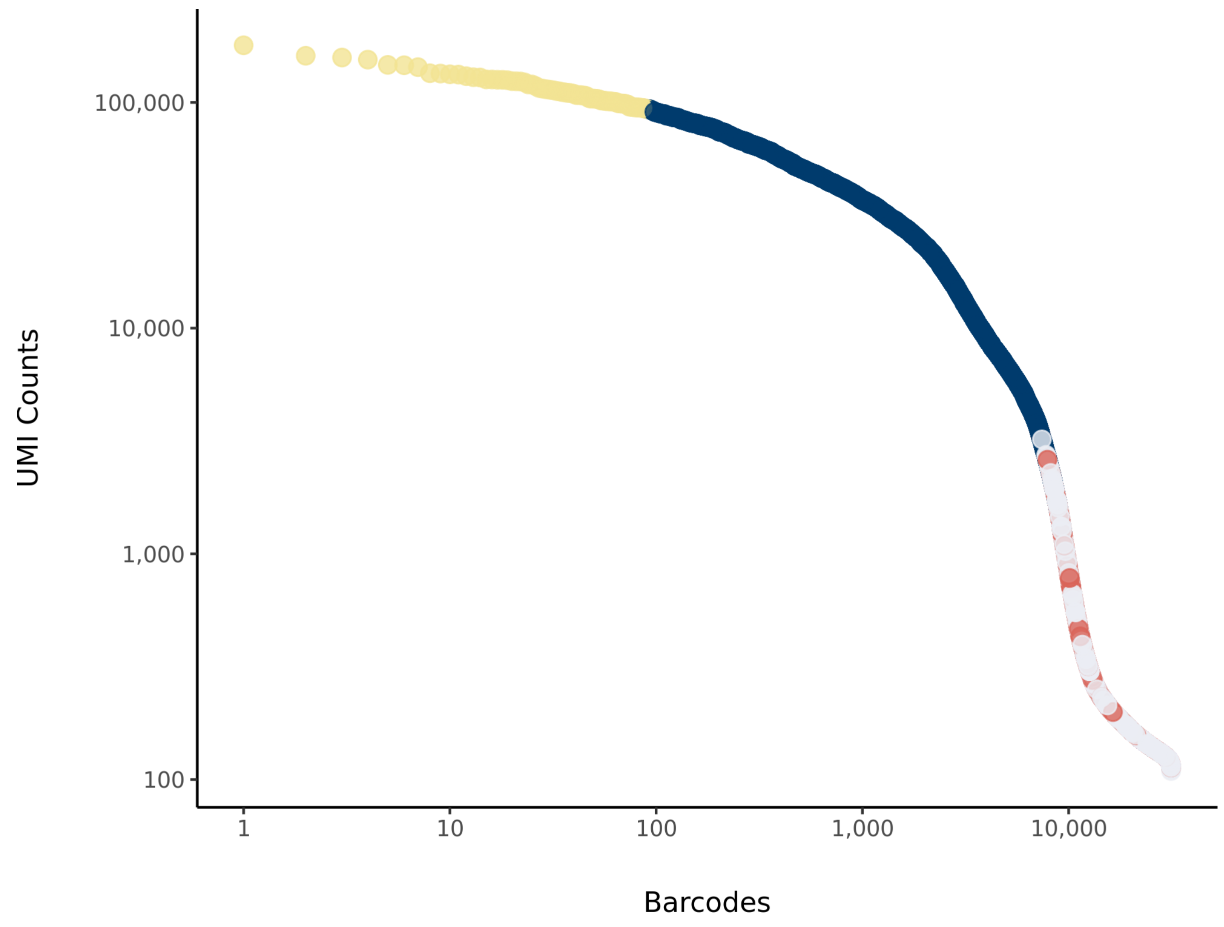

tnw1 Summary

Processed by COPILOT

Summary | Analysis

Parameters

Iteration of Filtering	1
Mitochondrial Expression Threshold	5 %
Top High Quality Cell Filtered	1 %
Doublet Removed	Yes

Cell Stats

Estimated Number of High Quality Cell	5,083
High Quality Cell	6.03 %
Total UMI Counts in High Quality Cell	108,012,603
UMI Counts in High Quality Cell	49.04 %
Median UMI Counts per High Quality Cell	12,798
Median Genes per High Quality Cell	3,426
Total Genes Detected in High Quality Cell	24,940
Cell above Mitochondrial Expression Threshold	4.84 %
Estimated Doublet Rate in High Quality Cell	3.86 %

Sequencing Stats

Number of Reads Processed	487,124,902
Reads Pseudoaligned	91.5 %
Reads on Whitelist	95.99 %
Total UMI Counts	220,259,890
Sequencing Technology	10xv3
Species	Arabidopsis thaliana
Transcriptome	TAIR10

Sample Stats

Sample	tnw1
Name	WT control1
Source	Benfey lab
Genotype	WT Col-0
Transgene	NA
Treatment	Untreated
Age	5_day
Timepoint	4
Rep	1
Target Cells	5,000
Date	2019-10-17
Seq Run	Nolan_6013

UMI Counts Histogram

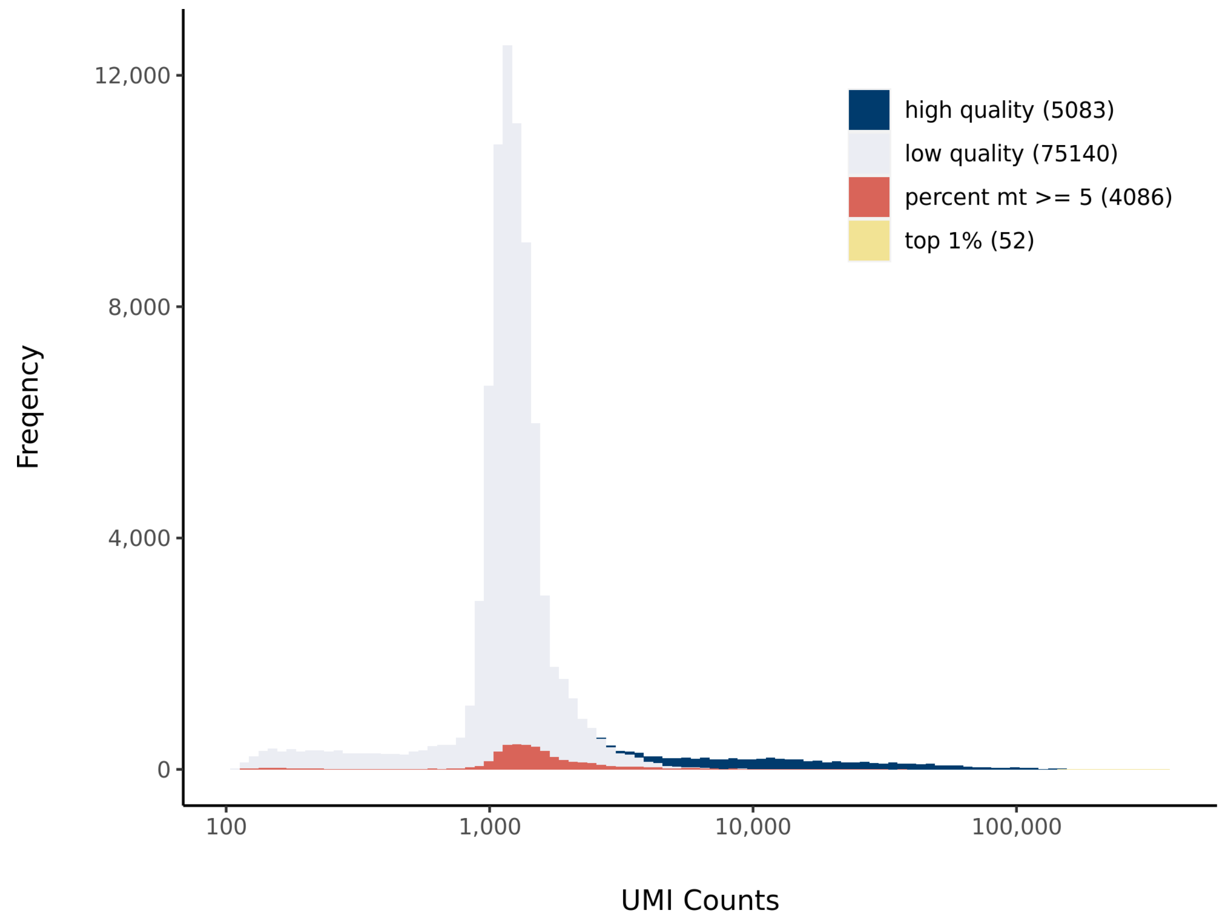

Number of Genes Histogram

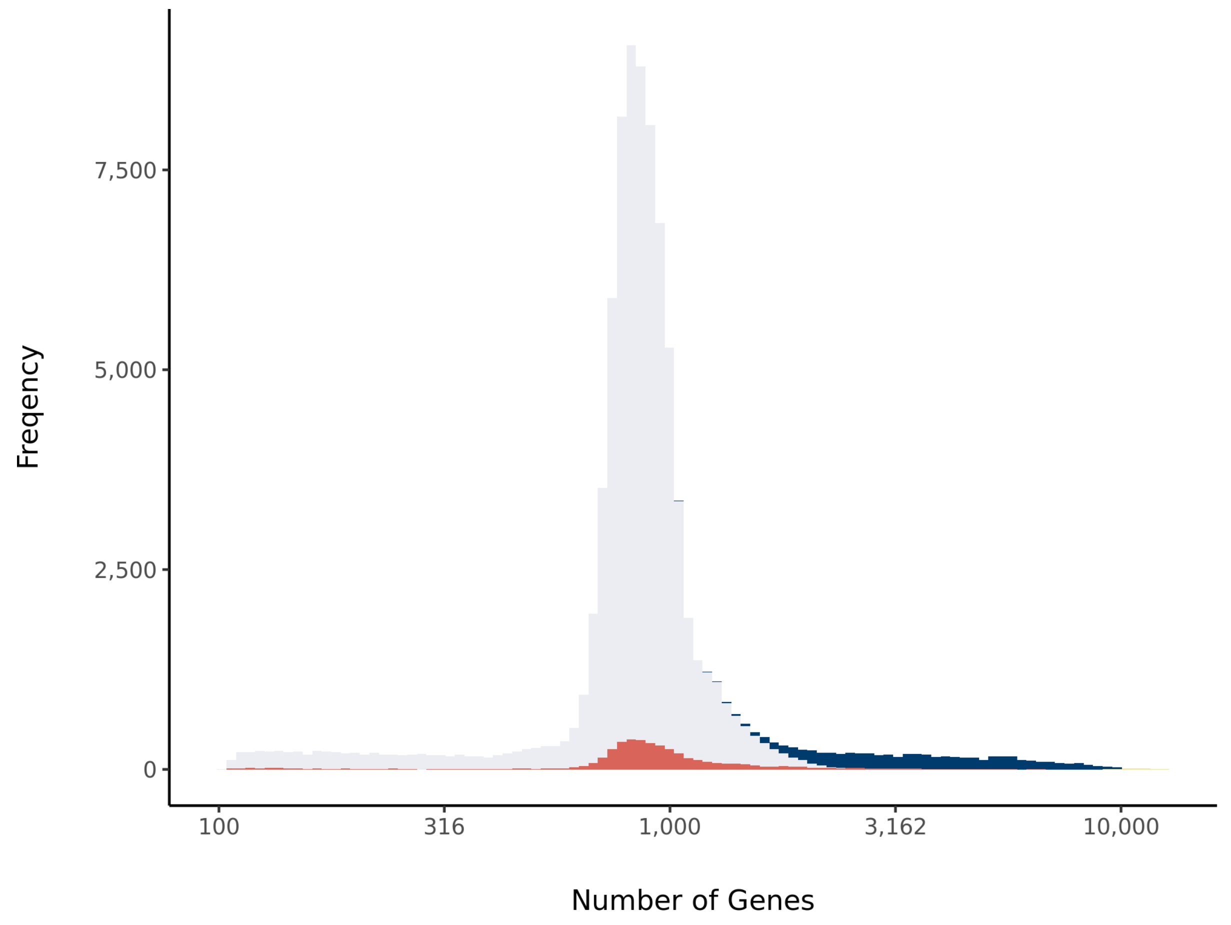

Barcode Rank Plot

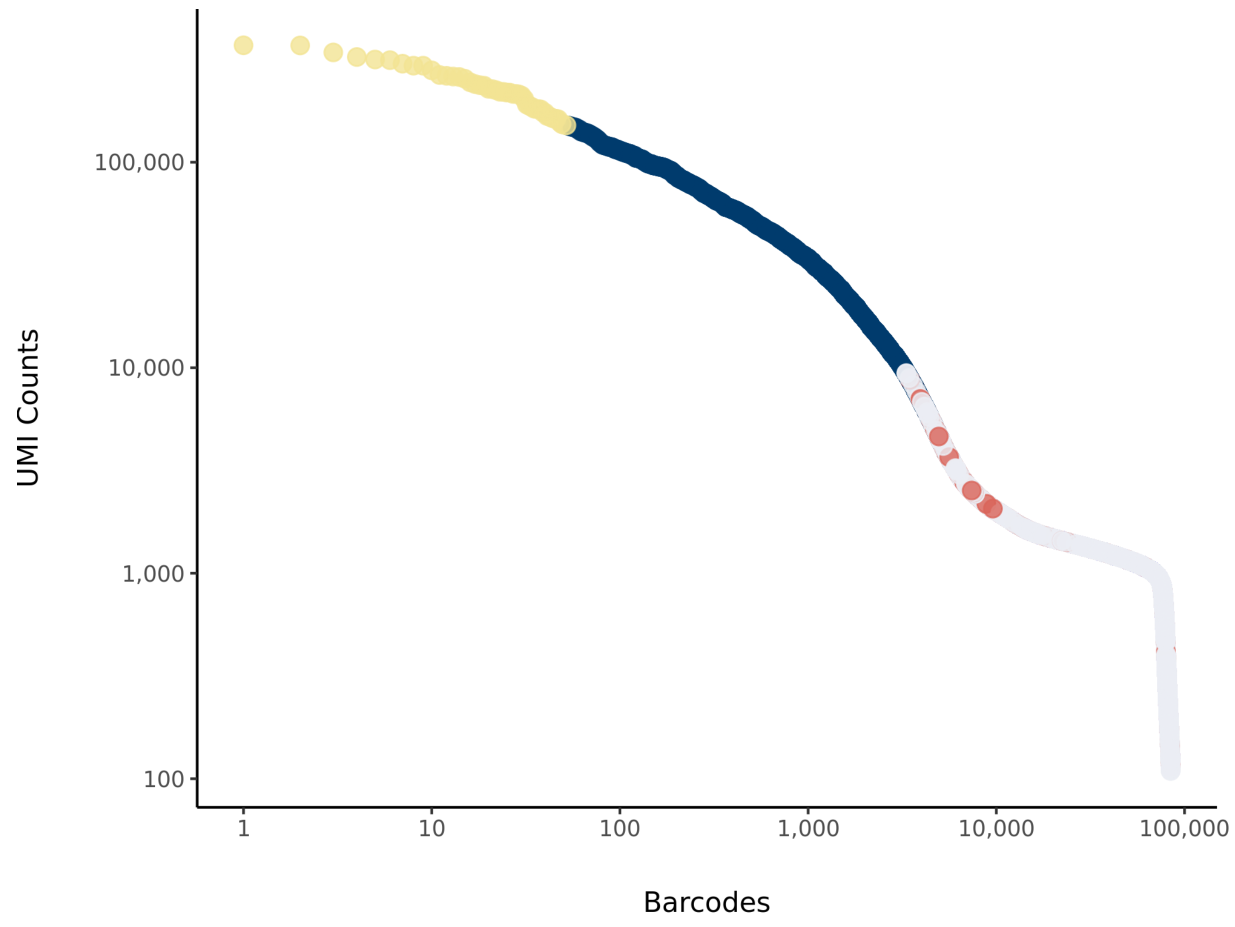

tnw2 Summary

Processed by COPILOT

Summary | Analysis

Parameters

Iteration of Filtering	1
Mitochondrial Expression Threshold	5 %
Top High Quality Cell Filtered	1 %
Doublet Removed	Yes

Cell Stats

Estimated Number of High Quality Cell	4,065
High Quality Cell	5.23 %
Total UMI Counts in High Quality Cell	114,947,259
UMI Counts in High Quality Cell	59.81 %
Median UMI Counts per High Quality Cell	15,794
Median Genes per High Quality Cell	3,557
Total Genes Detected in High Quality Cell	25,145
Cell above Mitochondrial Expression Threshold	14.57 %
Estimated Doublet Rate in High Quality Cell	3.12 %

Sequencing Stats

Number of Reads Processed	481,231,001
Reads Pseudoaligned	93.1 %
Reads on Whitelist	96.28 %
Total UMI Counts	192,190,179
Sequencing Technology	10xv3
Species	Arabidopsis thaliana
Transcriptome	TAIR10

Sample Stats

Sample	tnw2
Name	WT control2
Source	Benfey lab
Genotype	WT Col-0
Transgene	NA
Treatment	Untreated
Age	5_day
Timepoint	4
Rep	2
Target Cells	5,000
Date	2019-10-17
Seq Run	Nolan_6013

UMI Counts Histogram

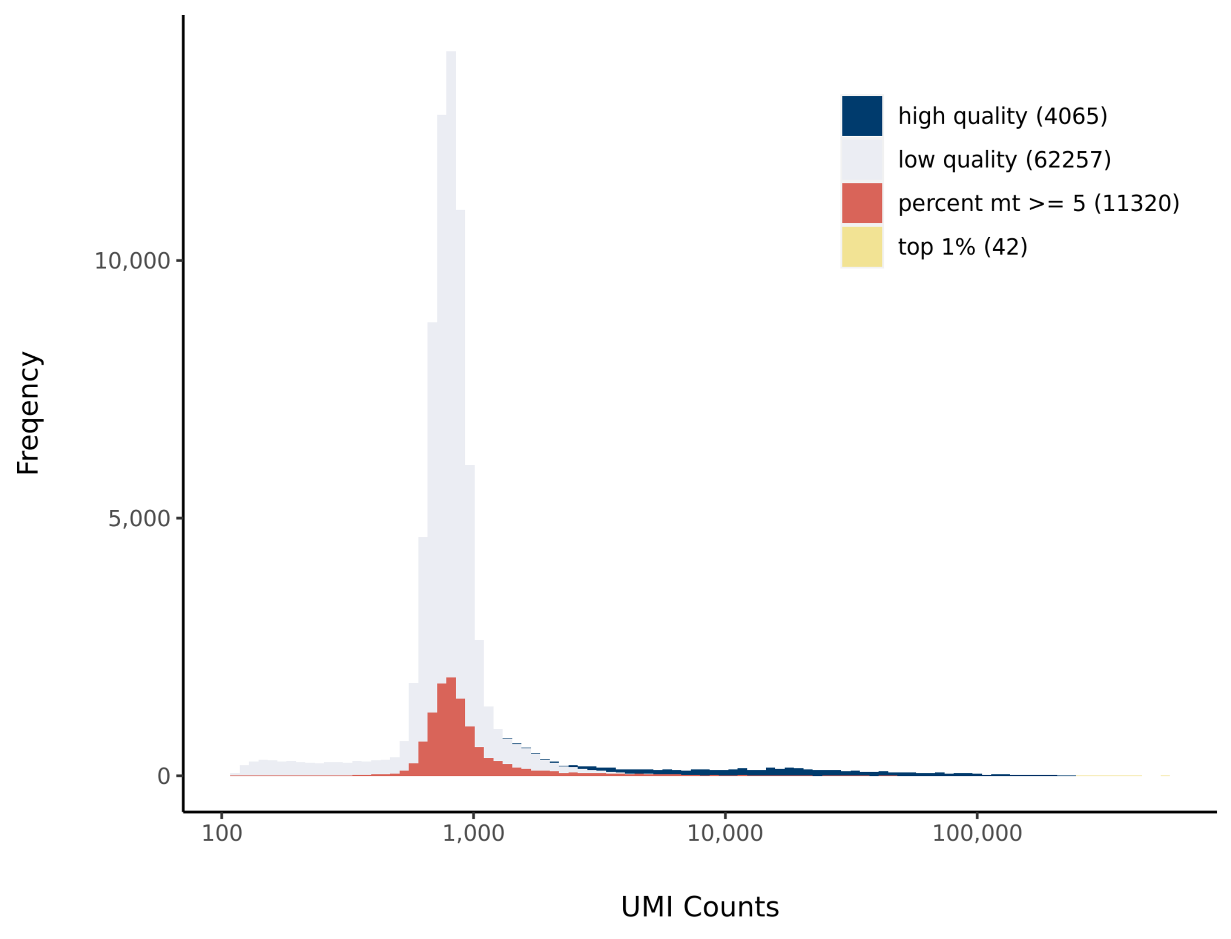

Number of Genes Histogram

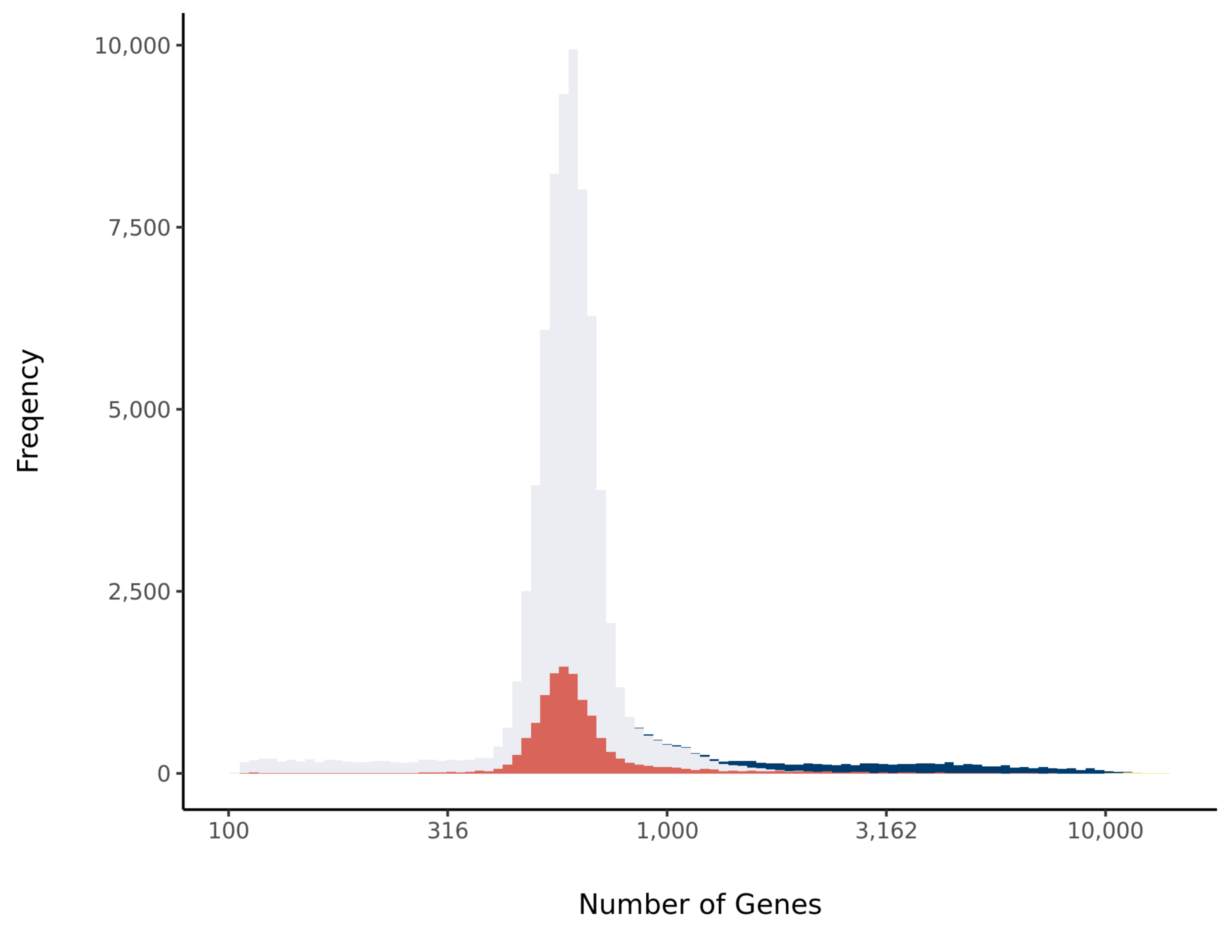

Barcode Rank Plot

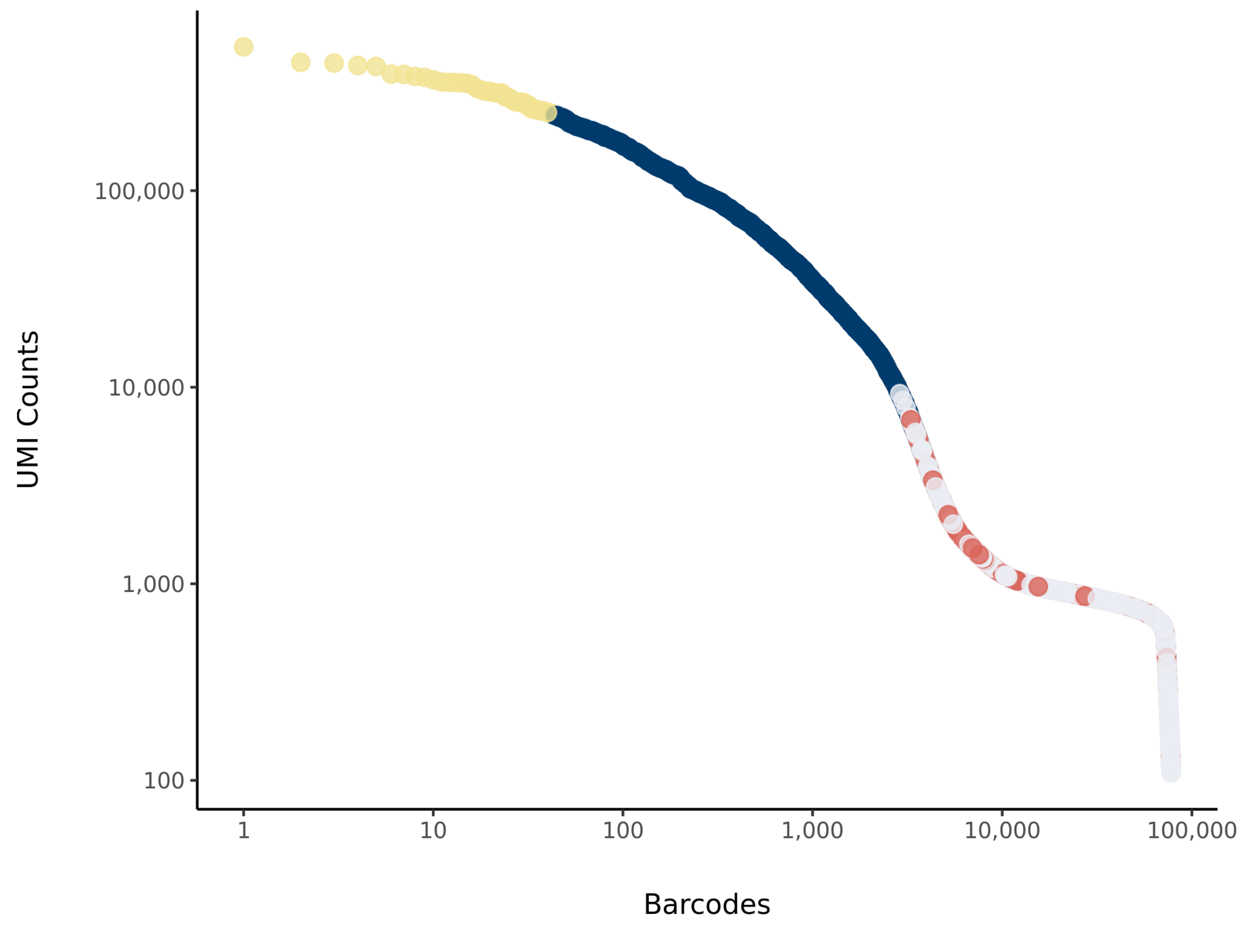

Supplement: 7 [file NIHMS1780093-supplement-7.pdf]
